# A Newly Identified Spike Protein Targeted Linear B‐Cell Epitope Based Dissolvable Microneedle Array Successfully Eliciting Neutralizing Activities against SARS‐CoV‐2 Wild‐Type Strain in Mice

**DOI:** 10.1002/advs.202207474

**Published:** 2023-05-10

**Authors:** Lin Li, Zhongpeng Zhao, Xiaolan Yang, Zhongyi Su, Wendong Li, Shaolong Chen, Lu Wang, Ting Sun, Chen Du, Ziyi Li, Zeqian Yang, Min Li, Tiecheng Wang, Ying Wang, Yubo Fan, Hui Wang, Jing Zhang

**Affiliations:** ^1^ Key Laboratory for Biomechanics and Mechanobiology of Ministry of Education Beijing Advanced Innovation Centre for Biomedical Engineering School of Engineering Medicine and School of Biological Science and Medical Engineering Beihang University Beijing 100083 P. R. China; ^2^ State Key Laboratory of Pathogen and Biosecurity Beijing Institute of Microbiology and Epidemiology Academy of Military Medical Sciences Beijing 100071 P. R. China; ^3^ Institute of Military Veterinary Academy of Military Medical Sciences 666 West Liuying Road Changchun Jilin 130122 P. R. China

**Keywords:** dissolvable microneedle array, linear B‐cell epitope, neutralizing antibody, SARS‐CoV‐2, spike protein

## Abstract

Vaccination is a cost‐effective medical intervention. Inactivated whole virusor large protein fragments‐based severe acute respiratory syndrome coronavirus (SARS‐CoV‐2) vaccines have high unnecessary antigenic load to induce allergenicity and/orreactogenicity, which can be avoided by peptide vaccines of short peptide fragments that may induce highly targeted immune response. However, epitope identification and peptide delivery remain the major obstacles in developing peptide vaccines. Here, a multi‐source data integrated linear B‐cell epitope screening strategy is presented and a linear B‐cell epitope enriched hotspot region is identified in Spike protein, from which a monomeric peptide vaccine (Epitope25) is developed and applied to subcutaneously immunize wildtype BALB/c mice. Indirect ELISA assay reveals specific and dose‐dependent binding between Epitope25 and serum IgG antibodies from immunized mice. The neutralizing activity of sera from vaccinated mice is validated by pseudo and live SARS‐CoV‐2 wild‐type strain neutralization assays. Then a dissolvable microneedle array (DMNA) is developed to pain‐freely deliver Epitope25. Compared with intramuscular injection, DMNA and subcutaneous injection elicit neutralizing activities against SARS‐CoV‐2 wild‐type strain as demonstrated by live SARS‐CoV‐2 virus neutralization assay. No obvious damages are found in major organs of immunized mice. This study may lay the foundation for developing linear B‐cell epitope‐based vaccines against SARS‐CoV‐2.

## Graphical abstract

A novel multisource data integrated linear B‐cell epitope screening strategy identifies a monomeric peptide vaccine candidate (Epitope25) against severe acute respiratory syndrome coronavirus‐2 (SARS‐CoV‐2). A dissolvable microneedle array achieves pain‐free delivery of the Epitope25 and successfully induces neutralizing activities against SARS‐CoV‐2 wild‐type strain in mice, laying the foundation for easy‐operating promising vaccines against SARS‐CoV‐2.

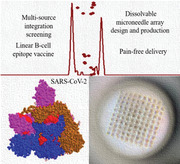

## 1 Introduction

The severe acute respiratory syndrome coronavirus‐2 (SARS‐CoV‐2) are highly contagious and can cause nonsymptomatic infection, mild flu‐like symptoms to pneumonia, severe acute respiratory distress syndrome or even deaths,^[^ ^1^, ^2^ ^]^ posing great threat to human lives.^[^ ^1^, ^3^, ^4^ ^]^ Vaccination is one of the most cost‐effective medical interventions, having already achieved the global eradication of small pox and the virtual eradication of poliomyelitis.^[^ ^5^ ^]^ To combat SARS‐CoV‐2, a variety of vaccines have been or are being developed including viral vector‐based vaccines, mRNA and DNA vaccines, subunit vaccines, nanoparticle‐based vaccines, and inactivated‐whole virus vaccines.^[^ ^6^, ^7^, ^8^, ^9^ ^]^ Side effects of SARS‐CoV‐2 vaccinations are usually mild, of short duration, self‐limiting and ambulatorily manageable,^[^ ^10^, ^11^ ^]^ but in some cases may require hospitalization or even admission to an intensive care unit.^[^ ^11^, ^12^ ^]^ Peptide vaccines have been considered an alternative,^[^ ^13^ ^]^ but there are no human epitope based peptide vaccines on the market yet. It is unprecedentedly meaningful to develop peptide vaccines against SARS‐CoV‐2.

Peptide epitopes are the minimal immunogenic region of a protein antigen, which may elicit a more focused immune response^[^ ^5^ ^]^ and limit significantly the chances for allergenic and/or reactogenic complications.^[^ ^13^ ^]^ However, not all regions of a protein antigen are equally immunogenic.^[^ ^14^ ^]^ The spike (S) protein is the well‐known and wildly investigated antigen for developing therapeutic antibodies and vaccines,^[^ ^15^, ^16^, ^17^ ^]^ as SARS‐CoV‐2 fuses and enters into the host cells through the S1 subunit receptor‐binding domain (RBD) of S protein binding to the angiotensin‐converting enzyme 2 (ACE2) receptor.^[^ ^18^ ^]^ It would be efficient to prevent virus entry through antibodies blocking the binding of S protein to ACE2.^[^ ^19^, ^20^, ^21^ ^]^ Various immune‐informatics methods have been used to predict large quantities of linear B‐cell epitopes with high immunogenicity,^[^ ^22^, ^23^, ^24^, ^25^, ^26^, ^27^, ^28^, ^29^, ^30^, ^31^, ^32^, ^33^, ^34^, ^35^, ^36^, ^37^, ^38^, ^39^, ^40^, ^41^, ^42^, ^43^, ^44^ ^]^ but most epitope candidates are purely in silico predictions without sufficiently solid evidences provided by biological validation experiments. It is difficult to move to the next step by aiming at a limited number of linear B‐cell epitope candidates for peptide vaccine development.

The obstacles that have hampered the clinical use of peptide vaccines are also associated with peptide stability and delivery.^[^ ^13^ ^]^ As there are some carriers and adjuvants that can help counterbalance the low‐molecular nature of oligopeptides, it is now becoming critical to select the appropriate delivery systems for peptide vaccines. Intramuscular immunization is not a good option due to few immune cells residing in muscles. The transdermal peptide delivery has many biological advantages including avoidance of the first‐pass metabolism and sustained therapeutic action,^[^ ^45^ ^]^ although the fear of sharp needles used in subcutaneous injection impedes many people from getting vaccinated. Microneedles can overcome the stratum corneum barrier and are minimally invasive that can drug into or through the skin barrier.^[^ ^45^ ^]^ Microneedles vary between 50 and 900 µm in height and can pain‐freely and effectively deliver vaccines to the epidermis and the dermis region with abundance of antigen presenting cells (APCs) and immune accessory cells.^[^ ^46^ ^]^ The application of microneedles into the skin forms microscopic aqueous pores to allow the diffusion of drugs to the skin's epidermal layer.^[^ ^47^ ^]^ Microneedles have been reported to deliver DNA nanovaccine and successfully induce a robust immune response against SARS‐CoV‐2.^[^ ^48^ ^]^ However, it is not yet clear about the performances of microneedles in delivering peptide vaccines against SARS‐CoV‐2.

To deal with these challenges, we presented a comprehensive linear B‐cell epitope screening strategy, which integrated multiple independent sources of information including predictions from eight widely used immune‐informatics methods, antigenicity, physiochemical properties, toxicity, discontinuous B‐cell epitopes, mutations, and 3D structure of S protein. One hotspot region in RBD region was identified to harbor linear B‐cell epitopes. Specific and dose dependent binding affinity were confirmed between one linear B‐cell epitope (named as Epitope25) in the hotspot region and serum antibodies from the corresponding immunized wild‐type BALB/c mice. Both pseudo and live SARS‐CoV‐2 virus validated the successful induction of neutralizing activities in mice inoculated with Epitope25. We then developed a dissolvable microneedle array (DMNA) to deliver Epitope25. Compared with intramuscular injection, neutralizing antibody titer validated that Epitope25‐DMNA successfully induced neutralizing activities, achieving similar effects as subcutaneous injection of Epitope25 in mice. All Epitope25 immunized mice did not exhibit any side effects, and hematoxylin–eosin (H&E) staining of tissues confirmed no damages in liver and kidney. These results provided multiple layers of evidences about the strong antigenicity of Epitope25 from the hotspot region and also proved that DMNA was an effective and safe delivery method for peptide vaccine, suggesting that Epitope25‐DMNA would be an important candidate for the development of peptide vaccine.

## 2 Results

### 2.1 Identification of Potential Hotspot Regions Harboring Linear B‐Cell Epitopes on the SARS‐CoV‐2 Spike Protein

**Figure 1:**
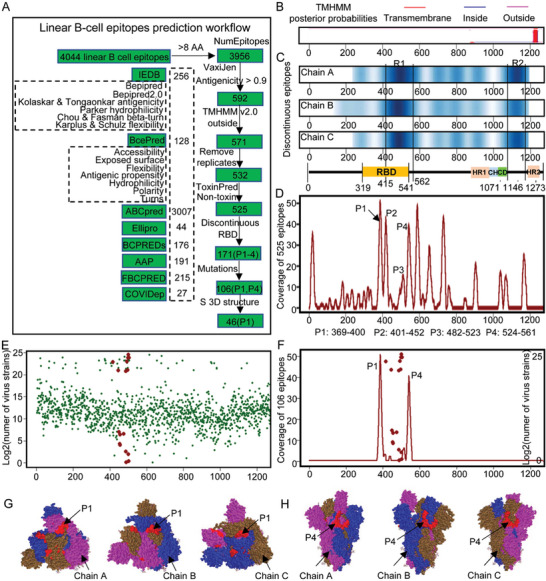
Identification of linear B‐cell epitope hotspot regions in the spike protein of SARS‐CoV‐2. A) The linear B‐cell epitope prediction workflow. B) The transmembrane topology of SARS‐CoV‐2 S protein predicted by TMHMM v2.0. Pink is transmembrane (1214–1236), blue is inside (1237–1273), and magenta is outside (1–1213). The *y*‐axis is posterior probabilities. C) The distribution of discontinuous B‐cell epitopes predicted for chain A, B, and C, respectively. D) The positions of the filtered 525 high antigenic linear B‐cell epitopes on the S protein amino acid sequence. The *y*‐axis is the coverage of linear B‐cell epitopes. The regions with coverage of epitopes larger than 10 were determined as the peaks. E) The distribution of mutations on the S protein. The *y*‐axis is the logarithmic transformation of the number of SARS‐CoV‐2 mutant strains. The *x*‐axis is the position of S protein amino acid sequence. Green dot is mutation. Red dot is the mutation on the key amino acids critical for protein interactions based on NGDC (December 2, 2022). F) The RBD domain overlapped peaks and critical mutation for protein interaction. The *x*‐axis is the position of S protein amino acid sequence. The curves are the coverage of linear B‐cell epitopes measured by the *y*‐axis on the left. The red dots are critical mutations for protein interactions measured by the logarithmic transformation of the number of SARS‐CoV‐2 mutant strains as indicated by the *y*‐axis on the right. G,H) The localizations of the linear B‐cell epitope hotspot regions of Peak 1 (G) and Peak 2 (H) mapped on 3D structure of SARS‐CoV‐2 S (PDB ID: 6VSB) protein. Magenta is chain A, blue is chain B, brown is chain C. Red is linear B‐cell hotspot regions.

Spike protein is an important target for vaccine development due to its indispensable function in helping SARS‐CoV‐2 entry into host cells. B‐cells can be guided through linear B‐cell epitopes to recognize and activate defense responses against viral infection. To construct a comprehensive linear B‐cell epitope candidate list, we first performed in silico prediction of B‐cell epitopes from S protein through eight methods, obtaining a total of 4044 linear B‐cell epitope candidates (256 epitopes for Bepipred and Bepipred2.0 with default parameter settings, Kolaskar and Tongaonkar antigenicity, Parker hydrophilicity, Chou and Fasman beta‐turn, and Karplus and Schulz flexibility provided by IEDB (Immune‐Epitope‐Database and Analysis‐Resource);^[^ ^49^ ^]^ 128 epitopes for BcePred^[^ ^50^ ^]^ using accessibility, antigenic propensity, exposed surface, flexibility, hydrophilicity, polarity, and turns; 3007 epitopes for the ANNpred‐based server ABCpred;^[^ ^51^ ^]^ 44 epitopes for Ellipro;^[^ ^52^ ^]^ 176 epitopes for BCPREDS;^[^ ^53^ ^]^ 191 epitopes for AAP;^[^ ^54^ ^]^ 215 epitopes for FBCPRED;^[^ ^53^ ^]^ and 27 epitopes for COVIDep^[^ ^55^ ^]^) (**Figure** **1**A; Table S1, Supporting Information).

To obtain linear B‐cell epitopes with high potential to initiate a defensive immune reaction, we adopted a series of high stringent criteria to filter out epitopes with low antigenicity (Figure 1A). B‐cell epitope candidates with less than eight amino acids were removed with 3956 epitopes remained. 592 epitopes were filtered to have high antigenicity scores estimated by VaxiJen 2.0^[^ ^56^ ^]^ (larger than 0.9 viewed adequate to initiate a defensive immune reaction) (Table S2, Supporting Information). The positions of the 592 linear B‐cell epitopes on the S protein amino acid sequence were examined to exclude the epitopes locating on the non‐outer surface based on the transmembrane topology of SARS‐CoV‐2 S protein predicted by TMHMM v2.0 (outside: 1–1213; transmembrane: 1214–1236; inside: 1237–1273), resulting in 571 epitopes (Figure 1B; Table S2, Supporting Information). We obtained 532 unique epitopes after removing duplicated epitopes predicted from different methods. 525 of 532 epitopes classified by ToxinPred^[^ ^57^ ^]^ as nontoxin were remained (Table S3, Supporting Information). Discontinuous B‐cell epitope residues predicted by Discotope 2.0 using A, B, and C chain of the 3D structure of S protein (PDB ID: 6VSB) (Table S4, Supporting Information) frequently appeared in two regions of S protein (R1: AA 415–562 and R2: AA 1071–1146). R1 essentially overlapped with RBD domain (AA 319–541) and R2 with regions between connector domain (CD) and heptad repeat 2 (HR2) (Figure 1C), providing another evidence that RBD may harbor highly antigenic B‐cell epitopes. The distribution of the 525 linear B‐cell epitopes along the S protein revealed a few hotspot regions (exhibiting as peaks), where a large number of B‐cell epitopes with varying length predicted by different algorithms converged (Figure 1D). Particularly, there were four hot regions predominantly overlapped with RBD domains including P1 (AA 369–400), P2 (AA 401–452), P3 (AA 482–523), and P4 (524–561) (Figure 1D), which included 171 linear B‐cell epitopes (Table S5, Supporting Information).

RBD is not an evolutionarily conserved region, which was confirmed by the conservation status of each residue on the S protein predicted by ConSurf using seven known coronaviruses including SARS‐CoV‐2, SARS‐CoV, MERS‐CoV, alpha coronavirus 229E, alpha coronavirus NL63, beta coronavirus OC43, and beta coronavirus HKU1. To investigate whether there are some B‐cell epitopes from regions that are resistant to mutations, we examined the distribution of mutations along the S protein based on the 2019 Novel Coronavirus Resource (2019nCoVR) released by the China National Center for Bioinformation, where a total of 28 170 mutations of 3816 sites are identified in S protein, of which, 7430 lead to amino acid changes. We discovered that all amino acid residues on the S protein had variants detected in a large number of virus strains (Figure 1E), revealing that SARS‐Cov‐2 has been universally mutating to increase fitness and spread quickly under immune pressure. However, there were only a few key amino acids critical for protein interactions (Figure 1E), which influenced 65 of 171 linear B‐cell epitopes mainly locating in P2 and P3 hotspot region (Figure 1F; Table S5, Supporting Information). P1 and P4 hotspot regions mapped to the 3D structure of the SARS‐CoV‐2 S protein (PDB ID: 6VSB) demonstrated that P1 located on the exposed area of spike head which was the area interacting with ACE2 (Figure 1G) and P4 on the outside of spike stem (Figure 1H). These results suggested that P1 hotspot region have large potentials to generate highly antigenic linear B‐cell epitopes (Table S5, Supporting Information), which could induce antibodies to block SARS‐CoV‐2 entry into cells.

### 2.2 Epitope25 from Hotspot Region Inducing Neutralizing Activities against SARS‐CoV‐2

To test whether the hotspot region (P1, AA 369–400) may harbor antigenic linear B cell epitopes, we selected the longest epitope of “YNSASFSTFKCYGVSPTKLNDLCFT” (named as Epitope25) from the 46 B‐cell epitopes in P1. Epitope25 exhibited good stability as it was unable to be cleaved by 24 of 37 enzymes based on prediction of PeptideCutter in Expasy. Epitope25 was also predicted as nontoxin and not causing allergenicity, although animal models are necessary to demonstrate efficacy and safety in the development of vaccines against SARS‐CoV‐2 infection.^[^ ^58^, ^59^ ^]^

**Figure 2:**
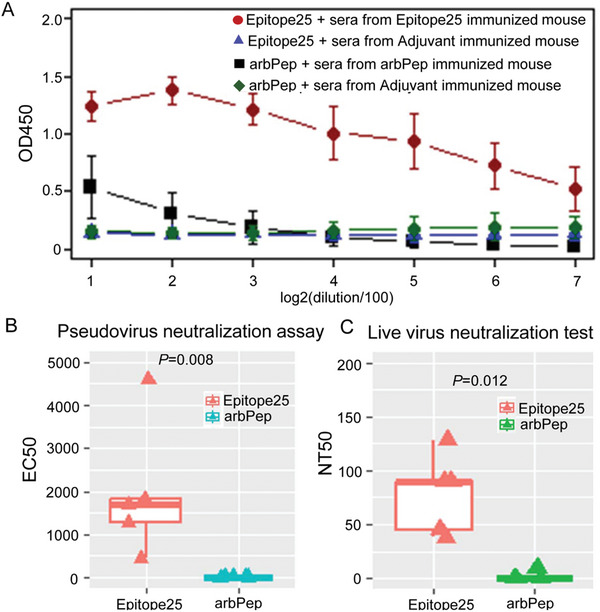
Epitope25 from hotspot regions inducing neutralization activity against both pseudo and live SARS‐CoV‐2 wildtype strain. A) The binding affinity assessed by indirect ELISA between linear B‐cell epitope of “YNSASFSTFKCYGVSPTKLNDLCFT” (Epitope25) and serum antibodies from immunized mice with Epitope25. The *x*‐axis is the dilution; *y*‐axis is the value of OD450. Red dot is the binding affinity between Epitope25 and sera from mice immunized with Epitope25, blue triangle is the binding affinity between Epitope25 and sera from mice immunized with Al(OH)3 Adjuvant, black square is the binding affinity between arbitrary peptide of “RRRRRRRRRRRRRRRR” (arbPep) and sera from mice immunized with arbPep, and green diamond is the binding affinity between arbPep and sera from mice immunized with Al(OH)3 Adjuvant. There are five mice in each group. B) The viral neutralization activity of the sera from Epitope25 or arbPep immunized mice as challenged in vitro with a SARS‐CoV‐2 pseudovirus. The *y*‐axis is EC_50_. Red is the sera from mice immunized with Epitope, turquoise is the sera from mice immunized with arbPep. There are five mice per group. Mann–Whitney *U* test was used to calculate the *P*‐value. *P*‐value ≤ 0.05 was determined as statistical significance. C) The neutralizing activity of sera from vaccinated mice with Epitope25 or arbPep against SARS‐CoV‐2 live virus (BetaCoV/Beijing/IMEBJ01/2020). The *y*‐axis is NT_50_. Red is the sera from mice immunized with Epitope, green is the sera from mice immunized with arbPep. There are five mice per group. Mann–Whitney *U* test was used to calculate the *P*‐value. *P*‐value ≤ 0.05 was determined as statistical significance.

To assess the binding affinity of Epitope25 with serum IgG antibodies against SARS‐CoV‐2, we used BALB/c mice, which are a good animal model for investigating SARS‐CoV‐2 infection in both upper and lower respiratory tracts.^[^ ^6^ ^]^ We immunized 10 six‐ to eight‐week‐old female BALB/c mice through five consequent subcutaneous injections of 5 µg dose of synthetic peptides of Epitope25 or one arbitrary control peptide (“RRRRRRRRRRRRRRRR”, named as arbPep) with 7 days interval between two consecutive injections (five mice per peptide). We also immunized another two groups of BALB/C mice (*n* = 5 mice per group) with Al(OH)3 adjuvant as a control for Epitope25 and arbPep groups, respectively. The binding affinities between each peptide and sera collected from each immunized mouse were assessed by indirect ELISA in triplicate. We discovered that Epitope25 reacted specifically and dose dependently with antibodies in sera from mice immunized with synthetic peptide of Epitope25 but not in sera from mice injected with arbPep or Al(OH)3 adjuvant (**Figure** **2**A). It was evident that a substantial percentage of antibodies were generated in vaccinated mice against Epitope25, suggesting that antibodies directed at Epitope25 could bind to SARS‐CoV‐2.

Pseudovirus neutralization assay is a sensitive and quantitative method as demonstrated in studies of SARS‐CoV and MERS‐CoV.^[^ ^60^ ^]^ In an attempt to measure SARS‐CoV‐2 to neutralization elicited by vaccination of Epitope25 in mice, we tested the viral neutralization activity of the sera from Epitope25 or arbPep immunized mice as challenged in vitro with a SARS‐CoV‐2 pseudovirus using a pseudotyped virus‐based neutralization assay developed recently for SARS‐CoV‐2,^[^ ^61^ ^]^ respectively. A high level of activity was observed in all five mice immunized with Epitope25 (half maximal effective concentration, EC50: mean value is 1963. 34) in a sharp contrast to the none observed in arbPep vaccinated mice (*p* = 0.008) (Figure 2B).

We next assessed the neutralizing activity of sera from vaccinated mice with Epitope25 or arbPep against SARS‐CoV‐2 live virus (BetaCoV/Beijing/IMEBJ01/2020), respectively. Epitope25 exhibited significantly stronger neutralization activity (half maximum neutralization titer, NT50: mean value is = 78.2) than arbPep, which had mean NT50 value close to zero (*p* = 0.012) (Figure 2C). These results demonstrated that Epitope25 was able to induce neutralizing activities against SARS‐CoV‐2 wild type strain, suggesting that it would be an important candidate for the development of peptide vaccine.

### 2.3 Construction of Epitope25 Based Dissolvable Microneedle Array (Epitope25‐DMNA)

The development of vaccines is not only relying on epitope identification but also depending on vaccine delivery system. The fear of painful needles used in intramuscular or subcutaneous injection is an important reason that many people are reluctant to getting vaccinated. Microneedle array with needles ranging from 50 to 900 µm offers a pain‐free method of effectively delivering vaccine to the epidermis and the dermis region, which contains abundance of APCs and immune accessory cells.^[^ ^46^ ^]^

**Figure 3:**
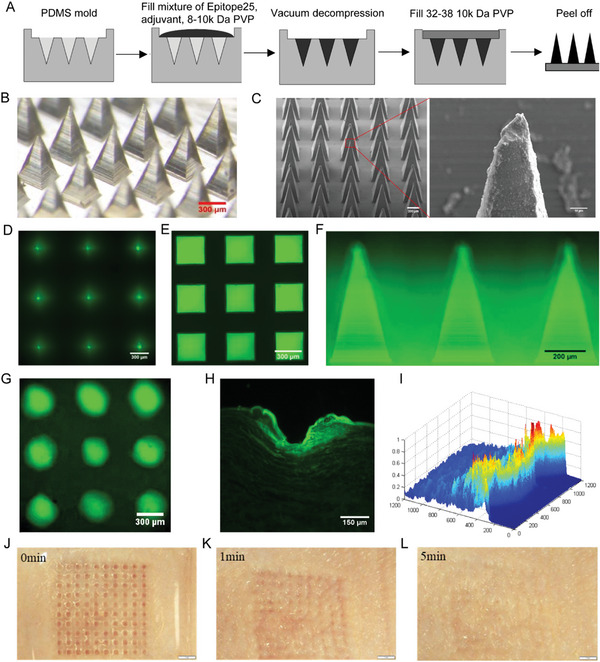
Schematic illustration and characteristics of DMNA. A) The fabrication workflow of DMNA. B,C) The images of microneedles on the DMNA using B) stereo microscope and C) scanning electron microscope. D,E) The green fluorescence image of microneedles on the DMNA using fluorescence microscope with (D) showing the tips of the microneedles, (E) the bottom of the microneedles and F) side‐view of the microneedles. G,H) The green fluorescence across the porcine skin following microneedle penetration from the top‐view (G) and side‐view (H), respectively. I) The distribution of green fluorescence intensities along the microscopic aqueous pores formed in the skin after microneedle penetration. J–L) The images of the skin of Sprague Dawley (SD) rat using lab‐made fisheye camera system at time of 0 min (J), 1 min (K), and 5 min (L) following DMNA penetration.

In this study, we developed a dissolvable microneedle array to deliver Epitope25 (named as Epitope25‐DMNA) to intradermal layer for effective vaccination. Epitope25‐DMNA was fabricated (**Figure** **3**A) to be composed of a water‐soluble backing layer made of 300 µL 32–38 × 10 kDa polyvinylpyrrolidone (PVP) (0.5 mg µL^−1^) and a 10 × 10 array of sharp and quadrangular pyramid shape microneedles (Figure 3B,C). The height of the microneedle was ≈700 µm and the length of each side of the bottom was ≈300 µm (Figure 3D–F). The microneedles in one array was made of a 2 µL mixture of Epitope25 (2.5 µg µL^−1^), aluminum hydroxide adjuvant (5 µg µL^−1^), and 8–11× kDa PVP (0.5 mg µL^−1^). Once Epitope25‐DMNA was pushed into skin, the microneedles began to dissolve quickly. The backing layer also began to dissolve from the side near skin in a slower speed due to the larger molecular weight of 32–38 × 10 kDa PVP, which would form a liquid thin film in 5–10 min. The liquid film was not only able to prevent the dissolved liquid mixture in the microneedles from seeping out of skin but also to make the backing layer be easily separated from skin.

We next used porcine skin as model to evaluate the skin penetration capability of DMNA with green fluorescence added in the microneedles. The successful insertion and formation of microconduits were observed in the fluorescence images of skin after the backing layer was removed (Figure 3G,H), indicating DMNA were able to deliver the peptide vaccine into the intradermal layer without damaging the nerves and vascular structures. The distribution of fluorescence showed that the mixture in the microneedles could diffuse into skin at depth of 400–600 µm surrounding the microneedles (Figure 3I). We also confirmed that DMNA were able to form microconduits in the skin of Sprague Dawley (SD) rat (Figure 3J). The microneedles gradually dissolved (Figure 3K) and completely released into the skin in 5 min (Figure 3L). The skin of the SD rat was also restored to its original shape, demonstrating that DMNA would not cause damages to the skin.

### 2.4 Epitope25‐DMNA Inducing Neutralizing Activities against SARS‐CoV‐2

**Figure 4:**
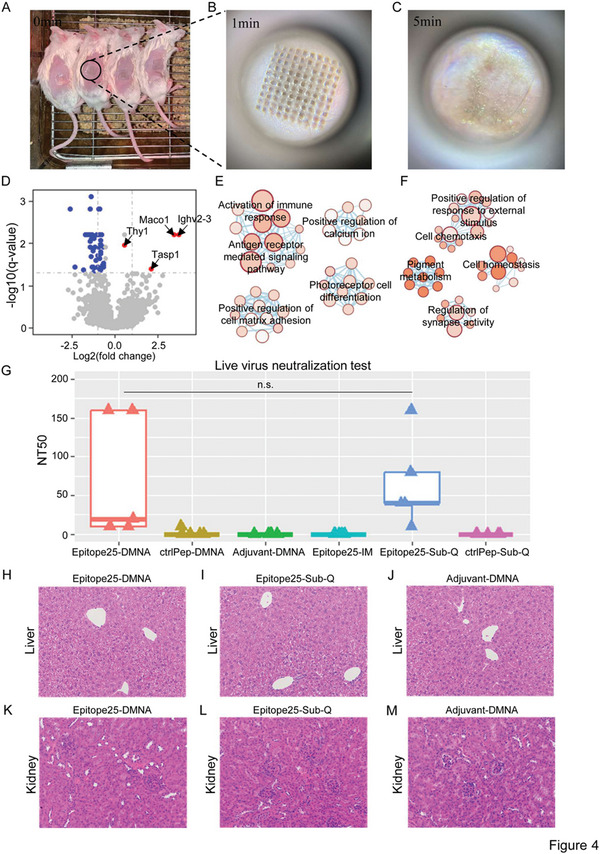
The neutralizing activities against live SARS‐CoV‐2 wild‐type strain induced by Epitope25‐DMNA. A–C) The images of microneedles in the skin of immunized mice with Epitope25‐DMNA (A) using lab‐made fisheye camera system at time of 1 min (B), and C) 5 min (C) following DMNA penetration. D) Differential expressed genes identified in PBMCs between mice immunized with Epitope25‐DMNA and those with Adjuvant‐DMNA. Red and blue is significantly up‐ and downregulated genes in PBMCs of mice immunized with Epitope25 compared with those with Adjuvant‐DMNA. *Q*‐value ≤ 0.05 and fold change ≥1.5 were deemed as significantly differentially expressed. The *x*‐axis is the logarithmic transformation of fold change. The *y*‐axis is the logarithmic transformation of *q*‐value. E,F). The enriched gene ontology terms among E) upregulated genes and F) downregulated genes in PBMCs of mice immunized with Epitope25 compared with those with Adjuvant‐DMNA. G) The neutralizing activity of sera from vaccinated mice with Epitope25‐DMNA, ctrlPep‐DMNA, Adjuvant‐DMNA, Epitope25‐IM, Epitope25‐Sub‐Q, or ctrlPep against SARS‐CoV‐2 live virus (BetaCoV/Beijing/IMEBJ01/2020). The *y*‐axis is NT_50_. There are five mice per group. Mann–Whitney *U* test was used to calculate the *P‐*value. *P*‐value ≤ 0.05 was determined as statistical significance. ctrlPep is “VKPSFYVYSRVKNLN” randomly selected from envelope protein of SARS‐CoV‐2. IM is intramuscular injection. Sub‐Q is subcutaneous injection. H–J) The H&E staining images of livers from sacrificed mice immunized with Epitope25‐DMNA (H), Epitope25‐Sub‐Q (I), and Adjuvant‐DMNA (J), respectively. (K–M) The H&E staining images of kidneys from sacrificed mice immunized with Epitope25‐DMNA (K), Epitope25‐Sub‐Q (L), and Adjuvant‐DMNA (M), respectively.

To evaluate the performance of Epitope25‐DMNA in inducing neutralizing activities against SARS‐CoV‐2, we also constructed dissolvable microneedle arrays for aluminum hydroxide adjuvant (Adjuvant‐DMNA), and “VKPSFYVYSRVKNLN”, which was randomly selected from envelope protein of SARS‐CoV‐2 and used as control peptide (ctrlPep‐DMNA), respectively. Another two major delivery approaches were also included for the purpose of comparison including intramuscular injection (IM) and subcutaneous injection (Sub‐Q). We therefore immunized 30 six‐ to eight‐week‐old female BALB/c mice through five consequent applications of Epitope25‐DMNA, Adjuvant‐DMNA, ctrlPep‐DMNA, Epitope25‐IM, Epitope25‐Sub‐Q, or ctrlPep‐Sub‐Q with 7 days interval between two consecutive applications (five mice per immunization method). As shown as an example, the microneedles of Epitope25‐DMNA in immunized mice (**Figure** **4**A) gradually dissolved (Figure 4B) until complete release into the skin in 5 min (Figure 4C).

To determine whether Epitope25‐DMNA could elicit immune response in vivo, we performed RNA sequencing (RNA‐seq) of the peripheral blood mononuclear cells (PBMCs) collected from each mouse immunized with Epitope25‐DMNA, Epitope25‐Sub‐Q, and Adjuvant‐DMNA, respectively. Differential gene expression analysis revealed that four genes (Ighv2‐3, Thy1, Tasp1, and Maco1) were statistically significantly upregulated and 49 genes downregulated in Epitope25‐DMNA compared with Adjuvant‐DMNA (Figure 4D; Table S6, Supporting Information). Notably, Ighv2‐3 (immunoglobulin heavy variable 2–3) was to enable antigen binding activity and immunoglobulin receptor binding activity based on gene information in NCBI. The upregulation of Ighv2‐3 in Epitope25‐DMNA group suggested that the humoral immunity process was initiated in mice by Epitope25. Gene Ontology Enrichment analysis demonstrated that the four upregulated genes in Epitope25‐DMNA group were mainly enriched in activation of immune response (*q*‐value = 0.007) and antigen receptor‐mediated signaling pathway (*q*‐value = 0.006) (Figure 4E; Table S7, Supporting Information) with those downregulated genes enriched in pigment metabolism (*q*‐value = 0.00006), and cell homeostasis (*q*‐value = 0.0001) (Figure 4F; Table S8, Supporting Information). Similarly, immune response associated pathways were also observed significantly enriched among upregulated genes in mice immunized with Epitope25‐Sub‐Q compared with Adjuvant‐DMNA (Tables S9–S11, Supporting Information). RNAseq data provided important evidences that both Epitope25‐DMNA and Epitope25‐Sub‐Q successfully activated immune system, and therefore we hypothesized that there could be neutralizing activities induced by Epitope25‐DMNA or Epitope25‐Sub‐Q in mice against SARS‐CoV‐2.

We next assessed and compared the neutralizing activities of sera against SARS‐CoV‐2 live virus (BetaCoV/Beijing/IMEBJ01/2020) across the six groups of mice. Epitope25‐DMNA achieved similar neutralization activity as Epitope‐Sub‐Q (mean NT50 = 72 for Epitope‐DMNA, mean NT50 = 66 for Epitope‐Sub‐Q) in contrast to Epitope25‐IM and three control groups (Adjuvant‐DMNA, ctrlPep‐DMNA, and ctrlPep‐Sub‐Q) which failed to induce neutralizing activities against SARS‐CoV‐2 (Figure 4G). These results independently confirmed again that Epitope25 was able to induce neutralizing activities against SARS‐CoV‐2 as we observed during Epitope25 identification. It also clearly showed that both dissolvable microneedle array and subcutaneous injection were effective approaches to deliver Epitope25, but intramuscular injection was not good vaccine delivering method as there was generally lack of immune cells in muscles.

In addition, all mice in any of the six groups were not observed to have aberrant behavior, weight loss, and side‐effects, although we still performed hematoxylin–eosin staining of tissues from major organs including kidneys and liver of mice immunized with Epitope25‐DMNA, mainly considering that the kidneys and liver are the principal organs involved in drug and xenobiotic metabolism. None of evident tissue injuries, abnormal blood clots and aberrant infections were observed in liver (Figure 4H–J) and kidneys (Figure 4K–M). Altogether, Epitope25‐DMNA was safe and effective in inducing neutralizing activities against SARS‐CoV‐2 virus, suggesting that it would be an important candidate for the development of peptide vaccine.

## 3 Discussion

SARS‐CoV‐2 has caused a serious pandemic all over the world. Multiple vaccines against SARS‐CoV‐2 have been urgently developed worldwide including viral vector‐based vaccines, mRNA and DNA vaccines, subunit vaccines, nanoparticle‐based vaccines, to inactivated‐whole virus vaccines,^[^ ^6^, ^7^, ^8^, ^9^, ^62^ ^]^ but various side effects are still observed and sporadically reported. Ongoing efforts are needed for developing new vaccine categories. An alternative option is peptide vaccines,^[^ ^13^ ^]^ but peptide identification, peptide stability and delivery are the major obstacle that hinder its development, leading to no human epitope based peptide vaccines on the market yet. It is extremely meaningful to develop peptide vaccines against SARS‐CoV‐2. Moreover, the technologies used in peptide vaccine development against SARS‐CoV‐2 can also be applied in other potential scenarios.

S protein is an important target in vaccine development for its critical functions for SARS‐CoV‐2 to fuse and enter into the host cells,^[^ ^15^, ^16^, ^17^ ^]^ but not all regions in the S protein are equally immunogenic. A large number of predicted linear B‐cell epitopes are generated within a highly accelerated time frame,^[^ ^22^, ^23^, ^24^, ^25^, ^26^, ^27^, ^28^, ^29^, ^30^, ^31^, ^32^, ^33^, ^34^, ^35^, ^36^, ^37^, ^38^, ^39^, ^40^, ^41^, ^42^, ^43^, ^44^ ^]^ but the selection of in silico prediction methods is based on the experiences and preferences of each individual researcher. The large quantity of predicted linear B‐cell epitopes lack solid evidences from biological validation experiments to support their antigenicity, making the development of epitope‐based peptide vaccine stagnant.

In this study, we presented a comprehensive linear B‐cell epitope screening workflow by integrating predictions from eight widely used immune‐informatics methods (Bepipred and Bepipred2.0 with default parameter settings, Kolaskar and Tongaonkar antigenicity, Parker hydrophilicity, Chou and Fasman beta‐turn, and Karplus and Schulz flexibility provided by IEDB,^[^ ^49^ ^]^ BcePred^[^ ^50^ ^]^ using accessibility, antigenic propensity, exposed surface, flexibility, hydrophilicity, polarity, and turns, ANNpred‐based server ABCpred,^[^ ^51^ ^]^ Ellipro,^[^ ^52^ ^]^ BCPREDS,^[^ ^53^ ^]^ AAP,^[^ ^54^ ^]^ FBCPRED,^[^ ^53^ ^]^ and COVIDep^[^ ^55^ ^]^) with a serious of information including antigenicity, physiochemical properties, toxicity, discontinuous B‐cell epitopes, mutations, and 3D structure of S protein. We found an important hotspot region, which harbor a large number of varying length linear B‐cell epitopes predicted from different methods. To the best of our knowledge, the linear B‐cell epitope candidate list we presented is one of the most comprehensive and valuable sources for developing peptide vaccines.

We next explored the antigenicity of the longest linear B cell epitope having 25 amino acids (Epitope25). Indirect ELISA assay of Epitope25 and serum antibodies from five mice subcutaneously immunized with synthetic peptide of Epitope25 confirmed that Epitope25 was able to induce Epitope25 peptide specific antibodies in mice, in contrast to the failure of the arbitrary control peptide. A pseudotyped virus‐based neutralization assay validated the high viral neutralization activity of the sera from Epitope25 immunized mice as challenged in vitro with a SARS‐CoV‐2 pseudovirus. Moreover, live virus neutralization titer assay provided another layer of solid evidence about the induced neutralizing activity of sera from vaccinated mice with Epitope25 against SARS‐CoV‐2 live virus (BetaCoV/Beijing/IMEBJ01/2020). These results demonstrated that Epitope25 was able to induce neutralizing activities against SARS‐CoV‐2 wild type strain in mice. We also noticed that the hotspot region does not include any mutations critical for ACE2 binding, suggesting that Epitope25 would also be able to induce neutralizing activities against mutated SARS‐CoV‐2 such as omicron subvariants. It needs further detailed examination of the efficacy of Epitope25 against a variety of SARS‐CoV‐2 variants. Additionally, Epitope25 is a monomer having 25 amino acids, although it was able to induce neutralizing activities. Peptide vaccines consisting of multiple peptides would significantly increase neutralizing antibodies titers, which would deserve further exploration.

How to deliver peptide vaccine is also critical in keeping both the vaccine effects and vaccination willingness. The fear of sharp needles seems inherent for many people. As microneedles vary between 50 and 900 µm in height and can pain‐freely and effectively deliver vaccine to the epidermis and the dermis region, microneedle arrays would be ideal option for vaccination. We assessed the vaccination effects through microneedles by fabricating dissolvable microneedle arrays (DMNAs) to deliver Epitope25 in mice. Epitope25‐DMNA elicited neutralizing activities in immunized mice, achieving similar effects as subcutaneous injection of Epitope25. The neutralizing tiers of mice vaccinated with Epitope25 through subcutaneous injection or microneedle delivery ranged from 10 to 160 (median value: 20 for Epitope25‐DMNA, median value: 40 for Epitope25‐Sub‐Q), similar to that detected in healthy human subjects receiving two doses of inactivated COVID‐19 vaccines (median value: 16 for Sinovac CoronoVac and Sinopharm BBIBP‐CorV vaccines).^[^ ^63^ ^]^ RNA sequencing of PBMC from vaccinated mice revealed that humoral immunity and T‐cell associated immune response were enriched in genes upregulated in those immunized with Epitope25‐DMNA and Epitope25‐Sub‐Q compared with Adjuvant‐DMNA, confirming the activation of pathways associated neutralizing antibody generation. It would need further investigation about whether there are T‐cell epitopes included in Epitope25 and how these T‐cell epitopes (if there are any) and Aluminum hydroxide (adjuvant) would individually and potentially synergistically contribute to the induction of T‐cell associated immune response. Furthermore, the body weights of mice were stable, and H&E staining confirmed that no obvious damages were in major organs collected from immunized mice. These data suggest that Epitope25‐DMNA developed in this study is effective and safe for in vivo treatment. Epitope25‐DMNA would be assessed in nonhuman primate models in the future to further confirm its effectiveness and safety.

In summary, we developed a tailor‐made linear B‐cell epitope screening workflow, through which a hotspot region was newly identified in RBD region of S protein. The hotspot region harbored a large number of linear B‐cell epitopes with varying number of amino acids predicted by different immune‐informatics methods. The longest predicted linear B‐cell epitope with 25 amino acids (Epitope25) in the hotspot region was selected as the monomeric peptide vaccine against SARS‐CoV‐2. Indirect ELISA assay confirmed that Epitope25 reacted specifically and dose‐dependently to bind to serum antibodies in mice subcutaneously immunized with Epitope25. Both pseudo and live SARS‐CoV‐2 virus neutralization assays independently validated the successful induction of neutralizing activities in immunized mice. Based on Epitope25, we developed a pain‐free DMNA. Epitope25‐DMNA was able to elicit neutralizing activities against SARS‐CoV‐2 wild type strain as confirmed by live SARS‐CoV‐2 virus neutralization assay, achieving similar effects as subcutaneous injection of Epitope25 in mice. No side‐effects were observed and no obvious damages were found as shown in hematoxylin–eosin staining of tissues from liver and kidney. This study may lay the foundation for identifying highly antigenic linear B‐cell epitopes and developing promising and easy operating vaccines against SARS‐CoV‐2. The screening workflow we presented can be broadly applied to identify B‐cell epitopes for a large repertoire of virus, not limited to coronavirus.

## 4 Experimental Section

### Data Retrieval and Structural Analysis

SARS‐CoV‐2 protein sequence (Accession No. MN908947.3) was extracted from the NCBI database.^[^ ^64^ ^]^ Experimentally solved 3D structure of SARS‐CoV‐2 S protein (PDB ID: 6VSB)^[^ ^18^ ^]^ was retrieved from Protein Data Bank. The transmembrane topology of S protein was examined by TMHMM.v2.0 (http://www.cbs.dtu.dk/services/TMHMM/). VaxiJen v2.0^[^ ^56^ ^]^ was used to estimate the antigenicity of full‐length SARS‐CoV‐2 S protein.

### Linear B‐Cell Epitope Prediction for SARS‐CoV‐2 S Protein

A total of eight algorithms were used to predict linear B‐cell epitopes for SARS‐CoV‐2 S protein. Bepipred and Bepipred2.0 with default parameter settings, Kolaskar and Tongaonkar antigenicity, Parker hydrophilicity, Chou and Fasman beta‐turn, and Karplus and Schulz flexibility provided by IEDB (Immune‐Epitope‐Database and Analysis‐Resource)^[^ ^49^ ^]^ were applied upon SARS‐CoV‐2 S protein sequence to predict linear B‐cell epitopes. Linear B‐cell epitopes were also predicted by BcePred^[^ ^50^ ^]^ using accessibility, antigenic propensity, exposed surface, flexibility, hydrophilicity, polarity, and turns. The ANNpred‐based server ABCpred,^[^ ^51^ ^]^ Ellipro,^[^ ^52^ ^]^ BCPREDS,^[^ ^53^ ^]^ AAP,^[^ ^54^ ^]^ FBCPRED,^[^ ^53^ ^]^ and COVIDep^[^ ^55^ ^]^ were also employed to predict linear B‐cell epitopes. All the linear B‐cell epitopes with more than or equal to eight amino acids were combined to the linear B‐cell epitope candidate list. The antigenicity of the remained linear B‐cell epitopes was evaluated by VaxiJen 2.0.^[^ ^56^ ^]^ A stringent criterion was employed to have linear B‐cell epitopes with an antigenicity score larger than 0.9 viewed adequate to initiate a defensive immune reaction. According to the transmembrane topology of SARS‐CoV‐2 S protein predicted by TMHMM v2.0 (outside: 1–1213; transmembrane: 1214–1236; inside: 1237–1273), the unique linear B‐cell epitopes on the outer surface were retained for downstream analysis with intracellular epitopes eliminated. Epitopes classified by ToxinPred^[^ ^57^ ^]^ as nontoxin were remained.

### Identification of Hotspot Regions Enriched with Highly Antigenic Linear B‐Cell Epitopes

The distribution of unique linear B‐cell epitopes was drawn along the S protein. The regions with coverage of epitopes larger than 10 were determined as the peaks, which included a large number of B‐cell epitopes with varying length predicted by different algorithms. Discontinuous B‐cell epitope residues were predicted by Discotope 2.0 using A, B, and C chain of the 3D structure of S protein (PDB ID: 6VSB). Two regions (R1: AA 415–562 and R2: AA 1071–1146) were identified containing more discontinuous B‐cell epitopes. The relationship between the two regions and RBD were examined. The B‐cell epitope peaks overlapped with RBD domains were kept including P1 (AA 369–400), P2 (AA 401–452), P3 (AA 482–523), and P4 (524–561).

The evolutionary conservation status for each residue of SARS‐CoV‐2 was investigated by ConSurf^[^ ^65^ ^]^ using the amino acid sequences of S protein from seven known coronaviruses including SARS‐CoV‐2 (YP_009724390.1), SARS‐CoV (NP_828851.1), MERS‐CoV (YP_009047204.1), alpha coronavirus 229E (NP_073551.1), alpha coronavirus NL63 (AFV53148.1), beta coronavirus OC43 (YP_009555241.1), and beta coronavirus HKU1 (AAT98580.1). 28 205 mutations of 3816 sites in S protein were extracted from an open‐access database NGDC (https://ngdc.cncb.ac.cn/ncov), which documented 6 842 316 SARS‐CoV‐2 virus strains’ sequences. 7430 of 28 205 mutations led to amino acid changes, of which 1157 were in RBD and 112 were from the key amino acids critical for protein interactions based on NGDC (December 2, 2022). Peaks of P2 and P3 included most of key amino acids critical for protein interactions, and therefore P2 and P3 were excluded. The locations of the P1 and P4 on the 3D structure of SARS‐CoV‐2 S protein (PDB ID: 6VSB) were examined through open‐source icn3d (https://structure.ncbi.nlm.nih.gov/Structure/icn3d/). P1 hotspot regions were found located on the exposed area of spike head of S protein. P1 hotspot region was determined as the region would harbor highly antigenic linear B‐cell epitopes.

“YNSASFSTFKCYGVSPTKLNDLCFT” (named as Epitope25) was selected for further validation as it had 25 amino acids and was the longest one among the 46 B‐cell epitopes in P1 hotspot regions. Allergenicity of Epitope25 was assessed by Allergen FP 1.0^[^ ^66^ ^]^ (http://ddgpharmfac.net/AllergenFP/). As the stable peptides digested by fewer enzymes are more favorable candidate vaccines,^[^ ^67^ ^]^ the digestion of Epitope25 by 37 enzymes was examined through protein digest server (https://web.expasy.org/peptide_cutter/) including Arg‐C proteinase, Asp‐N endopeptidase, Asp‐N endopeptidase + N‐terminal Glu, BNPS‐Skatole, Caspase1, Caspase2, Caspase3, Caspase4, Caspase5, Caspase6, Caspase7, Caspase8, Caspase9, Caspase10, Chymotrypsin‐high specificity (C‐term to [FYW], not before P), Chymotrypsin‐low specificity (C‐term to [FYWML], not before P), Clostripain, CNBr, Enterokinase, GranzymeB, Factor Xa, Formic acid, Glutamyl endopeptidase, Hydroxylamine, Iodosobenzoic acid, LysC, LysN, NTCB (2‐nitro‐5‐thiocyanobenzoic acid), Pepsin (pH1.3), Pepsin (pH > 2), Proline‐endopeptidase, Proteinase K, Staphylococcal peptidase I, Tobacco etch virus protease, Thermolysin, Thrombin, and Trypsin.

### Peptide Synthesis

The Epitope25, “RRRRRRRRRRRRRRRR” (arbPep), and “VKPSFYVYSRVKNLN” randomly selected from envelope protein of SARS‐CoV‐2 (ctrlPep) were synthesized by Scilight‐Peptide Inc., Beijing, China via a practical approach of Fmoc solid‐phase peptide synthesis. The unsophisticated peptides were purified using a Varian ProStar 218 high‐performance liquid chromatography (HPLC) instrument with an Agilent Venusil MP C18 reversed phase column. Peptides were eluted with a linear gradient of water, H_2_O, and acetonitrile, CAN, (both having 0.05% TFA) at a flow rate of 1 mL min^−1^. The separation was monitored at 220 nm using UV detection. Then peptides were subjected to Voyager‐DE STR mass spectrometric (MS) analysis. The solvents for gradient elution HPLC are: solvent A, CAN 2%, TFA 0.05% and solvent B, CAN 90%, TFA 0.05%. Peptides were dissolved in deionized H_2_O at a final concentration of 20 mg mL^−1^ and stored at −20 °C until further use.

### Cell Lines and Viruses

Vero cells (ATCC, CCL‐81) and 293T cells (ATCC, CRL‐11268) were maintained in Dulbecco's modified Eagle's medium (Gibco, USA) supplemented with 10% fetal bovine serum (Hyclone, USA), penicillin (Hyclone, USA, 100 units mL^−1^), and streptomycin (Hyclone, USA, 100 µg mL^−1^) (complete medium) in 5% CO2 environment at 37 °C and passaged every 2–3 days. BetaCoV/Beijing/IMEBJ01/2020 strain (Genome Warehouse Accession No. GWHACAX01000000) was isolated from a COVID‐19 patient and propagated in Vero cells. Virus titers were determined on Vero cells, and virus stocks were stored in aliquots at −80 °C until use (100 CCID50/0.05 mL). SARS‐CoV‐2 pseudoviruses were provided by Dr. Weijin Huang at Institute for Biological Product Control, National Institutes for Food and Drug Control (NIFDC) of China.^[^ ^68^ ^]^ The experiments with infectious SARS‐CoV‐2 were performed at the biosafety level 3 facilities in Beijing Institute of Microbiology and Epidemiology, Academy of Military Sciences, China.

### Animal Experiments

All animal experiments were approved by and carried out in accordance with the guidelines of the Institutional Experimental Animal Welfare and Ethics Committee. A total of 50 specific‐pathogen‐free female BALB/c mice aged 6–8 weeks and two SD rats were obtained from Beijing Vital River Laboratory Animal Technologies Co., Ltd (Beijing, China) and were housed and bred in the temperature‐, humidity‐, and light cycle‐controlled animal facility (20 ± 2 °C; 50 ± 10%; light, 7:00–19:00; dark, 19:00–7:00). Porcine skins (from adult pig) were purchased from a local slaughterhouse immediately following death.

### Vaccination of Mice for Assessing the Neutralizing Activities Induced by Epitope25

The peptide dosage (5 µg) was determined based on results from previous studies. CoVac‐1 vaccine peptides were reported to induce T‐cell immunity on the dose of 250 µg in human.^[^ ^69^ ^]^ After conversion of human doses to mouse equivalent doses, 5 µg is a reasonable dose for mice. Additionally, the dose of 5 µg is commonly used and reported to be safe and able to induce humoral immune response in mice for RBD‐Dc‐based COVID‐19 vaccine,^[^ ^70^ ^]^ an adjuvanted subunit SARS‐CoV‐2 spike protein vaccine,^[^ ^71^ ^]^ and mRNA vaccine.^[^ ^72^ ^]^ Linear B‐cell epitopes (“YNSASFSTFKCYGVSPTKLNDLCFT”, named as Epitope25) and one arbitrary control peptide (“RRRRRRRRRRRRRRRR”, named as arbPep) were respectively used to immunize BALB/c mice (*n* = 5 mice per peptide; each peptide: 5 µg per mouse) with aluminum hydroxide as adjuvant (10 µg per mouse) through five consequent subcutaneous injections of 5 µg dose with 7 days interval between two consecutive injections. Another two groups of BALB/C mice (*n* = 5 mice per group) were immunized with Al(OH)3 adjuvant (5 µg per mouse) as a control for Epitope25 and arbPep, respectively. Sera from mice immunized with Epitope25, arbPep, or Al(OH)3 adjuvant were collected for Indirect ELISA. Sera from mice immunized with Epitope25 or arbPep were collected for SARS‐CoV‐2 pseudovirus neutralization assay titration (EC50), and SARS‐CoV‐2 live virus neutralization assay titration (NT_50_).

### SARS‐CoV‐2 S1‐Specific IgG Assay in Mice (Indirect ELISA)

96‐well polystyrene microplates (Oriental Ocean Global Health, China) were coated with 2 µg mL^−1^ (50 µL per well) SARS‐CoV‐2 spike protein (S1 Subunit, His tag) (Sino Biological, China, cat no: 40591‐V08H) in carbonate bicarbonate buffer pH 9.6 and the plates were incubated at 4 °C overnight. The plates were then blocked at 37 °C for 1 h with PBS (Solarbio, China, cat no: A8020) pH 7.4 in 5% skim milk (blocking buffer) and washed with PBST (0.1% Tween‐80) three times. Serial dilutions of sera in dilution buffer (Solarbio, China, cat no: P1010) were added to the plates and incubated at 37 °C for 30 min. HRP‐conjugated goat anti‐mouse IgG (Southern Biotech, cat no: 1030‐05, 1:5000 dilution) was added to the plates, and the plates were incubated in incubator (ZHCHENG, China, ZXDP‐B2050) at 37 °C for 30 min and washed with PBST three times. The assay was developed for 10 min at 37 °C with 50 µL of TMB substrate solution (Solarbio, China, cat no: PR1200), stopped by the addition of 50 µL of stop solution (Solarbio, China, cat no: C1058), and then the optical density (OD) was measured at 450 nm (TECAN, INFINITE F50). The endpoint titer was defined as the highest reciprocal serum dilution that yielded anabsorbance ≥2.1‐fold over negative control serum values (OD value is set as 0.05, if it is less than 0.05). The indirect ELISA was performed at the biosafety level 2 facilities in Beijing Institute of Microbiology and Epidemiology, Academy of Military Medical Sciences, China.

### Fabrication and Characteristics of Dissolvable Microneedle Arrays

DMNAs incorporating Epitope25, control peptide of “VKPSFYVYSRVKNLN” (ctrlPep) randomly selected from envelope protein of SARS‐CoV‐2, and aluminum hydroxide adjuvant were fabricated from 8–11× kDa PVP at room temperature (22 °C), respectively. The MNA production molds were prepared by casting polydimethylsiloxane (SYLGARD 184) elastomer onto the copper MNA master molds that include 100 quadrangular pyramid shaped microneedles in a 10 × 10 array. The height, width, and apex angle of microneedles were chosen to be 700 µm, 300 µm, and 30°, respectively. Epitope25‐DMNA, ctrlPep‐DMNA, and Adjuvant‐DMNA were produced through a vacuum decompression molding technique. First, 5 µL solution mixture was prepared for each DMNA, which was made of Epitope25 (2.5 µg µL^−1^), aluminum hydroxide adjuvant (5 µg µL^−1^), and 8–11× kDa PVP (0.5 mg µL^−1^) for Epitope25‐DMNA, ctrlPep (2.5 µg µL^−1^), aluminum hydroxide adjuvant (5 µg µL^−1^), and 8–11 kDa PVP (0.5 mg µL^−1^) for ctrlPep‐DMNA, and aluminum hydroxide adjuvant (5 µg µL^−1^), and 8–11× kDa PVP (0.5 mg µL^−1^) for Adjuvant‐DMNA, respectively. The 5 µL solution mixture was dispensed onto each MNA production mold and then vacuum decompression (−100 kPa for 15 min) was carried out to fill the microneedle cavities. Subsequently, the excess solution was removed and the residue solution was dried at room temperature. The backing layer was made of 32–38 × 10 kDa PVP. 300 µL 32–38 × 10 kDa PVP (0.5 mg µL^−1^) were added to the surface of the microneedle and demolded after solidification (at 4 °C for 48 h) to obtain the final dissolvable MNA vaccines. The theoretical loading of each Epitope25‐DMNA, ctrlPep‐DMNA, or Adjuvant‐DMNA was 5 µg Epitope25, 5 µg ctrlPep and 10 µg aluminum hydroxide adjuvant, respectively.

The DMNAs were photographed using stereo microscopes (Motic, China) and scanning electron microscope (Zeiss, Germany). To prove the skin penetration capability and the diffusion of DMNA in the epidermis, a piece of DMNA containing Alexa Fluor 488 was inserted into a porcine skin at an application of force of 2 N. The section of this piece of skin was observed via fluorescence microscope (Olympus, Japan). The distribution of green fluorescence intensities along the microscopic aqueous pores formed in the skin after microneedle penetration was calculated using R. To prove no skin damages by DMNA in vivo, Adjuvant‐DMNAs were inserted onto the bare skin of SD rats at an application force of 2 N. The skin was subsequently imaged via lab‐made fisheye camera system at time of 0, 1, and 5 min.

### Vaccination of Mice for Assessing the Neutralizing Activities Induced by Epitope25‐DMNA

30 six‐ to eight‐week‐old female BALB/c mice were immunized through five consequent application with 7 days interval between two consecutive applications via six methods (five mice per immunization method) including three DMNA (Epitope25‐DMNA, ctrlPep‐DMNA, and Adjuvant‐DMNA), intramuscular injection (Epitope25‐IM), and two subcutaneous injection (Epitope25‐Sub‐Q and ctrlPep‐Sub‐Q), respectively. PBMCs from mice immunized with Epitope25‐DMNA and Adjuvant‐DMNA were collected for RNA sequencing. Sera from all mice were then collected for SARS‐CoV‐2 live virus neutralization assay titration (NT_50_).

### RNA‐seq Data Analysis

RNA‐seq was performed upon PBMCs of 15 mice immunized with Epitope25‐DMNA, Adjuvant‐DMNA, and Epitope25‐Sub‐Q (five mice per immunization method) by Novogene Co., Ltd. Genomic RNA (Life Tech, Cat#:15596‐018) was extracted and the mRNA‐Seq Sample Prep Kit (Illumina) was used to construct RNA‐seq libraries according to the manufacturer's instructions. The 7G raw FASTQ data per sample were generated by the Illumina HiSeq 2000 platform. FASTQ data were aligned to the mouse reference genome (GRCm38) using the STAR^[^ ^73^ ^]^ at default settings and Htseq‐count^[^ ^74^ ^]^ was used to compute read counts for each gene. DEseq2^[^ ^75^ ^]^ was applied to identify differential expression genes with adjusted *P*‐value cutoff of 0.05 and fold change value cutoff of 1.5. Gene Ontology Enrichment analysis upon differentially expressed genes was performed using clusterProfiler package in R. Cytoscape (v.3.9.0)^[^ ^76^ ^]^ was used to generate GO enrichment network.

### SARS‐CoV‐2 Pseudovirus Neutralization Assay

A pseudotyped virus‐based neutralization assay against SARS‐CoV‐2 in biosafety level 2 facilities was performed as previously described.^[^ ^61^ ^]^ Briefly, serial dilutions of the samples to be tested were mixed with 325‐1300 TCID_50_/mL of pseudotyped virus. The target cells were incubated for 24 h, and then the neutralizing antibody content of the sample was obtained by calculating the amount of pseudotyped virus entering the target cells which were detected by the expression of luciferase. The half maximal effective concentration (EC_50_) was calculated for the tested samples using the Reed–Muench method in GraphPad Prism 8 (GraphPad software, Inc., San Diego, CA, USA). The assays were performed at the biosafety level 2 facilities in Beijing Institute of Microbiology and Epidemiology, Academy of Military Medical Sciences, China.

### SARS‐CoV‐2 Live Virus Neutralization Assay

Microneutralization (MN) assay was performed to assess the neutralizing activity of sera from the mice. 50 µL (100 CCID_50_/0.05 mL) of SARS‐CoV‐2 IME‐BJ01 strain was incubated with serial dilution of heat‐inactivated sera in 5% CO_2_ environment at 37 °C for 1 h. The complexes of antibody‐virus (100TCID50/50 µL) were added to preplated Vero cell monolayers in 96‐well plates and incubated for 72 h. The Reed–Muench method was applied to estimate the dilution of sera required for NT_50_. The initial dilution of sera (1:16) was set as the confidence limit of the assay. Seropositivity was defined as a titer ≥ 16.

### H&E Staining

All mice immunized with Epitope25‐DMNA, Epitope25‐Sub‐Q, or Adjuvant‐DMNA were sacrificed to evaluate the size and weight of kidney and liver. Tissues from kidney and liver were collected and stained with Harris’ hematoxylin solution for 6 h at a temperature of 60–70 °C and were then rinsed in purified water until the water became colorless. The tissues were differentiated two times with 10% acetic acid and 85% ethanol in water for 2 and 10 h, respectively, followed by being rinsed with purified water. Saturated lithium carbonate solution was used to soak the tissues for 12 h, which were then rinsed with purified water again. Finally, eosin Y ethanol solution was applied to perform staining for 48 h.

### Statistical Analysis

Statistical analyses were performed using R. The differences between two different groups of mice were calculated using Mann–Whitney *U* test. *P*‐value ≤ 0.05 was determined as statistical significance.

### Ethical Statement

All animal studies were performed in strict accordance with the guidelines set by the Chinese Regulations of Laboratory Animals and Laboratory Animal‐Requirements of Environment and Housing Facilities. All animal procedures were reviewed and approved by the Animal Experiment Committee of Laboratory Animal Center, Academy of Military Medical Sciences (AMMS), China (Assurance No.: IACUC‐DWZX‐2020‐040). This study and all the relevant experiments were approved by Ethics Committee of Beijing Institute of Microbiology and Epidemiology, China (IACUC‐2020‐030).

## Conflict of Interest

The authors declare no conflict of interest.

## Author Contributions

L.L., Z.Z, and X.Y. contributed equally to this work. J.Z., H.W., Y.F., Y.W., and Z.Z. conceived the project, supervised the work, and interpreted the data. L.L., W.L., and T.S. performed the immune‐informatics analysis. L.W. performed mapping of RNAseq raw data and calculated gene read counts. J.Z. performed the differential gene expression analysis and enrichment analysis. W.Y., Z.Z., and J.Z. immunized all the mice. Z.Z., X.Y., S.C., M.L., and T.W. performed indirect ELISA, pseudo and live SARS‐CoV‐2 neutralization titer assay. W.Y., Z.S., C.D., Z.L., and Z.Y. fabricated dissolvable microneedle array and H&E staining. J.Z., H.W., Y.F., Y.W., and Z.Z. drafted the manuscript, edited the draft, and prepared the final manuscript, which was approved by all co‐authors. All the authors have approved the manuscript for publication and declare no potential conflicts of interest. The authors also certify that the manuscript is their own research work with nothing taken from the published or unpublished work of others, and has not simultaneously been submitted elsewhere.

## Supporting information

Supporting Information

## Data Availability

The data that support the findings of this study are openly available. SARS‐CoV‐2 protein sequence (Accession No. MN908947.3)^[^ ^63^ ^]^ was extracted from the NCBI database. Experimentally solved 3D structure of SARS‐CoV‐2 S protein (PDB ID: 6VSB)^[^ ^18^ ^]^ was retrieved from Protein Data Bank. The mutations and the key amino acids critical for protein interactions were extracted from an open‐access database NGDC (https://ngdc.cncb.ac.cn/ncov) (December 2, 2022). Peaks of P2 and P3 included most of key amino acids critical for protein interactions, and therefore P2 and P3 were excluded. The software that supported the findings of this study is openly available in the original literatures which were cited properly in the Experimental Section. The RNAseq data were uploaded to Genome Sequence Archive of National Genomics Data Center (Accession No. CRA009144). All the authors have approved the manuscript for publication and declare no potential conflicts of interest. We also certify that the manuscript is our own research work with nothing taken from the published or unpublished work of others, and has not simultaneously been submitted elsewhere.
