# Supplementary material for: A Newly Identified Spike Protein Targeted Linear B‐Cell Epitope Based Dissolvable Microneedle Array Successfully Eliciting Neutralizing Activities against SARS‐CoV‐2 Wild‐Type Strain in Mice
Source: Adv Sci (Weinh). 2023 May 10;10(20):2207474. doi: 10.1002/advs.202207474 (PMC10369230; doi:10.1002/advs.202207474)
Supplement: Supplementary file 1 — Supporting Information [file ADVS-10-2207474-s001.pdf]

ADVANCED SCIENCE
Open Access

Supporting Information

for *Adv. Sci.*, DOI 10.1002/advs.202207474

A Newly Identified Spike Protein Targeted Linear B-Cell Epitope Based Dissolvable Microneedle Array Successfully Eliciting Neutralizing Activities against SARS-CoV-2 Wild-Type Strain in Mice

Lin Li, Zhongpeng Zhao, Xiaolan Yang, Zhongyi Su, Wendong Li, Shaolong Chen, Lu Wang, Ting Sun, Chen Du, Ziyi Li, Zeqian Yang, Min Li, Tiecheng Wang, Ying Wang, Yubo Fan*, Hui Wang* and Jing Zhang**

Supplementary materials

Supplementary Table 1. The full list of linear B-cell epitopes from spike protein of SARS-CoV-2.

Supplementary Table 2. Predicted B-cell epitopes with > 7 AA and antigenicity > 0.9.

Supplementary Table 3. Unique list of predicted B-cell epitopes.

Supplementary Table 4. Discontinuous B-cell epitopes predicted by DiscoTope 2 with the discotope score threshold of -3.7 (default setting).

Supplementary Table 5. Predicted B-cell epitopes in peak 1, 2, 3 and 4.

Supplementary Table 6. Differentially expressed genes in PBMCs of mice immunized with Epitope25-DMNA compared with those with Adjuvant-DMNA.

Supplementary Table 7. Enriched GO termed for up-regualted genes in PBMCs of mice immunized with Epitope25-DMNA compared with those having Adjuvant-DMNA.

Supplementary Table 8. Enriched GO termed for down-regualted genes in PBMCs of mice immunized with Epitope25-DMNA compared with those having Adjuvant-DMNA.

Supplementary Table 9. Differentially expressed genes in PBMCs of mice immunized with Epitope25-Sub-Q compared with those having Adjuvant-DMNA.

Supplementary Table 10. Enriched GO termed for up-regualted genes in PBMCs of mice immunized with Epitope25-DMNA compared with those having Adjuvant-DMNA.

Supplementary Table 11. Enriched GO termed for down-regualted genes in PBMCs of mice immunized with Epitope25-Sub-Q compared with those having Adjuvant-DMNA.

Supplementary Table 1. Predicted B-cell epitopes.

start	end	peptide	Peptide Length	methods
375	384	STFKCYGVSP	10	ABCPRED
754	763	LQYGSFCTQL	10	ABCPRED
594	603	GVSVITPGTN	10	ABCPRED
489	498	YFPLQSYGFQ	10	ABCPRED
14	23	QCVNLTTRTQ	10	ABCPRED
666	675	IGAGICASYQ	10	ABCPRED
562	571	FQQFGRDIAD	10	ABCPRED
742	751	ICGDSTECSN	10	ABCPRED
417	426	KIADYNYKLP	10	ABCPRED
376	385	TFKCYGVSPT	10	ABCPRED
194	203	FKNIDGYFKI	10	ABCPRED
487	496	NCYFPLQSYG	10	ABCPRED
42	51	VFRSSVLHST	10	ABCPRED
97	106	KSNIIRGWIF	10	ABCPRED
632	641	TWRVYSTGSN	10	ABCPRED
406	415	EVRQIAPGQT	10	ABCPRED
401	410	VIRGDEVRQI	10	ABCPRED
152	161	WMESEFRVYS	10	ABCPRED
657	666	NNSYECDIPI	10	ABCPRED
64	73	WFHAIHVSGT	10	ABCPRED
61	70	NVTWFHAIHV	10	ABCPRED
53	62	DLFLPFFSNV	10	ABCPRED
472	481	IYQAGSTPCN	10	ABCPRED
445	454	VGGNYNYLYR	10	ABCPRED
269	278	YLQPRTFLLK	10	ABCPRED
440	449	NLDSKVGGNY	10	ABCPRED
424	433	KLPDDFTGCV	10	ABCPRED
382	391	VSPTKLNDLC	10	ABCPRED
270	279	LQPRTFLLKY	10	ABCPRED
242	251	LALHRSYLTP	10	ABCPRED
89	98	GVYFASTEKS	10	ABCPRED
60	69	SNVTWFHAIH	10	ABCPRED
580	589	QTLEILDITP	10	ABCPRED
23	32	QLPPAYTNSF	10	ABCPRED
190	199	REFVFKNIDG	10	ABCPRED
184	193	GNFKNLREFV	10	ABCPRED
723	732	TTEILPVSMT	10	ABCPRED
682	691	RRARSVASQS	10	ABCPRED
279	288	YNENGTITDA	10	ABCPRED
122	131	NATNVVIKVC	10	ABCPRED
678	687	TNSPRRARSV	10	ABCPRED
665	674	PIGAGICASY	10	ABCPRED
490	499	FPLQSYGFQP	10	ABCPRED
423	432	YKLPDDFTGC	10	ABCPRED
82	91	PVLPFNDGVY	10	ABCPRED
736	745	VDCTMYICGD	10	ABCPRED
617	626	CTEVPVAIHA	10	ABCPRED
555	564	SNKKFLPFQQ	10	ABCPRED
529	538	KSTNLVKNKC	10	ABCPRED
450	459	NYLYRLFRKS	10	ABCPRED
368	377	LYNSASFSTF	10	ABCPRED
359	368	SNCVADYSVL	10	ABCPRED
310	319	KGIYQTSNFR	10	ABCPRED
307	316	TVEKGIYQTS	10	ABCPRED
291	300	CALDPLSETK	10	ABCPRED
237	246	RFQTLLALHR	10	ABCPRED
217	226	PQGFSALEPL	10	ABCPRED
162	171	SANNCTFEYV	10	ABCPRED
11	20	VSSQCVNLTT	10	ABCPRED
99	108	NIIRGWIFGT	10	ABCPRED
789	798	YKTPPIKDFG	10	ABCPRED
694	703	AYTMSLGAEN	10	ABCPRED
673	682	SYQTQTNSPR	10	ABCPRED
627	636	DQLTPTWRVY	10	ABCPRED
624	633	IHADQLTPTW	10	ABCPRED
550	559	GVLTESNKKF	10	ABCPRED
436	445	WNSNNLDSKV	10	ABCPRED
386	395	KLNDLCFTNV	10	ABCPRED
325	334	SIVRFPNITN	10	ABCPRED
293	302	LDPLSETKCT	10	ABCPRED
251	260	PGDSSSGWTA	10	ABCPRED
206	215	KHTPINLVRD	10	ABCPRED
141	150	LGVYYHKNNK	10	ABCPRED
787	796	QIYKTPPIKD	10	ABCPRED
777	786	NTQEVFAQVK	10	ABCPRED
756	765	YGSFCTQLNR	10	ABCPRED
640	649	SNVFQTRAGC	10	ABCPRED
432	441	CVIAWNSNNL	10	ABCPRED
355	364	RKRISNCVAD	10	ABCPRED
350	359	VYAWNRKRIS	10	ABCPRED
332	341	ITNLCPFGEV	10	ABCPRED
298	307	ETKCTLKSFT	10	ABCPRED
274	283	TFLLKYNENG	10	ABCPRED
263	272	AAYYVGYLQP	10	ABCPRED
138	147	DPFLGVYYHK	10	ABCPRED
775	784	DKNTQEVFAQ	10	ABCPRED
741	750	YICGDSTECS	10	ABCPRED
738	747	CTMYICGDST	10	ABCPRED
721	730	SVTTEILPVS	10	ABCPRED
636	645	YSTGSNVFQT	10	ABCPRED
623	632	AIHADQLTPT	10	ABCPRED
574	583	DAVRDPQTLE	10	ABCPRED
55	64	FLPFFSNVTW	10	ABCPRED
438	447	SNNLDSKVGG	10	ABCPRED
414	423	QTGKIADYNY	10	ABCPRED
357	366	RISNCVADYS	10	ABCPRED
331	340	NITNLCPFGE	10	ABCPRED
317	326	NFRVQPTESI	10	ABCPRED
313	322	YQTSNFRVQP	10	ABCPRED
289	298	VDCALDPLSE	10	ABCPRED
272	281	PRTFLLKYNE	10	ABCPRED
248	257	YLTPGDSSSG	10	ABCPRED
19	28	TTRTQLPPAY	10	ABCPRED
177	186	MDLEGKQGNF	10	ABCPRED
143	152	VYYHKNNKSW	10	ABCPRED
87	96	NDGVYFASTE	10	ABCPRED
720	729	ISVTTEILPV	10	ABCPRED
711	720	SIAIPTNFTI	10	ABCPRED
706	715	AYSNNSIAIP	10	ABCPRED
704	713	SVAYSNNSIA	10	ABCPRED
692	701	IIAYTMSLGA	10	ABCPRED
634	643	RVYSTGSNVF	10	ABCPRED
607	616	QVAVLYQDVN	10	ABCPRED
598	607	ITPGTNTSNQ	10	ABCPRED
542	551	NFNGLTGTGV	10	ABCPRED
495	504	YGFQPTNGVG	10	ABCPRED
439	448	NNLDSKVGGN	10	ABCPRED
429	438	FTGCVIAWNS	10	ABCPRED
40	49	DKVFRSSVLH	10	ABCPRED
338	347	FGEVFNATRF	10	ABCPRED
255	264	SSGWTAGAAA	10	ABCPRED
203	212	IYSKHTPINL	10	ABCPRED
20	29	TRTQLPPAYT	10	ABCPRED
17	26	NLTTRTQLPP	10	ABCPRED
165	174	NCTFEYVSQP	10	ABCPRED
161	170	SSANNCTFEY	10	ABCPRED
743	752	CGDSTECSNL	10	ABCPRED
536	545	NKCVNFNFNG	10	ABCPRED
483	492	VEGFNCYFPL	10	ABCPRED
441	450	LDSKVGGNYN	10	ABCPRED
352	361	AWNRKRISNC	10	ABCPRED
302	311	TLKSFTVEKG	10	ABCPRED
276	285	LLKYNENGTI	10	ABCPRED
221	230	SALEPLVDLP	10	ABCPRED
205	214	SKHTPINLVR	10	ABCPRED
195	204	KNIDGYFKIY	10	ABCPRED
174	183	PFLMDLEGKQ	10	ABCPRED
159	168	VYSSANNCTF	10	ABCPRED
150	159	KSWMESEFRV	10	ABCPRED
786	795	KQIYKTPPIK	10	ABCPRED
772	781	VEQDKNTQEV	10	ABCPRED
74	83	NGTKRFDNPV	10	ABCPRED
729	738	VSMTKTSVDC	10	ABCPRED
687	696	VASQSIIAYT	10	ABCPRED
619	628	EVPVAIHADQ	10	ABCPRED
565	574	FGRDIADTTD	10	ABCPRED
541	550	FNFNGLTGTG	10	ABCPRED
46	55	SVLHSTQDLF	10	ABCPRED
41	50	KVFRSSVLHS	10	ABCPRED
399	408	SFVIRGDEVR	10	ABCPRED
283	292	GTITDAVDCA	10	ABCPRED
116	125	SLLIVNNATN	10	ABCPRED
94	103	STEKSNIIRG	10	ABCPRED
773	782	EQDKNTQEVF	10	ABCPRED
770	779	IAVEQDKNTQ	10	ABCPRED
618	627	TEVPVAIHAD	10	ABCPRED
596	605	SVITPGTNTS	10	ABCPRED
595	604	VSVITPGTNT	10	ABCPRED
560	569	LPFQQFGRDI	10	ABCPRED
379	388	CYGVSPTKLN	10	ABCPRED
296	305	LSETKCTLKS	10	ABCPRED
218	227	QGFSALEPLV	10	ABCPRED
198	207	DGYFKIYSKH	10	ABCPRED
188	197	NLREFVFKNI	10	ABCPRED
785	794	VKQIYKTPPI	10	ABCPRED
757	766	GSFCTQLNRA	10	ABCPRED
75	84	GTKRFDNPVL	10	ABCPRED
710	719	NSIAIPTNFT	10	ABCPRED
62	71	VTWFHAIHVS	10	ABCPRED
597	606	VITPGTNTSN	10	ABCPRED
528	537	KKSTNLVKNK	10	ABCPRED
481	490	NGVEGFNCYF	10	ABCPRED
469	478	STEIYQAGST	10	ABCPRED
463	472	PFERDISTEI	10	ABCPRED
455	464	LFRKSNLKPF	10	ABCPRED
443	452	SKVGGNYNYL	10	ABCPRED
427	436	DDFTGCVIAW	10	ABCPRED
393	402	TNVYADSFVI	10	ABCPRED
172	181	SQPFLMDLEG	10	ABCPRED
127	136	VIKVCEFQFC	10	ABCPRED
681	690	PRRARSVASQ	10	ABCPRED
604	613	TSNQVAVLYQ	10	ABCPRED
584	593	ILDITPCSFG	10	ABCPRED
58	67	FFSNVTWFHA	10	ABCPRED
577	586	RDPQTLEILD	10	ABCPRED
535	544	KNKCVNFNFN	10	ABCPRED
520	529	APATVCGPKK	10	ABCPRED
502	511	GVGYQPYRVV	10	ABCPRED
492	501	LQSYGFQPTN	10	ABCPRED
478	487	TPCNGVEGFN	10	ABCPRED
433	442	VIAWNSNNLD	10	ABCPRED
411	420	APGQTGKIAD	10	ABCPRED
315	324	TSNFRVQPTE	10	ABCPRED
312	321	IYQTSNFRVQ	10	ABCPRED
208	217	TPINLVRDLP	10	ABCPRED
196	205	NIDGYFKIYS	10	ABCPRED
178	187	DLEGKQGNFK	10	ABCPRED
164	173	NNCTFEYVSQ	10	ABCPRED
128	137	IKVCEFQFCN	10	ABCPRED
783	792	AQVKQIYKTP	10	ABCPRED
779	788	QEVFAQVKQI	10	ABCPRED
761	770	TQLNRALTGI	10	ABCPRED
654	663	EHVNNSYECD	10	ABCPRED
638	647	TGSNVFQTRA	10	ABCPRED
573	582	TDAVRDPQTL	10	ABCPRED
567	576	RDIADTTDAV	10	ABCPRED
508	517	YRVVVLSFEL	10	ABCPRED
505	514	YQPYRVVVLS	10	ABCPRED
49	58	HSTQDLFLPF	10	ABCPRED
47	56	VLHSTQDLFL	10	ABCPRED
398	407	DSFVIRGDEV	10	ABCPRED
394	403	NVYADSFVIR	10	ABCPRED
387	396	LNDLCFTNVY	10	ABCPRED
295	304	PLSETKCTLK	10	ABCPRED
207	216	HTPINLVRDL	10	ABCPRED
204	213	YSKHTPINLV	10	ABCPRED
9	18	PLVSSQCVNL	10	ABCPRED
771	780	AVEQDKNTQE	10	ABCPRED
737	746	DCTMYICGDS	10	ABCPRED
732	741	TKTSVDCTMY	10	ABCPRED
727	736	LPVSMTKTSV	10	ABCPRED
722	731	VTTEILPVSM	10	ABCPRED
72	81	GTNGTKRFDN	10	ABCPRED
695	704	YTMSLGAENS	10	ABCPRED
501	510	NGVGYQPYRV	10	ABCPRED
50	59	STQDLFLPFF	10	ABCPRED
392	401	FTNVYADSFV	10	ABCPRED
337	346	PFGEVFNATR	10	ABCPRED
271	280	QPRTFLLKYN	10	ABCPRED
256	265	SGWTAGAAAY	10	ABCPRED
254	263	SSSGWTAGAA	10	ABCPRED
220	229	FSALEPLVDL	10	ABCPRED
216	225	LPQGFSALEP	10	ABCPRED
145	154	YHKNNKSWME	10	ABCPRED
114	123	TQSLLIVNNA	10	ABCPRED
112	121	SKTQSLLIVN	10	ABCPRED
691	700	SIIAYTMSLG	10	ABCPRED
614	623	DVNCTEVPVA	10	ABCPRED
563	572	QQFGRDIADT	10	ABCPRED
530	539	STNLVKNKCV	10	ABCPRED
494	503	SYGFQPTNGV	10	ABCPRED
409	418	QIAPGQTGKI	10	ABCPRED
402	411	IRGDEVRQIA	10	ABCPRED
322	331	PTESIVRFPN	10	ABCPRED
105	114	IFGTTLDSKT	10	ABCPRED
95	104	TEKSNIIRGW	10	ABCPRED
790	799	KTPPIKDFGG	10	ABCPRED
658	667	NSYECDIPIG	10	ABCPRED
649	658	CLIGAEHVNN	10	ABCPRED
635	644	VYSTGSNVFQ	10	ABCPRED
575	584	AVRDPQTLEI	10	ABCPRED
559	568	FLPFQQFGRD	10	ABCPRED
524	533	VCGPKKSTNL	10	ABCPRED
486	495	FNCYFPLQSY	10	ABCPRED
407	416	VRQIAPGQTG	10	ABCPRED
391	400	CFTNVYADSF	10	ABCPRED
384	393	PTKLNDLCFT	10	ABCPRED
347	356	FASVYAWNRK	10	ABCPRED
31	40	SFTRGVYYPD	10	ABCPRED
250	259	TPGDSSSGWT	10	ABCPRED
239	248	QTLLALHRSY	10	ABCPRED
224	233	EPLVDLPIGI	10	ABCPRED
21	30	RTQLPPAYTN	10	ABCPRED
131	140	CEFQFCNDPF	10	ABCPRED
117	126	LLIVNNATNV	10	ABCPRED
763	772	LNRALTGIAV	10	ABCPRED
707	716	YSNNSIAIPT	10	ABCPRED
633	642	WRVYSTGSNV	10	ABCPRED
609	618	AVLYQDVNCT	10	ABCPRED
578	587	DPQTLEILDI	10	ABCPRED
454	463	RLFRKSNLKP	10	ABCPRED
362	371	VADYSVLYNS	10	ABCPRED
330	339	PNITNLCPFG	10	ABCPRED
327	336	VRFPNITNLC	10	ABCPRED
314	323	QTSNFRVQPT	10	ABCPRED
280	289	NENGTITDAV	10	ABCPRED
258	267	WTAGAAAYYV	10	ABCPRED
158	167	RVYSSANNCT	10	ABCPRED
10	19	LVSSQCVNLT	10	ABCPRED
768	777	TGIAVEQDKN	10	ABCPRED
751	760	NLLLQYGSFC	10	ABCPRED
739	748	TMYICGDSTE	10	ABCPRED
733	742	KTSVDCTMYI	10	ABCPRED
699	708	LGAENSVAYS	10	ABCPRED
670	679	ICASYQTQTN	10	ABCPRED
669	678	GICASYQTQT	10	ABCPRED
650	659	LIGAEHVNNS	10	ABCPRED
647	656	AGCLIGAEHV	10	ABCPRED
630	639	TPTWRVYSTG	10	ABCPRED
611	620	LYQDVNCTEV	10	ABCPRED

601	610	GTNTSNQVAV	10	ABCPRED
593	602	GGVSVITPGT	10	ABCPRED
572	581	TTDAVRDPQT	10	ABCPRED
519	528	HAPATVCGPK	10	ABCPRED
493	502	QSYGFQPTNG	10	ABCPRED
480	489	CNGVEGFNCY	10	ABCPRED
459	468	SNLKPFERDI	10	ABCPRED
420	429	DYNYKLPDDF	10	ABCPRED
416	425	GKIADYNYKL	10	ABCPRED
383	392	SPTKLNDLCF	10	ABCPRED
381	390	GVSPTKLNDL	10	ABCPRED
35	44	GVYYPDKVFR	10	ABCPRED
328	337	RFPNITNLCP	10	ABCPRED
290	299	DCALDPLSET	10	ABCPRED
29	38	TNSFTRGVYY	10	ABCPRED
103	112	GWIFGTTLDS	10	ABCPRED
731	740	MTKTSVDCTM	10	ABCPRED
730	739	SMTKTSVDCT	10	ABCPRED
676	685	TQTNSPRRAR	10	ABCPRED
639	648	GSNVFQTRAG	10	ABCPRED
631	640	PTWRVYSTGS	10	ABCPRED
629	638	LTPTWRVYST	10	ABCPRED
621	630	PVAIHADQLT	10	ABCPRED
568	577	DIADTTDAVR	10	ABCPRED
556	565	NKKFLPFQQF	10	ABCPRED
544	553	NGLTGTGVLT	10	ABCPRED
523	532	TVCGPKKSTN	10	ABCPRED
457	466	RKSNLKPFER	10	ABCPRED
373	382	SFSTFKCYGV	10	ABCPRED
247	256	SYLTPGDSSS	10	ABCPRED
222	231	ALEPLVDLPI	10	ABCPRED
214	223	RDLPQGFSAL	10	ABCPRED
212	221	LVRDLPQGFS	10	ABCPRED
200	209	YFKIYSKHTP	10	ABCPRED
93	102	ASTEKSNIIR	10	ABCPRED
85	94	PFNDGVYFAS	10	ABCPRED
71	80	SGTNGTKRFD	10	ABCPRED
622	631	VAIHADQLTP	10	ABCPRED
615	624	VNCTEVPVAI	10	ABCPRED
477	486	STPCNGVEGF	10	ABCPRED
426	435	PDDFTGCVIA	10	ABCPRED
349	358	SVYAWNRKRI	10	ABCPRED
344	353	ATRFASVYAW	10	ABCPRED
34	43	RGVYYPDKVF	10	ABCPRED
309	318	EKGIYQTSNF	10	ABCPRED
281	290	ENGTITDAVD	10	ABCPRED
264	273	AYYVGYLQPR	10	ABCPRED
231	240	IGINITRFQT	10	ABCPRED
18	27	LTTRTQLPPA	10	ABCPRED
163	172	ANNCTFEYVS	10	ABCPRED
153	162	MESEFRVYSS	10	ABCPRED
142	151	GVYYHKNNKS	10	ABCPRED
129	138	KVCEFQFCND	10	ABCPRED
120	129	VNNATNVVIK	10	ABCPRED
111	120	DSKTQSLLIV	10	ABCPRED
755	764	QYGSFCTQLN	10	ABCPRED
701	710	AENSVAYSNN	10	ABCPRED
599	608	TPGTNTSNQV	10	ABCPRED
590	599	CSFGGVSVIT	10	ABCPRED
583	592	EILDITPCSF	10	ABCPRED
515	524	FELLHAPATV	10	ABCPRED
452	461	LYRLFRKSNL	10	ABCPRED
44	53	RSSVLHSTQD	10	ABCPRED
403	412	RGDEVRQIAP	10	ABCPRED
390	399	LCFTNVYADS	10	ABCPRED
354	363	NRKRISNCVA	10	ABCPRED
335	344	LCPFGEVFNA	10	ABCPRED
299	308	TKCTLKSFTV	10	ABCPRED
297	306	SETKCTLKSF	10	ABCPRED
268	277	GYLQPRTFLL	10	ABCPRED
262	271	AAAYYVGYLQ	10	ABCPRED
25	34	PPAYTNSFTR	10	ABCPRED
160	169	YSSANNCTFE	10	ABCPRED

104	115	WIFGTTLDSKTQ	12	ABCPRED
813	824	SKRSFIEDLLFN	12	ABCPRED
790	801	KTPPIKDFGGFN	12	ABCPRED
23	34	QLPPAYTNSFTR	12	ABCPRED
450	461	NYLYRLFRKSNL	12	ABCPRED
106	117	FGTTLDSKTQSL	12	ABCPRED
908	919	GIGVTQNVLYEN	12	ABCPRED
183	194	QGNFKNLREFVF	12	ABCPRED
116	127	SLLIVNNATNVV	12	ABCPRED
397	408	ADSFVIRGDEVR	12	ABCPRED
222	233	ALEPLVDLPIGI	12	ABCPRED
8	19	LPLVSSQCVNLT	12	ABCPRED
391	402	CFTNVYADSFVI	12	ABCPRED
139	150	PFLGVYYHKNNK	12	ABCPRED
118	129	LIVNNATNVVIK	12	ABCPRED
946	957	GKLQDVVNQNAQ	12	ABCPRED
885	896	GWTFGAGAALQI	12	ABCPRED
827	838	TLADAGFIKQYG	12	ABCPRED
404	415	GDEVRQIAPGQT	12	ABCPRED
369	380	YNSASFSTFKCY	12	ABCPRED
947	958	KLQDVVNQNAQA	12	ABCPRED
197	208	IDGYFKIYSKHT	12	ABCPRED
740	751	MYICGDSTECSN	12	ABCPRED
71	82	SGTNGTKRFDNP	12	ABCPRED
621	632	PVAIHADQLTPT	12	ABCPRED
421	432	YNYKLPDDFTGC	12	ABCPRED
374	385	FSTFKCYGVSPT	12	ABCPRED
161	172	SSANNCTFEYVS	12	ABCPRED
851	862	CAQKFNGLTVLP	12	ABCPRED
687	698	VASQSIIAYTMS	12	ABCPRED
611	622	LYQDVNCTEVPV	12	ABCPRED
580	591	QTLEILDITPCS	12	ABCPRED
508	519	YRVVVLSFELLH	12	ABCPRED
459	470	SNLKPFERDIST	12	ABCPRED
379	390	CYGVSPTKLNDL	12	ABCPRED
348	359	ASVYAWNRKRIS	12	ABCPRED
98	109	SNIIRGWIFGTT	12	ABCPRED
716	727	TNFTISVTTEIL	12	ABCPRED
588	599	TPCSFGGVSVIT	12	ABCPRED
419	430	ADYNYKLPDDFT	12	ABCPRED
361	372	CVADYSVLYNSA	12	ABCPRED
234	245	NITRFQTLLALH	12	ABCPRED
818	829	IEDLLFNKVTLA	12	ABCPRED
797	808	FGGFNFSQILPD	12	ABCPRED
698	709	SLGAENSVAYSN	12	ABCPRED
64	75	WFHAIHVSGTNG	12	ABCPRED
595	606	VSVITPGTNTSN	12	ABCPRED
395	406	VYADSFVIRGDE	12	ABCPRED
390	401	LCFTNVYADSFV	12	ABCPRED
365	376	YSVLYNSASFST	12	ABCPRED
340	351	EVFNATRFASVY	12	ABCPRED
238	249	FQTLLALHRSYL	12	ABCPRED
236	247	TRFQTLLALHRS	12	ABCPRED
125	136	NVVIKVCEFQFC	12	ABCPRED
114	125	TQSLLIVNNATN	12	ABCPRED
712	723	IAIPTNFTISVT	12	ABCPRED
606	617	NQVAVLYQDVNC	12	ABCPRED
473	484	YQAGSTPCNGVE	12	ABCPRED
362	373	VADYSVLYNSAS	12	ABCPRED
316	327	SNFRVQPTESIV	12	ABCPRED
235	246	ITRFQTLLALHR	12	ABCPRED
803	814	SQILPDPSKPSK	12	ABCPRED
784	795	QVKQIYKTPPIK	12	ABCPRED
773	784	EQDKNTQEVFAQ	12	ABCPRED
724	735	TEILPVSMTKTS	12	ABCPRED
692	703	IIAYTMSLGAEN	12	ABCPRED
662	673	CDIPIGAGICAS	12	ABCPRED
442	453	DSKVGGNYNYLY	12	ABCPRED
167	178	TFEYVSQPFLMD	12	ABCPRED
140	151	FLGVYYHKNNKS	12	ABCPRED
96	107	EKSNIIRGWIFG	12	ABCPRED
902	913	MAYRFNGIGVTQ	12	ABCPRED
871	882	AQYTSALLAGTI	12	ABCPRED

862	873	PPLLTDEMIAQY	12	ABCPRED
73	84	TNGTKRFDNPVL	12	ABCPRED
685	696	RSVASQSIIAYT	12	ABCPRED
560	571	LPFQQFGRDIAD	12	ABCPRED
53	64	DLFLPFFSNVTW	12	ABCPRED
402	413	IRGDEVRQIAPG	12	ABCPRED
401	412	VIRGDEVRQIAP	12	ABCPRED
305	316	SFTVEKGIYQTS	12	ABCPRED
299	310	TKCTLKSFTVEK	12	ABCPRED
293	304	LDPLSETKCTLK	12	ABCPRED
248	259	YLTPGDSSSGWT	12	ABCPRED
201	212	FKIYSKHTPINL	12	ABCPRED
103	114	GWIFGTTLDSKT	12	ABCPRED
901	912	QMAYRFNGIGVT	12	ABCPRED
869	880	MIAQYTSALLAG	12	ABCPRED
824	835	NKVTLADAGFIK	12	ABCPRED
788	799	IYKTPPIKDFGG	12	ABCPRED
6	17	VLLPLVSSQCVN	12	ABCPRED
486	497	FNCYFPLQSYGF	12	ABCPRED
443	454	SKVGGNYNYLYR	12	ABCPRED
42	53	VFRSSVLHSTQD	12	ABCPRED
385	396	TKLNDLCFTNVY	12	ABCPRED
358	369	ISNCVADYSVLY	12	ABCPRED
341	352	VFNATRFASVYA	12	ABCPRED
331	342	NITNLCPFGEVF	12	ABCPRED
112	123	SKTQSLLIVNNA	12	ABCPRED
856	867	NGLTVLPPLLTD	12	ABCPRED
783	794	AQVKQIYKTPPI	12	ABCPRED
719	730	TISVTTEILPVS	12	ABCPRED
579	590	PQTLEILDITPC	12	ABCPRED
546	557	LTGTGVLTESNK	12	ABCPRED
501	512	NGVGYQPYRVVV	12	ABCPRED
353	364	WNRKRISNCVAD	12	ABCPRED
333	344	TNLCPFGEVFNA	12	ABCPRED
162	173	SANNCTFEYVSQ	12	ABCPRED
143	154	VYYHKNNKSWME	12	ABCPRED
133	144	FQFCNDPFLGVY	12	ABCPRED
940	951	STASALGKLQDV	12	ABCPRED
932	943	GKIQDSLSSTAS	12	ABCPRED
929	940	SAIGKIQDSLSS	12	ABCPRED
860	871	VLPPLLTDEMIA	12	ABCPRED
812	823	PSKRSFIEDLLF	12	ABCPRED
736	747	VDCTMYICGDST	12	ABCPRED
584	595	ILDITPCSFGGV	12	ABCPRED
500	511	TNGVGYQPYRVV	12	ABCPRED
479	490	PCNGVEGFNCYF	12	ABCPRED
417	428	KIADYNYKLPDD	12	ABCPRED
373	384	SFSTFKCYGVSP	12	ABCPRED
354	365	NRKRISNCVADY	12	ABCPRED
287	298	DAVDCALDPLSE	12	ABCPRED
244	255	LHRSYLTPGDSS	12	ABCPRED
134	145	QFCNDPFLGVYY	12	ABCPRED
900	911	MQMAYRFNGIGV	12	ABCPRED
678	689	TNSPRRARSVAS	12	ABCPRED
605	616	SNQVAVLYQDVN	12	ABCPRED
599	610	TPGTNTSNQVAV	12	ABCPRED
569	580	IADTTDAVRDPQ	12	ABCPRED
54	65	LFLPFFSNVTWF	12	ABCPRED
493	504	QSYGFQPTNGVG	12	ABCPRED
469	480	STEIYQAGSTPC	12	ABCPRED
233	244	INITRFQTLLAL	12	ABCPRED
207	218	HTPINLVRDLPQ	12	ABCPRED
132	143	EFQFCNDPFLGV	12	ABCPRED
101	112	IRGWIFGTTLDS	12	ABCPRED
941	952	TASALGKLQDVV	12	ABCPRED
933	944	KIQDSLSSTASA	12	ABCPRED
867	878	DEMIAQYTSALL	12	ABCPRED
771	782	AVEQDKNTQEVF	12	ABCPRED
594	605	GVSVITPGTNTS	12	ABCPRED
593	604	GGVSVITPGTNT	12	ABCPRED
585	596	LDITPCSFGGVS	12	ABCPRED
550	561	GVLTESNKKFLP	12	ABCPRED
371	382	SASFSTFKCYGV	12	ABCPRED

339	350	GEVFNATRFASV	12	ABCPRED
319	330	RVQPTESIVRFP	12	ABCPRED
26	37	PAYTNSFTRGVY	12	ABCPRED
229	240	LPIGINITRFQT	12	ABCPRED
205	216	SKHTPINLVRDL	12	ABCPRED
194	205	FKNIDGYFKIYS	12	ABCPRED
160	171	YSSANNCTFEYV	12	ABCPRED
150	161	KSWMESEFRVYS	12	ABCPRED
149	160	NKSWMESEFRVY	12	ABCPRED
147	158	KNNKSWMESEFR	12	ABCPRED
903	914	AYRFNGIGVTQN	12	ABCPRED
802	813	FSQILPDPSKPS	12	ABCPRED
668	679	AGICASYQTQTN	12	ABCPRED
63	74	TWFHAIHVSGTN	12	ABCPRED
609	620	AVLYQDVNCTEV	12	ABCPRED
505	516	YQPYRVVVLSFE	12	ABCPRED
382	393	VSPTKLNDLCFT	12	ABCPRED
363	374	ADYSVLYNSASF	12	ABCPRED
318	329	FRVQPTESIVRF	12	ABCPRED
250	261	TPGDSSSGWTAG	12	ABCPRED
228	239	DLPIGINITRFQ	12	ABCPRED
223	234	LEPLVDLPIGIN	12	ABCPRED
174	185	PFLMDLEGKQGN	12	ABCPRED
169	180	EYVSQPFLMDLE	12	ABCPRED
159	170	VYSSANNCTFEY	12	ABCPRED
110	121	LDSKTQSLLIVN	12	ABCPRED
886	897	WTFGAGAALQIP	12	ABCPRED
798	809	GGFNFSQILPDP	12	ABCPRED
778	789	TQEVFAQVKQIY	12	ABCPRED
76	87	TKRFDNPVLPFN	12	ABCPRED
734	745	TSVDCTMYICGD	12	ABCPRED
704	715	SVAYSNNSIAIP	12	ABCPRED
686	697	SVASQSIIAYTM	12	ABCPRED
61	72	NVTWFHAIHVSG	12	ABCPRED
590	601	CSFGGVSVITPG	12	ABCPRED
553	564	TESNKKFLPFQQ	12	ABCPRED
525	536	CGPKKSTNLVKN	12	ABCPRED
368	379	LYNSASFSTFKC	12	ABCPRED
2	13	FVFLVLLPLVSS	12	ABCPRED
16	27	VNLTTRTQLPPA	12	ABCPRED
151	162	SWMESEFRVYSS	12	ABCPRED
135	146	FCNDPFLGVYYH	12	ABCPRED
128	139	IKVCEFQFCNDP	12	ABCPRED
945	956	LGKLQDVVNQNA	12	ABCPRED
934	945	IQDSLSSTASAL	12	ABCPRED
884	895	SGWTFGAGAALQ	12	ABCPRED
861	872	LPPLLTDEMIAQ	12	ABCPRED
67	78	AIHVSGTNGTKR	12	ABCPRED
646	657	RAGCLIGAEHVN	12	ABCPRED
597	608	VITPGTNTSNQV	12	ABCPRED
394	405	NVYADSFVIRGD	12	ABCPRED
311	322	GIYQTSNFRVQP	12	ABCPRED
28	39	YTNSFTRGVYYP	12	ABCPRED
264	275	AYYVGYLQPRTF	12	ABCPRED
253	264	DSSSGWTAGAAA	12	ABCPRED
942	953	ASALGKLQDVVN	12	ABCPRED
852	863	AQKFNGLTVLPP	12	ABCPRED
84	95	LPFNDGVYFAST	12	ABCPRED
766	777	ALTGIAVEQDKN	12	ABCPRED
70	81	VSGTNGTKRFDN	12	ABCPRED
682	693	RRARSVASQSII	12	ABCPRED
631	642	PTWRVYSTGSNV	12	ABCPRED
463	474	PFERDISTEIYQ	12	ABCPRED
43	54	FRSSVLHSTQDL	12	ABCPRED
420	431	DYNYKLPDDFTG	12	ABCPRED
412	423	PGQTGKIADYNY	12	ABCPRED
857	868	GLTVLPPLLTDE	12	ABCPRED
849	860	LICAQKFNGLTV	12	ABCPRED
819	830	EDLLFNKVTLAD	12	ABCPRED
774	785	QDKNTQEVFAQV	12	ABCPRED
721	732	SVTTEILPVSMT	12	ABCPRED
632	643	TWRVYSTGSNVF	12	ABCPRED
582	593	LEILDITPCSFG	12	ABCPRED

475	486	AGSTPCNGVEGF	12	ABCPRED
448	459	NYNYLYRLFRKS	12	ABCPRED
410	421	IAPGQTGKIADY	12	ABCPRED
389	400	DLCFTNVYADSF	12	ABCPRED
335	346	LCPFGEVFNATR	12	ABCPRED
218	229	QGFSALEPLVDL	12	ABCPRED
198	209	DGYFKIYSKHTP	12	ABCPRED
94	105	STEKSNIIRGWI	12	ABCPRED
828	839	LADAGFIKQYGD	12	ABCPRED
822	833	LFNKVTLADAGF	12	ABCPRED
791	802	TPPIKDFGGFNF	12	ABCPRED
666	677	IGAGICASYQTQ	12	ABCPRED
648	659	GCLIGAEHVNNS	12	ABCPRED
628	639	QLTPTWRVYSTG	12	ABCPRED
601	612	GTNTSNQVAVLY	12	ABCPRED
561	572	PFQQFGRDIADT	12	ABCPRED
56	67	LPFFSNVTWFHA	12	ABCPRED
540	551	NFNFNGLTGTGV	12	ABCPRED
535	546	KNKCVNFNFNGL	12	ABCPRED
532	543	NLVKNKCVNFNF	12	ABCPRED
530	541	STNLVKNKCVNF	12	ABCPRED
523	534	TVCGPKKSTNLV	12	ABCPRED
496	507	GFQPTNGVGYQP	12	ABCPRED
48	59	LHSTQDLFLPFF	12	ABCPRED
415	426	TGKIADYNYKLP	12	ABCPRED
378	389	KCYGVSPTKLND	12	ABCPRED
367	378	VLYNSASFSTFK	12	ABCPRED
36	47	VYYPDKVFRSSV	12	ABCPRED
343	354	NATRFASVYAWN	12	ABCPRED
260	271	AGAAAYYVGYLQ	12	ABCPRED
258	269	WTAGAAAYYVGY	12	ABCPRED
204	215	YSKHTPINLVRD	12	ABCPRED
20	31	TRTQLPPAYTNS	12	ABCPRED
184	195	GNFKNLREFVFK	12	ABCPRED
12	23	SSQCVNLTTRTQ	12	ABCPRED
937	948	SLSSTASALGKL	12	ABCPRED
838	849	GDCLGDIAARDL	12	ABCPRED
830	841	DAGFIKQYGDCL	12	ABCPRED
809	820	PSKPSKRSFIED	12	ABCPRED
768	779	TGIAVEQDKNTQ	12	ABCPRED
730	741	SMTKTSVDCTMY	12	ABCPRED
592	603	FGGVSVITPGTN	12	ABCPRED
562	573	FQQFGRDIADTT	12	ABCPRED
556	567	NKKFLPFQQFGR	12	ABCPRED
414	425	QTGKIADYNYKL	12	ABCPRED
413	424	GQTGKIADYNYK	12	ABCPRED
400	411	FVIRGDEVRQIA	12	ABCPRED
285	296	ITDAVDCALDPL	12	ABCPRED
262	273	AAAYYVGYLQPR	12	ABCPRED
239	250	QTLLALHRSYLT	12	ABCPRED
225	236	PLVDLPIGINIT	12	ABCPRED
206	217	KHTPINLVRDLP	12	ABCPRED
144	155	YYHKNNKSWMES	12	ABCPRED
14	25	QCVNLTTRTQLP	12	ABCPRED
936	947	DSLSSTASALGK	12	ABCPRED
866	877	TDEMIAQYTSAL	12	ABCPRED
816	827	SFIEDLLFNKVT	12	ABCPRED
800	811	FNFSQILPDPSK	12	ABCPRED
776	787	KNTQEVFAQVKQ	12	ABCPRED
775	786	DKNTQEVFAQVK	12	ABCPRED
769	780	GIAVEQDKNTQE	12	ABCPRED
757	768	GSFCTQLNRALT	12	ABCPRED
710	721	NSIAIPTNFTIS	12	ABCPRED
673	684	SYQTQTNSPRRA	12	ABCPRED
619	630	EVPVAIHADQLT	12	ABCPRED
58	69	FFSNVTWFHAIH	12	ABCPRED
504	515	GYQPYRVVVLSF	12	ABCPRED
478	489	TPCNGVEGFNCY	12	ABCPRED
403	414	RGDEVRQIAPGQ	12	ABCPRED
380	391	YGVSPTKLNDLC	12	ABCPRED
321	332	QPTESIVRFPNI	12	ABCPRED
246	257	RSYLTPGDSSSG	12	ABCPRED
210	221	INLVRDLPQGFS	12	ABCPRED

190	201	REFVFKNIDGYF	12	ABCPRED
157	168	FRVYSSANNCTF	12	ABCPRED
95	106	TEKSNIIRGWIF	12	ABCPRED
906	917	FNGIGVTQNVLY	12	ABCPRED
882	893	ITSGWTFGAGAA	12	ABCPRED
881	892	TITSGWTFGAGA	12	ABCPRED
844	855	IAARDLICAQKF	12	ABCPRED
744	755	GDSTECSNLLLQ	12	ABCPRED
680	691	SPRRARSVASQS	12	ABCPRED
679	690	NSPRRARSVASQ	12	ABCPRED
477	488	STPCNGVEGFNC	12	ABCPRED
327	338	VRFPNITNLCPF	12	ABCPRED
315	326	TSNFRVQPTESI	12	ABCPRED
298	309	ETKCTLKSFTVE	12	ABCPRED
254	265	SSSGWTAGAAAY	12	ABCPRED
245	256	HRSYLTPGDSSS	12	ABCPRED
158	169	RVYSSANNCTFE	12	ABCPRED
115	126	QSLLIVNNATNV	12	ABCPRED
910	921	GVTQNVLYENQK	12	ABCPRED
842	853	GDIAARDLICAQ	12	ABCPRED
789	800	YKTPPIKDFGGF	12	ABCPRED
755	766	QYGSFCTQLNRA	12	ABCPRED
74	85	NGTKRFDNPVLP	12	ABCPRED
671	682	CASYQTQTNSPR	12	ABCPRED
591	602	SFGGVSVITPGT	12	ABCPRED
554	565	ESNKKFLPFQQF	12	ABCPRED
52	63	QDLFLPFFSNVT	12	ABCPRED
514	525	SFELLHAPATVC	12	ABCPRED
46	57	SVLHSTQDLFLP	12	ABCPRED
405	416	DEVRQIAPGQTG	12	ABCPRED
376	387	TFKCYGVSPTKL	12	ABCPRED
349	360	SVYAWNRKRISN	12	ABCPRED
329	340	FPNITNLCPFGE	12	ABCPRED
312	323	IYQTSNFRVQPT	12	ABCPRED
187	198	KNLREFVFKNID	12	ABCPRED
136	147	CNDPFLGVYYHK	12	ABCPRED
943	954	SALGKLQDVVNQ	12	ABCPRED
931	942	IGKIQDSLSSTA	12	ABCPRED
890	901	AGAALQIPFAMQ	12	ABCPRED
863	874	PLLTDEMIAQYT	12	ABCPRED
699	710	LGAENSVAYSNN	12	ABCPRED
602	613	TNTSNQVAVLYQ	12	ABCPRED
510	521	VVVLSFELLHAP	12	ABCPRED
490	501	FPLQSYGFQPTN	12	ABCPRED
398	409	DSFVIRGDEVRQ	12	ABCPRED
370	381	NSASFSTFKCYG	12	ABCPRED
360	371	NCVADYSVLYNS	12	ABCPRED
347	358	FASVYAWNRKRI	12	ABCPRED
337	348	PFGEVFNATRFA	12	ABCPRED
336	347	CPFGEVFNATRF	12	ABCPRED
283	294	GTITDAVDCALD	12	ABCPRED
171	182	VSQPFLMDLEGK	12	ABCPRED
164	175	NNCTFEYVSQPF	12	ABCPRED
137	148	NDPFLGVYYHKN	12	ABCPRED
122	133	NATNVVIKVCEF	12	ABCPRED
944	955	ALGKLQDVVNQN	12	ABCPRED
888	899	FGAGAALQIPFA	12	ABCPRED
876	887	ALLAGTITSGWT	12	ABCPRED
864	875	LLTDEMIAQYTS	12	ABCPRED
836	847	QYGDCLGDIAAR	12	ABCPRED
820	831	DLLFNKVTLADA	12	ABCPRED
817	828	FIEDLLFNKVTL	12	ABCPRED
808	819	DPSKPSKRSFIE	12	ABCPRED
77	88	KRFDNPVLPFND	12	ABCPRED
732	743	TKTSVDCTMYIC	12	ABCPRED
711	722	SIAIPTNFTISV	12	ABCPRED
7	18	LLPLVSSQCVNL	12	ABCPRED
688	699	ASQSIIAYTMSL	12	ABCPRED
675	686	QTQTNSPRRARS	12	ABCPRED
639	650	GSNVFQTRAGCL	12	ABCPRED
617	628	CTEVPVAIHADQ	12	ABCPRED
575	586	AVRDPQTLEILD	12	ABCPRED
566	577	GRDIADTTDAVR	12	ABCPRED

524	535	VCGPKKSTNLVK	12	ABCPRED
482	493	GVEGFNCYFPLQ	12	ABCPRED
466	477	RDISTEIYQAGS	12	ABCPRED
465	476	ERDISTEIYQAG	12	ABCPRED
416	427	GKIADYNYKLPD	12	ABCPRED
31	42	SFTRGVYYPDKV	12	ABCPRED
30	41	NSFTRGVYYPDK	12	ABCPRED
282	293	NGTITDAVDCAL	12	ABCPRED
278	289	KYNENGTITDAV	12	ABCPRED
267	278	VGYLQPRTFLLK	12	ABCPRED
249	260	LTPGDSSSGWTA	12	ABCPRED
192	203	FVFKNIDGYFKI	12	ABCPRED
148	159	NNKSWMESEFRV	12	ABCPRED
935	946	QDSLSSTASALG	12	ABCPRED
904	915	YRFNGIGVTQNV	12	ABCPRED
898	909	FAMQMAYRFNGI	12	ABCPRED
829	840	ADAGFIKQYGDC	12	ABCPRED
633	644	WRVYSTGSNVFQ	12	ABCPRED
598	609	ITPGTNTSNQVA	12	ABCPRED
513	524	LSFELLHAPATV	12	ABCPRED
506	517	QPYRVVVLSFEL	12	ABCPRED
483	494	VEGFNCYFPLQS	12	ABCPRED
455	466	LFRKSNLKPFER	12	ABCPRED
40	51	DKVFRSSVLHST	12	ABCPRED
356	367	KRISNCVADYSV	12	ABCPRED
300	311	KCTLKSFTVEKG	12	ABCPRED
284	295	TITDAVDCALDP	12	ABCPRED
276	287	LLKYNENGTITD	12	ABCPRED
242	253	LALHRSYLTPGD	12	ABCPRED
230	241	PIGINITRFQTL	12	ABCPRED
188	199	NLREFVFKNIDG	12	ABCPRED
924	935	ANQFNSAIGKIQ	12	ABCPRED
895	906	QIPFAMQMAYRF	12	ABCPRED
878	889	LAGTITSGWTFG	12	ABCPRED
859	870	TVLPPLLTDEMI	12	ABCPRED
841	852	LGDIAARDLICA	12	ABCPRED
81	92	NPVLPFNDGVYF	12	ABCPRED
777	788	NTQEVFAQVKQI	12	ABCPRED
669	680	GICASYQTQTNS	12	ABCPRED
638	649	TGSNVFQTRAGC	12	ABCPRED
610	621	VLYQDVNCTEVP	12	ABCPRED
604	615	TSNQVAVLYQDV	12	ABCPRED
60	71	SNVTWFHAIHVS	12	ABCPRED
55	66	FLPFFSNVTWFH	12	ABCPRED
529	540	KSTNLVKNKCVN	12	ABCPRED
5	16	LVLLPLVSSQCV	12	ABCPRED
472	483	IYQAGSTPCNGV	12	ABCPRED
470	481	TEIYQAGSTPCN	12	ABCPRED
468	479	ISTEIYQAGSTP	12	ABCPRED
454	465	RLFRKSNLKPFE	12	ABCPRED
453	464	YRLFRKSNLKPF	12	ABCPRED
423	434	YKLPDDFTGCVI	12	ABCPRED
364	375	DYSVLYNSASFS	12	ABCPRED
345	356	TRFASVYAWNRK	12	ABCPRED
326	337	IVRFPNITNLCP	12	ABCPRED
32	43	FTRGVYYPDKVF	12	ABCPRED
303	314	LKSFTVEKGIYQ	12	ABCPRED
286	297	TDAVDCALDPLS	12	ABCPRED
279	290	YNENGTITDAVD	12	ABCPRED
21	32	RTQLPPAYTNSF	12	ABCPRED
195	206	KNIDGYFKIYSK	12	ABCPRED
108	119	TTLDSKTQSLLI	12	ABCPRED
10	21	LVSSQCVNLTTR	12	ABCPRED
279	292	YNENGTITDAVDCA	14	ABCPRED
495	508	YGFQPTNGVGYQPY	14	ABCPRED
25	38	PPAYTNSFTRGVYY	14	ABCPRED
416	429	GKIADYNYKLPDDF	14	ABCPRED
374	387	FSTFKCYGVSPTKL	14	ABCPRED
771	784	AVEQDKNTQEVFAQ	14	ABCPRED
726	739	ILPVSMTKTSVDCT	14	ABCPRED
83	96	VLPFNDGVYFASTE	14	ABCPRED
413	426	GQTGKIADYNYKLP	14	ABCPRED
878	891	LAGTITSGWTFGAG	14	ABCPRED

682	695	RRARSVASQSIIAY	14	ABCPRED
647	660	AGCLIGAEHVNNSY	14	ABCPRED
474	487	QAGSTPCNGVEGFN	14	ABCPRED
943	956	SALGKLQDVVNQNA	14	ABCPRED
895	908	QIPFAMQMAYRFNG	14	ABCPRED
70	83	VSGTNGTKRFDNPV	14	ABCPRED
514	527	SFELLHAPATVCGP	14	ABCPRED
345	358	TRFASVYAWNRKRI	14	ABCPRED
330	343	PNITNLCPFGEVFN	14	ABCPRED
194	207	FKNIDGYFKIYSKH	14	ABCPRED
143	156	VYYHKNNKSWMESE	14	ABCPRED
938	951	LSSTASALGKLQDV	14	ABCPRED
640	653	SNVFQTRAGCLIGA	14	ABCPRED
59	72	FSNVTWFHAIHVSG	14	ABCPRED
967	980	SSNFGAISSVLNDI	14	ABCPRED
962	975	LVKQLSSNFGAISS	14	ABCPRED
477	490	STPCNGVEGFNCYF	14	ABCPRED
228	241	DLPIGINITRFQTL	14	ABCPRED
112	125	SKTQSLLIVNNATN	14	ABCPRED
741	754	YICGDSTECSNLLL	14	ABCPRED
666	679	IGAGICASYQTQTN	14	ABCPRED
632	645	TWRVYSTGSNVFQT	14	ABCPRED
55	68	FLPFFSNVTWFHAI	14	ABCPRED
546	559	LTGTGVLTESNKKF	14	ABCPRED
497	510	FQPTNGVGYQPYRV	14	ABCPRED
244	257	LHRSYLTPGDSSSG	14	ABCPRED
650	663	LIGAEHVNNSYECD	14	ABCPRED
563	576	QQFGRDIADTTDAV	14	ABCPRED
331	344	NITNLCPFGEVFNA	14	ABCPRED
281	294	ENGTITDAVDCALD	14	ABCPRED
1093	1106	GVFVSNGTHWFVTQ	14	ABCPRED
913	926	QNVLYENQKLIANQ	14	ABCPRED
903	916	AYRFNGIGVTQNVL	14	ABCPRED
691	704	SIIAYTMSLGAENS	14	ABCPRED
685	698	RSVASQSIIAYTMS	14	ABCPRED
642	655	VFQTRAGCLIGAEH	14	ABCPRED
639	652	GSNVFQTRAGCLIG	14	ABCPRED
583	596	EILDITPCSFGGVS	14	ABCPRED
578	591	DPQTLEILDITPCS	14	ABCPRED
432	445	CVIAWNSNNLDSKV	14	ABCPRED
286	299	TDAVDCALDPLSET	14	ABCPRED
1102	1115	WFVTQRNFYEPQII	14	ABCPRED
889	902	GAGAALQIPFAMQM	14	ABCPRED
772	785	VEQDKNTQEVFAQV	14	ABCPRED
706	719	AYSNNSIAIPTNFT	14	ABCPRED
539	552	VNFNFNGLTGTGVL	14	ABCPRED
346	359	RFASVYAWNRKRIS	14	ABCPRED
981	994	LSRLDKVEAEVQID	14	ABCPRED
945	958	LGKLQDVVNQNAQA	14	ABCPRED
864	877	LLTDEMIAQYTSAL	14	ABCPRED
619	632	EVPVAIHADQLTPT	14	ABCPRED
406	419	EVRQIAPGQTGKIA	14	ABCPRED
224	237	EPLVDLPIGINITR	14	ABCPRED
114	127	TQSLLIVNNATNVV	14	ABCPRED
73	86	TNGTKRFDNPVLPF	14	ABCPRED
585	598	LDITPCSFGGVSVI	14	ABCPRED
531	544	TNLVKNKCVNFNFN	14	ABCPRED
506	519	QPYRVVVLSFELLH	14	ABCPRED
22	35	TQLPPAYTNSFTRG	14	ABCPRED
219	232	GFSALEPLVDLPIG	14	ABCPRED
1059	1072	GVVFLHVTYVPAQE	14	ABCPRED
994	1007	DRLITGRLQSLQTY	14	ABCPRED
788	801	IYKTPPIKDFGGFN	14	ABCPRED
646	659	RAGCLIGAEHVNNS	14	ABCPRED
597	610	VITPGTNTSNQVAV	14	ABCPRED
453	466	YRLFRKSNLKPFER	14	ABCPRED
303	316	LKSFTVEKGIYQTS	14	ABCPRED
268	281	GYLQPRTFLLKYNE	14	ABCPRED
223	236	LEPLVDLPIGINIT	14	ABCPRED
155	168	SEFRVYSSANNCTF	14	ABCPRED
978	991	NDILSRLDKVEAEV	14	ABCPRED
953	966	NQNAQALNTLVKQL	14	ABCPRED
912	925	TQNVLYENQKLIAN	14	ABCPRED

860	873	VLPPLLTDEMIAQY	14	ABCPRED
8	21	LPLVSSQCVNLTTR	14	ABCPRED
769	782	GIAVEQDKNTQEVF	14	ABCPRED
738	751	CTMYICGDSTECSN	14	ABCPRED
469	482	STEIYQAGSTPCNG	14	ABCPRED
254	267	SSSGWTAGAAAYYV	14	ABCPRED
220	233	FSALEPLVDLPIGI	14	ABCPRED
216	229	LPQGFSALEPLVDL	14	ABCPRED
163	176	ANNCTFEYVSQPFL	14	ABCPRED
1001	1014	LQSLQTYVTQQLIR	14	ABCPRED
984	997	LDKVEAEVQIDRLI	14	ABCPRED
983	996	RLDKVEAEVQIDRL	14	ABCPRED
954	967	QNAQALNTLVKQLS	14	ABCPRED
720	733	ISVTTEILPVSMTK	14	ABCPRED
60	73	SNVTWFHAIHVSGT	14	ABCPRED
573	586	TDAVRDPQTLEILD	14	ABCPRED
365	378	YSVLYNSASFSTFK	14	ABCPRED
170	183	YVSQPFLMDLEGKQ	14	ABCPRED
117	130	LLIVNNATNVVIKV	14	ABCPRED
1085	1098	GKAHFPREGVFVSN	14	ABCPRED
949	962	QDVVNQNAQALNTL	14	ABCPRED
621	634	PVAIHADQLTPTWR	14	ABCPRED
431	444	GCVIAWNSNNLDSK	14	ABCPRED
367	380	VLYNSASFSTFKCY	14	ABCPRED
356	369	KRISNCVADYSVLY	14	ABCPRED
353	366	WNRKRISNCVADYS	14	ABCPRED
301	314	CTLKSFTVEKGIYQ	14	ABCPRED
20	33	TRTQLPPAYTNSFT	14	ABCPRED
186	199	FKNLREFVFKNIDG	14	ABCPRED
174	187	PFLMDLEGKQGNFK	14	ABCPRED
15	28	CVNLTTRTQLPPAY	14	ABCPRED
951	964	VVNQNAQALNTLVK	14	ABCPRED
766	779	ALTGIAVEQDKNTQ	14	ABCPRED
713	726	AIPTNFTISVTTEI	14	ABCPRED
695	708	YTMSLGAENSVAYS	14	ABCPRED
670	683	ICASYQTQTNSPRR	14	ABCPRED
418	431	IADYNYKLPDDFTG	14	ABCPRED
362	375	VADYSVLYNSASFS	14	ABCPRED
326	339	IVRFPNITNLCPFG	14	ABCPRED
85	98	PFNDGVYFASTEKS	14	ABCPRED
842	855	GDIAARDLICAQKF	14	ABCPRED
690	703	QSIIAYTMSLGAEN	14	ABCPRED
594	607	GVSVITPGTNTSNQ	14	ABCPRED
560	573	LPFQQFGRDIADTT	14	ABCPRED
553	566	TESNKKFLPFQQFG	14	ABCPRED
519	532	HAPATVCGPKKSTN	14	ABCPRED
500	513	TNGVGYQPYRVVVL	14	ABCPRED
429	442	FTGCVIAWNSNNLD	14	ABCPRED
403	416	RGDEVRQIAPGQTG	14	ABCPRED
325	338	SIVRFPNITNLCPF	14	ABCPRED
284	297	TITDAVDCALDPLS	14	ABCPRED
259	272	TAGAAAYYVGYLQP	14	ABCPRED
1029	1042	MSECVLGQSKRVDF	14	ABCPRED
909	922	IGVTQNVLYENQKL	14	ABCPRED
734	747	TSVDCTMYICGDST	14	ABCPRED
643	656	FQTRAGCLIGAEHV	14	ABCPRED
520	533	APATVCGPKKSTNL	14	ABCPRED
241	254	LLALHRSYLTPGDS	14	ABCPRED
1065	1078	VTYVPAQEKNFTTA	14	ABCPRED
1052	1065	FPQSAPHGVVFLHV	14	ABCPRED
1051	1064	SFPQSAPHGVVFLH	14	ABCPRED
946	959	GKLQDVVNQNAQAL	14	ABCPRED
618	631	TEVPVAIHADQLTP	14	ABCPRED
568	581	DIADTTDAVRDPQT	14	ABCPRED
522	535	ATVCGPKKSTNLVK	14	ABCPRED
295	308	PLSETKCTLKSFTV	14	ABCPRED
257	270	GWTAGAAAYYVGYL	14	ABCPRED
245	258	HRSYLTPGDSSSGW	14	ABCPRED
136	149	CNDPFLGVYYHKNN	14	ABCPRED
988	1001	EAEVQIDRLITGRL	14	ABCPRED
972	985	AISSVLNDILSRLD	14	ABCPRED
968	981	SNFGAISSVLNDIL	14	ABCPRED
941	954	TASALGKLQDVVNQ	14	ABCPRED

813	826	SKRSFIEDLLFNKV	14	ABCPRED
81	94	NPVLPFNDGVYFAS	14	ABCPRED
795	808	KDFGGFNFSQILPD	14	ABCPRED
754	767	LQYGSFCTQLNRAL	14	ABCPRED
742	755	ICGDSTECSNLLLQ	14	ABCPRED
736	749	VDCTMYICGDSTEC	14	ABCPRED
683	696	RARSVASQSIIAYT	14	ABCPRED
534	547	VKNKCVNFNFNGLT	14	ABCPRED
377	390	FKCYGVSPTKLNDL	14	ABCPRED
327	340	VRFPNITNLCPFGE	14	ABCPRED
242	255	LALHRSYLTPGDSS	14	ABCPRED
221	234	SALEPLVDLPIGIN	14	ABCPRED
172	185	SQPFLMDLEGKQGN	14	ABCPRED
129	142	KVCEFQFCNDPFLG	14	ABCPRED
959	972	LNTLVKQLSSNFGA	14	ABCPRED
937	950	SLSSTASALGKLQD	14	ABCPRED
890	903	AGAALQIPFAMQMA	14	ABCPRED
705	718	VAYSNNSIAIPTNF	14	ABCPRED
571	584	DTTDAVRDPQTLEI	14	ABCPRED
312	325	IYQTSNFRVQPTES	14	ABCPRED
291	304	CALDPLSETKCTLK	14	ABCPRED
252	265	GDSSSGWTAGAAAY	14	ABCPRED
246	259	RSYLTPGDSSSGWT	14	ABCPRED
1103	1116	FVTQRNFYEPQIIT	14	ABCPRED
1082	1095	CHDGKAHFPREGVF	14	ABCPRED
1019	1032	RASANLAATKMSEC	14	ABCPRED
782	795	FAQVKQIYKTPPIK	14	ABCPRED
77	90	KRFDNPVLPFNDGV	14	ABCPRED
687	700	VASQSIIAYTMSLG	14	ABCPRED
672	685	ASYQTQTNSPRRAR	14	ABCPRED
665	678	PIGAGICASYQTQT	14	ABCPRED
660	673	YECDIPIGAGICAS	14	ABCPRED
66	79	HAIHVSGTNGTKRF	14	ABCPRED
58	71	FFSNVTWFHAIHVS	14	ABCPRED
551	564	VLTESNKKFLPFQQ	14	ABCPRED
479	492	PCNGVEGFNCYFPL	14	ABCPRED
436	449	WNSNNLDSKVGGNY	14	ABCPRED
383	396	SPTKLNDLCFTNVY	14	ABCPRED
298	311	ETKCTLKSFTVEKG	14	ABCPRED
253	266	DSSSGWTAGAAAYY	14	ABCPRED
213	226	VRDLPQGFSALEPL	14	ABCPRED
1100	1113	THWFVTQRNFYEPQ	14	ABCPRED
1035	1048	GQSKRVDFCGKGYH	14	ABCPRED
993	1006	IDRLITGRLQSLQT	14	ABCPRED
906	919	FNGIGVTQNVLYEN	14	ABCPRED
886	899	WTFGAGAALQIPFA	14	ABCPRED
870	883	IAQYTSALLAGTIT	14	ABCPRED
87	100	NDGVYFASTEKSNI	14	ABCPRED
740	753	MYICGDSTECSNLL	14	ABCPRED
644	657	QTRAGCLIGAEHVN	14	ABCPRED
559	572	FLPFQQFGRDIADT	14	ABCPRED
547	560	TGTGVLTESNKKFL	14	ABCPRED
444	457	KVGGNYNYLYRLFR	14	ABCPRED
352	365	AWNRKRISNCVADY	14	ABCPRED
290	303	DCALDPLSETKCTL	14	ABCPRED
260	273	AGAAAYYVGYLQPR	14	ABCPRED
255	268	SSGWTAGAAAYYVG	14	ABCPRED
167	180	TFEYVSQPFLMDLE	14	ABCPRED
1089	1102	FPREGVFVSNGTHW	14	ABCPRED
1064	1077	HVTYVPAQEKNFTT	14	ABCPRED
1053	1066	PQSAPHGVVFLHVT	14	ABCPRED
935	948	QDSLSSTASALGKL	14	ABCPRED
922	935	LIANQFNSAIGKIQ	14	ABCPRED
91	104	YFASTEKSNIIRGW	14	ABCPRED
679	692	NSPRRARSVASQSI	14	ABCPRED
607	620	QVAVLYQDVNCTEV	14	ABCPRED
537	550	KCVNFNFNGLTGTG	14	ABCPRED
513	526	LSFELLHAPATVCG	14	ABCPRED
50	63	STQDLFLPFFSNVT	14	ABCPRED
461	474	LKPFERDISTEIYQ	14	ABCPRED
389	402	DLCFTNVYADSFVI	14	ABCPRED
370	383	NSASFSTFKCYGVS	14	ABCPRED
351	364	YAWNRKRISNCVAD	14	ABCPRED

180	193	EGKQGNFKNLREFV	14	ABCPRED
128	141	IKVCEFQFCNDPFL	14	ABCPRED
1057	1070	PHGVVFLHVTYVPA	14	ABCPRED
1047	1060	YHLMSFPQSAPHGV	14	ABCPRED
1018	1031	IRASANLAATKMSE	14	ABCPRED
819	832	EDLLFNKVTLADAG	14	ABCPRED
774	787	QDKNTQEVFAQVKQ	14	ABCPRED
731	744	MTKTSVDCTMYICG	14	ABCPRED
540	553	NFNFNGLTGTGVLT	14	ABCPRED
501	514	NGVGYQPYRVVVLS	14	ABCPRED
240	253	TLLALHRSYLTPGD	14	ABCPRED
169	182	EYVSQPFLMDLEGK	14	ABCPRED
1096	1109	VSNGTHWFVTQRNF	14	ABCPRED
1081	1094	ICHDGKAHFPREGV	14	ABCPRED
108	121	TTLDSKTQSLLIVN	14	ABCPRED
1026	1039	ATKMSECVLGQSKR	14	ABCPRED
1025	1038	AATKMSECVLGQSK	14	ABCPRED
1009	1022	TQQLIRAAEIRASA	14	ABCPRED
999	1012	GRLQSLQTYVTQQL	14	ABCPRED
947	960	KLQDVVNQNAQALN	14	ABCPRED
910	923	GVTQNVLYENQKLI	14	ABCPRED
892	905	AALQIPFAMQMAYR	14	ABCPRED
863	876	PLLTDEMIAQYTSA	14	ABCPRED
861	874	LPPLLTDEMIAQYT	14	ABCPRED
783	796	AQVKQIYKTPPIKD	14	ABCPRED
756	769	YGSFCTQLNRALTG	14	ABCPRED
633	646	WRVYSTGSNVFQTR	14	ABCPRED
626	639	ADQLTPTWRVYSTG	14	ABCPRED
491	504	PLQSYGFQPTNGVG	14	ABCPRED
404	417	GDEVRQIAPGQTGK	14	ABCPRED
400	413	FVIRGDEVRQIAPG	14	ABCPRED
332	345	ITNLCPFGEVFNAT	14	ABCPRED
300	313	KCTLKSFTVEKGIY	14	ABCPRED
1078	1091	APAICHDGKAHFPR	14	ABCPRED
973	986	ISSVLNDILSRLDK	14	ABCPRED
942	955	ASALGKLQDVVNQN	14	ABCPRED
9	22	PLVSSQCVNLTTRT	14	ABCPRED
89	102	GVYFASTEKSNIIR	14	ABCPRED
885	898	GWTFGAGAALQIPF	14	ABCPRED
881	894	TITSGWTFGAGAAL	14	ABCPRED
832	845	GFIKQYGDCLGDIA	14	ABCPRED
789	802	YKTPPIKDFGGFNF	14	ABCPRED
775	788	DKNTQEVFAQVKQI	14	ABCPRED
610	623	VLYQDVNCTEVPVA	14	ABCPRED
584	597	ILDITPCSFGGVSV	14	ABCPRED
57	70	PFFSNVTWFHAIHV	14	ABCPRED
54	67	LFLPFFSNVTWFHA	14	ABCPRED
423	436	YKLPDDFTGCVIAW	14	ABCPRED
373	386	SFSTFKCYGVSPTK	14	ABCPRED
237	250	RFQTLLALHRSYLT	14	ABCPRED
229	242	LPIGINITRFQTLL	14	ABCPRED
157	170	FRVYSSANNCTFEY	14	ABCPRED
1033	1046	VLGQSKRVDFCGKG	14	ABCPRED
915	928	VLYENQKLIANQFN	14	ABCPRED
907	920	NGIGVTQNVLYENQ	14	ABCPRED
762	775	QLNRALTGIAVEQD	14	ABCPRED
612	625	YQDVNCTEVPVAIH	14	ABCPRED
496	509	GFQPTNGVGYQPYR	14	ABCPRED
464	477	FERDISTEIYQAGS	14	ABCPRED
460	473	NLKPFERDISTEIY	14	ABCPRED
44	57	RSSVLHSTQDLFLP	14	ABCPRED
35	48	GVYYPDKVFRSSVL	14	ABCPRED
309	322	EKGIYQTSNFRVQP	14	ABCPRED
275	288	FLLKYNENGTITDA	14	ABCPRED
247	260	SYLTPGDSSSGWTA	14	ABCPRED
144	157	YYHKNNKSWMESEF	14	ABCPRED
1080	1093	AICHDGKAHFPREG	14	ABCPRED
1077	1090	TAPAICHDGKAHFP	14	ABCPRED
1069	1082	PAQEKNFTTAPAIC	14	ABCPRED
103	116	GWIFGTTLDSKTQS	14	ABCPRED
833	846	FIKQYGDCLGDIAA	14	ABCPRED
828	841	LADAGFIKQYGDCL	14	ABCPRED
780	793	EVFAQVKQIYKTPP	14	ABCPRED

755	768	QYGSFCTQLNRALT	14	ABCPRED
753	766	LLQYGSFCTQLNRA	14	ABCPRED
667	680	GAGICASYQTQTNS	14	ABCPRED
63	76	TWFHAIHVSGTNGT	14	ABCPRED
595	608	VSVITPGTNTSNQV	14	ABCPRED
536	549	NKCVNFNFNGLTGT	14	ABCPRED
528	541	KKSTNLVKNKCVNF	14	ABCPRED
523	536	TVCGPKKSTNLVKN	14	ABCPRED
402	415	IRGDEVRQIAPGQT	14	ABCPRED
372	385	ASFSTFKCYGVSPT	14	ABCPRED
359	372	SNCVADYSVLYNSA	14	ABCPRED
358	371	ISNCVADYSVLYNS	14	ABCPRED
321	334	QPTESIVRFPNITN	14	ABCPRED
21	34	RTQLPPAYTNSFTR	14	ABCPRED
151	164	SWMESEFRVYSSAN	14	ABCPRED
1021	1034	SANLAATKMSECVL	14	ABCPRED
979	992	DILSRLDKVEAEVQ	14	ABCPRED
961	974	TLVKQLSSNFGAIS	14	ABCPRED
896	909	IPFAMQMAYRFNGI	14	ABCPRED
86	99	FNDGVYFASTEKSN	14	ABCPRED
846	859	ARDLICAQKFNGLT	14	ABCPRED
823	836	FNKVTLADAGFIKQ	14	ABCPRED
797	810	FGGFNFSQILPDPS	14	ABCPRED
768	781	TGIAVEQDKNTQEV	14	ABCPRED
728	741	PVSMTKTSVDCTMY	14	ABCPRED
689	702	SQSIIAYTMSLGAE	14	ABCPRED
67	80	AIHVSGTNGTKRFD	14	ABCPRED
652	665	GAEHVNNSYECDIP	14	ABCPRED
611	624	LYQDVNCTEVPVAI	14	ABCPRED
572	585	TTDAVRDPQTLEIL	14	ABCPRED
535	548	KNKCVNFNFNGLTG	14	ABCPRED
53	66	DLFLPFFSNVTWFH	14	ABCPRED
502	515	GVGYQPYRVVVLSF	14	ABCPRED
48	61	LHSTQDLFLPFFSN	14	ABCPRED
337	350	PFGEVFNATRFASV	14	ABCPRED
304	317	KSFTVEKGIYQTSN	14	ABCPRED
299	312	TKCTLKSFTVEKGI	14	ABCPRED
273	286	RTFLLKYNENGTIT	14	ABCPRED
121	134	NNATNVVIKVCEFQ	14	ABCPRED
987	1000	VEAEVQIDRLITGR	14	ABCPRED
975	988	SVLNDILSRLDKVE	14	ABCPRED
927	940	FNSAIGKIQDSLSS	14	ABCPRED
883	896	TSGWTFGAGAALQI	14	ABCPRED
841	854	LGDIAARDLICAQK	14	ABCPRED
814	827	KRSFIEDLLFNKVT	14	ABCPRED
763	776	LNRALTGIAVEQDK	14	ABCPRED
725	738	EILPVSMTKTSVDC	14	ABCPRED
420	433	DYNYKLPDDFTGCV	14	ABCPRED
410	423	IAPGQTGKIADYNY	14	ABCPRED
187	200	KNLREFVFKNIDGY	14	ABCPRED
165	178	NCTFEYVSQPFLMD	14	ABCPRED
1084	1097	DGKAHFPREGVFVS	14	ABCPRED
1083	1096	HDGKAHFPREGVFV	14	ABCPRED
90	103	VYFASTEKSNIIRG	14	ABCPRED
853	866	QKFNGLTVLPPLLT	14	ABCPRED
804	817	QILPDPSKPSKRSF	14	ABCPRED
761	774	TQLNRALTGIAVEQ	14	ABCPRED
711	724	SIAIPTNFTISVTT	14	ABCPRED
631	644	PTWRVYSTGSNVFQ	14	ABCPRED
604	617	TSNQVAVLYQDVNC	14	ABCPRED
602	615	TNTSNQVAVLYQDV	14	ABCPRED
570	583	ADTTDAVRDPQTLE	14	ABCPRED
38	51	YPDKVFRSSVLHST	14	ABCPRED
364	377	DYSVLYNSASFSTF	14	ABCPRED
339	352	GEVFNATRFASVYA	14	ABCPRED
287	300	DAVDCALDPLSETK	14	ABCPRED
262	275	AAAYYVGYLQPRTF	14	ABCPRED
227	240	VDLPIGINITRFQT	14	ABCPRED
197	210	IDGYFKIYSKHTPI	14	ABCPRED
152	165	WMESEFRVYSSANN	14	ABCPRED
131	144	CEFQFCNDPFLGVY	14	ABCPRED
977	990	LNDILSRLDKVEAE	14	ABCPRED
974	987	SSVLNDILSRLDKV	14	ABCPRED

960	973	NTLVKQLSSNFGAI	14	ABCPRED
92	105	FASTEKSNIIRGWI	14	ABCPRED
859	872	TVLPPLLTDEMIAQ	14	ABCPRED
831	844	AGFIKQYGDCLGDI	14	ABCPRED
824	837	NKVTLADAGFIKQY	14	ABCPRED
790	803	KTPPIKDFGGFNFS	14	ABCPRED
634	647	RVYSTGSNVFQTRA	14	ABCPRED
588	601	TPCSFGGVSVITPG	14	ABCPRED
576	589	VRDPQTLEILDITP	14	ABCPRED
564	577	QFGRDIADTTDAVR	14	ABCPRED
562	575	FQQFGRDIADTTDA	14	ABCPRED
51	64	TQDLFLPFFSNVTW	14	ABCPRED
509	522	RVVVLSFELLHAPA	14	ABCPRED
507	520	PYRVVVLSFELLHA	14	ABCPRED
471	484	EIYQAGSTPCNGVE	14	ABCPRED
371	384	SASFSTFKCYGVSP	14	ABCPRED
340	353	EVFNATRFASVYAW	14	ABCPRED
338	351	FGEVFNATRFASVY	14	ABCPRED
310	323	KGIYQTSNFRVQPT	14	ABCPRED
308	321	VEKGIYQTSNFRVQ	14	ABCPRED
24	37	LPPAYTNSFTRGVY	14	ABCPRED
214	227	RDLPQGFSALEPLV	14	ABCPRED
1088	1101	HFPREGVFVSNGTH	14	ABCPRED
101	114	IRGWIFGTTLDSKT	14	ABCPRED
916	929	LYENQKLIANQFNS	14	ABCPRED
879	892	AGTITSGWTFGAGA	14	ABCPRED
871	884	AQYTSALLAGTITS	14	ABCPRED
837	850	YGDCLGDIAARDLI	14	ABCPRED
798	811	GGFNFSQILPDPSK	14	ABCPRED
678	691	TNSPRRARSVASQS	14	ABCPRED
637	650	STGSNVFQTRAGCL	14	ABCPRED
628	641	QLTPTWRVYSTGSN	14	ABCPRED
590	603	CSFGGVSVITPGTN	14	ABCPRED
552	565	LTESNKKFLPFQQF	14	ABCPRED
470	483	TEIYQAGSTPCNGV	14	ABCPRED
390	403	LCFTNVYADSFVIR	14	ABCPRED
288	301	AVDCALDPLSETKC	14	ABCPRED
274	287	TFLLKYNENGTITD	14	ABCPRED
204	217	YSKHTPINLVRDLP	14	ABCPRED
201	214	FKIYSKHTPINLVR	14	ABCPRED
156	169	EFRVYSSANNCTFE	14	ABCPRED
1092	1105	EGVFVSNGTHWFVT	14	ABCPRED
1087	1100	AHFPREGVFVSNGT	14	ABCPRED
1067	1080	YVPAQEKNFTTAPA	14	ABCPRED
1031	1044	ECVLGQSKRVDFCG	14	ABCPRED
1008	1021	VTQQLIRAAEIRAS	14	ABCPRED
1003	1016	SLQTYVTQQLIRAA	14	ABCPRED
965	978	QLSSNFGAISSVLN	14	ABCPRED
96	109	EKSNIIRGWIFGTT	14	ABCPRED
924	937	ANQFNSAIGKIQDS	14	ABCPRED
894	907	LQIPFAMQMAYRFN	14	ABCPRED
829	842	ADAGFIKQYGDCLG	14	ABCPRED
784	797	QVKQIYKTPPIKDF	14	ABCPRED
730	743	SMTKTSVDCTMYIC	14	ABCPRED
721	734	SVTTEILPVSMTKT	14	ABCPRED
72	85	GTNGTKRFDNPVLP	14	ABCPRED
707	720	YSNNSIAIPTNFTI	14	ABCPRED
669	682	GICASYQTQTNSPR	14	ABCPRED
648	661	GCLIGAEHVNNSYE	14	ABCPRED
569	582	IADTTDAVRDPQTL	14	ABCPRED
475	488	AGSTPCNGVEGFNC	14	ABCPRED
468	481	ISTEIYQAGSTPCN	14	ABCPRED
458	471	KSNLKPFERDISTE	14	ABCPRED
408	421	RQIAPGQTGKIADY	14	ABCPRED
342	355	FNATRFASVYAWNR	14	ABCPRED
17	30	NLTTRTQLPPAYTN	14	ABCPRED
160	173	YSSANNCTFEYVSQ	14	ABCPRED
135	148	FCNDPFLGVYYHKN	14	ABCPRED
125	138	NVVIKVCEFQFCND	14	ABCPRED
1050	1063	MSFPQSAPHGVVFL	14	ABCPRED
917	930	YENQKLIANQFNSA	14	ABCPRED
857	870	GLTVLPPLLTDEMI	14	ABCPRED
82	95	PVLPFNDGVYFAST	14	ABCPRED

818	831	IEDLLFNKVTLADA	14	ABCPRED
806	819	LPDPSKPSKRSFIE	14	ABCPRED
76	89	TKRFDNPVLPFNDG	14	ABCPRED
677	690	QTNSPRRARSVASQ	14	ABCPRED
65	78	FHAIHVSGTNGTKR	14	ABCPRED
605	618	SNQVAVLYQDVNCT	14	ABCPRED
577	590	RDPQTLEILDITPC	14	ABCPRED
530	543	STNLVKNKCVNFNF	14	ABCPRED
447	460	GNYNYLYRLFRKSN	14	ABCPRED
422	435	NYKLPDDFTGCVIA	14	ABCPRED
292	305	ALDPLSETKCTLKS	14	ABCPRED
203	216	IYSKHTPINLVRDL	14	ABCPRED
179	192	LEGKQGNFKNLREF	14	ABCPRED
1094	1107	VFVSNGTHWFVTQR	14	ABCPRED
100	113	IIRGWIFGTTLDSK	14	ABCPRED
963	976	VKQLSSNFGAISSV	14	ABCPRED
805	818	ILPDPSKPSKRSFI	14	ABCPRED
773	786	EQDKNTQEVFAQVK	14	ABCPRED
760	773	CTQLNRALTGIAVE	14	ABCPRED
735	748	SVDCTMYICGDSTE	14	ABCPRED
673	686	SYQTQTNSPRRARS	14	ABCPRED
587	600	ITPCSFGGVSVITP	14	ABCPRED
579	592	PQTLEILDITPCSF	14	ABCPRED
556	569	NKKFLPFQQFGRDI	14	ABCPRED
493	506	QSYGFQPTNGVGYQ	14	ABCPRED
47	60	VLHSTQDLFLPFFS	14	ABCPRED
414	427	QTGKIADYNYKLPD	14	ABCPRED
348	361	ASVYAWNRKRISNC	14	ABCPRED
315	328	TSNFRVQPTESIVR	14	ABCPRED
134	147	QFCNDPFLGVYYHK	14	ABCPRED
111	124	DSKTQSLLIVNNAT	14	ABCPRED
1091	1104	REGVFVSNGTHWFV	14	ABCPRED
1063	1076	LHVTYVPAQEKNFT	14	ABCPRED
10	23	LVSSQCVNLTTRTQ	14	ABCPRED
991	1004	VQIDRLITGRLQSL	14	ABCPRED
856	869	NGLTVLPPLLTDEM	14	ABCPRED
844	857	IAARDLICAQKFNG	14	ABCPRED
786	799	KQIYKTPPIKDFGG	14	ABCPRED
714	727	IPTNFTISVTTEIL	14	ABCPRED
697	710	MSLGAENSVAYSNN	14	ABCPRED
694	707	AYTMSLGAENSVAY	14	ABCPRED
680	693	SPRRARSVASQSII	14	ABCPRED
625	638	HADQLTPTWRVYST	14	ABCPRED
606	619	NQVAVLYQDVNCTE	14	ABCPRED
581	594	TLEILDITPCSFGG	14	ABCPRED
554	567	ESNKKFLPFQQFGR	14	ABCPRED
508	521	YRVVVLSFELLHAP	14	ABCPRED
450	463	NYLYRLFRKSNLKP	14	ABCPRED
409	422	QIAPGQTGKIADYN	14	ABCPRED
347	360	FASVYAWNRKRISN	14	ABCPRED
28	41	YTNSFTRGVYYPDK	14	ABCPRED
277	290	LKYNENGTITDAVD	14	ABCPRED
276	289	LLKYNENGTITDAV	14	ABCPRED
199	212	GYFKIYSKHTPINL	14	ABCPRED
189	202	LREFVFKNIDGYFK	14	ABCPRED
130	143	VCEFQFCNDPFLGV	14	ABCPRED
879	894	AGTITSGWTFGAGAAL	16	ABCPRED
594	609	GVSVITPGTNTSNQVA	16	ABCPRED
257	272	GWTAGAAAYYVGYLQP	16	ABCPRED
1112	1127	PQIITTDNTFVSGNCD	16	ABCPRED
245	260	HRSYLTPGDSSSGWTA	16	ABCPRED
1180	1195	QKEIDRLNEVAKNLNE	16	ABCPRED
476	491	GSTPCNGVEGFNCYFP	16	ABCPRED
307	322	TVEKGIYQTSNFRVQP	16	ABCPRED
648	663	GCLIGAEHVNNSYECD	16	ABCPRED
492	507	LQSYGFQPTNGVGYQP	16	ABCPRED
1247	1262	CCSCGSCCKFDEDDSE	16	ABCPRED
470	485	TEIYQAGSTPCNGVEG	16	ABCPRED
236	251	TRFQTLLALHRSYLTP	16	ABCPRED
1206	1221	YEQYIKWPWYIWLGFI	16	ABCPRED
1058	1073	HGVVFLHVTYVPAQEK	16	ABCPRED
931	946	IGKIQDSLSSTASALG	16	ABCPRED
898	913	FAMQMAYRFNGIGVTQ	16	ABCPRED

882	897	ITSGWTFGAGAALQIP	16	ABCPRED
1064	1079	HVTYVPAQEKNFTTAP	16	ABCPRED
786	801	KQIYKTPPIKDFGGFN	16	ABCPRED
473	488	YQAGSTPCNGVEGFNC	16	ABCPRED
525	540	CGPKKSTNLVKNKCVN	16	ABCPRED
464	479	FERDISTEIYQAGSTP	16	ABCPRED
266	281	YVGYLQPRTFLLKYNE	16	ABCPRED
151	166	SWMESEFRVYSSANNC	16	ABCPRED
1240	1255	CCSCLKGCCSCGSCCK	16	ABCPRED
881	896	TITSGWTFGAGAALQI	16	ABCPRED
732	747	TKTSVDCTMYICGDST	16	ABCPRED
406	421	EVRQIAPGQTGKIADY	16	ABCPRED
391	406	CFTNVYADSFVIRGDE	16	ABCPRED
310	325	KGIYQTSNFRVQPTES	16	ABCPRED
152	167	WMESEFRVYSSANNCT	16	ABCPRED
1050	1065	MSFPQSAPHGVVFLHV	16	ABCPRED
97	112	KSNIIRGWIFGTTLDS	16	ABCPRED
741	756	YICGDSTECSNLLLQY	16	ABCPRED
739	754	TMYICGDSTECSNLLL	16	ABCPRED
630	645	TPTWRVYSTGSNVFQT	16	ABCPRED
415	430	TGKIADYNYKLPDDFT	16	ABCPRED
200	215	YFKIYSKHTPINLVRD	16	ABCPRED
1234	1249	LCCMTSCCSCLKGCCS	16	ABCPRED
1084	1099	DGKAHFPREGVFVSNG	16	ABCPRED
897	912	PFAMQMAYRFNGIGVT	16	ABCPRED
847	862	RDLICAQKFNGLTVLP	16	ABCPRED
604	619	TSNQVAVLYQDVNCTE	16	ABCPRED
280	295	NENGTITDAVDCALDP	16	ABCPRED
1211	1226	KWPWYIWLGFIAGLIA	16	ABCPRED
1195	1210	ESLIDLQELGKYEQYI	16	ABCPRED
1129	1144	VIGIVNNTVYDPLQPE	16	ABCPRED
101	116	IRGWIFGTTLDSKTQS	16	ABCPRED
70	85	VSGTNGTKRFDNPVLP	16	ABCPRED
689	704	SQSIIAYTMSLGAENS	16	ABCPRED
627	642	DQLTPTWRVYSTGSNV	16	ABCPRED
600	615	PGTNTSNQVAVLYQDV	16	ABCPRED
564	579	QFGRDIADTTDAVRDP	16	ABCPRED
329	344	FPNITNLCPFGEVFNA	16	ABCPRED
900	915	MQMAYRFNGIGVTQNV	16	ABCPRED
791	806	TPPIKDFGGFNFSQIL	16	ABCPRED
735	750	SVDCTMYICGDSTECS	16	ABCPRED
347	362	FASVYAWNRKRISNCV	16	ABCPRED
309	324	EKGIYQTSNFRVQPTE	16	ABCPRED
260	275	AGAAAYYVGYLQPRTF	16	ABCPRED
1209	1224	YIKWPWYIWLGFIAGL	16	ABCPRED
1041	1056	DFCGKGYHLMSFPQSA	16	ABCPRED
936	951	DSLSSTASALGKLQDV	16	ABCPRED
803	818	SQILPDPSKPSKRSFI	16	ABCPRED
674	689	YQTQTNSPRRARSVAS	16	ABCPRED
634	649	RVYSTGSNVFQTRAGC	16	ABCPRED
583	598	EILDITPCSFGGVSVI	16	ABCPRED
49	64	HSTQDLFLPFFSNVTW	16	ABCPRED
412	427	PGQTGKIADYNYKLPD	16	ABCPRED
320	335	VQPTESIVRFPNITNL	16	ABCPRED
288	303	AVDCALDPLSETKCTL	16	ABCPRED
194	209	FKNIDGYFKIYSKHTP	16	ABCPRED
124	139	TNVVIKVCEFQFCNDP	16	ABCPRED
829	844	ADAGFIKQYGDCLGDI	16	ABCPRED
798	813	GGFNFSQILPDPSKPS	16	ABCPRED
71	86	SGTNGTKRFDNPVLPF	16	ABCPRED
67	82	AIHVSGTNGTKRFDNP	16	ABCPRED
625	640	HADQLTPTWRVYSTGS	16	ABCPRED
360	375	NCVADYSVLYNSASFS	16	ABCPRED
19	34	TTRTQLPPAYTNSFTR	16	ABCPRED
159	174	VYSSANNCTFEYVSQP	16	ABCPRED
148	163	NNKSWMESEFRVYSSA	16	ABCPRED
688	703	ASQSIIAYTMSLGAEN	16	ABCPRED
653	668	AEHVNNSYECDIPIGA	16	ABCPRED
566	581	GRDIADTTDAVRDPQT	16	ABCPRED
1250	1265	CGSCCKFDEDDSEPVL	16	ABCPRED
1233	1248	MLCCMTSCCSCLKGCC	16	ABCPRED
1134	1149	NNTVYDPLQPELDSFK	16	ABCPRED
107	122	GTTLDSKTQSLLIVNN	16	ABCPRED

994	1009	DRLITGRLQSLQTYVT	16	ABCPRED
719	734	TISVTTEILPVSMTKT	16	ABCPRED
662	677	CDIPIGAGICASYQTQ	16	ABCPRED
657	672	NNSYECDIPIGAGICA	16	ABCPRED
526	541	GPKKSTNLVKNKCVNF	16	ABCPRED
521	536	PATVCGPKKSTNLVKN	16	ABCPRED
501	516	NGVGYQPYRVVVLSFE	16	ABCPRED
270	285	LQPRTFLLKYNENGTI	16	ABCPRED
24	39	LPPAYTNSFTRGVYYP	16	ABCPRED
1241	1256	CSCLKGCCSCGSCCKF	16	ABCPRED
1227	1242	IVMVTIMLCCMTSCCS	16	ABCPRED
102	117	RGWIFGTTLDSKTQSL	16	ABCPRED
941	956	TASALGKLQDVVNQNA	16	ABCPRED
838	853	GDCLGDIAARDLICAQ	16	ABCPRED
709	724	NNSIAIPTNFTISVTT	16	ABCPRED
695	710	YTMSLGAENSVAYSNN	16	ABCPRED
596	611	SVITPGTNTSNQVAVL	16	ABCPRED
517	532	LLHAPATVCGPKKSTN	16	ABCPRED
374	389	FSTFKCYGVSPTKLND	16	ABCPRED
301	316	CTLKSFTVEKGIYQTS	16	ABCPRED
251	266	PGDSSSGWTAGAAAYY	16	ABCPRED
153	168	MESEFRVYSSANNCTF	16	ABCPRED
1146	1161	DSFKEELDKYFKNHTS	16	ABCPRED
778	793	TQEVFAQVKQIYKTPP	16	ABCPRED
666	681	IGAGICASYQTQTNSP	16	ABCPRED
333	348	TNLCPFGEVFNATRFA	16	ABCPRED
1147	1162	SFKEELDKYFKNHTSP	16	ABCPRED
1139	1154	DPLQPELDSFKEELDK	16	ABCPRED
1110	1125	YEPQIITTDNTFVSGN	16	ABCPRED
815	830	RSFIEDLLFNKVTLAD	16	ABCPRED
765	780	RALTGIAVEQDKNTQE	16	ABCPRED
722	737	VTTEILPVSMTKTSVD	16	ABCPRED
673	688	SYQTQTNSPRRARSVA	16	ABCPRED
663	678	DIPIGAGICASYQTQT	16	ABCPRED
475	490	AGSTPCNGVEGFNCYF	16	ABCPRED
413	428	GQTGKIADYNYKLPDD	16	ABCPRED
377	392	FKCYGVSPTKLNDLCF	16	ABCPRED
366	381	SVLYNSASFSTFKCYG	16	ABCPRED
34	49	RGVYYPDKVFRSSVLH	16	ABCPRED
300	315	KCTLKSFTVEKGIYQT	16	ABCPRED
282	297	NGTITDAVDCALDPLS	16	ABCPRED
1103	1118	FVTQRNFYEPQIITTD	16	ABCPRED
988	1003	EAEVQIDRLITGRLQS	16	ABCPRED
860	875	VLPPLLTDEMIAQYTS	16	ABCPRED
841	856	LGDIAARDLICAQKFN	16	ABCPRED
65	80	FHAIHVSGTNGTKRFD	16	ABCPRED
649	664	CLIGAEHVNNSYECDI	16	ABCPRED
622	637	VAIHADQLTPTWRVYS	16	ABCPRED
547	562	TGTGVLTESNKKFLPF	16	ABCPRED
520	535	APATVCGPKKSTNLVK	16	ABCPRED
484	499	EGFNCYFPLQSYGFQP	16	ABCPRED
411	426	APGQTGKIADYNYKLP	16	ABCPRED
379	394	CYGVSPTKLNDLCFTN	16	ABCPRED
348	363	ASVYAWNRKRISNCVA	16	ABCPRED
331	346	NITNLCPFGEVFNATR	16	ABCPRED
136	151	CNDPFLGVYYHKNNKS	16	ABCPRED
1245	1260	KGCCSCGSCCKFDEDD	16	ABCPRED
1176	1191	VVNIQKEIDRLNEVAK	16	ABCPRED
1166	1181	LGDISGINASVVNIQK	16	ABCPRED
1009	1024	TQQLIRAAEIRASANL	16	ABCPRED
88	103	DGVYFASTEKSNIIRG	16	ABCPRED
747	762	TECSNLLLQYGSFCTQ	16	ABCPRED
545	560	GLTGTGVLTESNKKFL	16	ABCPRED
544	559	NGLTGTGVLTESNKKF	16	ABCPRED
343	358	NATRFASVYAWNRKRI	16	ABCPRED
319	334	RVQPTESIVRFPNITN	16	ABCPRED
249	264	LTPGDSSSGWTAGAAA	16	ABCPRED
149	164	NKSWMESEFRVYSSAN	16	ABCPRED
133	148	FQFCNDPFLGVYYHKN	16	ABCPRED
1239	1254	SCCSCLKGCCSCGSCC	16	ABCPRED
1094	1109	VFVSNGTHWFVTQRNF	16	ABCPRED
990	1005	EVQIDRLITGRLQSLQ	16	ABCPRED
953	968	NQNAQALNTLVKQLSS	16	ABCPRED

833	848	FIKQYGDCLGDIAARD	16	ABCPRED
770	785	IAVEQDKNTQEVFAQV	16	ABCPRED
727	742	LPVSMTKTSVDCTMYI	16	ABCPRED
636	651	YSTGSNVFQTRAGCLI	16	ABCPRED
631	646	PTWRVYSTGSNVFQTR	16	ABCPRED
539	554	VNFNFNGLTGTGVLTE	16	ABCPRED
445	460	VGGNYNYLYRLFRKSN	16	ABCPRED
430	445	TGCVIAWNSNNLDSKV	16	ABCPRED
423	438	YKLPDDFTGCVIAWNS	16	ABCPRED
355	370	RKRISNCVADYSVLYN	16	ABCPRED
1198	1213	IDLQELGKYEQYIKWP	16	ABCPRED
1062	1077	FLHVTYVPAQEKNFTT	16	ABCPRED
1001	1016	LQSLQTYVTQQLIRAA	16	ABCPRED
729	744	VSMTKTSVDCTMYICG	16	ABCPRED
700	715	GAENSVAYSNNSIAIP	16	ABCPRED
671	686	CASYQTQTNSPRRARS	16	ABCPRED
624	639	IHADQLTPTWRVYSTG	16	ABCPRED
612	627	YQDVNCTEVPVAIHAD	16	ABCPRED
448	463	NYNYLYRLFRKSNLKP	16	ABCPRED
444	459	KVGGNYNYLYRLFRKS	16	ABCPRED
432	447	CVIAWNSNNLDSKVGG	16	ABCPRED
369	384	YNSASFSTFKCYGVSP	16	ABCPRED
323	338	TESIVRFPNITNLCPF	16	ABCPRED
322	337	PTESIVRFPNITNLCP	16	ABCPRED
289	304	VDCALDPLSETKCTLK	16	ABCPRED
25	40	PPAYTNSFTRGVYYPD	16	ABCPRED
1237	1252	MTSCCSCLKGCCSCGS	16	ABCPRED
1213	1228	PWYIWLGFIAGLIAIV	16	ABCPRED
1121	1136	FVSGNCDVVIGIVNNT	16	ABCPRED
1007	1022	YVTQQLIRAAEIRASA	16	ABCPRED
890	905	AGAALQIPFAMQMAYR	16	ABCPRED
89	104	GVYFASTEKSNIIRGW	16	ABCPRED
768	783	TGIAVEQDKNTQEVFA	16	ABCPRED
696	711	TMSLGAENSVAYSNNS	16	ABCPRED
690	705	QSIIAYTMSLGAENSV	16	ABCPRED
472	487	IYQAGSTPCNGVEGFN	16	ABCPRED
428	443	DFTGCVIAWNSNNLDS	16	ABCPRED
400	415	FVIRGDEVRQIAPGQT	16	ABCPRED
378	393	KCYGVSPTKLNDLCFT	16	ABCPRED
141	156	LGVYYHKNNKSWMESE	16	ABCPRED
1153	1168	DKYFKNHTSPDVDLGD	16	ABCPRED
1078	1093	APAICHDGKAHFPREG	16	ABCPRED
998	1013	TGRLQSLQTYVTQQLI	16	ABCPRED
831	846	AGFIKQYGDCLGDIAA	16	ABCPRED
814	829	KRSFIEDLLFNKVTLA	16	ABCPRED
61	76	NVTWFHAIHVSGTNGT	16	ABCPRED
565	580	FGRDIADTTDAVRDPQ	16	ABCPRED
375	390	STFKCYGVSPTKLNDL	16	ABCPRED
345	360	TRFASVYAWNRKRISN	16	ABCPRED
294	309	DPLSETKCTLKSFTVE	16	ABCPRED
254	269	SSSGWTAGAAAYYVGY	16	ABCPRED
244	259	LHRSYLTPGDSSSGWT	16	ABCPRED
240	255	TLLALHRSYLTPGDSS	16	ABCPRED
1179	1194	IQKEIDRLNEVAKNLN	16	ABCPRED
1111	1126	EPQIITTDNTFVSGNC	16	ABCPRED
939	954	SSTASALGKLQDVVNQ	16	ABCPRED
574	589	DAVRDPQTLEILDITP	16	ABCPRED
573	588	TDAVRDPQTLEILDIT	16	ABCPRED
500	515	TNGVGYQPYRVVVLSF	16	ABCPRED
410	425	IAPGQTGKIADYNYKL	16	ABCPRED
403	418	RGDEVRQIAPGQTGKI	16	ABCPRED
339	354	GEVFNATRFASVYAWN	16	ABCPRED
1246	1261	GCCSCGSCCKFDEDDS	16	ABCPRED
1128	1143	VVIGIVNNTVYDPLQP	16	ABCPRED
1101	1116	HWFVTQRNFYEPQIIT	16	ABCPRED
1067	1082	YVPAQEKNFTTAPAIC	16	ABCPRED
1051	1066	SFPQSAPHGVVFLHVT	16	ABCPRED
910	925	GVTQNVLYENQKLIAN	16	ABCPRED
846	861	ARDLICAQKFNGLTVL	16	ABCPRED
771	786	AVEQDKNTQEVFAQVK	16	ABCPRED
740	755	MYICGDSTECSNLLLQ	16	ABCPRED
731	746	MTKTSVDCTMYICGDS	16	ABCPRED
568	583	DIADTTDAVRDPQTLE	16	ABCPRED

567	582	RDIADTTDAVRDPQTL	16	ABCPRED
523	538	TVCGPKKSTNLVKNKC	16	ABCPRED
511	526	VVLSFELLHAPATVCG	16	ABCPRED
467	482	DISTEIYQAGSTPCNG	16	ABCPRED
414	429	QTGKIADYNYKLPDDF	16	ABCPRED
398	413	DSFVIRGDEVRQIAPG	16	ABCPRED
35	50	GVYYPDKVFRSSVLHS	16	ABCPRED
338	353	FGEVFNATRFASVYAW	16	ABCPRED
3	18	VFLVLLPLVSSQCVNL	16	ABCPRED
297	312	SETKCTLKSFTVEKGI	16	ABCPRED
286	301	TDAVDCALDPLSETKC	16	ABCPRED
263	278	AAYYVGYLQPRTFLLK	16	ABCPRED
1161	1176	SPDVDLGDISGINASV	16	ABCPRED
1159	1174	HTSPDVDLGDISGINA	16	ABCPRED
1105	1120	TQRNFYEPQIITTDNT	16	ABCPRED
1042	1057	FCGKGYHLMSFPQSAP	16	ABCPRED
909	924	IGVTQNVLYENQKLIA	16	ABCPRED
908	923	GIGVTQNVLYENQKLI	16	ABCPRED
873	888	YTSALLAGTITSGWTF	16	ABCPRED
868	883	EMIAQYTSALLAGTIT	16	ABCPRED
728	743	PVSMTKTSVDCTMYIC	16	ABCPRED
675	690	QTQTNSPRRARSVASQ	16	ABCPRED
609	624	AVLYQDVNCTEVPVAI	16	ABCPRED
606	621	NQVAVLYQDVNCTEVP	16	ABCPRED
519	534	HAPATVCGPKKSTNLV	16	ABCPRED
485	500	GFNCYFPLQSYGFQPT	16	ABCPRED
457	472	RKSNLKPFERDISTEI	16	ABCPRED
442	457	DSKVGGNYNYLYRLFR	16	ABCPRED
394	409	NVYADSFVIRGDEVRQ	16	ABCPRED
224	239	EPLVDLPIGINITRFQ	16	ABCPRED
174	189	PFLMDLEGKQGNFKNL	16	ABCPRED
1251	1266	GSCCKFDEDDSEPVLK	16	ABCPRED
1163	1178	DVDLGDISGINASVVN	16	ABCPRED
11	26	VSSQCVNLTTRTQLPP	16	ABCPRED
1063	1078	LHVTYVPAQEKNFTTA	16	ABCPRED
975	990	SVLNDILSRLDKVEAE	16	ABCPRED
801	816	NFSQILPDPSKPSKRS	16	ABCPRED
736	751	VDCTMYICGDSTECSN	16	ABCPRED
584	599	ILDITPCSFGGVSVIT	16	ABCPRED
447	462	GNYNYLYRLFRKSNLK	16	ABCPRED
401	416	VIRGDEVRQIAPGQTG	16	ABCPRED
139	154	PFLGVYYHKNNKSWME	16	ABCPRED
1257	1272	DEDDSEPVLKGVKLHY	16	ABCPRED
1229	1244	MVTIMLCCMTSCCSCL	16	ABCPRED
1205	1220	KYEQYIKWPWYIWLGF	16	ABCPRED
1120	1135	TFVSGNCDVVIGIVNN	16	ABCPRED
110	125	LDSKTQSLLIVNNATN	16	ABCPRED
1088	1103	HFPREGVFVSNGTHWF	16	ABCPRED
1087	1102	AHFPREGVFVSNGTHW	16	ABCPRED
1074	1089	NFTTAPAICHDGKAHF	16	ABCPRED
1047	1062	YHLMSFPQSAPHGVVF	16	ABCPRED
964	979	KQLSSNFGAISSVLND	16	ABCPRED
907	922	NGIGVTQNVLYENQKL	16	ABCPRED
839	854	DCLGDIAARDLICAQK	16	ABCPRED
82	97	PVLPFNDGVYFASTEK	16	ABCPRED
787	802	QIYKTPPIKDFGGFNF	16	ABCPRED
784	799	QVKQIYKTPPIKDFGG	16	ABCPRED
76	91	TKRFDNPVLPFNDGVY	16	ABCPRED
699	714	LGAENSVAYSNNSIAI	16	ABCPRED
670	685	ICASYQTQTNSPRRAR	16	ABCPRED
664	679	IPIGAGICASYQTQTN	16	ABCPRED
661	676	ECDIPIGAGICASYQT	16	ABCPRED
647	662	AGCLIGAEHVNNSYEC	16	ABCPRED
643	658	FQTRAGCLIGAEHVNN	16	ABCPRED
591	606	SFGGVSVITPGTNTSN	16	ABCPRED
399	414	SFVIRGDEVRQIAPGQ	16	ABCPRED
295	310	PLSETKCTLKSFTVEK	16	ABCPRED
171	186	VSQPFLMDLEGKQGNF	16	ABCPRED
14	29	QCVNLTTRTQLPPAYT	16	ABCPRED
1155	1170	YFKNHTSPDVDLGDIS	16	ABCPRED
1097	1112	SNGTHWFVTQRNFYEP	16	ABCPRED
989	1004	AEVQIDRLITGRLQSL	16	ABCPRED
95	110	TEKSNIIRGWIFGTTL	16	ABCPRED

904	919	YRFNGIGVTQNVLYEN	16	ABCPRED
806	821	LPDPSKPSKRSFIEDL	16	ABCPRED
766	781	ALTGIAVEQDKNTQEV	16	ABCPRED
737	752	DCTMYICGDSTECSNL	16	ABCPRED
659	674	SYECDIPIGAGICASY	16	ABCPRED
615	630	VNCTEVPVAIHADQLT	16	ABCPRED
588	603	TPCSFGGVSVITPGTN	16	ABCPRED
486	501	FNCYFPLQSYGFQPTN	16	ABCPRED
434	449	IAWNSNNLDSKVGGNY	16	ABCPRED
420	435	DYNYKLPDDFTGCVIA	16	ABCPRED
384	399	PTKLNDLCFTNVYADS	16	ABCPRED
256	271	SGWTAGAAAYYVGYLQ	16	ABCPRED
146	161	HKNNKSWMESEFRVYS	16	ABCPRED
1228	1243	VMVTIMLCCMTSCCSC	16	ABCPRED
1082	1097	CHDGKAHFPREGVFVS	16	ABCPRED
1080	1095	AICHDGKAHFPREGVF	16	ABCPRED
1053	1068	PQSAPHGVVFLHVTYV	16	ABCPRED
98	113	SNIIRGWIFGTTLDSK	16	ABCPRED
945	960	LGKLQDVVNQNAQALN	16	ABCPRED
867	882	DEMIAQYTSALLAGTI	16	ABCPRED
866	881	TDEMIAQYTSALLAGT	16	ABCPRED
832	847	GFIKQYGDCLGDIAAR	16	ABCPRED
745	760	DSTECSNLLLQYGSFC	16	ABCPRED
581	596	TLEILDITPCSFGGVS	16	ABCPRED
550	565	GVLTESNKKFLPFQQF	16	ABCPRED
546	561	LTGTGVLTESNKKFLP	16	ABCPRED
498	513	QPTNGVGYQPYRVVVL	16	ABCPRED
468	483	ISTEIYQAGSTPCNGV	16	ABCPRED
43	58	FRSSVLHSTQDLFLPF	16	ABCPRED
308	323	VEKGIYQTSNFRVQPT	16	ABCPRED
168	183	FEYVSQPFLMDLEGKQ	16	ABCPRED
1254	1269	CKFDEDDSEPVLKGVK	16	ABCPRED
1207	1222	EQYIKWPWYIWLGFIA	16	ABCPRED
1081	1096	ICHDGKAHFPREGVFV	16	ABCPRED
1032	1047	CVLGQSKRVDFCGKGY	16	ABCPRED
1026	1041	ATKMSECVLGQSKRVD	16	ABCPRED
725	740	EILPVSMTKTSVDCTM	16	ABCPRED
710	725	NSIAIPTNFTISVTTE	16	ABCPRED
658	673	NSYECDIPIGAGICAS	16	ABCPRED
482	497	GVEGFNCYFPLQSYGF	16	ABCPRED
453	468	YRLFRKSNLKPFERDI	16	ABCPRED
383	398	SPTKLNDLCFTNVYAD	16	ABCPRED
23	38	QLPPAYTNSFTRGVYY	16	ABCPRED
138	153	DPFLGVYYHKNNKSWM	16	ABCPRED
1201	1216	QELGKYEQYIKWPWYI	16	ABCPRED
1143	1158	PELDSFKEELDKYFKN	16	ABCPRED
1109	1124	FYEPQIITTDNTFVSG	16	ABCPRED
1086	1101	KAHFPREGVFVSNGTH	16	ABCPRED
1079	1094	PAICHDGKAHFPREGV	16	ABCPRED
1043	1058	CGKGYHLMSFPQSAPH	16	ABCPRED
1037	1052	SKRVDFCGKGYHLMSF	16	ABCPRED
1029	1044	MSECVLGQSKRVDFCG	16	ABCPRED
1020	1035	ASANLAATKMSECVLG	16	ABCPRED
1010	1025	QQLIRAAEIRASANLA	16	ABCPRED
943	958	SALGKLQDVVNQNAQA	16	ABCPRED
887	902	TFGAGAALQIPFAMQM	16	ABCPRED
857	872	GLTVLPPLLTDEMIAQ	16	ABCPRED
783	798	AQVKQIYKTPPIKDFG	16	ABCPRED
717	732	NFTISVTTEILPVSMT	16	ABCPRED
676	691	TQTNSPRRARSVASQS	16	ABCPRED
646	661	RAGCLIGAEHVNNSYE	16	ABCPRED
592	607	FGGVSVITPGTNTSNQ	16	ABCPRED
589	604	PCSFGGVSVITPGTNT	16	ABCPRED
456	471	FRKSNLKPFERDISTE	16	ABCPRED
450	465	NYLYRLFRKSNLKPFE	16	ABCPRED
405	420	DEVRQIAPGQTGKIAD	16	ABCPRED
371	386	SASFSTFKCYGVSPTK	16	ABCPRED
346	361	RFASVYAWNRKRISNC	16	ABCPRED
316	331	SNFRVQPTESIVRFPN	16	ABCPRED
31	46	SFTRGVYYPDKVFRSS	16	ABCPRED
258	273	WTAGAAAYYVGYLQPR	16	ABCPRED
255	270	SSGWTAGAAAYYVGYL	16	ABCPRED
219	234	GFSALEPLVDLPIGIN	16	ABCPRED

210	225	INLVRDLPQGFSALEP	16	ABCPRED
1252	1267	SCCKFDEDDSEPVLKG	16	ABCPRED
1203	1218	LGKYEQYIKWPWYIWL	16	ABCPRED
1169	1184	ISGINASVVNIQKEID	16	ABCPRED
1164	1179	VDLGDISGINASVVNI	16	ABCPRED
112	127	SKTQSLLIVNNATNVV	16	ABCPRED
1098	1113	NGTHWFVTQRNFYEPQ	16	ABCPRED
1083	1098	HDGKAHFPREGVFVSN	16	ABCPRED
1015	1030	AAEIRASANLAATKMS	16	ABCPRED
100	115	IIRGWIFGTTLDSKTQ	16	ABCPRED
973	988	ISSVLNDILSRLDKVE	16	ABCPRED
837	852	YGDCLGDIAARDLICA	16	ABCPRED
826	841	VTLADAGFIKQYGDCL	16	ABCPRED
8	23	LPLVSSQCVNLTTRTQ	16	ABCPRED
777	792	NTQEVFAQVKQIYKTP	16	ABCPRED
750	765	SNLLLQYGSFCTQLNR	16	ABCPRED
724	739	TEILPVSMTKTSVDCT	16	ABCPRED
469	484	STEIYQAGSTPCNGVE	16	ABCPRED
418	433	IADYNYKLPDDFTGCV	16	ABCPRED
32	47	FTRGVYYPDKVFRSSV	16	ABCPRED
252	267	GDSSSGWTAGAAAYYV	16	ABCPRED
228	243	DLPIGINITRFQTLLA	16	ABCPRED
134	149	QFCNDPFLGVYYHKNN	16	ABCPRED
1017	1032	EIRASANLAATKMSEC	16	ABCPRED
946	961	GKLQDVVNQNAQALNT	16	ABCPRED
808	823	DPSKPSKRSFIEDLLF	16	ABCPRED
802	817	FSQILPDPSKPSKRSF	16	ABCPRED
757	772	GSFCTQLNRALTGIAV	16	ABCPRED
74	89	NGTKRFDNPVLPFNDG	16	ABCPRED
730	745	SMTKTSVDCTMYICGD	16	ABCPRED
697	712	MSLGAENSVAYSNNSI	16	ABCPRED
593	608	GGVSVITPGTNTSNQV	16	ABCPRED
515	530	FELLHAPATVCGPKKS	16	ABCPRED
493	508	QSYGFQPTNGVGYQPY	16	ABCPRED
317	332	NFRVQPTESIVRFPNI	16	ABCPRED
305	320	SFTVEKGIYQTSNFRV	16	ABCPRED
277	292	LKYNENGTITDAVDCA	16	ABCPRED
242	257	LALHRSYLTPGDSSSG	16	ABCPRED
202	217	KIYSKHTPINLVRDLP	16	ABCPRED
193	208	VFKNIDGYFKIYSKHT	16	ABCPRED
182	197	KQGNFKNLREFVFKNI	16	ABCPRED
17	32	NLTTRTQLPPAYTNSF	16	ABCPRED
154	169	ESEFRVYSSANNCTFE	16	ABCPRED
1255	1270	KFDEDDSEPVLKGVKL	16	ABCPRED
1075	1090	FTTAPAICHDGKAHFP	16	ABCPRED
914	929	NVLYENQKLIANQFNS	16	ABCPRED
672	687	ASYQTQTNSPRRARSV	16	ABCPRED
628	643	QLTPTWRVYSTGSNVF	16	ABCPRED
582	597	LEILDITPCSFGGVSV	16	ABCPRED
572	587	TTDAVRDPQTLEILDI	16	ABCPRED
437	452	NSNNLDSKVGGNYNYL	16	ABCPRED
272	287	PRTFLLKYNENGTITD	16	ABCPRED
262	277	AAAYYVGYLQPRTFLL	16	ABCPRED
248	263	YLTPGDSSSGWTAGAA	16	ABCPRED
1114	1129	IITTDNTFVSGNCDVV	16	ABCPRED
1069	1084	PAQEKNFTTAPAICHD	16	ABCPRED
1048	1063	HLMSFPQSAPHGVVFL	16	ABCPRED
1027	1042	TKMSECVLGQSKRVDF	16	ABCPRED
970	985	FGAISSVLNDILSRLD	16	ABCPRED
961	976	TLVKQLSSNFGAISSV	16	ABCPRED
932	947	GKIQDSLSSTASALGK	16	ABCPRED
835	850	KQYGDCLGDIAARDLI	16	ABCPRED
824	839	NKVTLADAGFIKQYGD	16	ABCPRED
773	788	EQDKNTQEVFAQVKQI	16	ABCPRED
68	83	IHVSGTNGTKRFDNPV	16	ABCPRED
522	537	ATVCGPKKSTNLVKNK	16	ABCPRED
407	422	VRQIAPGQTGKIADYN	16	ABCPRED
372	387	ASFSTFKCYGVSPTKL	16	ABCPRED
359	374	SNCVADYSVLYNSASF	16	ABCPRED
354	369	NRKRISNCVADYSVLY	16	ABCPRED
349	364	SVYAWNRKRISNCVAD	16	ABCPRED
201	216	FKIYSKHTPINLVRDL	16	ABCPRED
181	196	GKQGNFKNLREFVFKN	16	ABCPRED

1243	1258	CLKGCCSCGSCCKFDE	16	ABCPRED
118	133	LIVNNATNVVIKVCEF	16	ABCPRED
984	999	LDKVEAEVQIDRLITG	16	ABCPRED
93	108	ASTEKSNIIRGWIFGT	16	ABCPRED
922	937	LIANQFNSAIGKIQDS	16	ABCPRED
893	908	ALQIPFAMQMAYRFNG	16	ABCPRED
760	775	CTQLNRALTGIAVEQD	16	ABCPRED
686	701	SVASQSIIAYTMSLGA	16	ABCPRED
66	81	HAIHVSGTNGTKRFDN	16	ABCPRED
629	644	LTPTWRVYSTGSNVFQ	16	ABCPRED
617	632	CTEVPVAIHADQLTPT	16	ABCPRED
518	533	LHAPATVCGPKKSTNL	16	ABCPRED
496	511	GFQPTNGVGYQPYRVV	16	ABCPRED
490	505	FPLQSYGFQPTNGVGY	16	ABCPRED
351	366	YAWNRKRISNCVADYS	16	ABCPRED
328	343	RFPNITNLCPFGEVFN	16	ABCPRED
28	43	YTNSFTRGVYYPDKVF	16	ABCPRED
191	206	EFVFKNIDGYFKIYSK	16	ABCPRED
16	31	VNLTTRTQLPPAYTNS	16	ABCPRED
150	165	KSWMESEFRVYSSANN	16	ABCPRED
131	146	CEFQFCNDPFLGVYYH	16	ABCPRED
128	143	IKVCEFQFCNDPFLGV	16	ABCPRED
1173	1188	NASVVNIQKEIDRLNE	16	ABCPRED
1168	1183	DISGINASVVNIQKEI	16	ABCPRED
1148	1163	FKEELDKYFKNHTSPD	16	ABCPRED
1099	1114	GTHWFVTQRNFYEPQI	16	ABCPRED
1023	1038	NLAATKMSECVLGQSK	16	ABCPRED
844	859	IAARDLICAQKFNGLT	16	ABCPRED
825	840	KVTLADAGFIKQYGDC	16	ABCPRED
763	778	LNRALTGIAVEQDKNT	16	ABCPRED
761	776	TQLNRALTGIAVEQDK	16	ABCPRED
693	708	IAYTMSLGAENSVAYS	16	ABCPRED
639	654	GSNVFQTRAGCLIGAE	16	ABCPRED
63	78	TWFHAIHVSGTNGTKR	16	ABCPRED
580	595	QTLEILDITPCSFGGV	16	ABCPRED
549	564	TGVLTESNKKFLPFQQ	16	ABCPRED
495	510	YGFQPTNGVGYQPYRV	16	ABCPRED
408	423	RQIAPGQTGKIADYNY	16	ABCPRED
381	396	GVSPTKLNDLCFTNVY	16	ABCPRED
313	328	YQTSNFRVQPTESIVR	16	ABCPRED
26	41	PAYTNSFTRGVYYPDK	16	ABCPRED
178	193	DLEGKQGNFKNLREFV	16	ABCPRED
1235	1250	CCMTSCCSCLKGCCSC	16	ABCPRED
1223	1238	GLIAIVMVTIMLCCMT	16	ABCPRED
1204	1219	GKYEQYIKWPWYIWLG	16	ABCPRED
1158	1173	NHTSPDVDLGDISGIN	16	ABCPRED
111	126	DSKTQSLLIVNNATNV	16	ABCPRED
1060	1075	VVFLHVTYVPAQEKNF	16	ABCPRED
1019	1034	RASANLAATKMSECVL	16	ABCPRED
1002	1017	QSLQTYVTQQLIRAAE	16	ABCPRED
10	25	LVSSQCVNLTTRTQLP	16	ABCPRED
969	984	NFGAISSVLNDILSRL	16	ABCPRED
928	943	NSAIGKIQDSLSSTAS	16	ABCPRED
792	807	PPIKDFGGFNFSQILP	16	ABCPRED
789	804	YKTPPIKDFGGFNFSQ	16	ABCPRED
69	84	HVSGTNGTKRFDNPVL	16	ABCPRED
642	657	VFQTRAGCLIGAEHVN	16	ABCPRED
603	618	NTSNQVAVLYQDVNCT	16	ABCPRED
598	613	ITPGTNTSNQVAVLYQ	16	ABCPRED
585	600	LDITPCSFGGVSVITP	16	ABCPRED
56	71	LPFFSNVTWFHAIHVS	16	ABCPRED
516	531	ELLHAPATVCGPKKST	16	ABCPRED
489	504	YFPLQSYGFQPTNGVG	16	ABCPRED
465	480	ERDISTEIYQAGSTPC	16	ABCPRED
425	440	LPDDFTGCVIAWNSNN	16	ABCPRED
388	403	NDLCFTNVYADSFVIR	16	ABCPRED
315	330	TSNFRVQPTESIVRFP	16	ABCPRED
172	187	SQPFLMDLEGKQGNFK	16	ABCPRED
1253	1268	CCKFDEDDSEPVLKGV	16	ABCPRED
1236	1251	CMTSCCSCLKGCCSCG	16	ABCPRED
1194	1209	NESLIDLQELGKYEQY	16	ABCPRED
1187	1202	NEVAKNLNESLIDLQE	16	ABCPRED
1030	1045	SECVLGQSKRVDFCGK	16	ABCPRED

1025	1040	AATKMSECVLGQSKRV	16	ABCPRED
977	992	LNDILSRLDKVEAEVQ	16	ABCPRED
927	942	FNSAIGKIQDSLSSTA	16	ABCPRED
888	903	FGAGAALQIPFAMQMA	16	ABCPRED
828	843	LADAGFIKQYGDCLGD	16	ABCPRED
738	753	CTMYICGDSTECSNLL	16	ABCPRED
733	748	KTSVDCTMYICGDSTE	16	ABCPRED
632	647	TWRVYSTGSNVFQTRA	16	ABCPRED
587	602	ITPCSFGGVSVITPGT	16	ABCPRED
529	544	KSTNLVKNKCVNFNFN	16	ABCPRED
462	477	KPFERDISTEIYQAGS	16	ABCPRED
422	437	NYKLPDDFTGCVIAWN	16	ABCPRED
417	432	KIADYNYKLPDDFTGC	16	ABCPRED
380	395	YGVSPTKLNDLCFTNV	16	ABCPRED
356	371	KRISNCVADYSVLYNS	16	ABCPRED
33	48	TRGVYYPDKVFRSSVL	16	ABCPRED
290	305	DCALDPLSETKCTLKS	16	ABCPRED
22	37	TQLPPAYTNSFTRGVY	16	ABCPRED
216	231	LPQGFSALEPLVDLPI	16	ABCPRED
160	175	YSSANNCTFEYVSQPF	16	ABCPRED
1258	1273	EDDSEPVLKGVKLHYT	16	ABCPRED
1248	1263	CSCGSCCKFDEDDSEP	16	ABCPRED
1220	1235	FIAGLIAIVMVTIMLC	16	ABCPRED
1200	1215	LQELGKYEQYIKWPWY	16	ABCPRED
109	124	TLDSKTQSLLIVNNAT	16	ABCPRED
1034	1049	LGQSKRVDFCGKGYHL	16	ABCPRED
1012	1027	LIRAAEIRASANLAAT	16	ABCPRED
159	176	VYSSANNCTFEYVSQPFL	18	ABCPRED
423	440	YKLPDDFTGCVIAWNSNN	18	ABCPRED
868	885	EMIAQYTSALLAGTITSG	18	ABCPRED
317	334	NFRVQPTESIVRFPNITN	18	ABCPRED
634	651	RVYSTGSNVFQTRAGCLI	18	ABCPRED
530	547	STNLVKNKCVNFNFNGLT	18	ABCPRED
526	543	GPKKSTNLVKNKCVNFNF	18	ABCPRED
1159	1176	HTSPDVDLGDISGINASV	18	ABCPRED
367	384	VLYNSASFSTFKCYGVSP	18	ABCPRED
1181	1198	KEIDRLNEVAKNLNESLI	18	ABCPRED
968	985	SNFGAISSVLNDILSRLD	18	ABCPRED
217	234	PQGFSALEPLVDLPIGIN	18	ABCPRED
188	205	NLREFVFKNIDGYFKIYS	18	ABCPRED
727	744	LPVSMTKTSVDCTMYICG	18	ABCPRED
652	669	GAEHVNNSYECDIPIGAG	18	ABCPRED
447	464	GNYNYLYRLFRKSNLKPF	18	ABCPRED
842	859	GDIAARDLICAQKFNGLT	18	ABCPRED
725	742	EILPVSMTKTSVDCTMYI	18	ABCPRED
635	652	VYSTGSNVFQTRAGCLIG	18	ABCPRED
601	618	GTNTSNQVAVLYQDVNCT	18	ABCPRED
495	512	YGFQPTNGVGYQPYRVVV	18	ABCPRED
142	159	GVYYHKNNKSWMESEFRV	18	ABCPRED
1227	1244	IVMVTIMLCCMTSCCSCL	18	ABCPRED
1128	1145	VVIGIVNNTVYDPLQPEL	18	ABCPRED
1008	1025	VTQQLIRAAEIRASANLA	18	ABCPRED
732	749	TKTSVDCTMYICGDSTEC	18	ABCPRED
568	585	DIADTTDAVRDPQTLEIL	18	ABCPRED
35	52	GVYYPDKVFRSSVLHSTQ	18	ABCPRED
1240	1257	CCSCLKGCCSCGSCCKFD	18	ABCPRED
94	111	STEKSNIIRGWIFGTTLD	18	ABCPRED
798	815	GGFNFSQILPDPSKPSKR	18	ABCPRED
674	691	YQTQTNSPRRARSVASQS	18	ABCPRED
590	607	CSFGGVSVITPGTNTSNQ	18	ABCPRED
533	550	LVKNKCVNFNFNGLTGTG	18	ABCPRED
515	532	FELLHAPATVCGPKKSTN	18	ABCPRED
41	58	KVFRSSVLHSTQDLFLPF	18	ABCPRED
938	955	LSSTASALGKLQDVVNQN	18	ABCPRED
873	890	YTSALLAGTITSGWTFGA	18	ABCPRED
458	475	KSNLKPFERDISTEIYQA	18	ABCPRED
346	363	RFASVYAWNRKRISNCVA	18	ABCPRED
168	185	FEYVSQPFLMDLEGKQGN	18	ABCPRED
1206	1223	YEQYIKWPWYIWLGFIAG	18	ABCPRED
693	710	IAYTMSLGAENSVAYSNN	18	ABCPRED
589	606	PCSFGGVSVITPGTNTSN	18	ABCPRED
385	402	TKLNDLCFTNVYADSFVI	18	ABCPRED
264	281	AYYVGYLQPRTFLLKYNE	18	ABCPRED

191	208	EFVFKNIDGYFKIYSKHT	18	ABCPRED
160	177	YSSANNCTFEYVSQPFLM	18	ABCPRED
1199	1216	DLQELGKYEQYIKWPWYI	18	ABCPRED
1105	1122	TQRNFYEPQIITTDNTFV	18	ABCPRED
1059	1076	GVVFLHVTYVPAQEKNFT	18	ABCPRED
957	974	QALNTLVKQLSSNFGAIS	18	ABCPRED
910	927	GVTQNVLYENQKLIANQF	18	ABCPRED
756	773	YGSFCTQLNRALTGIAVE	18	ABCPRED
613	630	QDVNCTEVPVAIHADQLT	18	ABCPRED
607	624	QVAVLYQDVNCTEVPVAI	18	ABCPRED
599	616	TPGTNTSNQVAVLYQDVN	18	ABCPRED
198	215	DGYFKIYSKHTPINLVRD	18	ABCPRED
161	178	SSANNCTFEYVSQPFLMD	18	ABCPRED
134	151	QFCNDPFLGVYYHKNNKS	18	ABCPRED
1090	1107	PREGVFVSNGTHWFVTQR	18	ABCPRED
866	883	TDEMIAQYTSALLAGTIT	18	ABCPRED
758	775	SFCTQLNRALTGIAVEQD	18	ABCPRED
695	712	YTMSLGAENSVAYSNNSI	18	ABCPRED
669	686	GICASYQTQTNSPRRARS	18	ABCPRED
633	650	WRVYSTGSNVFQTRAGCL	18	ABCPRED
227	244	VDLPIGINITRFQTLLAL	18	ABCPRED
143	160	VYYHKNNKSWMESEFRVY	18	ABCPRED
1160	1177	TSPDVDLGDISGINASVV	18	ABCPRED
1101	1118	HWFVTQRNFYEPQIITTD	18	ABCPRED
1072	1089	EKNFTTAPAICHDGKAHF	18	ABCPRED
1057	1074	PHGVVFLHVTYVPAQEKN	18	ABCPRED
838	855	GDCLGDIAARDLICAQKF	18	ABCPRED
724	741	TEILPVSMTKTSVDCTMY	18	ABCPRED
684	701	ARSVASQSIIAYTMSLGA	18	ABCPRED
631	648	PTWRVYSTGSNVFQTRAG	18	ABCPRED
559	576	FLPFQQFGRDIADTTDAV	18	ABCPRED
498	515	QPTNGVGYQPYRVVVLSF	18	ABCPRED
475	492	AGSTPCNGVEGFNCYFPL	18	ABCPRED
473	490	YQAGSTPCNGVEGFNCYF	18	ABCPRED
44	61	RSSVLHSTQDLFLPFFSN	18	ABCPRED
25	42	PPAYTNSFTRGVYYPDKV	18	ABCPRED
202	219	KIYSKHTPINLVRDLPQG	18	ABCPRED
146	163	HKNNKSWMESEFRVYSSA	18	ABCPRED
1161	1178	SPDVDLGDISGINASVVN	18	ABCPRED
1110	1127	YEPQIITTDNTFVSGNCD	18	ABCPRED
884	901	SGWTFGAGAALQIPFAMQ	18	ABCPRED
874	891	TSALLAGTITSGWTFGAG	18	ABCPRED
870	887	IAQYTSALLAGTITSGWT	18	ABCPRED
743	760	CGDSTECSNLLLQYGSFC	18	ABCPRED
70	87	VSGTNGTKRFDNPVLPFN	18	ABCPRED
543	560	FNGLTGTGVLTESNKKFL	18	ABCPRED
396	413	YADSFVIRGDEVRQIAPG	18	ABCPRED
200	217	YFKIYSKHTPINLVRDLP	18	ABCPRED
1228	1245	VMVTIMLCCMTSCCSCLK	18	ABCPRED
1210	1227	IKWPWYIWLGFIAGLIAI	18	ABCPRED
1157	1174	KNHTSPDVDLGDISGINA	18	ABCPRED
1100	1117	THWFVTQRNFYEPQIITT	18	ABCPRED
1031	1048	ECVLGQSKRVDFCGKGYH	18	ABCPRED
1018	1035	IRASANLAATKMSECVLG	18	ABCPRED
953	970	NQNAQALNTLVKQLSSNF	18	ABCPRED
949	966	QDVVNQNAQALNTLVKQL	18	ABCPRED
819	836	EDLLFNKVTLADAGFIKQ	18	ABCPRED
66	83	HAIHVSGTNGTKRFDNPV	18	ABCPRED
482	499	GVEGFNCYFPLQSYGFQP	18	ABCPRED
360	377	NCVADYSVLYNSASFSTF	18	ABCPRED
218	235	QGFSALEPLVDLPIGINI	18	ABCPRED
1196	1213	SLIDLQELGKYEQYIKWP	18	ABCPRED
1119	1136	NTFVSGNCDVVIGIVNNT	18	ABCPRED
1055	1072	SAPHGVVFLHVTYVPAQE	18	ABCPRED
1052	1069	FPQSAPHGVVFLHVTYVP	18	ABCPRED
951	968	VVNQNAQALNTLVKQLSS	18	ABCPRED
935	952	QDSLSSTASALGKLQDVV	18	ABCPRED
676	693	TQTNSPRRARSVASQSII	18	ABCPRED
427	444	DDFTGCVIAWNSNNLDSK	18	ABCPRED
361	378	CVADYSVLYNSASFSTFK	18	ABCPRED
347	364	FASVYAWNRKRISNCVAD	18	ABCPRED
1202	1219	ELGKYEQYIKWPWYIWLG	18	ABCPRED
869	886	MIAQYTSALLAGTITSGW	18	ABCPRED

780	797	EVFAQVKQIYKTPPIKDF	18	ABCPRED
722	739	VTTEILPVSMTKTSVDCT	18	ABCPRED
678	695	TNSPRRARSVASQSIIAY	18	ABCPRED
632	649	TWRVYSTGSNVFQTRAGC	18	ABCPRED
449	466	YNYLYRLFRKSNLKPFER	18	ABCPRED
426	443	PDDFTGCVIAWNSNNLDS	18	ABCPRED
378	395	KCYGVSPTKLNDLCFTNV	18	ABCPRED
319	336	RVQPTESIVRFPNITNLC	18	ABCPRED
314	331	QTSNFRVQPTESIVRFPN	18	ABCPRED
195	212	KNIDGYFKIYSKHTPINL	18	ABCPRED
192	209	FVFKNIDGYFKIYSKHTP	18	ABCPRED
1246	1263	GCCSCGSCCKFDEDDSEP	18	ABCPRED
122	139	NATNVVIKVCEFQFCNDP	18	ABCPRED
1064	1081	HVTYVPAQEKNFTTAPAI	18	ABCPRED
1010	1027	QQLIRAAEIRASANLAAT	18	ABCPRED
903	920	AYRFNGIGVTQNVLYENQ	18	ABCPRED
872	889	QYTSALLAGTITSGWTFG	18	ABCPRED
754	771	LQYGSFCTQLNRALTGIA	18	ABCPRED
720	737	ISVTTEILPVSMTKTSVD	18	ABCPRED
640	657	SNVFQTRAGCLIGAEHVN	18	ABCPRED
5	22	LVLLPLVSSQCVNLTTRT	18	ABCPRED
400	417	FVIRGDEVRQIAPGQTGK	18	ABCPRED
309	326	EKGIYQTSNFRVQPTESI	18	ABCPRED
304	321	KSFTVEKGIYQTSNFRVQ	18	ABCPRED
28	45	YTNSFTRGVYYPDKVFRS	18	ABCPRED
26	43	PAYTNSFTRGVYYPDKVF	18	ABCPRED
19	36	TTRTQLPPAYTNSFTRGV	18	ABCPRED
136	153	CNDPFLGVYYHKNNKSWM	18	ABCPRED
131	148	CEFQFCNDPFLGVYYHKN	18	ABCPRED
1149	1166	KEELDKYFKNHTSPDVDL	18	ABCPRED
967	984	SSNFGAISSVLNDILSRL	18	ABCPRED
861	878	LPPLLTDEMIAQYTSALL	18	ABCPRED
80	97	DNPVLPFNDGVYFASTEK	18	ABCPRED
751	768	NLLLQYGSFCTQLNRALT	18	ABCPRED
721	738	SVTTEILPVSMTKTSVDC	18	ABCPRED
691	708	SIIAYTMSLGAENSVAYS	18	ABCPRED
608	625	VAVLYQDVNCTEVPVAIH	18	ABCPRED
586	603	DITPCSFGGVSVITPGTN	18	ABCPRED
506	523	QPYRVVVLSFELLHAPAT	18	ABCPRED
331	348	NITNLCPFGEVFNATRFA	18	ABCPRED
1239	1256	SCCSCLKGCCSCGSCCKF	18	ABCPRED
1214	1231	WYIWLGFIAGLIAIVMVT	18	ABCPRED
1150	1167	EELDKYFKNHTSPDVDLG	18	ABCPRED
1136	1153	TVYDPLQPELDSFKEELD	18	ABCPRED
83	100	VLPFNDGVYFASTEKSNI	18	ABCPRED
660	677	YECDIPIGAGICASYQTQ	18	ABCPRED
591	608	SFGGVSVITPGTNTSNQV	18	ABCPRED
469	486	STEIYQAGSTPCNGVEGF	18	ABCPRED
453	470	YRLFRKSNLKPFERDIST	18	ABCPRED
184	201	GNFKNLREFVFKNIDGYF	18	ABCPRED
1200	1217	LQELGKYEQYIKWPWYIW	18	ABCPRED
1167	1184	GDISGINASVVNIQKEID	18	ABCPRED
111	128	DSKTQSLLIVNNATNVVI	18	ABCPRED
959	976	LNTLVKQLSSNFGAISSV	18	ABCPRED
771	788	AVEQDKNTQEVFAQVKQI	18	ABCPRED
7	24	LLPLVSSQCVNLTTRTQL	18	ABCPRED
595	612	VSVITPGTNTSNQVAVLY	18	ABCPRED
581	598	TLEILDITPCSFGGVSVI	18	ABCPRED
557	574	KKFLPFQQFGRDIADTTD	18	ABCPRED
455	472	LFRKSNLKPFERDISTEI	18	ABCPRED
352	369	AWNRKRISNCVADYSVLY	18	ABCPRED
1247	1264	CCSCGSCCKFDEDDSEPV	18	ABCPRED
1106	1123	QRNFYEPQIITTDNTFVS	18	ABCPRED
1047	1064	YHLMSFPQSAPHGVVFLH	18	ABCPRED
1015	1032	AAEIRASANLAATKMSEC	18	ABCPRED
10	27	LVSSQCVNLTTRTQLPPA	18	ABCPRED
942	959	ASALGKLQDVVNQNAQAL	18	ABCPRED
908	925	GIGVTQNVLYENQKLIAN	18	ABCPRED
891	908	GAALQIPFAMQMAYRFNG	18	ABCPRED
824	841	NKVTLADAGFIKQYGDCL	18	ABCPRED
744	761	GDSTECSNLLLQYGSFCT	18	ABCPRED
734	751	TSVDCTMYICGDSTECSN	18	ABCPRED
686	703	SVASQSIIAYTMSLGAEN	18	ABCPRED

474	491	QAGSTPCNGVEGFNCYFP	18	ABCPRED
460	477	NLKPFERDISTEIYQAGS	18	ABCPRED
46	63	SVLHSTQDLFLPFFSNVT	18	ABCPRED
379	396	CYGVSPTKLNDLCFTNVY	18	ABCPRED
355	372	RKRISNCVADYSVLYNSA	18	ABCPRED
208	225	TPINLVRDLPQGFSALEP	18	ABCPRED
179	196	LEGKQGNFKNLREFVFKN	18	ABCPRED
1229	1246	MVTIMLCCMTSCCSCLKG	18	ABCPRED
1192	1209	NLNESLIDLQELGKYEQY	18	ABCPRED
1091	1108	REGVFVSNGTHWFVTQRN	18	ABCPRED
1060	1077	VVFLHVTYVPAQEKNFTT	18	ABCPRED
911	928	VTQNVLYENQKLIANQFN	18	ABCPRED
858	875	LTVLPPLLTDEMIAQYTS	18	ABCPRED
788	805	IYKTPPIKDFGGFNFSQI	18	ABCPRED
728	745	PVSMTKTSVDCTMYICGD	18	ABCPRED
63	80	TWFHAIHVSGTNGTKRFD	18	ABCPRED
623	640	AIHADQLTPTWRVYSTGS	18	ABCPRED
456	473	FRKSNLKPFERDISTEIY	18	ABCPRED
436	453	WNSNNLDSKVGGNYNYLY	18	ABCPRED
313	330	YQTSNFRVQPTESIVRFP	18	ABCPRED
310	327	KGIYQTSNFRVQPTESIV	18	ABCPRED
1205	1222	KYEQYIKWPWYIWLGFIA	18	ABCPRED
1176	1193	VVNIQKEIDRLNEVAKNL	18	ABCPRED
116	133	SLLIVNNATNVVIKVCEF	18	ABCPRED
1147	1164	SFKEELDKYFKNHTSPDV	18	ABCPRED
1093	1110	GVFVSNGTHWFVTQRNFY	18	ABCPRED
1014	1031	RAAEIRASANLAATKMSE	18	ABCPRED
853	870	QKFNGLTVLPPLLTDEMI	18	ABCPRED
801	818	NFSQILPDPSKPSKRSFI	18	ABCPRED
749	766	CSNLLLQYGSFCTQLNRA	18	ABCPRED
747	764	TECSNLLLQYGSFCTQLN	18	ABCPRED
736	753	VDCTMYICGDSTECSNLL	18	ABCPRED
642	659	VFQTRAGCLIGAEHVNNS	18	ABCPRED
637	654	STGSNVFQTRAGCLIGAE	18	ABCPRED
627	644	DQLTPTWRVYSTGSNVFQ	18	ABCPRED
619	636	EVPVAIHADQLTPTWRVY	18	ABCPRED
611	628	LYQDVNCTEVPVAIHADQ	18	ABCPRED
56	73	LPFFSNVTWFHAIHVSGT	18	ABCPRED
499	516	PTNGVGYQPYRVVVLSFE	18	ABCPRED
491	508	PLQSYGFQPTNGVGYQPY	18	ABCPRED
472	489	IYQAGSTPCNGVEGFNCY	18	ABCPRED
40	57	DKVFRSSVLHSTQDLFLP	18	ABCPRED
395	412	VYADSFVIRGDEVRQIAP	18	ABCPRED
348	365	ASVYAWNRKRISNCVADY	18	ABCPRED
221	238	SALEPLVDLPIGINITRF	18	ABCPRED
135	152	FCNDPFLGVYYHKNNKSW	18	ABCPRED
1251	1268	GSCCKFDEDDSEPVLKGV	18	ABCPRED
1249	1266	SCGSCCKFDEDDSEPVLK	18	ABCPRED
1165	1182	DLGDISGINASVVNIQKE	18	ABCPRED
941	958	TASALGKLQDVVNQNAQA	18	ABCPRED
928	945	NSAIGKIQDSLSSTASAL	18	ABCPRED
901	918	QMAYRFNGIGVTQNVLYE	18	ABCPRED
885	902	GWTFGAGAALQIPFAMQM	18	ABCPRED
879	896	AGTITSGWTFGAGAALQI	18	ABCPRED
817	834	FIEDLLFNKVTLADAGFI	18	ABCPRED
762	779	QLNRALTGIAVEQDKNTQ	18	ABCPRED
694	711	AYTMSLGAENSVAYSNNS	18	ABCPRED
681	698	PRRARSVASQSIIAYTMS	18	ABCPRED
617	634	CTEVPVAIHADQLTPTWR	18	ABCPRED
578	595	DPQTLEILDITPCSFGGV	18	ABCPRED
520	537	APATVCGPKKSTNLVKNK	18	ABCPRED
492	509	LQSYGFQPTNGVGYQPYR	18	ABCPRED
438	455	SNNLDSKVGGNYNYLYRL	18	ABCPRED
397	414	ADSFVIRGDEVRQIAPGQ	18	ABCPRED
340	357	EVFNATRFASVYAWNRKR	18	ABCPRED
289	306	VDCALDPLSETKCTLKSF	18	ABCPRED
163	180	ANNCTFEYVSQPFLMDLE	18	ABCPRED
144	161	YYHKNNKSWMESEFRVYS	18	ABCPRED
139	156	PFLGVYYHKNNKSWMESE	18	ABCPRED
1231	1248	TIMLCCMTSCCSCLKGCC	18	ABCPRED
1222	1239	AGLIAIVMVTIMLCCMTS	18	ABCPRED
1154	1171	KYFKNHTSPDVDLGDISG	18	ABCPRED
1032	1049	CVLGQSKRVDFCGKGYHL	18	ABCPRED

992	1009	QIDRLITGRLQSLQTYVT	18	ABCPRED
923	940	IANQFNSAIGKIQDSLSS	18	ABCPRED
875	892	SALLAGTITSGWTFGAGA	18	ABCPRED
816	833	SFIEDLLFNKVTLADAGF	18	ABCPRED
814	831	KRSFIEDLLFNKVTLADA	18	ABCPRED
799	816	GFNFSQILPDPSKPSKRS	18	ABCPRED
765	782	RALTGIAVEQDKNTQEVF	18	ABCPRED
723	740	TTEILPVSMTKTSVDCTM	18	ABCPRED
714	731	IPTNFTISVTTEILPVSM	18	ABCPRED
711	728	SIAIPTNFTISVTTEILP	18	ABCPRED
675	692	QTQTNSPRRARSVASQSI	18	ABCPRED
668	685	AGICASYQTQTNSPRRAR	18	ABCPRED
564	581	QFGRDIADTTDAVRDPQT	18	ABCPRED
562	579	FQQFGRDIADTTDAVRDP	18	ABCPRED
349	366	SVYAWNRKRISNCVADYS	18	ABCPRED
245	262	HRSYLTPGDSSSGWTAGA	18	ABCPRED
18	35	LTTRTQLPPAYTNSFTRG	18	ABCPRED
165	182	NCTFEYVSQPFLMDLEGK	18	ABCPRED
1132	1149	IVNNTVYDPLQPELDSFK	18	ABCPRED
1011	1028	QLIRAAEIRASANLAATK	18	ABCPRED
940	957	STASALGKLQDVVNQNAQ	18	ABCPRED
864	881	LLTDEMIAQYTSALLAGT	18	ABCPRED
836	853	QYGDCLGDIAARDLICAQ	18	ABCPRED
832	849	GFIKQYGDCLGDIAARDL	18	ABCPRED
825	842	KVTLADAGFIKQYGDCLG	18	ABCPRED
805	822	ILPDPSKPSKRSFIEDLL	18	ABCPRED
778	795	TQEVFAQVKQIYKTPPIK	18	ABCPRED
772	789	VEQDKNTQEVFAQVKQIY	18	ABCPRED
739	756	TMYICGDSTECSNLLLQY	18	ABCPRED
700	717	GAENSVAYSNNSIAIPTN	18	ABCPRED
517	534	LLHAPATVCGPKKSTNLV	18	ABCPRED
500	517	TNGVGYQPYRVVVLSFEL	18	ABCPRED
486	503	FNCYFPLQSYGFQPTNGV	18	ABCPRED
440	457	NLDSKVGGNYNYLYRLFR	18	ABCPRED
424	441	KLPDDFTGCVIAWNSNNL	18	ABCPRED
415	432	TGKIADYNYKLPDDFTGC	18	ABCPRED
371	388	SASFSTFKCYGVSPTKLN	18	ABCPRED
362	379	VADYSVLYNSASFSTFKC	18	ABCPRED
267	284	VGYLQPRTFLLKYNENGT	18	ABCPRED
1245	1262	KGCCSCGSCCKFDEDDSE	18	ABCPRED
1244	1261	LKGCCSCGSCCKFDEDDS	18	ABCPRED
1153	1170	DKYFKNHTSPDVDLGDIS	18	ABCPRED
1097	1114	SNGTHWFVTQRNFYEPQI	18	ABCPRED
1088	1105	HFPREGVFVSNGTHWFVT	18	ABCPRED
991	1008	VQIDRLITGRLQSLQTYV	18	ABCPRED
921	938	KLIANQFNSAIGKIQDSL	18	ABCPRED
84	101	LPFNDGVYFASTEKSNII	18	ABCPRED
826	843	VTLADAGFIKQYGDCLGD	18	ABCPRED
77	94	KRFDNPVLPFNDGVYFAS	18	ABCPRED
764	781	NRALTGIAVEQDKNTQEV	18	ABCPRED
647	664	AGCLIGAEHVNNSYECDI	18	ABCPRED
638	655	TGSNVFQTRAGCLIGAEH	18	ABCPRED
592	609	FGGVSVITPGTNTSNQVA	18	ABCPRED
587	604	ITPCSFGGVSVITPGTNT	18	ABCPRED
377	394	FKCYGVSPTKLNDLCFTN	18	ABCPRED
344	361	ATRFASVYAWNRKRISNC	18	ABCPRED
326	343	IVRFPNITNLCPFGEVFN	18	ABCPRED
321	338	QPTESIVRFPNITNLCPF	18	ABCPRED
296	313	LSETKCTLKSFTVEKGIY	18	ABCPRED
276	293	LLKYNENGTITDAVDCAL	18	ABCPRED
157	174	FRVYSSANNCTFEYVSQP	18	ABCPRED
1198	1215	IDLQELGKYEQYIKWPWY	18	ABCPRED
118	135	LIVNNATNVVIKVCEFQF	18	ABCPRED
1134	1151	NNTVYDPLQPELDSFKEE	18	ABCPRED
101	118	IRGWIFGTTLDSKTQSLL	18	ABCPRED
95	112	TEKSNIIRGWIFGTTLDS	18	ABCPRED
902	919	MAYRFNGIGVTQNVLYEN	18	ABCPRED
856	873	NGLTVLPPLLTDEMIAQY	18	ABCPRED
843	860	DIAARDLICAQKFNGLTV	18	ABCPRED
81	98	NPVLPFNDGVYFASTEKS	18	ABCPRED
682	699	RRARSVASQSIIAYTMSL	18	ABCPRED
656	673	VNNSYECDIPIGAGICAS	18	ABCPRED
650	667	LIGAEHVNNSYECDIPIG	18	ABCPRED

393	410	TNVYADSFVIRGDEVRQI	18	ABCPRED
291	308	CALDPLSETKCTLKSFTV	18	ABCPRED
239	256	QTLLALHRSYLTPGDSSS	18	ABCPRED
226	243	LVDLPIGINITRFQTLLA	18	ABCPRED
17	34	NLTTRTQLPPAYTNSFTR	18	ABCPRED
162	179	SANNCTFEYVSQPFLMDL	18	ABCPRED
1220	1237	FIAGLIAIVMVTIMLCCM	18	ABCPRED
1193	1210	LNESLIDLQELGKYEQYI	18	ABCPRED
1142	1159	QPELDSFKEELDKYFKNH	18	ABCPRED
956	973	AQALNTLVKQLSSNFGAI	18	ABCPRED
859	876	TVLPPLLTDEMIAQYTSA	18	ABCPRED
800	817	FNFSQILPDPSKPSKRSF	18	ABCPRED
760	777	CTQLNRALTGIAVEQDKN	18	ABCPRED
659	676	SYECDIPIGAGICASYQT	18	ABCPRED
649	666	CLIGAEHVNNSYECDIPI	18	ABCPRED
606	623	NQVAVLYQDVNCTEVPVA	18	ABCPRED
529	546	KSTNLVKNKCVNFNFNGL	18	ABCPRED
524	541	VCGPKKSTNLVKNKCVNF	18	ABCPRED
484	501	EGFNCYFPLQSYGFQPTN	18	ABCPRED
457	474	RKSNLKPFERDISTEIYQ	18	ABCPRED
443	460	SKVGGNYNYLYRLFRKSN	18	ABCPRED
386	403	KLNDLCFTNVYADSFVIR	18	ABCPRED
300	317	KCTLKSFTVEKGIYQTSN	18	ABCPRED
27	44	AYTNSFTRGVYYPDKVFR	18	ABCPRED
262	279	AAAYYVGYLQPRTFLLKY	18	ABCPRED
234	251	NITRFQTLLALHRSYLTP	18	ABCPRED
150	167	KSWMESEFRVYSSANNCT	18	ABCPRED
990	1007	EVQIDRLITGRLQSLQTY	18	ABCPRED
91	108	YFASTEKSNIIRGWIFGT	18	ABCPRED
865	882	LTDEMIAQYTSALLAGTI	18	ABCPRED
863	880	PLLTDEMIAQYTSALLAG	18	ABCPRED
846	863	ARDLICAQKFNGLTVLPP	18	ABCPRED
827	844	TLADAGFIKQYGDCLGDI	18	ABCPRED
729	746	VSMTKTSVDCTMYICGDS	18	ABCPRED
706	723	AYSNNSIAIPTNFTISVT	18	ABCPRED
703	720	NSVAYSNNSIAIPTNFTI	18	ABCPRED
677	694	QTNSPRRARSVASQSIIA	18	ABCPRED
546	563	LTGTGVLTESNKKFLPFQ	18	ABCPRED
521	538	PATVCGPKKSTNLVKNKC	18	ABCPRED
504	521	GYQPYRVVVLSFELLHAP	18	ABCPRED
441	458	LDSKVGGNYNYLYRLFRK	18	ABCPRED
434	451	IAWNSNNLDSKVGGNYNY	18	ABCPRED
354	371	NRKRISNCVADYSVLYNS	18	ABCPRED
350	367	VYAWNRKRISNCVADYSV	18	ABCPRED
302	319	TLKSFTVEKGIYQTSNFR	18	ABCPRED
279	296	YNENGTITDAVDCALDPL	18	ABCPRED
21	38	RTQLPPAYTNSFTRGVYY	18	ABCPRED
1141	1158	LQPELDSFKEELDKYFKN	18	ABCPRED
1069	1086	PAQEKNFTTAPAICHDGK	18	ABCPRED
1020	1037	ASANLAATKMSECVLGQS	18	ABCPRED
977	994	LNDILSRLDKVEAEVQID	18	ABCPRED
976	993	VLNDILSRLDKVEAEVQI	18	ABCPRED
972	989	AISSVLNDILSRLDKVEA	18	ABCPRED
897	914	PFAMQMAYRFNGIGVTQN	18	ABCPRED
840	857	CLGDIAARDLICAQKFNG	18	ABCPRED
792	809	PPIKDFGGFNFSQILPDP	18	ABCPRED
752	769	LLLQYGSFCTQLNRALTG	18	ABCPRED
75	92	GTKRFDNPVLPFNDGVYF	18	ABCPRED
719	736	TISVTTEILPVSMTKTSV	18	ABCPRED
717	734	NFTISVTTEILPVSMTKT	18	ABCPRED
654	671	EHVNNSYECDIPIGAGIC	18	ABCPRED
604	621	TSNQVAVLYQDVNCTEVP	18	ABCPRED
549	566	TGVLTESNKKFLPFQQFG	18	ABCPRED
413	430	GQTGKIADYNYKLPDDFT	18	ABCPRED
391	408	CFTNVYADSFVIRGDEVR	18	ABCPRED
375	392	STFKCYGVSPTKLNDLCF	18	ABCPRED
338	355	FGEVFNATRFASVYAWNR	18	ABCPRED
337	354	PFGEVFNATRFASVYAWN	18	ABCPRED
297	314	SETKCTLKSFTVEKGIYQ	18	ABCPRED
282	299	NGTITDAVDCALDPLSET	18	ABCPRED
206	223	KHTPINLVRDLPQGFSAL	18	ABCPRED
177	194	MDLEGKQGNFKNLREFVF	18	ABCPRED
175	192	FLMDLEGKQGNFKNLREF	18	ABCPRED

148	165	NNKSWMESEFRVYSSANN	18	ABCPRED
13	30	SQCVNLTTRTQLPPAYTN	18	ABCPRED
1243	1260	CLKGCCSCGSCCKFDEDD	18	ABCPRED
1216	1233	IWLGFIAGLIAIVMVTIM	18	ABCPRED
1145	1162	LDSFKEELDKYFKNHTSP	18	ABCPRED
1109	1126	FYEPQIITTDNTFVSGNC	18	ABCPRED
1107	1124	RNFYEPQIITTDNTFVSG	18	ABCPRED
1104	1121	VTQRNFYEPQIITTDNTF	18	ABCPRED
1044	1061	GKGYHLMSFPQSAPHGVV	18	ABCPRED
939	956	SSTASALGKLQDVVNQNA	18	ABCPRED
894	911	LQIPFAMQMAYRFNGIGV	18	ABCPRED
8	25	LPLVSSQCVNLTTRTQLP	18	ABCPRED
776	793	KNTQEVFAQVKQIYKTPP	18	ABCPRED
73	90	TNGTKRFDNPVLPFNDGV	18	ABCPRED
715	732	PTNFTISVTTEILPVSMT	18	ABCPRED
67	84	AIHVSGTNGTKRFDNPVL	18	ABCPRED
657	674	NNSYECDIPIGAGICASY	18	ABCPRED
597	614	VITPGTNTSNQVAVLYQD	18	ABCPRED
585	602	LDITPCSFGGVSVITPGT	18	ABCPRED
580	597	QTLEILDITPCSFGGVSV	18	ABCPRED
418	435	IADYNYKLPDDFTGCVIA	18	ABCPRED
394	411	NVYADSFVIRGDEVRQIA	18	ABCPRED
390	407	LCFTNVYADSFVIRGDEV	18	ABCPRED
374	391	FSTFKCYGVSPTKLNDLC	18	ABCPRED
315	332	TSNFRVQPTESIVRFPNI	18	ABCPRED
290	307	DCALDPLSETKCTLKSFT	18	ABCPRED
246	263	RSYLTPGDSSSGWTAGAA	18	ABCPRED
209	226	PINLVRDLPQGFSALEPL	18	ABCPRED
132	149	EFQFCNDPFLGVYYHKNN	18	ABCPRED
1252	1269	SCCKFDEDDSEPVLKGVK	18	ABCPRED
1162	1179	PDVDLGDISGINASVVNI	18	ABCPRED
1156	1173	FKNHTSPDVDLGDISGIN	18	ABCPRED
1140	1157	PLQPELDSFKEELDKYFK	18	ABCPRED
1113	1130	QIITTDNTFVSGNCDVVI	18	ABCPRED
1079	1096	PAICHDGKAHFPREGVFV	18	ABCPRED
1054	1071	QSAPHGVVFLHVTYVPAQ	18	ABCPRED
1033	1050	VLGQSKRVDFCGKGYHLM	18	ABCPRED
929	946	SAIGKIQDSLSSTASALG	18	ABCPRED
907	924	NGIGVTQNVLYENQKLIA	18	ABCPRED
886	903	WTFGAGAALQIPFAMQMA	18	ABCPRED
86	103	FNDGVYFASTEKSNIIRG	18	ABCPRED
851	868	CAQKFNGLTVLPPLLTDE	18	ABCPRED
85	102	PFNDGVYFASTEKSNIIR	18	ABCPRED
830	847	DAGFIKQYGDCLGDIAAR	18	ABCPRED
687	704	VASQSIIAYTMSLGAENS	18	ABCPRED
685	702	RSVASQSIIAYTMSLGAE	18	ABCPRED
628	645	QLTPTWRVYSTGSNVFQT	18	ABCPRED
624	641	IHADQLTPTWRVYSTGSN	18	ABCPRED
553	570	TESNKKFLPFQQFGRDIA	18	ABCPRED
535	552	KNKCVNFNFNGLTGTGVL	18	ABCPRED
463	480	PFERDISTEIYQAGSTPC	18	ABCPRED
370	387	NSASFSTFKCYGVSPTKL	18	ABCPRED
341	358	VFNATRFASVYAWNRKRI	18	ABCPRED
292	309	ALDPLSETKCTLKSFTVE	18	ABCPRED
260	277	AGAAAYYVGYLQPRTFLL	18	ABCPRED
1208	1225	QYIKWPWYIWLGFIAGLI	18	ABCPRED
1007	1024	YVTQQLIRAAEIRASANL	18	ABCPRED
847	864	RDLICAQKFNGLTVLPPL	18	ABCPRED
794	811	IKDFGGFNFSQILPDPSK	18	ABCPRED
79	96	FDNPVLPFNDGVYFASTE	18	ABCPRED
781	798	VFAQVKQIYKTPPIKDFG	18	ABCPRED
76	93	TKRFDNPVLPFNDGVYFA	18	ABCPRED
705	722	VAYSNNSIAIPTNFTISV	18	ABCPRED
688	705	ASQSIIAYTMSLGAENSV	18	ABCPRED
645	662	TRAGCLIGAEHVNNSYEC	18	ABCPRED
558	575	KFLPFQQFGRDIADTTDA	18	ABCPRED
552	569	LTESNKKFLPFQQFGRDI	18	ABCPRED
490	507	FPLQSYGFQPTNGVGYQP	18	ABCPRED
429	446	FTGCVIAWNSNNLDSKVG	18	ABCPRED
359	376	SNCVADYSVLYNSASFST	18	ABCPRED
269	286	YLQPRTFLLKYNENGTIT	18	ABCPRED
251	268	PGDSSSGWTAGAAAYYVG	18	ABCPRED
250	267	TPGDSSSGWTAGAAAYYV	18	ABCPRED

22	39	TQLPPAYTNSFTRGVYYP	18	ABCPRED
1194	1211	NESLIDLQELGKYEQYIK	18	ABCPRED
1190	1207	AKNLNESLIDLQELGKYE	18	ABCPRED
113	130	KTQSLLIVNNATNVVIKV	18	ABCPRED
1121	1138	FVSGNCDVVIGIVNNTVY	18	ABCPRED
1108	1125	NFYEPQIITTDNTFVSGN	18	ABCPRED
1076	1093	TTAPAICHDGKAHFPREG	18	ABCPRED
1040	1057	VDFCGKGYHLMSFPQSAP	18	ABCPRED
1030	1047	SECVLGQSKRVDFCGKGY	18	ABCPRED
1016	1033	AEIRASANLAATKMSECV	18	ABCPRED
936	953	DSLSSTASALGKLQDVVN	18	ABCPRED
857	874	GLTVLPPLLTDEMIAQYT	18	ABCPRED
811	828	KPSKRSFIEDLLFNKVTL	18	ABCPRED
775	792	DKNTQEVFAQVKQIYKTP	18	ABCPRED
741	758	YICGDSTECSNLLLQYGS	18	ABCPRED
698	715	SLGAENSVAYSNNSIAIP	18	ABCPRED
697	714	MSLGAENSVAYSNNSIAI	18	ABCPRED
547	564	TGTGVLTESNKKFLPFQQ	18	ABCPRED
538	555	CVNFNFNGLTGTGVLTES	18	ABCPRED
487	504	NCYFPLQSYGFQPTNGVG	18	ABCPRED
335	352	LCPFGEVFNATRFASVYA	18	ABCPRED
330	347	PNITNLCPFGEVFNATRF	18	ABCPRED
327	344	VRFPNITNLCPFGEVFNA	18	ABCPRED
278	295	KYNENGTITDAVDCALDP	18	ABCPRED
277	294	LKYNENGTITDAVDCALD	18	ABCPRED
243	260	ALHRSYLTPGDSSSGWTA	18	ABCPRED
231	248	IGINITRFQTLLALHRSY	18	ABCPRED
223	240	LEPLVDLPIGINITRFQT	18	ABCPRED
197	214	IDGYFKIYSKHTPINLVR	18	ABCPRED
129	146	KVCEFQFCNDPFLGVYYH	18	ABCPRED
1212	1229	WPWYIWLGFIAGLIAIVM	18	ABCPRED
121	138	NNATNVVIKVCEFQFCND	18	ABCPRED
1168	1185	DISGINASVVNIQKEIDR	18	ABCPRED
1122	1139	VSGNCDVVIGIVNNTVYD	18	ABCPRED
1118	1135	DNTFVSGNCDVVIGIVNN	18	ABCPRED
1051	1068	SFPQSAPHGVVFLHVTYV	18	ABCPRED
1035	1052	GQSKRVDFCGKGYHLMSF	18	ABCPRED
100	117	IIRGWIFGTTLDSKTQSL	18	ABCPRED
98	115	SNIIRGWIFGTTLDSKTQ	18	ABCPRED
946	963	GKLQDVVNQNAQALNTLV	18	ABCPRED
844	861	IAARDLICAQKFNGLTVL	18	ABCPRED
673	690	SYQTQTNSPRRARSVASQ	18	ABCPRED
651	668	IGAEHVNNSYECDIPIGA	18	ABCPRED
636	653	YSTGSNVFQTRAGCLIGA	18	ABCPRED
544	561	NGLTGTGVLTESNKKFLP	18	ABCPRED
508	525	YRVVVLSFELLHAPATVC	18	ABCPRED
446	463	GGNYNYLYRLFRKSNLKP	18	ABCPRED
392	409	FTNVYADSFVIRGDEVRQ	18	ABCPRED
311	328	GIYQTSNFRVQPTESIVR	18	ABCPRED
257	274	GWTAGAAAYYVGYLQPRT	18	ABCPRED
176	193	LMDLEGKQGNFKNLREFV	18	ABCPRED
172	189	SQPFLMDLEGKQGNFKNL	18	ABCPRED
138	155	DPFLGVYYHKNNKSWMES	18	ABCPRED
1248	1265	CSCGSCCKFDEDDSEPVL	18	ABCPRED
1209	1226	YIKWPWYIWLGFIAGLIA	18	ABCPRED
1023	1040	NLAATKMSECVLGQSKRV	18	ABCPRED
1012	1029	LIRAAEIRASANLAATKM	18	ABCPRED
974	991	SSVLNDILSRLDKVEAEV	18	ABCPRED
958	975	ALNTLVKQLSSNFGAISS	18	ABCPRED
882	899	ITSGWTFGAGAALQIPFA	18	ABCPRED
841	858	LGDIAARDLICAQKFNGL	18	ABCPRED
813	830	SKRSFIEDLLFNKVTLAD	18	ABCPRED
785	802	VKQIYKTPPIKDFGGFNF	18	ABCPRED
777	794	NTQEVFAQVKQIYKTPPI	18	ABCPRED
766	783	ALTGIAVEQDKNTQEVFA	18	ABCPRED
71	88	SGTNGTKRFDNPVLPFND	18	ABCPRED
582	599	LEILDITPCSFGGVSVIT	18	ABCPRED
542	559	NFNGLTGTGVLTESNKKF	18	ABCPRED
387	404	LNDLCFTNVYADSFVIRG	18	ABCPRED
364	381	DYSVLYNSASFSTFKCYG	18	ABCPRED
333	350	TNLCPFGEVFNATRFASV	18	ABCPRED
236	253	TRFQTLLALHRSYLTPGD	18	ABCPRED
125	142	NVVIKVCEFQFCNDPFLG	18	ABCPRED

1238	1255	TSCCSCLKGCCSCGSCCK	18	ABCPRED
1223	1240	GLIAIVMVTIMLCCMTSC	18	ABCPRED
1207	1224	EQYIKWPWYIWLGFIAGL	18	ABCPRED
11	28	VSSQCVNLTTRTQLPPAY	18	ABCPRED
1019	1036	RASANLAATKMSECVLGQ	18	ABCPRED
943	960	SALGKLQDVVNQNAQALN	18	ABCPRED
876	893	ALLAGTITSGWTFGAGAA	18	ABCPRED
822	839	LFNKVTLADAGFIKQYGD	18	ABCPRED
737	754	DCTMYICGDSTECSNLLL	18	ABCPRED
730	747	SMTKTSVDCTMYICGDST	18	ABCPRED
667	684	GAGICASYQTQTNSPRRA	18	ABCPRED
603	620	NTSNQVAVLYQDVNCTEV	18	ABCPRED
588	605	TPCSFGGVSVITPGTNTS	18	ABCPRED
541	558	FNFNGLTGTGVLTESNKK	18	ABCPRED
514	531	SFELLHAPATVCGPKKST	18	ABCPRED
493	510	QSYGFQPTNGVGYQPYRV	18	ABCPRED
442	459	DSKVGGNYNYLYRLFRKS	18	ABCPRED
43	60	FRSSVLHSTQDLFLPFFS	18	ABCPRED
358	375	ISNCVADYSVLYNSASFS	18	ABCPRED
228	245	DLPIGINITRFQTLLALH	18	ABCPRED
1235	1252	CCMTSCCSCLKGCCSCGS	18	ABCPRED
1187	1204	NEVAKNLNESLIDLQELG	18	ABCPRED
1073	1090	KNFTTAPAICHDGKAHFP	18	ABCPRED
1071	1088	QEKNFTTAPAICHDGKAH	18	ABCPRED
1024	1041	LAATKMSECVLGQSKRVD	18	ABCPRED
826	845	VTLADAGFIKQYGDCLGDIA	20	ABCPRED
651	670	IGAEHVNNSYECDIPIGAGI	20	ABCPRED
393	412	TNVYADSFVIRGDEVRQIAP	20	ABCPRED
143	162	VYYHKNNKSWMESEFRVYSS	20	ABCPRED
68	87	IHVSGTNGTKRFDNPVLPFN	20	ABCPRED
614	633	DVNCTEVPVAIHADQLTPTW	20	ABCPRED
247	266	SYLTPGDSSSGWTAGAAAYY	20	ABCPRED
206	225	KHTPINLVRDLPQGFSALEP	20	ABCPRED
126	145	VVIKVCEFQFCNDPFLGVYY	20	ABCPRED
994	1013	DRLITGRLQSLQTYVTQQLI	20	ABCPRED
337	356	PFGEVFNATRFASVYAWNRK	20	ABCPRED
1008	1027	VTQQLIRAAEIRASANLAAT	20	ABCPRED
389	408	DLCFTNVYADSFVIRGDEVR	20	ABCPRED
147	166	KNNKSWMESEFRVYSSANNC	20	ABCPRED
1118	1137	DNTFVSGNCDVVIGIVNNTV	20	ABCPRED
108	127	TTLDSKTQSLLIVNNATNVV	20	ABCPRED
992	1011	QIDRLITGRLQSLQTYVTQQ	20	ABCPRED
505	524	YQPYRVVVLSFELLHAPATV	20	ABCPRED
44	63	RSSVLHSTQDLFLPFFSNVT	20	ABCPRED
356	375	KRISNCVADYSVLYNSASFS	20	ABCPRED
1216	1235	IWLGFIAGLIAIVMVTIMLC	20	ABCPRED
103	122	GWIFGTTLDSKTQSLLIVNN	20	ABCPRED
1009	1028	TQQLIRAAEIRASANLAATK	20	ABCPRED
914	933	NVLYENQKLIANQFNSAIGK	20	ABCPRED
34	53	RGVYYPDKVFRSSVLHSTQD	20	ABCPRED
107	126	GTTLDSKTQSLLIVNNATNV	20	ABCPRED
973	992	ISSVLNDILSRLDKVEAEVQ	20	ABCPRED
879	898	AGTITSGWTFGAGAALQIPF	20	ABCPRED
720	739	ISVTTEILPVSMTKTSVDCT	20	ABCPRED
619	638	EVPVAIHADQLTPTWRVYST	20	ABCPRED
589	608	PCSFGGVSVITPGTNTSNQV	20	ABCPRED
988	1007	EAEVQIDRLITGRLQSLQTY	20	ABCPRED
774	793	QDKNTQEVFAQVKQIYKTPP	20	ABCPRED
566	585	GRDIADTTDAVRDPQTLEIL	20	ABCPRED
563	582	QQFGRDIADTTDAVRDPQTL	20	ABCPRED
535	554	KNKCVNFNFNGLTGTGVLTE	20	ABCPRED
459	478	SNLKPFERDISTEIYQAGST	20	ABCPRED
290	309	DCALDPLSETKCTLKSFTVE	20	ABCPRED
248	267	YLTPGDSSSGWTAGAAAYYV	20	ABCPRED
178	197	DLEGKQGNFKNLREFVFKNI	20	ABCPRED
171	190	VSQPFLMDLEGKQGNFKNLR	20	ABCPRED
166	185	CTFEYVSQPFLMDLEGKQGN	20	ABCPRED
160	179	YSSANNCTFEYVSQPFLMDL	20	ABCPRED
909	928	IGVTQNVLYENQKLIANQFN	20	ABCPRED
832	851	GFIKQYGDCLGDIAARDLIC	20	ABCPRED
621	640	PVAIHADQLTPTWRVYSTGS	20	ABCPRED
431	450	GCVIAWNSNNLDSKVGGNYN	20	ABCPRED
985	1004	DKVEAEVQIDRLITGRLQSL	20	ABCPRED

906	925	FNGIGVTQNVLYENQKLIAN	20	ABCPRED
748	767	ECSNLLLQYGSFCTQLNRAL	20	ABCPRED
613	632	QDVNCTEVPVAIHADQLTPT	20	ABCPRED
588	607	TPCSFGGVSVITPGTNTSNQ	20	ABCPRED
388	407	NDLCFTNVYADSFVIRGDEV	20	ABCPRED
381	400	GVSPTKLNDLCFTNVYADSF	20	ABCPRED
249	268	LTPGDSSSGWTAGAAAYYVG	20	ABCPRED
167	186	TFEYVSQPFLMDLEGKQGNF	20	ABCPRED
986	1005	KVEAEVQIDRLITGRLQSLQ	20	ABCPRED
974	993	SSVLNDILSRLDKVEAEVQI	20	ABCPRED
969	988	NFGAISSVLNDILSRLDKVE	20	ABCPRED
962	981	LVKQLSSNFGAISSVLNDIL	20	ABCPRED
89	108	GVYFASTEKSNIIRGWIFGT	20	ABCPRED
595	614	VSVITPGTNTSNQVAVLYQD	20	ABCPRED
1043	1062	CGKGYHLMSFPQSAPHGVVF	20	ABCPRED
92	111	FASTEKSNIIRGWIFGTTLD	20	ABCPRED
880	899	GTITSGWTFGAGAALQIPFA	20	ABCPRED
85	104	PFNDGVYFASTEKSNIIRGW	20	ABCPRED
821	840	LLFNKVTLADAGFIKQYGDC	20	ABCPRED
688	707	ASQSIIAYTMSLGAENSVAY	20	ABCPRED
679	698	NSPRRARSVASQSIIAYTMS	20	ABCPRED
196	215	NIDGYFKIYSKHTPINLVRD	20	ABCPRED
993	1012	IDRLITGRLQSLQTYVTQQL	20	ABCPRED
93	112	ASTEKSNIIRGWIFGTTLDS	20	ABCPRED
908	927	GIGVTQNVLYENQKLIANQF	20	ABCPRED
842	861	GDIAARDLICAQKFNGLTVL	20	ABCPRED
808	827	DPSKPSKRSFIEDLLFNKVT	20	ABCPRED
735	754	SVDCTMYICGDSTECSNLLL	20	ABCPRED
567	586	RDIADTTDAVRDPQTLEILD	20	ABCPRED
499	518	PTNGVGYQPYRVVVLSFELL	20	ABCPRED
436	455	WNSNNLDSKVGGNYNYLYRL	20	ABCPRED
951	970	VVNQNAQALNTLVKQLSSNF	20	ABCPRED
915	934	VLYENQKLIANQFNSAIGKI	20	ABCPRED
835	854	KQYGDCLGDIAARDLICAQK	20	ABCPRED
82	101	PVLPFNDGVYFASTEKSNII	20	ABCPRED
81	100	NPVLPFNDGVYFASTEKSNI	20	ABCPRED
682	701	RRARSVASQSIIAYTMSLGA	20	ABCPRED
615	634	VNCTEVPVAIHADQLTPTWR	20	ABCPRED
583	602	EILDITPCSFGGVSVITPGT	20	ABCPRED
517	536	LLHAPATVCGPKKSTNLVKN	20	ABCPRED
42	61	VFRSSVLHSTQDLFLPFFSN	20	ABCPRED
395	414	VYADSFVIRGDEVRQIAPGQ	20	ABCPRED
276	295	LLKYNENGTITDAVDCALDP	20	ABCPRED
1223	1242	GLIAIVMVTIMLCCMTSCCS	20	ABCPRED
1170	1189	SGINASVVNIQKEIDRLNEV	20	ABCPRED
110	129	LDSKTQSLLIVNNATNVVIK	20	ABCPRED
617	636	CTEVPVAIHADQLTPTWRVY	20	ABCPRED
577	596	RDPQTLEILDITPCSFGGVS	20	ABCPRED
536	555	NKCVNFNFNGLTGTGVLTES	20	ABCPRED
515	534	FELLHAPATVCGPKKSTNLV	20	ABCPRED
411	430	APGQTGKIADYNYKLPDDFT	20	ABCPRED
408	427	RQIAPGQTGKIADYNYKLPD	20	ABCPRED
352	371	AWNRKRISNCVADYSVLYNS	20	ABCPRED
272	291	PRTFLLKYNENGTITDAVDC	20	ABCPRED
252	271	GDSSSGWTAGAAAYYVGYLQ	20	ABCPRED
235	254	ITRFQTLLALHRSYLTPGDS	20	ABCPRED
182	201	KQGNFKNLREFVFKNIDGYF	20	ABCPRED
13	32	SQCVNLTTRTQLPPAYTNSF	20	ABCPRED
84	103	LPFNDGVYFASTEKSNIIRG	20	ABCPRED
837	856	YGDCLGDIAARDLICAQKFN	20	ABCPRED
810	829	SKPSKRSFIEDLLFNKVTLA	20	ABCPRED
778	797	TQEVFAQVKQIYKTPPIKDF	20	ABCPRED
736	755	VDCTMYICGDSTECSNLLLQ	20	ABCPRED
598	617	ITPGTNTSNQVAVLYQDVNC	20	ABCPRED
546	565	LTGTGVLTESNKKFLPFQQF	20	ABCPRED
368	387	LYNSASFSTFKCYGVSPTKL	20	ABCPRED
304	323	KSFTVEKGIYQTSNFRVQPT	20	ABCPRED
251	270	PGDSSSGWTAGAAAYYVGYL	20	ABCPRED
179	198	LEGKQGNFKNLREFVFKNID	20	ABCPRED
118	137	LIVNNATNVVIKVCEFQFCN	20	ABCPRED
1128	1147	VVIGIVNNTVYDPLQPELDS	20	ABCPRED
997	1016	ITGRLQSLQTYVTQQLIRAA	20	ABCPRED
975	994	SVLNDILSRLDKVEAEVQID	20	ABCPRED

910	929	GVTQNVLYENQKLIANQFNS	20	ABCPRED
883	902	TSGWTFGAGAALQIPFAMQM	20	ABCPRED
812	831	PSKRSFIEDLLFNKVTLADA	20	ABCPRED
806	825	LPDPSKPSKRSFIEDLLFNK	20	ABCPRED
79	98	FDNPVLPFNDGVYFASTEKS	20	ABCPRED
78	97	RFDNPVLPFNDGVYFASTEK	20	ABCPRED
779	798	QEVFAQVKQIYKTPPIKDFG	20	ABCPRED
760	779	CTQLNRALTGIAVEQDKNTQ	20	ABCPRED
600	619	PGTNTSNQVAVLYQDVNCTE	20	ABCPRED
590	609	CSFGGVSVITPGTNTSNQVA	20	ABCPRED
540	559	NFNFNGLTGTGVLTESNKKF	20	ABCPRED
489	508	YFPLQSYGFQPTNGVGYQPY	20	ABCPRED
309	328	EKGIYQTSNFRVQPTESIVR	20	ABCPRED
246	265	RSYLTPGDSSSGWTAGAAAY	20	ABCPRED
220	239	FSALEPLVDLPIGINITRFQ	20	ABCPRED
163	182	ANNCTFEYVSQPFLMDLEGK	20	ABCPRED
150	169	KSWMESEFRVYSSANNCTFE	20	ABCPRED
127	146	VIKVCEFQFCNDPFLGVYYH	20	ABCPRED
1248	1267	CSCGSCCKFDEDDSEPVLKG	20	ABCPRED
1214	1233	WYIWLGFIAGLIAIVMVTIM	20	ABCPRED
990	1009	EVQIDRLITGRLQSLQTYVT	20	ABCPRED
827	846	TLADAGFIKQYGDCLGDIAA	20	ABCPRED
809	828	PSKPSKRSFIEDLLFNKVTL	20	ABCPRED
773	792	EQDKNTQEVFAQVKQIYKTP	20	ABCPRED
725	744	EILPVSMTKTSVDCTMYICG	20	ABCPRED
648	667	GCLIGAEHVNNSYECDIPIG	20	ABCPRED
639	658	GSNVFQTRAGCLIGAEHVNN	20	ABCPRED
594	613	GVSVITPGTNTSNQVAVLYQ	20	ABCPRED
565	584	FGRDIADTTDAVRDPQTLEI	20	ABCPRED
523	542	TVCGPKKSTNLVKNKCVNFN	20	ABCPRED
509	528	RVVVLSFELLHAPATVCGPK	20	ABCPRED
384	403	PTKLNDLCFTNVYADSFVIR	20	ABCPRED
274	293	TFLLKYNENGTITDAVDCAL	20	ABCPRED
1230	1249	VTIMLCCMTSCCSCLKGCCS	20	ABCPRED
1129	1148	VIGIVNNTVYDPLQPELDSF	20	ABCPRED
1089	1108	FPREGVFVSNGTHWFVTQRN	20	ABCPRED
981	1000	LSRLDKVEAEVQIDRLITGR	20	ABCPRED
834	853	IKQYGDCLGDIAARDLICAQ	20	ABCPRED
807	826	PDPSKPSKRSFIEDLLFNKV	20	ABCPRED
80	99	DNPVLPFNDGVYFASTEKSN	20	ABCPRED
776	795	KNTQEVFAQVKQIYKTPPIK	20	ABCPRED
630	649	TPTWRVYSTGSNVFQTRAGC	20	ABCPRED
63	82	TWFHAIHVSGTNGTKRFDNP	20	ABCPRED
596	615	SVITPGTNTSNQVAVLYQDV	20	ABCPRED
507	526	PYRVVVLSFELLHAPATVCG	20	ABCPRED
488	507	CYFPLQSYGFQPTNGVGYQP	20	ABCPRED
454	473	RLFRKSNLKPFERDISTEIY	20	ABCPRED
416	435	GKIADYNYKLPDDFTGCVIA	20	ABCPRED
287	306	DAVDCALDPLSETKCTLKSF	20	ABCPRED
283	302	GTITDAVDCALDPLSETKCT	20	ABCPRED
193	212	VFKNIDGYFKIYSKHTPINL	20	ABCPRED
1168	1187	DISGINASVVNIQKEIDRLN	20	ABCPRED
1149	1168	KEELDKYFKNHTSPDVDLGD	20	ABCPRED
943	962	SALGKLQDVVNQNAQALNTL	20	ABCPRED
822	841	LFNKVTLADAGFIKQYGDCL	20	ABCPRED
815	834	RSFIEDLLFNKVTLADAGFI	20	ABCPRED
787	806	QIYKTPPIKDFGGFNFSQIL	20	ABCPRED
764	783	NRALTGIAVEQDKNTQEVFA	20	ABCPRED
726	745	ILPVSMTKTSVDCTMYICGD	20	ABCPRED
498	517	QPTNGVGYQPYRVVVLSFEL	20	ABCPRED
443	462	SKVGGNYNYLYRLFRKSNLK	20	ABCPRED
412	431	PGQTGKIADYNYKLPDDFTG	20	ABCPRED
399	418	SFVIRGDEVRQIAPGQTGKI	20	ABCPRED
335	354	LCPFGEVFNATRFASVYAWN	20	ABCPRED
28	47	YTNSFTRGVYYPDKVFRSSV	20	ABCPRED
1253	1272	CCKFDEDDSEPVLKGVKLHY	20	ABCPRED
1238	1257	TSCCSCLKGCCSCGSCCKFD	20	ABCPRED
916	935	LYENQKLIANQFNSAIGKIQ	20	ABCPRED
846	865	ARDLICAQKFNGLTVLPPLL	20	ABCPRED
692	711	IIAYTMSLGAENSVAYSNNS	20	ABCPRED
654	673	EHVNNSYECDIPIGAGICAS	20	ABCPRED
503	522	VGYQPYRVVVLSFELLHAPA	20	ABCPRED
446	465	GGNYNYLYRLFRKSNLKPFE	20	ABCPRED

440	459	NLDSKVGGNYNYLYRLFRKS	20	ABCPRED
433	452	VIAWNSNNLDSKVGGNYNYL	20	ABCPRED
357	376	RISNCVADYSVLYNSASFST	20	ABCPRED
35	54	GVYYPDKVFRSSVLHSTQDL	20	ABCPRED
321	340	QPTESIVRFPNITNLCPFGE	20	ABCPRED
164	183	NNCTFEYVSQPFLMDLEGKQ	20	ABCPRED
1229	1248	MVTIMLCCMTSCCSCLKGCC	20	ABCPRED
1156	1175	FKNHTSPDVDLGDISGINAS	20	ABCPRED
1148	1167	FKEELDKYFKNHTSPDVDLG	20	ABCPRED
1135	1154	NTVYDPLQPELDSFKEELDK	20	ABCPRED
1101	1120	HWFVTQRNFYEPQIITTDNT	20	ABCPRED
1046	1065	GYHLMSFPQSAPHGVVFLHV	20	ABCPRED
967	986	SSNFGAISSVLNDILSRLDK	20	ABCPRED
952	971	VNQNAQALNTLVKQLSSNFG	20	ABCPRED
813	832	SKRSFIEDLLFNKVTLADAG	20	ABCPRED
71	90	SGTNGTKRFDNPVLPFNDGV	20	ABCPRED
306	325	FTVEKGIYQTSNFRVQPTES	20	ABCPRED
255	274	SSGWTAGAAAYYVGYLQPRT	20	ABCPRED
1154	1173	KYFKNHTSPDVDLGDISGIN	20	ABCPRED
1131	1150	GIVNNTVYDPLQPELDSFKE	20	ABCPRED
1116	1135	TTDNTFVSGNCDVVIGIVNN	20	ABCPRED
1035	1054	GQSKRVDFCGKGYHLMSFPQ	20	ABCPRED
979	998	DILSRLDKVEAEVQIDRLIT	20	ABCPRED
961	980	TLVKQLSSNFGAISSVLNDI	20	ABCPRED
86	105	FNDGVYFASTEKSNIIRGWI	20	ABCPRED
828	847	LADAGFIKQYGDCLGDIAAR	20	ABCPRED
732	751	TKTSVDCTMYICGDSTECSN	20	ABCPRED
657	676	NNSYECDIPIGAGICASYQT	20	ABCPRED
640	659	SNVFQTRAGCLIGAEHVNNS	20	ABCPRED
586	605	DITPCSFGGVSVITPGTNTS	20	ABCPRED
580	599	QTLEILDITPCSFGGVSVIT	20	ABCPRED
520	539	APATVCGPKKSTNLVKNKCV	20	ABCPRED
372	391	ASFSTFKCYGVSPTKLNDLC	20	ABCPRED
32	51	FTRGVYYPDKVFRSSVLHST	20	ABCPRED
299	318	TKCTLKSFTVEKGIYQTSNF	20	ABCPRED
201	220	FKIYSKHTPINLVRDLPQGF	20	ABCPRED
1224	1243	LIAIVMVTIMLCCMTSCCSC	20	ABCPRED
1006	1025	TYVTQQLIRAAEIRASANLA	20	ABCPRED
978	997	NDILSRLDKVEAEVQIDRLI	20	ABCPRED
878	897	LAGTITSGWTFGAGAALQIP	20	ABCPRED
782	801	FAQVKQIYKTPPIKDFGGFN	20	ABCPRED
777	796	NTQEVFAQVKQIYKTPPIKD	20	ABCPRED
750	769	SNLLLQYGSFCTQLNRALTG	20	ABCPRED
562	581	FQQFGRDIADTTDAVRDPQT	20	ABCPRED
555	574	SNKKFLPFQQFGRDIADTTD	20	ABCPRED
514	533	SFELLHAPATVCGPKKSTNL	20	ABCPRED
439	458	NNLDSKVGGNYNYLYRLFRK	20	ABCPRED
40	59	DKVFRSSVLHSTQDLFLPFF	20	ABCPRED
267	286	VGYLQPRTFLLKYNENGTIT	20	ABCPRED
204	223	YSKHTPINLVRDLPQGFSAL	20	ABCPRED
180	199	EGKQGNFKNLREFVFKNIDG	20	ABCPRED
1093	1112	GVFVSNGTHWFVTQRNFYEP	20	ABCPRED
109	128	TLDSKTQSLLIVNNATNVVI	20	ABCPRED
971	990	GAISSVLNDILSRLDKVEAE	20	ABCPRED
970	989	FGAISSVLNDILSRLDKVEA	20	ABCPRED
963	982	VKQLSSNFGAISSVLNDILS	20	ABCPRED
881	900	TITSGWTFGAGAALQIPFAM	20	ABCPRED
841	860	LGDIAARDLICAQKFNGLTV	20	ABCPRED
70	89	VSGTNGTKRFDNPVLPFNDG	20	ABCPRED
694	713	AYTMSLGAENSVAYSNNSIA	20	ABCPRED
653	672	AEHVNNSYECDIPIGAGICA	20	ABCPRED
616	635	NCTEVPVAIHADQLTPTWRV	20	ABCPRED
59	78	FSNVTWFHAIHVSGTNGTKR	20	ABCPRED
584	603	ILDITPCSFGGVSVITPGTN	20	ABCPRED
582	601	LEILDITPCSFGGVSVITPG	20	ABCPRED
544	563	NGLTGTGVLTESNKKFLPFQ	20	ABCPRED
527	546	PKKSTNLVKNKCVNFNFNGL	20	ABCPRED
512	531	VLSFELLHAPATVCGPKKST	20	ABCPRED
241	260	LLALHRSYLTPGDSSSGWTA	20	ABCPRED
239	258	QTLLALHRSYLTPGDSSSGW	20	ABCPRED
1201	1220	QELGKYEQYIKWPWYIWLGF	20	ABCPRED
12	31	SSQCVNLTTRTQLPPAYTNS	20	ABCPRED
1153	1172	DKYFKNHTSPDVDLGDISGI	20	ABCPRED

1115	1134	ITTDNTFVSGNCDVVIGIVN	20	ABCPRED
1110	1129	YEPQIITTDNTFVSGNCDVV	20	ABCPRED
1047	1066	YHLMSFPQSAPHGVVFLHVT	20	ABCPRED
100	119	IIRGWIFGTTLDSKTQSLLI	20	ABCPRED
927	946	FNSAIGKIQDSLSSTASALG	20	ABCPRED
886	905	WTFGAGAALQIPFAMQMAYR	20	ABCPRED
731	750	MTKTSVDCTMYICGDSTECS	20	ABCPRED
674	693	YQTQTNSPRRARSVASQSII	20	ABCPRED
599	618	TPGTNTSNQVAVLYQDVNCT	20	ABCPRED
587	606	ITPCSFGGVSVITPGTNTSN	20	ABCPRED
490	509	FPLQSYGFQPTNGVGYQPYR	20	ABCPRED
430	449	TGCVIAWNSNNLDSKVGGNY	20	ABCPRED
380	399	YGVSPTKLNDLCFTNVYADS	20	ABCPRED
222	241	ALEPLVDLPIGINITRFQTL	20	ABCPRED
16	35	VNLTTRTQLPPAYTNSFTRG	20	ABCPRED
1213	1232	PWYIWLGFIAGLIAIVMVTI	20	ABCPRED
1146	1165	DSFKEELDKYFKNHTSPDVD	20	ABCPRED
1105	1124	TQRNFYEPQIITTDNTFVSG	20	ABCPRED
97	116	KSNIIRGWIFGTTLDSKTQS	20	ABCPRED
872	891	QYTSALLAGTITSGWTFGAG	20	ABCPRED
746	765	STECSNLLLQYGSFCTQLNR	20	ABCPRED
693	712	IAYTMSLGAENSVAYSNNSI	20	ABCPRED
609	628	AVLYQDVNCTEVPVAIHADQ	20	ABCPRED
608	627	VAVLYQDVNCTEVPVAIHAD	20	ABCPRED
564	583	QFGRDIADTTDAVRDPQTLE	20	ABCPRED
551	570	VLTESNKKFLPFQQFGRDIA	20	ABCPRED
524	543	VCGPKKSTNLVKNKCVNFNF	20	ABCPRED
500	519	TNGVGYQPYRVVVLSFELLH	20	ABCPRED
495	514	YGFQPTNGVGYQPYRVVVLS	20	ABCPRED
421	440	YNYKLPDDFTGCVIAWNSNN	20	ABCPRED
359	378	SNCVADYSVLYNSASFSTFK	20	ABCPRED
319	338	RVQPTESIVRFPNITNLCPF	20	ABCPRED
31	50	SFTRGVYYPDKVFRSSVLHS	20	ABCPRED
187	206	KNLREFVFKNIDGYFKIYSK	20	ABCPRED
169	188	EYVSQPFLMDLEGKQGNFKN	20	ABCPRED
14	33	QCVNLTTRTQLPPAYTNSFT	20	ABCPRED
1195	1214	ESLIDLQELGKYEQYIKWPW	20	ABCPRED
1183	1202	IDRLNEVAKNLNESLIDLQE	20	ABCPRED
116	135	SLLIVNNATNVVIKVCEFQF	20	ABCPRED
1127	1146	DVVIGIVNNTVYDPLQPELD	20	ABCPRED
965	984	QLSSNFGAISSVLNDILSRL	20	ABCPRED
900	919	MQMAYRFNGIGVTQNVLYEN	20	ABCPRED
867	886	DEMIAQYTSALLAGTITSGW	20	ABCPRED
853	872	QKFNGLTVLPPLLTDEMIAQ	20	ABCPRED
793	812	PIKDFGGFNFSQILPDPSKP	20	ABCPRED
715	734	PTNFTISVTTEILPVSMTKT	20	ABCPRED
61	80	NVTWFHAIHVSGTNGTKRFD	20	ABCPRED
522	541	ATVCGPKKSTNLVKNKCVNF	20	ABCPRED
516	535	ELLHAPATVCGPKKSTNLVK	20	ABCPRED
501	520	NGVGYQPYRVVVLSFELLHA	20	ABCPRED
404	423	GDEVRQIAPGQTGKIADYNY	20	ABCPRED
385	404	TKLNDLCFTNVYADSFVIRG	20	ABCPRED
305	324	SFTVEKGIYQTSNFRVQPTE	20	ABCPRED
30	49	NSFTRGVYYPDKVFRSSVLH	20	ABCPRED
22	41	TQLPPAYTNSFTRGVYYPDK	20	ABCPRED
199	218	GYFKIYSKHTPINLVRDLPQ	20	ABCPRED
1007	1026	YVTQQLIRAAEIRASANLAA	20	ABCPRED
980	999	ILSRLDKVEAEVQIDRLITG	20	ABCPRED
894	913	LQIPFAMQMAYRFNGIGVTQ	20	ABCPRED
794	813	IKDFGGFNFSQILPDPSKPS	20	ABCPRED
729	748	VSMTKTSVDCTMYICGDSTE	20	ABCPRED
727	746	LPVSMTKTSVDCTMYICGDS	20	ABCPRED
722	741	VTTEILPVSMTKTSVDCTMY	20	ABCPRED
678	697	TNSPRRARSVASQSIIAYTM	20	ABCPRED
539	558	VNFNFNGLTGTGVLTESNKK	20	ABCPRED
338	357	FGEVFNATRFASVYAWNRKR	20	ABCPRED
316	335	SNFRVQPTESIVRFPNITNL	20	ABCPRED
310	329	KGIYQTSNFRVQPTESIVRF	20	ABCPRED
289	308	VDCALDPLSETKCTLKSFTV	20	ABCPRED
20	39	TRTQLPPAYTNSFTRGVYYP	20	ABCPRED
170	189	YVSQPFLMDLEGKQGNFKNL	20	ABCPRED
1196	1215	SLIDLQELGKYEQYIKWPWY	20	ABCPRED
115	134	QSLLIVNNATNVVIKVCEFQ	20	ABCPRED

1068	1087	VPAQEKNFTTAPAICHDGKA	20	ABCPRED
976	995	VLNDILSRLDKVEAEVQIDR	20	ABCPRED
871	890	AQYTSALLAGTITSGWTFGA	20	ABCPRED
857	876	GLTVLPPLLTDEMIAQYTSA	20	ABCPRED
851	870	CAQKFNGLTVLPPLLTDEMI	20	ABCPRED
825	844	KVTLADAGFIKQYGDCLGDI	20	ABCPRED
645	664	TRAGCLIGAEHVNNSYECDI	20	ABCPRED
471	490	EIYQAGSTPCNGVEGFNCYF	20	ABCPRED
415	434	TGKIADYNYKLPDDFTGCVI	20	ABCPRED
383	402	SPTKLNDLCFTNVYADSFVI	20	ABCPRED
318	337	FRVQPTESIVRFPNITNLCP	20	ABCPRED
303	322	LKSFTVEKGIYQTSNFRVQP	20	ABCPRED
205	224	SKHTPINLVRDLPQGFSALE	20	ABCPRED
177	196	MDLEGKQGNFKNLREFVFKN	20	ABCPRED
1152	1171	LDKYFKNHTSPDVDLGDISG	20	ABCPRED
1133	1152	VNNTVYDPLQPELDSFKEEL	20	ABCPRED
1081	1100	ICHDGKAHFPREGVFVSNGT	20	ABCPRED
105	124	IFGTTLDSKTQSLLIVNNAT	20	ABCPRED
954	973	QNAQALNTLVKQLSSNFGAI	20	ABCPRED
905	924	RFNGIGVTQNVLYENQKLIA	20	ABCPRED
902	921	MAYRFNGIGVTQNVLYENQK	20	ABCPRED
861	880	LPPLLTDEMIAQYTSALLAG	20	ABCPRED
775	794	DKNTQEVFAQVKQIYKTPPI	20	ABCPRED
706	725	AYSNNSIAIPTNFTISVTTE	20	ABCPRED
641	660	NVFQTRAGCLIGAEHVNNSY	20	ABCPRED
612	631	YQDVNCTEVPVAIHADQLTP	20	ABCPRED
579	598	PQTLEILDITPCSFGGVSVI	20	ABCPRED
538	557	CVNFNFNGLTGTGVLTESNK	20	ABCPRED
453	472	YRLFRKSNLKPFERDISTEI	20	ABCPRED
281	300	ENGTITDAVDCALDPLSETK	20	ABCPRED
279	298	YNENGTITDAVDCALDPLSE	20	ABCPRED
268	287	GYLQPRTFLLKYNENGTITD	20	ABCPRED
232	251	GINITRFQTLLALHRSYLTP	20	ABCPRED
198	217	DGYFKIYSKHTPINLVRDLP	20	ABCPRED
1237	1256	MTSCCSCLKGCCSCGSCCKF	20	ABCPRED
121	140	NNATNVVIKVCEFQFCNDPF	20	ABCPRED
1203	1222	LGKYEQYIKWPWYIWLGFIA	20	ABCPRED
113	132	KTQSLLIVNNATNVVIKVCE	20	ABCPRED
1064	1083	HVTYVPAQEKNFTTAPAICH	20	ABCPRED
102	121	RGWIFGTTLDSKTQSLLIVN	20	ABCPRED
1001	1020	LQSLQTYVTQQLIRAAEIRA	20	ABCPRED
868	887	EMIAQYTSALLAGTITSGWT	20	ABCPRED
840	859	CLGDIAARDLICAQKFNGLT	20	ABCPRED
758	777	SFCTQLNRALTGIAVEQDKN	20	ABCPRED
757	776	GSFCTQLNRALTGIAVEQDK	20	ABCPRED
737	756	DCTMYICGDSTECSNLLLQY	20	ABCPRED
723	742	TTEILPVSMTKTSVDCTMYI	20	ABCPRED
719	738	TISVTTEILPVSMTKTSVDC	20	ABCPRED
661	680	ECDIPIGAGICASYQTQTNS	20	ABCPRED
649	668	CLIGAEHVNNSYECDIPIGA	20	ABCPRED
58	77	FFSNVTWFHAIHVSGTNGTK	20	ABCPRED
532	551	NLVKNKCVNFNFNGLTGTGV	20	ABCPRED
519	538	HAPATVCGPKKSTNLVKNKC	20	ABCPRED
487	506	NCYFPLQSYGFQPTNGVGYQ	20	ABCPRED
469	488	STEIYQAGSTPCNGVEGFNC	20	ABCPRED
458	477	KSNLKPFERDISTEIYQAGS	20	ABCPRED
450	469	NYLYRLFRKSNLKPFERDIS	20	ABCPRED
429	448	FTGCVIAWNSNNLDSKVGGN	20	ABCPRED
424	443	KLPDDFTGCVIAWNSNNLDS	20	ABCPRED
407	426	VRQIAPGQTGKIADYNYKLP	20	ABCPRED
379	398	CYGVSPTKLNDLCFTNVYAD	20	ABCPRED
378	397	KCYGVSPTKLNDLCFTNVYA	20	ABCPRED
330	349	PNITNLCPFGEVFNATRFAS	20	ABCPRED
308	327	VEKGIYQTSNFRVQPTESIV	20	ABCPRED
242	261	LALHRSYLTPGDSSSGWTAG	20	ABCPRED
225	244	PLVDLPIGINITRFQTLLAL	20	ABCPRED
15	34	CVNLTTRTQLPPAYTNSFTR	20	ABCPRED
1200	1219	LQELGKYEQYIKWPWYIWLG	20	ABCPRED
114	133	TQSLLIVNNATNVVIKVCEF	20	ABCPRED
1137	1156	VYDPLQPELDSFKEELDKYF	20	ABCPRED
1052	1071	FPQSAPHGVVFLHVTYVPAQ	20	ABCPRED
863	882	PLLTDEMIAQYTSALLAGTI	20	ABCPRED
860	879	VLPPLLTDEMIAQYTSALLA	20	ABCPRED

859	878	TVLPPLLTDEMIAQYTSALL	20	ABCPRED
833	852	FIKQYGDCLGDIAARDLICA	20	ABCPRED
829	848	ADAGFIKQYGDCLGDIAARD	20	ABCPRED
770	789	IAVEQDKNTQEVFAQVKQIY	20	ABCPRED
761	780	TQLNRALTGIAVEQDKNTQE	20	ABCPRED
680	699	SPRRARSVASQSIIAYTMSL	20	ABCPRED
64	83	WFHAIHVSGTNGTKRFDNPV	20	ABCPRED
581	600	TLEILDITPCSFGGVSVITP	20	ABCPRED
57	76	PFFSNVTWFHAIHVSGTNGT	20	ABCPRED
494	513	SYGFQPTNGVGYQPYRVVVL	20	ABCPRED
483	502	VEGFNCYFPLQSYGFQPTNG	20	ABCPRED
438	457	SNNLDSKVGGNYNYLYRLFR	20	ABCPRED
390	409	LCFTNVYADSFVIRGDEVRQ	20	ABCPRED
350	369	VYAWNRKRISNCVADYSVLY	20	ABCPRED
154	173	ESEFRVYSSANNCTFEYVSQ	20	ABCPRED
148	167	NNKSWMESEFRVYSSANNCT	20	ABCPRED
141	160	LGVYYHKNNKSWMESEFRVY	20	ABCPRED
139	158	PFLGVYYHKNNKSWMESEFR	20	ABCPRED
137	156	NDPFLGVYYHKNNKSWMESE	20	ABCPRED
1228	1247	VMVTIMLCCMTSCCSCLKGC	20	ABCPRED
1197	1216	LIDLQELGKYEQYIKWPWYI	20	ABCPRED
1097	1116	SNGTHWFVTQRNFYEPQIIT	20	ABCPRED
1028	1047	KMSECVLGQSKRVDFCGKGY	20	ABCPRED
1010	1029	QQLIRAAEIRASANLAATKM	20	ABCPRED
10	29	LVSSQCVNLTTRTQLPPAYT	20	ABCPRED
989	1008	AEVQIDRLITGRLQSLQTYV	20	ABCPRED
955	974	NAQALNTLVKQLSSNFGAIS	20	ABCPRED
950	969	DVVNQNAQALNTLVKQLSSN	20	ABCPRED
948	967	LQDVVNQNAQALNTLVKQLS	20	ABCPRED
849	868	LICAQKFNGLTVLPPLLTDE	20	ABCPRED
848	867	DLICAQKFNGLTVLPPLLTD	20	ABCPRED
772	791	VEQDKNTQEVFAQVKQIYKT	20	ABCPRED
753	772	LLQYGSFCTQLNRALTGIAV	20	ABCPRED
745	764	DSTECSNLLLQYGSFCTQLN	20	ABCPRED
733	752	KTSVDCTMYICGDSTECSNL	20	ABCPRED
686	705	SVASQSIIAYTMSLGAENSV	20	ABCPRED
658	677	NSYECDIPIGAGICASYQTQ	20	ABCPRED
633	652	WRVYSTGSNVFQTRAGCLIG	20	ABCPRED
605	624	SNQVAVLYQDVNCTEVPVAI	20	ABCPRED
603	622	NTSNQVAVLYQDVNCTEVPV	20	ABCPRED
601	620	GTNTSNQVAVLYQDVNCTEV	20	ABCPRED
530	549	STNLVKNKCVNFNFNGLTGT	20	ABCPRED
437	456	NSNNLDSKVGGNYNYLYRLF	20	ABCPRED
402	421	IRGDEVRQIAPGQTGKIADY	20	ABCPRED
313	332	YQTSNFRVQPTESIVRFPNI	20	ABCPRED
257	276	GWTAGAAAYYVGYLQPRTFL	20	ABCPRED
202	221	KIYSKHTPINLVRDLPQGFS	20	ABCPRED
192	211	FVFKNIDGYFKIYSKHTPIN	20	ABCPRED
1106	1125	QRNFYEPQIITTDNTFVSGN	20	ABCPRED
1069	1088	PAQEKNFTTAPAICHDGKAH	20	ABCPRED
1017	1036	EIRASANLAATKMSECVLGQ	20	ABCPRED
9	28	PLVSSQCVNLTTRTQLPPAY	20	ABCPRED
882	901	ITSGWTFGAGAALQIPFAMQ	20	ABCPRED
870	889	IAQYTSALLAGTITSGWTFG	20	ABCPRED
734	753	TSVDCTMYICGDSTECSNLL	20	ABCPRED
701	720	AENSVAYSNNSIAIPTNFTI	20	ABCPRED
659	678	SYECDIPIGAGICASYQTQT	20	ABCPRED
65	84	FHAIHVSGTNGTKRFDNPVL	20	ABCPRED
610	629	VLYQDVNCTEVPVAIHADQL	20	ABCPRED
585	604	LDITPCSFGGVSVITPGTNT	20	ABCPRED
578	597	DPQTLEILDITPCSFGGVSV	20	ABCPRED
504	523	GYQPYRVVVLSFELLHAPAT	20	ABCPRED
482	501	GVEGFNCYFPLQSYGFQPTN	20	ABCPRED
481	500	NGVEGFNCYFPLQSYGFQPT	20	ABCPRED
414	433	QTGKIADYNYKLPDDFTGCV	20	ABCPRED
216	235	LPQGFSALEPLVDLPIGINI	20	ABCPRED
162	181	SANNCTFEYVSQPFLMDLEG	20	ABCPRED
1247	1266	CCSCGSCCKFDEDDSEPVLK	20	ABCPRED
122	141	NATNVVIKVCEFQFCNDPFL	20	ABCPRED
1175	1194	SVVNIQKEIDRLNEVAKNLN	20	ABCPRED
1117	1136	TDNTFVSGNCDVVIGIVNNT	20	ABCPRED
1114	1133	IITTDNTFVSGNCDVVIGIV	20	ABCPRED
1084	1103	DGKAHFPREGVFVSNGTHWF	20	ABCPRED

1050	1069	MSFPQSAPHGVVFLHVTYVP	20	ABCPRED
99	118	NIIRGWIFGTTLDSKTQSLL	20	ABCPRED
94	113	STEKSNIIRGWIFGTTLDSK	20	ABCPRED
934	953	IQDSLSSTASALGKLQDVVN	20	ABCPRED
930	949	AIGKIQDSLSSTASALGKLQ	20	ABCPRED
786	805	KQIYKTPPIKDFGGFNFSQI	20	ABCPRED
752	771	LLLQYGSFCTQLNRALTGIA	20	ABCPRED
708	727	SNNSIAIPTNFTISVTTEIL	20	ABCPRED
618	637	TEVPVAIHADQLTPTWRVYS	20	ABCPRED
593	612	GGVSVITPGTNTSNQVAVLY	20	ABCPRED
547	566	TGTGVLTESNKKFLPFQQFG	20	ABCPRED
537	556	KCVNFNFNGLTGTGVLTESN	20	ABCPRED
48	67	LHSTQDLFLPFFSNVTWFHA	20	ABCPRED
271	290	QPRTFLLKYNENGTITDAVD	20	ABCPRED
210	229	INLVRDLPQGFSALEPLVDL	20	ABCPRED
208	227	TPINLVRDLPQGFSALEPLV	20	ABCPRED
173	192	QPFLMDLEGKQGNFKNLREF	20	ABCPRED
1235	1254	CCMTSCCSCLKGCCSCGSCC	20	ABCPRED
1161	1180	SPDVDLGDISGINASVVNIQ	20	ABCPRED
1139	1158	DPLQPELDSFKEELDKYFKN	20	ABCPRED
1060	1079	VVFLHVTYVPAQEKNFTTAP	20	ABCPRED
901	920	QMAYRFNGIGVTQNVLYENQ	20	ABCPRED
890	909	AGAALQIPFAMQMAYRFNGI	20	ABCPRED
824	843	NKVTLADAGFIKQYGDCLGD	20	ABCPRED
804	823	QILPDPSKPSKRSFIEDLLF	20	ABCPRED
797	816	FGGFNFSQILPDPSKPSKRS	20	ABCPRED
791	810	TPPIKDFGGFNFSQILPDPS	20	ABCPRED
635	654	VYSTGSNVFQTRAGCLIGAE	20	ABCPRED
575	594	AVRDPQTLEILDITPCSFGG	20	ABCPRED
528	547	KKSTNLVKNKCVNFNFNGLT	20	ABCPRED
513	532	LSFELLHAPATVCGPKKSTN	20	ABCPRED
506	525	QPYRVVVLSFELLHAPATVC	20	ABCPRED
493	512	QSYGFQPTNGVGYQPYRVVV	20	ABCPRED
49	68	HSTQDLFLPFFSNVTWFHAI	20	ABCPRED
245	264	HRSYLTPGDSSSGWTAGAAA	20	ABCPRED
226	245	LVDLPIGINITRFQTLLALH	20	ABCPRED
194	213	FKNIDGYFKIYSKHTPINLV	20	ABCPRED
185	204	NFKNLREFVFKNIDGYFKIY	20	ABCPRED
184	203	GNFKNLREFVFKNIDGYFKI	20	ABCPRED
181	200	GKQGNFKNLREFVFKNIDGY	20	ABCPRED
156	175	EFRVYSSANNCTFEYVSQPF	20	ABCPRED
1218	1237	LGFIAGLIAIVMVTIMLCCM	20	ABCPRED
1103	1122	FVTQRNFYEPQIITTDNTFV	20	ABCPRED
1088	1107	HFPREGVFVSNGTHWFVTQR	20	ABCPRED
1054	1073	QSAPHGVVFLHVTYVPAQEK	20	ABCPRED
1048	1067	HLMSFPQSAPHGVVFLHVTY	20	ABCPRED
1018	1037	IRASANLAATKMSECVLGQS	20	ABCPRED
893	912	ALQIPFAMQMAYRFNGIGVT	20	ABCPRED
87	106	NDGVYFASTEKSNIIRGWIF	20	ABCPRED
814	833	KRSFIEDLLFNKVTLADAGF	20	ABCPRED
796	815	DFGGFNFSQILPDPSKPSKR	20	ABCPRED
792	811	PPIKDFGGFNFSQILPDPSK	20	ABCPRED
676	695	TQTNSPRRARSVASQSIIAY	20	ABCPRED
572	591	TTDAVRDPQTLEILDITPCS	20	ABCPRED
511	530	VVLSFELLHAPATVCGPKKS	20	ABCPRED
462	481	KPFERDISTEIYQAGSTPCN	20	ABCPRED
386	405	KLNDLCFTNVYADSFVIRGD	20	ABCPRED
354	373	NRKRISNCVADYSVLYNSAS	20	ABCPRED
345	364	TRFASVYAWNRKRISNCVAD	20	ABCPRED
324	343	ESIVRFPNITNLCPFGEVFN	20	ABCPRED
315	334	TSNFRVQPTESIVRFPNITN	20	ABCPRED
312	331	IYQTSNFRVQPTESIVRFPN	20	ABCPRED
223	242	LEPLVDLPIGINITRFQTLL	20	ABCPRED
161	180	SSANNCTFEYVSQPFLMDLE	20	ABCPRED
130	149	VCEFQFCNDPFLGVYYHKNN	20	ABCPRED
1227	1246	IVMVTIMLCCMTSCCSCLKG	20	ABCPRED
1155	1174	YFKNHTSPDVDLGDISGINA	20	ABCPRED
1095	1114	FVSNGTHWFVTQRNFYEPQI	20	ABCPRED
1039	1058	RVDFCGKGYHLMSFPQSAPH	20	ABCPRED
960	979	NTLVKQLSSNFGAISSVLND	20	ABCPRED
898	917	FAMQMAYRFNGIGVTQNVLY	20	ABCPRED
896	915	IPFAMQMAYRFNGIGVTQNV	20	ABCPRED
862	881	PPLLTDEMIAQYTSALLAGT	20	ABCPRED

805	824	ILPDPSKPSKRSFIEDLLFN	20	ABCPRED
800	819	FNFSQILPDPSKPSKRSFIE	20	ABCPRED
788	807	IYKTPPIKDFGGFNFSQILP	20	ABCPRED
714	733	IPTNFTISVTTEILPVSMTK	20	ABCPRED
672	691	ASYQTQTNSPRRARSVASQS	20	ABCPRED
56	75	LPFFSNVTWFHAIHVSGTNG	20	ABCPRED
480	499	CNGVEGFNCYFPLQSYGFQP	20	ABCPRED
4	23	FLVLLPLVSSQCVNLTTRTQ	20	ABCPRED
397	416	ADSFVIRGDEVRQIAPGQTG	20	ABCPRED
266	285	YVGYLQPRTFLLKYNENGTI	20	ABCPRED
250	269	TPGDSSSGWTAGAAAYYVGY	20	ABCPRED
228	247	DLPIGINITRFQTLLALHRS	20	ABCPRED
214	233	RDLPQGFSALEPLVDLPIGI	20	ABCPRED
1193	1212	LNESLIDLQELGKYEQYIKW	20	ABCPRED
1143	1162	PELDSFKEELDKYFKNHTSP	20	ABCPRED
1109	1128	FYEPQIITTDNTFVSGNCDV	20	ABCPRED
1200	1211	LQELGKYEQYIK	12	AAP
1111	1122	EPQIITTDNTFV	12	AAP
80	91	DNPVLPFNDGVY	12	AAP
312	323	IYQTSNFRVQPT	12	AAP
398	409	DSFVIRGDEVRQ	12	AAP
1064	1075	HVTYVPAQEKNF	12	AAP
101	112	IRGWIFGTTLDS	12	AAP
1185	1196	RLNEVAKNLNES	12	AAP
141	152	LGVYYHKNNKSW	12	AAP
31	42	SFTRGVYYPDKV	12	AAP
673	684	SYQTQTNSPRRA	12	AAP
627	638	DQLTPTWRVYST	12	AAP
1007	1018	YVTQQLIRAAEI	12	AAP
906	917	FNGIGVTQNVLY	12	AAP
599	610	TPGTNTSNQVAV	12	AAP
572	583	TTDAVRDPQTLE	12	AAP
772	783	VEQDKNTQEVFA	12	AAP
753	764	LLQYGSFCTQLN	12	AAP
1096	1107	VSNGTHWFVTQR	12	AAP
380	391	YGVSPTKLNDLC	12	AAP
419	430	ADYNYKLPDDFT	12	AAP
947	958	KLQDVVNQNAQA	12	AAP
1134	1145	NNTVYDPLQPEL	12	AAP
439	450	NNLDSKVGGNYN	12	AAP
694	705	AYTMSLGAENSV	12	AAP
249	260	LTPGDSSSGWTA	12	AAP
786	797	KQIYKTPPIKDF	12	AAP
285	296	ITDAVDCALDPL	12	AAP
523	534	TVCGPKKSTNLV	12	AAP
612	623	YQDVNCTEVPVA	12	AAP
1073	1086	KNFTTAPAICHDGK	14	AAP
981	994	LSRLDKVEAEVQID	14	AAP
404	417	GDEVRQIAPGQTGK	14	AAP
314	327	QTSNFRVQPTESIV	14	AAP
768	781	TGIAVEQDKNTQEV	14	AAP
1108	1121	NFYEPQIITTDNTF	14	AAP
247	260	SYLTPGDSSSGWTA	14	AAP
19	32	TTRTQLPPAYTNSF	14	AAP
723	736	TTEILPVSMTKTSV	14	AAP
883	896	TSGWTFGAGAALQI	14	AAP
949	962	QDVVNQNAQALNTL	14	AAP
70	83	VSGTNGTKRFDNPV	14	AAP
520	533	APATVCGPKKSTNL	14	AAP
180	193	EGKQGNFKNLREFV	14	AAP
571	584	DTTDAVRDPQTLEI	14	AAP
283	296	GTITDAVDCALDPL	14	AAP
609	622	AVLYQDVNCTEVPV	14	AAP
753	766	LLQYGSFCTQLNRA	14	AAP
1053	1066	PQSAPHGVVFLHVT	14	AAP
161	174	SSANNCTFEYVSQP	14	AAP
145	158	YHKNNKSWMESEFR	14	AAP
1013	1026	IRAAEIRASANLAA	14	AAP
1251	1264	GSCCKFDEDDSEPV	14	AAP
1200	1213	LQELGKYEQYIKWP	14	AAP
1138	1151	YDPLQPELDSFKEE	14	AAP
437	450	NSNNLDSKVGGNYN	14	AAP
100	113	IIRGWIFGTTLDSK	14	AAP

924	937	ANQFNSAIGKIQDS	14	AAP
1159	1172	HTSPDVDLGDISGI	14	AAP
783	796	AQVKQIYKTPPIKD	14	AAP
738	751	CTMYICGDSTECSN	14	AAP
262	275	AAAYYVGYLQPRTF	14	AAP
375	388	STFKCYGVSPTKLN	14	AAP
835	848	KQYGDCLGDIAARD	14	AAP
43	56	FRSSVLHSTQDLFL	14	AAP
1131	1146	GIVNNTVYDPLQPELD	16	AAP
1160	1175	TSPDVDLGDISGINAS	16	AAP
251	266	PGDSSSGWTAGAAAYY	16	AAP
722	737	VTTEILPVSMTKTSVD	16	AAP
772	787	VEQDKNTQEVFAQVKQ	16	AAP
1103	1118	FVTQRNFYEPQIITTD	16	AAP
338	353	FGEVFNATRFASVYAW	16	AAP
607	622	QVAVLYQDVNCTEVPV	16	AAP
743	758	CGDSTECSNLLLQYGS	16	AAP
76	91	TKRFDNPVLPFNDGVY	16	AAP
402	417	IRGDEVRQIAPGQTGK	16	AAP
16	31	VNLTTRTQLPPAYTNS	16	AAP
1258	1273	EDDSEPVLKGVKLHYT	16	AAP
275	290	FLLKYNENGTITDAVD	16	AAP
877	892	LLAGTITSGWTFGAGA	16	AAP
315	330	TSNFRVQPTESIVRFP	16	AAP
520	535	APATVCGPKKSTNLVK	16	AAP
1008	1023	VTQQLIRAAEIRASAN	16	AAP
473	488	YQAGSTPCNGVEGFNC	16	AAP
572	587	TTDAVRDPQTLEILDI	16	AAP
499	514	PTNGVGYQPYRVVVLS	16	AAP
1086	1101	KAHFPREGVFVSNGTH	16	AAP
153	168	MESEFRVYSSANNCTF	16	AAP
1065	1080	VTYVPAQEKNFTTAPA	16	AAP
180	195	EGKQGNFKNLREFVFK	16	AAP
100	115	IIRGWIFGTTLDSKTQ	16	AAP
136	151	CNDPFLGVYYHKNNKS	16	AAP
834	849	IKQYGDCLGDIAARDL	16	AAP
1200	1215	LQELGKYEQYIKWPWY	16	AAP
590	605	CSFGGVSVITPGTNTS	16	AAP
435	450	AWNSNNLDSKVGGNYN	16	AAP
33	48	TRGVYYPDKVFRSSVL	16	AAP
894	909	LQIPFAMQMAYRFNGI	16	AAP
456	471	FRKSNLKPFERDISTE	16	AAP
797	812	FGGFNFSQILPDPSKP	16	AAP
1233	1248	MLCCMTSCCSCLKGCC	16	AAP
57	72	PFFSNVTWFHAIHVSG	16	AAP
537	552	KCVNFNFNGLTGTGVL	16	AAP
1133	1150	VNNTVYDPLQPELDSFKE	18	AAP
523	540	TVCGPKKSTNLVKNKCVN	18	AAP
294	311	DPLSETKCTLKSFTVEKG	18	AAP
18	35	LTTRTQLPPAYTNSFTRG	18	AAP
591	608	SFGGVSVITPGTNTSNQV	18	AAP
401	418	VIRGDEVRQIAPGQTGKI	18	AAP
679	696	NSPRRARSVASQSIIAYT	18	AAP
259	276	TAGAAAYYVGYLQPRTFL	18	AAP
106	123	FGTTLDSKTQSLLIVNNA	18	AAP
722	739	VTTEILPVSMTKTSVDCT	18	AAP
498	515	QPTNGVGYQPYRVVVLSF	18	AAP
1084	1101	DGKAHFPREGVFVSNGTH	18	AAP
1109	1126	FYEPQIITTDNTFVSGNC	18	AAP
348	365	ASVYAWNRKRISNCVADY	18	AAP
370	387	NSASFSTFKCYGVSPTKL	18	AAP
803	820	SQILPDPSKPSKRSFIED	18	AAP
314	331	QTSNFRVQPTESIVRFPN	18	AAP
1256	1273	FDEDDSEPVLKGVKLHYT	18	AAP
572	589	TTDAVRDPQTLEILDITP	18	AAP
783	800	AQVKQIYKTPPIKDFGGF	18	AAP
1009	1026	TQQLIRAAEIRASANLAA	18	AAP
746	763	STECSNLLLQYGSFCTQL	18	AAP
148	165	NNKSWMESEFRVYSSANN	18	AAP
1029	1046	MSECVLGQSKRVDFCGKG	18	AAP
652	669	GAEHVNNSYECDIPIGAG	18	AAP
475	492	AGSTPCNGVEGFNCYFPL	18	AAP
615	632	VNCTEVPVAIHADQLTPT	18	AAP

216	233	LPQGFSALEPLVDLPIGI	18	AAP
1050	1067	MSFPQSAPHGVVFLHVTY	18	AAP
69	86	HVSGTNGTKRFDNPVLPF	18	AAP
823	840	FNKVTLADAGFIKQYGDC	18	AAP
950	967	DVVNQNAQALNTLVKQLS	18	AAP
927	944	FNSAIGKIQDSLSSTASA	18	AAP
443	460	SKVGGNYNYLYRLFRKSN	18	AAP
899	916	AMQMAYRFNGIGVTQNVL	18	AAP
700	717	GAENSVAYSNNSIAIPTN	18	AAP
719	738	TISVTTEILPVSMTKTSVDC	20	AAP
250	269	TPGDSSSGWTAGAAAYYVGY	20	AAP
148	167	NNKSWMESEFRVYSSANNCT	20	AAP
603	622	NTSNQVAVLYQDVNCTEVPV	20	AAP
22	41	TQLPPAYTNSFTRGVYYPDK	20	AAP
1251	1270	GSCCKFDEDDSEPVLKGVKL	20	AAP
399	418	SFVIRGDEVRQIAPGQTGKI	20	AAP
1159	1178	HTSPDVDLGDISGINASVVN	20	AAP
308	327	VEKGIYQTSNFRVQPTESIV	20	AAP
107	126	GTTLDSKTQSLLIVNNATNV	20	AAP
79	98	FDNPVLPFNDGVYFASTEKS	20	AAP
513	532	LSFELLHAPATVCGPKKSTN	20	AAP
873	892	YTSALLAGTITSGWTFGAGA	20	AAP
672	691	ASYQTQTNSPRRARSVASQS	20	AAP
777	796	NTQEVFAQVKQIYKTPPIKD	20	AAP
271	290	QPRTFLLKYNENGTITDAVD	20	AAP
1117	1136	TDNTFVSGNCDVVIGIVNNT	20	AAP
1062	1081	FLHVTYVPAQEKNFTTAPAI	20	AAP
630	649	TPTWRVYSTGSNVFQTRAGC	20	AAP
1083	1102	HDGKAHFPREGVFVSNGTHW	20	AAP
421	440	YNYKLPDDFTGCVIAWNSNN	20	AAP
743	762	CGDSTECSNLLLQYGSFCTQ	20	AAP
1027	1046	TKMSECVLGQSKRVDFCGKG	20	AAP
378	397	KCYGVSPTKLNDLCFTNVYA	20	AAP
1002	1021	QSLQTYVTQQLIRAAEIRAS	20	AAP
1196	1215	SLIDLQELGKYEQYIKWPWY	20	AAP
397	418	ADSFVIRGDEVRQIAPGQTGKI	22	AAP
818	839	IEDLLFNKVTLADAGFIKQYGD	22	AAP
574	595	DAVRDPQTLEILDITPCSFGGV	22	AAP
269	290	YLQPRTFLLKYNENGTITDAVD	22	AAP
1104	1125	VTQRNFYEPQIITTDNTFVSGN	22	AAP
1067	1088	YVPAQEKNFTTAPAICHDGKAH	22	AAP
600	621	PGTNTSNQVAVLYQDVNCTEVP	22	AAP
304	325	KSFTVEKGIYQTSNFRVQPTES	22	AAP
21	42	RTQLPPAYTNSFTRGVYYPDKV	22	AAP
1134	1155	NNTVYDPLQPELDSFKEELDKY	22	AAP
664	685	IPIGAGICASYQTQTNSPRRAR	22	AAP
473	494	YQAGSTPCNGVEGFNCYFPLQS	22	AAP
93	114	ASTEKSNIIRGWIFGTTLDSKT	22	AAP
708	729	SNNSIAIPTNFTISVTTEILPV	22	AAP
779	800	QEVFAQVKQIYKTPPIKDFGGF	22	AAP
547	568	TGTGVLTESNKKFLPFQQFGRD	22	AAP
70	91	VSGTNGTKRFDNPVLPFNDGVY	22	AAP
246	267	RSYLTPGDSSSGWTAGAAAYYV	22	AAP
1194	1215	NESLIDLQELGKYEQYIKWPWY	22	AAP
163	184	ANNCTFEYVSQPFLMDLEGKQG	22	AAP
1252	1273	SCCKFDEDDSEPVLKGVKLHYT	22	AAP
429	450	FTGCVIAWNSNNLDSKVGGNYN	22	AAP
952	973	VNQNAQALNTLVKQLSSNFGAI	22	AAP
743	764	CGDSTECSNLLLQYGSFCTQLN	22	AAP
872	893	QYTSALLAGTITSGWTFGAGAA	22	AAP
369	390	YNSASFSTFKCYGVSPTKLNDL	22	AAP
17	46	NLTTRTQLPPAYTNSFTRGVYYPDKVFRSS	30	BCEPRED
71	83	SGTNGTKRFDNPV	13	BCEPRED
91	102	YFASTEKSNIIR	12	BCEPRED
108	117	TTLDSKTQSL	10	BCEPRED
142	160	GVYYHKNNKSWMESEFRVY	19	BCEPRED
177	192	MDLEGKQGNFKNLREF	16	BCEPRED
200	211	YFKIYSKHTPIN	12	BCEPRED
268	284	GYLQPRTFLLKYNENGT	17	BCEPRED
294	325	DPLSETKCTLKSFTVEKGIYQTSNFRVQPTES	32	BCEPRED
350	361	VYAWNRKRISNC	12	BCEPRED
383	390	SPTKLNDL	8	BCEPRED
417	430	KIADYNYKLPDDFT	14	BCEPRED

437	445	NSNNLDSKV	9	BCEPRED
447	471	GNYNYLYRLFRKSNLKPFERDISTE	25	BCEPRED
503	511	VGYQPYRVV	9	BCEPRED
525	537	CGPKKSTNLVKNK	13	BCEPRED
552	561	LTESNKKFLP	10	BCEPRED
573	583	TDAVRDPQTLE	11	BCEPRED
599	608	TPGTNTSNQV	10	BCEPRED
654	663	EHVNNSYECD	10	BCEPRED
672	688	ASYQTQTNSPRRARSVA	17	BCEPRED
771	782	AVEQDKNTQEVF	12	BCEPRED
806	820	LPDPSKPSKRSFIED	15	BCEPRED
913	923	QNVLYENQKLI	11	BCEPRED
982	989	SRLDKVEA	8	BCEPRED
1000	1011	RLQSLQTYVTQQ	12	BCEPRED
1035	1042	GQSKRVDF	8	BCEPRED
1067	1079	YVPAQEKNFTTAP	13	BCEPRED
1104	1113	VTQRNFYEPQ	10	BCEPRED
1134	1163	NNTVYDPLQPELDSFKEELDKYFKNHTSPD	30	BCEPRED
1178	1197	NIQKEIDRLNEVAKNLNESL	20	BCEPRED
1201	1213	QELGKYEQYIKWP	13	BCEPRED
1254	1264	CKFDEDDSEPV	11	BCEPRED
1	20	MFVFLVLLPLVSSQCVNLTT	20	BCEPRED
36	62	VYYPDKVFRSSVLHSTQDLFLPFFSNV	27	BCEPRED
114	121	TQSLLIVN	8	BCEPRED
124	147	TNVVIKVCEFQFCNDPFLGVYYHK	24	BCEPRED
166	177	CTFEYVSQPFLM	12	BCEPRED
207	218	HTPINLVRDLPQ	12	BCEPRED
223	233	LEPLVDLPIGI	11	BCEPRED
265	273	YYVGYLQPR	9	BCEPRED
332	342	ITNLCPFGEVF	11	BCEPRED
364	371	DYSVLYNS	8	BCEPRED
374	385	FSTFKCYGVSPT	12	BCEPRED
389	396	DLCFTNVY	8	BCEPRED
449	457	YNYLYRLFR	9	BCEPRED
486	495	FNCYFPLQSY	10	BCEPRED
505	519	YQPYRVVVLSFELLH	15	BCEPRED
584	601	ILDITPCSFGGVSVITPG	18	BCEPRED
610	622	VLYQDVNCTEVPV	13	BCEPRED
736	744	VDCTMYICG	9	BCEPRED
748	760	ECSNLLLQYGSFC	13	BCEPRED
857	867	GLTVLPPLLTD	11	BCEPRED
946	954	GKLQDVVNQ	9	BCEPRED
961	968	TLVKQLSS	8	BCEPRED
975	982	SVLNDILS	8	BCEPRED
1001	1013	LQSLQTYVTQQLI	13	BCEPRED
1028	1038	KMSECVLGQSK	11	BCEPRED
1057	1069	PHGVVFLHVTYVP	13	BCEPRED
1122	1134	VSGNCDVVIGIVN	13	BCEPRED
1136	1143	TVYDPLQP	8	BCEPRED
1227	1256	IVMVTIMLCCMTSCCSCLKGCCSCGSCCKF	30	BCEPRED
1262	1273	EPVLKGVKLHYT	12	BCEPRED
144	153	YYHKNNKSWM	10	BCEPRED
351	360	YAWNRKRISN	10	BCEPRED
456	463	FRKSNLKP	8	BCEPRED
526	533	GPKKSTNL	8	BCEPRED
552	559	LTESNKKF	8	BCEPRED
677	685	QTNSPRRAR	9	BCEPRED
772	781	VEQDKNTQEV	10	BCEPRED
784	796	QVKQIYKTPPIKD	13	BCEPRED
808	817	DPSKPSKRSF	10	BCEPRED
1148	1160	FKEELDKYFKNHT	13	BCEPRED
1179	1186	IQKEIDRL	8	BCEPRED
68	76	IHVSGTNGT	9	BCEPRED
90	97	VYFASTEK	8	BCEPRED
108	115	TTLDSKTQ	8	BCEPRED
247	255	SYLTPGDSS	9	BCEPRED
351	358	YAWNRKRI	8	BCEPRED
439	446	NNLDSKVG	8	BCEPRED
454	461	RLFRKSNL	8	BCEPRED
522	531	ATVCGPKKST	10	BCEPRED
550	557	GVLTESNK	8	BCEPRED
597	605	VITPGTNTS	9	BCEPRED
672	684	ASYQTQTNSPRRA	13	BCEPRED

742	749	ICGDSTEC	8	BCEPRED
770	778	IAVEQDKNT	9	BCEPRED
804	815	QILPDPSKPSKR	12	BCEPRED
932	939	GKIQDSLS	8	BCEPRED
1031	1039	ECVLGQSKR	9	BCEPRED
1254	1261	CKFDEDDS	8	BCEPRED
70	78	VSGTNGTKR	9	BCEPRED
250	257	TPGDSSSG	8	BCEPRED
278	287	KYNENGTITD	10	BCEPRED
437	448	NSNNLDSKVGGN	12	BCEPRED
525	532	CGPKKSTN	8	BCEPRED
568	578	DIADTTDAVRD	11	BCEPRED
673	684	SYQTQTNSPRRA	12	BCEPRED
743	751	CGDSTECSN	9	BCEPRED
771	781	AVEQDKNTQEV	11	BCEPRED
807	816	PDPSKPSKRS	10	BCEPRED
1157	1165	KNHTSPDVD	9	BCEPRED
144	160	YYHKNNKSWMESEFRVY	17	BCEPRED
185	196	NFKNLREFVFKN	12	BCEPRED
201	208	FKIYSKHT	8	BCEPRED
298	310	ETKCTLKSFTVEK	13	BCEPRED
351	361	YAWNRKRISNC	11	BCEPRED
402	409	IRGDEVRQ	8	BCEPRED
452	471	LYRLFRKSNLKPFERDISTE	20	BCEPRED
552	560	LTESNKKFL	9	BCEPRED
679	688	NSPRRARSVA	10	BCEPRED
809	820	PSKPSKRSFIED	12	BCEPRED
982	992	SRLDKVEAEVQ	11	BCEPRED
1013	1020	IRAAEIRA	8	BCEPRED
1082	1094	CHDGKAHFPREGV	13	BCEPRED
1144	1160	ELDSFKEELDKYFKNHT	17	BCEPRED
1179	1191	IQKEIDRLNEVAK	13	BCEPRED
1201	1211	QELGKYEQYIK	11	BCEPRED
1253	1264	CCKFDEDDSEPV	12	BCEPRED
134	141	QFCNDPFL	8	BCEPRED
145	152	YHKNNKSW	8	BCEPRED
160	168	YSSANNCTF	9	BCEPRED
435	444	AWNSNNLDSK	10	BCEPRED
538	546	CVNFNFNGL	9	BCEPRED
601	608	GTNTSNQV	8	BCEPRED
653	661	AEHVNNSYE	9	BCEPRED
706	713	AYSNNSIA	8	BCEPRED
1156	1163	FKNHTSPD	8	BCEPRED
597	608	VITPGTNTSNQV	12	BCPREDS
33	44	TRGVYYPDKVFR	12	BCPREDS
408	419	RQIAPGQTGKIA	12	BCPREDS
322	333	PTESIVRFPNIT	12	BCPREDS
1134	1145	NNTVYDPLQPEL	12	BCPREDS
250	261	TPGDSSSGWTAG	12	BCPREDS
93	104	ASTEKSNIIRGW	12	BCPREDS
1064	1075	HVTYVPAQEKNF	12	BCPREDS
1250	1261	CGSCCKFDEDDS	12	BCPREDS
50	61	STQDLFLPFFSN	12	BCPREDS
674	685	YQTQTNSPRRAR	12	BCPREDS
789	800	YKTPPIKDFGGF	12	BCPREDS
661	672	ECDIPIGAGICA	12	BCPREDS
1115	1126	ITTDNTFVSGNC	12	BCPREDS
15	26	CVNLTTRTQLPP	12	BCPREDS
540	551	NFNFNGLTGTGV	12	BCPREDS
832	843	GFIKQYGDCLGD	12	BCPREDS
378	389	KCYGVSPTKLND	12	BCPREDS
1147	1158	SFKEELDKYFKN	12	BCPREDS
143	154	VYYHKNNKSWME	12	BCPREDS
854	865	KFNGLTVLPPLL	12	BCPREDS
1206	1217	YEQYIKWPWYIW	12	BCPREDS
636	647	YSTGSNVFQTRA	12	BCPREDS
1002	1013	QSLQTYVTQQLI	12	BCPREDS
181	192	GKQGNFKNLREF	12	BCPREDS
263	274	AAYYVGYLQPRT	12	BCPREDS
107	118	GTTLDSKTQSLL	12	BCPREDS
754	765	LQYGSFCTQLNR	12	BCPREDS
74	85	NGTKRFDNPVLP	12	BCPREDS
421	432	YNYKLPDDFTGC	12	BCPREDS

772	783	VEQDKNTQEVFA	12	BCPREDS
807	818	PDPSKPSKRSFI	12	BCPREDS
882	893	ITSGWTFGAGAA	12	BCPREDS
475	486	AGSTPCNGVEGF	12	BCPREDS
519	530	HAPATVCGPKKS	12	BCPREDS
443	454	SKVGGNYNYLYR	12	BCPREDS
611	622	LYQDVNCTEVPV	12	BCPREDS
120	131	VNNATNVVIKVC	12	BCPREDS
297	308	SETKCTLKSFTV	12	BCPREDS
906	917	FNGIGVTQNVLY	12	BCPREDS
736	747	VDCTMYICGDST	12	BCPREDS
1097	1108	SNGTHWFVTQRN	12	BCPREDS
217	228	PQGFSALEPLVD	12	BCPREDS
700	711	GAENSVAYSNNS	12	BCPREDS
348	359	ASVYAWNRKRIS	12	BCPREDS
159	170	VYSSANNCTFEY	12	BCPREDS
502	513	GVGYQPYRVVVL	12	BCPREDS
553	564	TESNKKFLPFQQ	12	BCPREDS
1082	1093	CHDGKAHFPREG	12	BCPREDS
949	960	QDVVNQNAQALN	12	BCPREDS
194	205	FKNIDGYFKIYS	12	BCPREDS
1038	1049	KRVDFCGKGYHL	12	BCPREDS
715	726	PTNFTISVTTEI	12	BCPREDS
1067	1080	YVPAQEKNFTTAPA	14	BCPREDS
596	609	SVITPGTNTSNQVA	14	BCPREDS
14	27	QCVNLTTRTQLPPA	14	BCPREDS
114	127	TQSLLIVNNATNVV	14	BCPREDS
516	529	ELLHAPATVCGPKK	14	BCPREDS
1239	1252	SCCSCLKGCCSCGS	14	BCPREDS
410	423	IAPGQTGKIADYNY	14	BCPREDS
831	844	AGFIKQYGDCLGDI	14	BCPREDS
1142	1155	QPELDSFKEELDKY	14	BCPREDS
143	156	VYYHKNNKSWMESE	14	BCPREDS
653	666	AEHVNNSYECDIPI	14	BCPREDS
33	46	TRGVYYPDKVFRSS	14	BCPREDS
249	262	LTPGDSSSGWTAGA	14	BCPREDS
700	713	GAENSVAYSNNSIA	14	BCPREDS
321	334	QPTESIVRFPNITN	14	BCPREDS
374	387	FSTFKCYGVSPTKL	14	BCPREDS
345	358	TRFASVYAWNRKRI	14	BCPREDS
91	104	YFASTEKSNIIRGW	14	BCPREDS
790	803	KTPPIKDFGGFNFS	14	BCPREDS
435	448	AWNSNNLDSKVGGN	14	BCPREDS
1116	1129	TTDNTFVSGNCDVV	14	BCPREDS
1096	1109	VSNGTHWFVTQRNF	14	BCPREDS
999	1012	GRLQSLQTYVTQQL	14	BCPREDS
1165	1178	DLGDISGINASVVN	14	BCPREDS
1206	1219	YEQYIKWPWYIWLG	14	BCPREDS
949	962	QDVVNQNAQALNTL	14	BCPREDS
727	740	LPVSMTKTSVDCTM	14	BCPREDS
531	544	TNLVKNKCVNFNFN	14	BCPREDS
161	174	SSANNCTFEYVSQP	14	BCPREDS
668	681	AGICASYQTQTNSP	14	BCPREDS
1139	1154	DPLQPELDSFKEELDK	16	BCPREDS
246	261	RSYLTPGDSSSGWTAG	16	BCPREDS
674	689	YQTQTNSPRRARSVAS	16	BCPREDS
19	34	TTRTQLPPAYTNSFTR	16	BCPREDS
1112	1127	PQIITTDNTFVSGNCD	16	BCPREDS
1065	1080	VTYVPAQEKNFTTAPA	16	BCPREDS
1246	1261	GCCSCGSCCKFDEDDS	16	BCPREDS
598	613	ITPGTNTSNQVAVLYQ	16	BCPREDS
154	169	ESEFRVYSSANNCTFE	16	BCPREDS
322	337	PTESIVRFPNITNLCP	16	BCPREDS
501	516	NGVGYQPYRVVVLSFE	16	BCPREDS
630	645	TPTWRVYSTGSNVFQT	16	BCPREDS
1201	1216	QELGKYEQYIKWPWYI	16	BCPREDS
653	668	AEHVNNSYECDIPIGA	16	BCPREDS
88	103	DGVYFASTEKSNIIRG	16	BCPREDS
343	358	NATRFASVYAWNRKRI	16	BCPREDS
702	717	ENSVAYSNNSIAIPTN	16	BCPREDS
1090	1105	PREGVFVSNGTHWFVT	16	BCPREDS
596	613	SVITPGTNTSNQVAVLYQ	18	BCPREDS
25	42	PPAYTNSFTRGVYYPDKV	18	BCPREDS

671	688	CASYQTQTNSPRRARSVA	18	BCPREDS
1110	1127	YEPQIITTDNTFVSGNCD	18	BCPREDS
1137	1154	VYDPLQPELDSFKEELDK	18	BCPREDS
245	262	HRSYLTPGDSSSGWTAGA	18	BCPREDS
1200	1217	LQELGKYEQYIKWPWYIW	18	BCPREDS
406	423	EVRQIAPGQTGKIADYNY	18	BCPREDS
1063	1080	LHVTYVPAQEKNFTTAPA	18	BCPREDS
495	512	YGFQPTNGVGYQPYRVVV	18	BCPREDS
700	717	GAENSVAYSNNSIAIPTN	18	BCPREDS
1244	1261	LKGCCSCGSCCKFDEDDS	18	BCPREDS
652	669	GAEHVNNSYECDIPIGAG	18	BCPREDS
630	647	TPTWRVYSTGSNVFQTRA	18	BCPREDS
369	386	YNSASFSTFKCYGVSPTK	18	BCPREDS
143	160	VYYHKNNKSWMESEFRVY	18	BCPREDS
776	793	KNTQEVFAQVKQIYKTPP	18	BCPREDS
346	363	RFASVYAWNRKRISNCVA	18	BCPREDS
472	489	IYQAGSTPCNGVEGFNCY	18	BCPREDS
93	110	ASTEKSNIIRGWIFGTTL	18	BCPREDS
314	331	QTSNFRVQPTESIVRFPN	18	BCPREDS
426	443	PDDFTGCVIAWNSNNLDS	18	BCPREDS
519	536	HAPATVCGPKKSTNLVKN	18	BCPREDS
112	129	SKTQSLLIVNNATNVVIK	18	BCPREDS
796	813	DFGGFNFSQILPDPSKPS	18	BCPREDS
904	921	YRFNGIGVTQNVLYENQK	18	BCPREDS
829	846	ADAGFIKQYGDCLGDIAA	18	BCPREDS
1167	1184	GDISGINASVVNIQKEID	18	BCPREDS
71	88	SGTNGTKRFDNPVLPFND	18	BCPREDS
672	691	ASYQTQTNSPRRARSVASQS	20	BCPREDS
19	38	TTRTQLPPAYTNSFTRGVYY	20	BCPREDS
1135	1154	NTVYDPLQPELDSFKEELDK	20	BCPREDS
589	608	PCSFGGVSVITPGTNTSNQV	20	BCPREDS
1063	1082	LHVTYVPAQEKNFTTAPAIC	20	BCPREDS
114	133	TQSLLIVNNATNVVIKVCEF	20	BCPREDS
1108	1127	NFYEPQIITTDNTFVSGNCD	20	BCPREDS
700	719	GAENSVAYSNNSIAIPTNFT	20	BCPREDS
782	801	FAQVKQIYKTPPIKDFGGFN	20	BCPREDS
93	112	ASTEKSNIIRGWIFGTTLDS	20	BCPREDS
408	427	RQIAPGQTGKIADYNYKLPD	20	BCPREDS
243	262	ALHRSYLTPGDSSSGWTAGA	20	BCPREDS
1200	1219	LQELGKYEQYIKWPWYIWLG	20	BCPREDS
148	167	NNKSWMESEFRVYSSANNCT	20	BCPREDS
311	330	GIYQTSNFRVQPTESIVRFP	20	BCPREDS
493	512	QSYGFQPTNGVGYQPYRVVV	20	BCPREDS
627	646	DQLTPTWRVYSTGSNVFQTR	20	BCPREDS
1165	1184	DLGDISGINASVVNIQKEID	20	BCPREDS
651	670	IGAEHVNNSYECDIPIGAGI	20	BCPREDS
723	742	TTEILPVSMTKTSVDCTMYI	20	BCPREDS
472	491	IYQAGSTPCNGVEGFNCYFP	20	BCPREDS
519	538	HAPATVCGPKKSTNLVKNKC	20	BCPREDS
19	40	TTRTQLPPAYTNSFTRGVYYPD	22	BCPREDS
596	617	SVITPGTNTSNQVAVLYQDVNC	22	BCPREDS
410	431	IAPGQTGKIADYNYKLPDDFTG	22	BCPREDS
1240	1261	CCSCLKGCCSCGSCCKFDEDDS	22	BCPREDS
1133	1154	VNNTVYDPLQPELDSFKEELDK	22	BCPREDS
664	685	IPIGAGICASYQTQTNSPRRAR	22	BCPREDS
93	114	ASTEKSNIIRGWIFGTTLDSKT	22	BCPREDS
1064	1085	HVTYVPAQEKNFTTAPAICHDG	22	BCPREDS
242	263	LALHRSYLTPGDSSSGWTAGAA	22	BCPREDS
1106	1127	QRNFYEPQIITTDNTFVSGNCD	22	BCPREDS
772	793	VEQDKNTQEVFAQVKQIYKTPP	22	BCPREDS
64	85	WFHAIHVSGTNGTKRFDNPVLP	22	BCPREDS
625	646	HADQLTPTWRVYSTGSNVFQTR	22	BCPREDS
1174	1195	ASVVNIQKEIDRLNEVAKNLNE	22	BCPREDS
139	160	PFLGVYYHKNNKSWMESEFRVY	22	BCPREDS
691	712	SIIAYTMSLGAENSVAYSNNSI	22	BCPREDS
380	401	YGVSPTKLNDLCFTNVYADSFV	22	BCPREDS
560	581	LPFQQFGRDIADTTDAVRDPQT	22	BCPREDS
473	494	YQAGSTPCNGVEGFNCYFPLQS	22	BCPREDS
313	334	YQTSNFRVQPTESIVRFPNITN	22	BCPREDS
899	920	AMQMAYRFNGIGVTQNVLYENQ	22	BCPREDS
1198	1219	IDLQELGKYEQYIKWPWYIWLG	22	BCPREDS
530	551	STNLVKNKCVNFNFNGLTGTGV	22	BCPREDS
270	291	LQPRTFLLKYNENGTITDAVDC	22	BCPREDS

950	966	DVVNQNAQALNTLVKQL	17	COVIDep
988	1004	EAEVQIDRLITGRLQSL	17	COVIDep
1182	1209	EIDRLNEVAKNLNESLIDLQELGKYEQY	28	COVIDep
1188	1204	EVAKNLNESLIDLQELG	17	COVIDep
338	345	FGEVFNAT	8	COVIDep
817	833	FIEDLLFNKVTLADAGF	17	COVIDep
891	907	GAALQIPFAMQMAYRFN	17	COVIDep
667	674	GAGICASY	8	COVIDep
972	988	AISSVLNDILSRLDKVE	17	COVIDep
757	764	GSFCTQLN	8	COVIDep
980	996	ILSRLDKVEAEVQIDRL	17	COVIDep
899	906	AMQMAYRF	8	COVIDep
1157	1173	KNHTSPDVDLGDISGIN	17	COVIDep
902	918	MAYRFNGIGVTQNVLYE	17	COVIDep
1025	1041	AATKMSECVLGQSKRVD	17	COVIDep
897	913	PFAMQMAYRFNGIGVTQ	17	COVIDep
957	973	QALNTLVKQLSSNFGAI	17	COVIDep
1011	1027	QLIRAAEIRASANLAAT	17	COVIDep
563	568	QQFGRD	6	COVIDep
1019	1035	RASANLAATKMSECVLG	17	COVIDep
995	1011	RLITGRLQSLQTYVTQQ	17	COVIDep
1003	1019	SLQTYVTQQLIRAAEIR	17	COVIDep
1254	1273	CKFDEDDSEPVLKGVKLHYT	20	COVIDep
1136	1152	TVYDPLQPELDSFKEEL	17	COVIDep
327	343	VRFPNITNLCPFGEVFN	17	COVIDep
1259	1273	DDSEPVLKGVKLHYT	15	COVIDep
1165	1181	DLGDISGINASVVNIQK	17	COVIDep
56	195	FFSNVTWFHFDNPVLPFNDGVYFASTNIIRGWIFGTTLDSKTQSLLIVNNATNVVIKVCEFQFCNDPFLGEFRVYSSANNCTFEYVSQPFLKNLREFVF	103	ellipro
393	514	TNVYADSFVIRGDEVRQIAPGQTGKIADYNYKLPDDFTGCVIAWNSNNLDSYNYLYRPLQSYGFQPTVGYQPYRVVVLSF	80	ellipro
1069	1146	PAQEKNFTTAPAICHDGKAHFPREGVFVSNGTHWFVTQRNFYEPQIITTDNTFVSGNCDVVIGIVNNTVYDPLQPELD	78	ellipro
201	269	FKIYSKHTPINLVRDLPQGFSALEPLVDLPIGINITRFQTLLALHRGAAAYYVGYL	56	ellipro
327	387	VRLCPFGEVFNATRFASVYAWNRKRISNCVADYSVLYNSASFSTFKCYGVSPTKLN	56	ellipro
27	37	AYTNSFTRGVY	11	ellipro
701	719	AENSVAYSNNSIAIPTNFT	19	ellipro
789	805	YKTPPIKDFGGFNFSQI	17	ellipro
687	691	VASQS	5	ellipro
577	584	RDPQTLEI	8	ellipro
892	900	AALQIPFAM	9	ellipro
653	659	AEHVNNS	7	ellipro
554	563	ESNKKFLPFQ	10	ellipro
807	814	PDPSKR	6	ellipro
522	537	ATVCGPKKSTNLVKNK	16	ellipro
617	641	CTEVSNV	7	ellipro
56	194	FFSNVTWFHNPVLPFNDGVYFASTEKSNIIRGWIFGTTLDSKTQSLLIVNNATNVVIKVCEFQFCNDPFLSEFRVYSSANNCTFEYVSQPFLKNLREFV	103	ellipro
201	269	FKIYSKHTPPQGFSALEPLVDLPIGINITRFQTLLAAYYVGYL	43	ellipro
436	459	WNSNNLDSYNYLYRN	15	ellipro
464	510	FERDISTEIYNCYFPLQSYGFQPTVGYQPYRVV	33	ellipro
577	585	RDPQTLEIL	9	ellipro
700	719	GAENSVAYSNNSIAIPTNFT	20	ellipro
322	375	PTESIVRFPNITNLCPFGEVFNATRFASVYAWNRKRISNCVADYSVLYNSASFS	54	ellipro
520	537	APATVCGPKKSTNLVKNK	18	ellipro
553	564	TESNKKFLPFQQ	12	ellipro
672	693	AVASQSIIA	9	ellipro
652	661	GAEHVNNSYE	10	ellipro
892	903	AALQIPFAMQMA	12	ellipro
616	642	NCTEVTGSNVF	11	ellipro
603	608	NTSNQV	6	ellipro
281	286	ENGTIT	6	ellipro
56	195	LPFFSNVTWFHDNPVLPFNDGVYFASTNIIRGWIFGTTLDSKTQSLLIVNNATNVVIKVCEFQFCNDPFFRVYSSANNCTFEYVSQPFLKNLREFVFKN	99	ellipro
1070	1146	AQEKNFTTAPAICHDGKAHFPREGVFVSNGTHWFVTQRNFYEPQIITTDNTFVSGNCDVVIGIVNNTVYDPLQPELD	77	ellipro
198	269	DGYFKIYSKHTPINLVRDLPQGFSALEPLVDLPIGINITRFQTLLALHGAAAYYVGYL	58	ellipro
465	508	ERDISTENCYFPLQSYGFQVGYQPYR	26	ellipro
327	367	VRFPNITNLCPFGEVFNATRFASVYAWNRKRISNCVADYSV	41	ellipro
526	537	GPKKSTNLVKNK	12	ellipro
789	814	YKTPPIKDFGGFNFSQILPDPSKR	24	ellipro
687	693	VASQSII	7	ellipro
652	659	GAEHVNNS	8	ellipro
553	563	TESNKKFLPFQ	11	ellipro
370	374	NSASF	5	ellipro
280	286	NENGTIT	7	ellipro
617	641	CTEVNV	6	ellipro
598	609	ITPGTNTSNQVA	12	FBCPred
1114	1125	IITTDNTFVSGN	12	FBCPred

1252	1263	SCCKFDEDDSEP	12	FBCPred
880	891	GTITSGWTFGAG	12	FBCPred
676	687	TQTNSPRRARSV	12	FBCPred
1140	1151	PLQPELDSFKEE	12	FBCPred
858	869	LTVLPPLLTDEM	12	FBCPred
700	711	GAENSVAYSNNS	12	FBCPred
981	992	LSRLDKVEAEVQ	12	FBCPred
250	261	TPGDSSSGWTAG	12	FBCPred
159	170	VYSSANNCTFEY	12	FBCPred
15	26	CVNLTTRTQLPP	12	FBCPred
1206	1217	YEQYIKWPWYIW	12	FBCPred
29	40	TNSFTRGVYYPD	12	FBCPred
119	130	IVNNATNVVIKV	12	FBCPred
294	305	DPLSETKCTLKS	12	FBCPred
279	290	YNENGTITDAVD	12	FBCPred
1064	1075	HVTYVPAQEKNF	12	FBCPred
217	228	PQGFSALEPLVD	12	FBCPred
430	441	TGCVIAWNSNNL	12	FBCPred
345	356	TRFASVYAWNRK	12	FBCPred
835	846	KQYGDCLGDIAA	12	FBCPred
71	82	SGTNGTKRFDNP	12	FBCPred
1238	1249	TSCCSCLKGCCS	12	FBCPred
364	375	DYSVLYNSASFS	12	FBCPred
612	623	YQDVNCTEVPVA	12	FBCPred
389	400	DLCFTNVYADSF	12	FBCPred
719	730	TISVTTEILPVS	12	FBCPred
626	637	ADQLTPTWRVYS	12	FBCPred
655	666	HVNNSYECDIPI	12	FBCPred
323	334	TESIVRFPNITN	12	FBCPred
904	915	YRFNGIGVTQNV	12	FBCPred
145	156	YHKNNKSWMESE	12	FBCPred
502	513	GVGYQPYRVVVL	12	FBCPred
1097	1108	SNGTHWFVTQRN	12	FBCPred
474	485	QAGSTPCNGVEG	12	FBCPred
1162	1173	PDVDLGDISGIN	12	FBCPred
408	419	RQIAPGQTGKIA	12	FBCPred
568	579	DIADTTDAVRDP	12	FBCPred
309	320	EKGIYQTSNFRV	12	FBCPred
445	456	VGGNYNYLYRLF	12	FBCPred
533	544	LVKNKCVNFNFN	12	FBCPred
1050	1061	MSFPQSAPHGVV	12	FBCPred
779	790	QEVFAQVKQIYK	12	FBCPred
1192	1203	NLNESLIDLQEL	12	FBCPred
597	610	VITPGTNTSNQVAV	14	FBCPred
1114	1127	IITTDNTFVSGNCD	14	FBCPred
1141	1154	LQPELDSFKEELDK	14	FBCPred
1250	1263	CGSCCKFDEDDSEP	14	FBCPred
676	689	TQTNSPRRARSVAS	14	FBCPred
879	892	AGTITSGWTFGAGA	14	FBCPred
698	711	SLGAENSVAYSNNS	14	FBCPred
1205	1218	KYEQYIKWPWYIWL	14	FBCPred
27	40	AYTNSFTRGVYYPD	14	FBCPred
246	259	RSYLTPGDSSSGWT	14	FBCPred
859	872	TVLPPLLTDEMIAQ	14	FBCPred
981	994	LSRLDKVEAEVQID	14	FBCPred
159	172	VYSSANNCTFEYVS	14	FBCPred
1067	1080	YVPAQEKNFTTAPA	14	FBCPred
71	84	SGTNGTKRFDNPVL	14	FBCPred
345	358	TRFASVYAWNRKRI	14	FBCPred
9	22	PLVSSQCVNLTTRT	14	FBCPred
294	307	DPLSETKCTLKSFT	14	FBCPred
831	844	AGFIKQYGDCLGDI	14	FBCPred
279	292	YNENGTITDAVDCA	14	FBCPred
427	440	DDFTGCVIAWNSNN	14	FBCPred
323	336	TESIVRFPNITNLC	14	FBCPred
1235	1248	CCMTSCCSCLKGCC	14	FBCPred
215	228	DLPQGFSALEPLVD	14	FBCPred
626	639	ADQLTPTWRVYSTG	14	FBCPred
362	375	VADYSVLYNSASFS	14	FBCPred
388	401	NDLCFTNVYADSFV	14	FBCPred
716	729	TNFTISVTTEILPV	14	FBCPred
907	920	NGIGVTQNVLYENQ	14	FBCPred
118	131	LIVNNATNVVIKVC	14	FBCPred

1158	1171	NHTSPDVDLGDISG	14	FBCPred
653	666	AEHVNNSYECDIPI	14	FBCPred
446	459	GGNYNYLYRLFRKS	14	FBCPred
500	513	TNGVGYQPYRVVVL	14	FBCPred
1097	1110	SNGTHWFVTQRNFY	14	FBCPred
143	156	VYYHKNNKSWMESE	14	FBCPred
1190	1203	AKNLNESLIDLQEL	14	FBCPred
568	581	DIADTTDAVRDPQT	14	FBCPred
404	417	GDEVRQIAPGQTGK	14	FBCPred
472	485	IYQAGSTPCNGVEG	14	FBCPred
731	744	MTKTSVDCTMYICG	14	FBCPred
533	546	LVKNKCVNFNFNGL	14	FBCPred
775	788	DKNTQEVFAQVKQI	14	FBCPred
597	612	VITPGTNTSNQVAVLY	16	FBCPred
1114	1129	IITTDNTFVSGNCDVV	16	FBCPred
1140	1155	PLQPELDSFKEELDKY	16	FBCPred
1248	1263	CSCGSCCKFDEDDSEP	16	FBCPred
25	40	PPAYTNSFTRGVYYPD	16	FBCPred
676	691	TQTNSPRRARSVASQS	16	FBCPred
696	711	TMSLGAENSVAYSNNS	16	FBCPred
1199	1214	DLQELGKYEQYIKWPW	16	FBCPred
154	169	ESEFRVYSSANNCTFE	16	FBCPred
1065	1080	VTYVPAQEKNFTTAPA	16	FBCPred
246	261	RSYLTPGDSSSGWTAG	16	FBCPred
426	441	PDDFTGCVIAWNSNNL	16	FBCPred
343	358	NATRFASVYAWNRKRI	16	FBCPred
69	84	HVSGTNGTKRFDNPVL	16	FBCPred
981	996	LSRLDKVEAEVQIDRL	16	FBCPred
322	337	PTESIVRFPNITNLCP	16	FBCPred
859	874	TVLPPLLTDEMIAQYT	16	FBCPred
831	846	AGFIKQYGDCLGDIAA	16	FBCPred
292	307	ALDPLSETKCTLKSFT	16	FBCPred
393	408	TNVYADSFVIRGDEVR	16	FBCPred
626	641	ADQLTPTWRVYSTGSN	16	FBCPred
271	286	QPRTFLLKYNENGTIT	16	FBCPred
715	730	PTNFTISVTTEILPVS	16	FBCPred
1165	1180	DLGDISGINASVVNIQ	16	FBCPred
360	375	NCVADYSVLYNSASFS	16	FBCPred
213	228	VRDLPQGFSALEPLVD	16	FBCPred
498	513	QPTNGVGYQPYRVVVL	16	FBCPred
8	23	LPLVSSQCVNLTTRTQ	16	FBCPred
905	920	RFNGIGVTQNVLYENQ	16	FBCPred
651	666	IGAEHVNNSYECDIPI	16	FBCPred
568	583	DIADTTDAVRDPQTLE	16	FBCPred
1231	1246	TIMLCCMTSCCSCLKG	16	FBCPred
1097	1112	SNGTHWFVTQRNFYEP	16	FBCPred
877	892	LLAGTITSGWTFGAGA	16	FBCPred
444	459	KVGGNYNYLYRLFRKS	16	FBCPred
119	134	IVNNATNVVIKVCEFQ	16	FBCPred
476	491	GSTPCNGVEGFNCYFP	16	FBCPred
775	790	DKNTQEVFAQVKQIYK	16	FBCPred
597	614	VITPGTNTSNQVAVLYQD	18	FBCPred
1246	1263	GCCSCGSCCKFDEDDSEP	18	FBCPred
1110	1127	YEPQIITTDNTFVSGNCD	18	FBCPred
1137	1154	VYDPLQPELDSFKEELDK	18	FBCPred
25	42	PPAYTNSFTRGVYYPDKV	18	FBCPred
1200	1217	LQELGKYEQYIKWPWYIW	18	FBCPred
695	712	YTMSLGAENSVAYSNNSI	18	FBCPred
669	686	GICASYQTQTNSPRRARS	18	FBCPred
154	171	ESEFRVYSSANNCTFEYV	18	FBCPred
427	444	DDFTGCVIAWNSNNLDSK	18	FBCPred
1063	1080	LHVTYVPAQEKNFTTAPA	18	FBCPred
71	88	SGTNGTKRFDNPVLPFND	18	FBCPred
244	261	LHRSYLTPGDSSSGWTAG	18	FBCPred
289	306	VDCALDPLSETKCTLKSF	18	FBCPred
342	359	FNATRFASVYAWNRKRIS	18	FBCPred
722	739	VTTEILPVSMTKTSVDCT	18	FBCPred
391	408	CFTNVYADSFVIRGDEVR	18	FBCPred
269	286	YLQPRTFLLKYNENGTIT	18	FBCPred
322	339	PTESIVRFPNITNLCPFG	18	FBCPred
496	513	GFQPTNGVGYQPYRVVVL	18	FBCPred
625	642	HADQLTPTWRVYSTGSNV	18	FBCPred
368	385	LYNSASFSTFKCYGVSPT	18	FBCPred

859	876	TVLPPLLTDEMIAQYTSA	18	FBCPred
978	995	NDILSRLDKVEAEVQIDR	18	FBCPred
829	846	ADAGFIKQYGDCLGDIAA	18	FBCPred
211	228	NLVRDLPQGFSALEPLVD	18	FBCPred
1163	1180	DVDLGDISGINASVVNIQ	18	FBCPred
903	920	AYRFNGIGVTQNVLYENQ	18	FBCPred
566	583	GRDIADTTDAVRDPQTLE	18	FBCPred
476	493	GSTPCNGVEGFNCYFPLQ	18	FBCPred
119	136	IVNNATNVVIKVCEFQFC	18	FBCPred
773	790	EQDKNTQEVFAQVKQIYK	18	FBCPred
649	666	CLIGAEHVNNSYECDIPI	18	FBCPred
596	615	SVITPGTNTSNQVAVLYQDV	20	FBCPred
1110	1129	YEPQIITTDNTFVSGNCDVV	20	FBCPred
1244	1263	LKGCCSCGSCCKFDEDDSEP	20	FBCPred
1135	1154	NTVYDPLQPELDSFKEELDK	20	FBCPred
23	42	QLPPAYTNSFTRGVYYPDKV	20	FBCPred
1199	1218	DLQELGKYEQYIKWPWYIWL	20	FBCPred
667	686	GAGICASYQTQTNSPRRARS	20	FBCPred
694	713	AYTMSLGAENSVAYSNNSIA	20	FBCPred
154	173	ESEFRVYSSANNCTFEYVSQ	20	FBCPred
425	444	LPDDFTGCVIAWNSNNLDSK	20	FBCPred
69	88	HVSGTNGTKRFDNPVLPFND	20	FBCPred
366	385	SVLYNSASFSTFKCYGVSPT	20	FBCPred
287	306	DAVDCALDPLSETKCTLKSF	20	FBCPred
343	362	NATRFASVYAWNRKRISNCV	20	FBCPred
720	739	ISVTTEILPVSMTKTSVDCT	20	FBCPred
1064	1083	HVTYVPAQEKNFTTAPAICH	20	FBCPred
494	513	SYGFQPTNGVGYQPYRVVVL	20	FBCPred
389	408	DLCFTNVYADSFVIRGDEVR	20	FBCPred
243	262	ALHRSYLTPGDSSSGWTAGA	20	FBCPred
623	642	AIHADQLTPTWRVYSTGSNV	20	FBCPred
322	341	PTESIVRFPNITNLCPFGEV	20	FBCPred
859	878	TVLPPLLTDEMIAQYTSALL	20	FBCPred
1162	1181	PDVDLGDISGINASVVNIQK	20	FBCPred
209	228	PINLVRDLPQGFSALEPLVD	20	FBCPred
901	920	QMAYRFNGIGVTQNVLYENQ	20	FBCPred
829	848	ADAGFIKQYGDCLGDIAARD	20	FBCPred
974	993	SSVLNDILSRLDKVEAEVQI	20	FBCPred
473	492	YQAGSTPCNGVEGFNCYFPL	20	FBCPred
564	583	QFGRDIADTTDAVRDPQTLE	20	FBCPred
774	793	QDKNTQEVFAQVKQIYKTPP	20	FBCPred
119	138	IVNNATNVVIKVCEFQFCND	20	FBCPred
598	619	ITPGTNTSNQVAVLYQDVNCTE	22	FBCPred
1242	1263	SCLKGCCSCGSCCKFDEDDSEP	22	FBCPred
1108	1129	NFYEPQIITTDNTFVSGNCDVV	22	FBCPred
1134	1155	NNTVYDPLQPELDSFKEELDKY	22	FBCPred
19	40	TTRTQLPPAYTNSFTRGVYYPD	22	FBCPred
667	688	GAGICASYQTQTNSPRRARSVA	22	FBCPred
1199	1220	DLQELGKYEQYIKWPWYIWLGF	22	FBCPred
148	169	NNKSWMESEFRVYSSANNCTFE	22	FBCPred
423	444	YKLPDDFTGCVIAWNSNNLDSK	22	FBCPred
696	717	TMSLGAENSVAYSNNSIAIPTN	22	FBCPred
282	303	NGTITDAVDCALDPLSETKCTL	22	FBCPred
364	385	DYSVLYNSASFSTFKCYGVSPT	22	FBCPred
71	92	SGTNGTKRFDNPVLPFNDGVYF	22	FBCPred
720	741	ISVTTEILPVSMTKTSVDCTMY	22	FBCPred
1064	1085	HVTYVPAQEKNFTTAPAICHDG	22	FBCPred
388	409	NDLCFTNVYADSFVIRGDEVRQ	22	FBCPred
492	513	LQSYGFQPTNGVGYQPYRVVVL	22	FBCPred
316	337	SNFRVQPTESIVRFPNITNLCP	22	FBCPred
1159	1180	HTSPDVDLGDISGINASVVNIQ	22	FBCPred
626	647	ADQLTPTWRVYSTGSNVFQTRA	22	FBCPred
341	362	VFNATRFASVYAWNRKRISNCV	22	FBCPred
899	920	AMQMAYRFNGIGVTQNVLYENQ	22	FBCPred
207	228	HTPINLVRDLPQGFSALEPLVD	22	FBCPred
972	993	AISSVLNDILSRLDKVEAEVQI	22	FBCPred
568	589	DIADTTDAVRDPQTLEILDITP	22	FBCPred
21	31	RTQLPPAYTNS	11	IEDB
71	81	SGTNGTKRFDN	11	IEDB
181	186	GKQGNF	6	IEDB
249	261	LTPGDSSSGWTAG	13	IEDB
282	287	NGTITD	6	IEDB
318	324	FRVQPTE	7	IEDB

407	420	VRQIAPGQTGKIAD	14	IEDB
423	428	YKLPDD	6	IEDB
439	447	NNLDSKVGG	9	IEDB
473	483	YQAGSTPCNGV	11	IEDB
495	506	YGFQPTNGVGYQ	12	IEDB
523	532	TVCGPKKSTN	10	IEDB
567	580	RDIADTTDAVRDPQ	14	IEDB
597	606	VITPGTNTSN	10	IEDB
675	687	QTQTNSPRRARSV	13	IEDB
772	780	VEQDKNTQE	9	IEDB
788	797	IYKTPPIKDF	10	IEDB
805	816	ILPDPSKPSKRS	12	IEDB
936	941	DSLSST	6	IEDB
1069	1077	PAQEKNFTT	9	IEDB
1137	1148	VYDPLQPELDSF	12	IEDB
1157	1167	KNHTSPDVDLG	11	IEDB
1256	1265	FDEDDSEPVL	10	IEDB
13	37	SQCVNLTTRTQLPPAYTNSFTRGVY	25	IEDB
59	81	FSNVTWFHAIHVSGTNGTKRFDN	23	IEDB
138	154	DPFLGVYYHKNNKSWME	17	IEDB
177	189	MDLEGKQGNFKNL	13	IEDB
206	221	KHTPINLVRDLPQGFS	16	IEDB
250	260	TPGDSSSGWTA	11	IEDB
304	322	KSFTVEKGIYQTSNFRVQP	19	IEDB
329	363	FPNITNLCPFGEVFNATRFASVYAWNRKRISNCVA	35	IEDB
369	393	YNSASFSTFKCYGVSPTKLNDLCFT	25	IEDB
404	426	GDEVRQIAPGQTGKIADYNYKLP	23	IEDB
440	501	NLDSKVGGNYNYLYRLFRKSNLKPFERDISTEIYQAGSTPCNGVEGFNCYFPLQSYGFQPTN	62	IEDB
516	536	ELLHAPATVCGPKKSTNLVKN	21	IEDB
555	562	SNKKFLPF	8	IEDB
616	632	NCTEVPVAIHADQLTPT	17	IEDB
634	644	RVYSTGSNVFQ	11	IEDB
656	666	VNNSYECDIPI	11	IEDB
672	690	ASYQTQTNSPRRARSVASQ	19	IEDB
695	710	YTMSLGAENSVAYSNN	16	IEDB
773	779	EQDKNTQ	7	IEDB
786	800	KQIYKTPPIKDFGGF	15	IEDB
807	814	PDPSKPSK	8	IEDB
828	842	LADAGFIKQYGDCLG	15	IEDB
1035	1043	GQSKRVDFC	9	IEDB
1107	1118	RNFYEPQIITTD	12	IEDB
1133	1172	VNNTVYDPLQPELDSFKEELDKYFKNHTSPDVDLGDISGI	40	IEDB
1252	1267	SCCKFDEDDSEPVLKG	16	IEDB
4	18	FLVLLPLVSSQCVNL	15	IEDB
34	41	RGVYYPDK	8	IEDB
44	51	RSSVLHST	8	IEDB
53	60	DLFLPFFS	8	IEDB
65	70	FHAIHV	6	IEDB
81	87	NPVLPFN	7	IEDB
115	121	QSLLIVN	7	IEDB
125	134	NVVIKVCEFQ	10	IEDB
136	146	CNDPFLGVYYH	11	IEDB
168	174	FEYVSQP	7	IEDB
210	216	INLVRDL	7	IEDB
223	230	LEPLVDLP	8	IEDB
239	248	QTLLALHRSY	10	IEDB
263	270	AAYYVGYL	8	IEDB
272	278	PRTFLLK	7	IEDB
288	295	AVDCALDP	8	IEDB
333	339	TNLCPFG	7	IEDB
359	371	SNCVADYSVLYNS	13	IEDB
376	385	TFKCYGVSPT	10	IEDB
430	435	TGCVIA	6	IEDB
488	495	CYFPLQSY	8	IEDB
505	527	YQPYRVVVLSFELLHAPATVCGP	23	IEDB
592	599	FGGVSVIT	8	IEDB
607	615	QVAVLYQDV	9	IEDB
617	627	CTEVPVAIHAD	11	IEDB
647	653	AGCLIGA	7	IEDB
667	674	GAGICASY	8	IEDB
687	693	VASQSII	7	IEDB
723	730	TTEILPVS	8	IEDB
735	741	SVDCTMY	7	IEDB

750	763	SNLLLQYGSFCTQL	14	IEDB
781	788	VFAQVKQI	8	IEDB
803	808	SQILPD	6	IEDB
837	843	YGDCLGD	7	IEDB
847	853	RDLICAQ	7	IEDB
858	864	LTVLPPL	7	IEDB
873	880	YTSALLAG	8	IEDB
959	966	LNTLVKQL	8	IEDB
973	979	ISSVLND	7	IEDB
1002	1011	QSLQTYVTQQ	10	IEDB
1030	1037	SECVLGQS	8	IEDB
1057	1070	PHGVVFLHVTYVPA	14	IEDB
1079	1085	PAICHDG	7	IEDB
1123	1132	SGNCDVVIGI	10	IEDB
1174	1179	ASVVNI	6	IEDB
1221	1256	IAGLIAIVMVTIMLCCMTSCCSCLKGCCSCGSCCKF	36	IEDB
1262	1270	EPVLKGVKL	9	IEDB
14	20	QCVNLTT	7	IEDB
28	33	YTNSFT	6	IEDB
47	52	VLHSTQ	6	IEDB
70	80	VSGTNGTKRFD	11	IEDB
94	100	STEKSNI	7	IEDB
109	115	TLDSKTQ	7	IEDB
145	153	YHKNNKSWM	9	IEDB
161	167	SSANNCT	7	IEDB
180	188	EGKQGNFKN	9	IEDB
248	263	YLTPGDSSSGWTAGAA	16	IEDB
280	292	NENGTITDAVDCA	13	IEDB
295	302	PLSETKCT	8	IEDB
316	325	SNFRVQPTES	10	IEDB
356	364	KRISNCVAD	9	IEDB
381	388	GVSPTKLN	8	IEDB
404	409	GDEVRQ	6	IEDB
411	425	APGQTGKIADYNYKL	15	IEDB
437	449	NSNNLDSKVGGNY	13	IEDB
464	470	FERDIST	7	IEDB
472	485	IYQAGSTPCNGVEG	14	IEDB
496	507	GFQPTNGVGYQP	12	IEDB
522	535	ATVCGPKKSTNLVK	14	IEDB
550	557	GVLTESNK	8	IEDB
565	581	FGRDIADTTDAVRDPQT	17	IEDB
599	607	TPGTNTSNQ	9	IEDB
614	620	DVNCTEV	7	IEDB
637	647	STGSNVFQTRA	11	IEDB
654	662	EHVNNSYEC	9	IEDB
672	689	ASYQTQTNSPRRARSVAS	18	IEDB
699	712	LGAENSVAYSNNSI	14	IEDB
731	738	MTKTSVDC	8	IEDB
743	750	CGDSTECS	8	IEDB
771	781	AVEQDKNTQEV	11	IEDB
806	814	LPDPSKPSK	9	IEDB
837	842	YGDCLG	6	IEDB
926	944	QFNSAIGKIQDSLSSTASA	19	IEDB
949	958	QDVVNQNAQA	10	IEDB
966	972	LSSNFGA	7	IEDB
1033	1046	VLGQSKRVDFCGKG	14	IEDB
1053	1058	PQSAPH	6	IEDB
1081	1091	ICHDGKAHFPR	11	IEDB
1114	1128	IITTDNTFVSGNCDV	15	IEDB
1141	1154	LQPELDSFKEELDK	14	IEDB
1156	1168	FKNHTSPDVDLGD	13	IEDB
1187	1194	NEVAKNLN	8	IEDB
1238	1264	TSCCSCLKGCCSCGSCCKFDEDDSEPV	27	IEDB
18	32	LTTRTQLPPAYTNSF	15	IEDB
35	43	GVYYPDKVF	9	IEDB
73	80	TNGTKRFD	8	IEDB
110	115	LDSKTQ	6	IEDB
144	153	YYHKNNKSWM	10	IEDB
179	185	LEGKQGN	7	IEDB
202	208	KIYSKHT	7	IEDB
250	255	TPGDSS	6	IEDB
278	284	KYNENGT	7	IEDB
314	323	QTSNFRVQPT	10	IEDB

352	357	AWNRKR	6	IEDB
419	428	ADYNYKLPDD	10	IEDB
437	442	NSNNLD	6	IEDB
455	468	LFRKSNLKPFERDI	14	IEDB
495	500	YGFQPT	6	IEDB
569	581	IADTTDAVRDPQT	13	IEDB
601	606	GTNTSN	6	IEDB
627	636	DQLTPTWRVY	10	IEDB
655	660	HVNNSY	6	IEDB
674	685	YQTQTNSPRRAR	12	IEDB
786	794	KQIYKTPPI	9	IEDB
808	817	DPSKPSKRSF	10	IEDB
914	920	NVLYENQ	7	IEDB
1068	1076	VPAQEKNFT	9	IEDB
1105	1111	TQRNFYE	7	IEDB
1139	1162	DPLQPELDSFKEELDKYFKNHTSP	24	IEDB
1179	1186	IQKEIDRL	8	IEDB
1202	1210	ELGKYEQYI	9	IEDB
1256	1261	FDEDDS	6	IEDB
23	34	QLPPAYTNSFTR	12	IEDB
36	43	VYYPDKVF	8	IEDB
71	91	SGTNGTKRFDNPVLPFNDGVY	21	IEDB
135	142	FCNDPFLG	8	IEDB
145	152	YHKNNKSW	8	IEDB
181	188	GKQGNFKN	8	IEDB
248	260	YLTPGDSSSGWTA	13	IEDB
279	285	YNENGTI	7	IEDB
292	298	ALDPLSE	7	IEDB
313	320	YQTSNFRV	8	IEDB
367	374	VLYNSASF	8	IEDB
380	389	YGVSPTKLND	10	IEDB
411	430	APGQTGKIADYNYKLPDDFT	20	IEDB
436	452	WNSNNLDSKVGGNYNYL	17	IEDB
474	507	QAGSTPCNGVEGFNCYFPLQSYGFQPTNGVGYQP	34	IEDB
523	535	TVCGPKKSTNLVK	13	IEDB
537	548	KCVNFNFNGLTG	12	IEDB
588	594	TPCSFGG	7	IEDB
599	606	TPGTNTSN	8	IEDB
636	643	YSTGSNVF	8	IEDB
655	666	HVNNSYECDIPI	12	IEDB
674	683	YQTQTNSPRR	10	IEDB
705	714	VAYSNNSIAI	10	IEDB
740	750	MYICGDSTECS	11	IEDB
789	802	YKTPPIKDFGGFNF	14	IEDB
804	815	QILPDPSKPSKR	12	IEDB
835	842	KQYGDCLG	8	IEDB
926	931	QFNSAI	6	IEDB
934	943	IQDSLSSTAS	10	IEDB
1033	1038	VLGQSK	6	IEDB
1040	1048	VDFCGKGYH	9	IEDB
1052	1058	FPQSAPH	7	IEDB
1082	1088	CHDGKAH	7	IEDB
1096	1101	VSNGTH	6	IEDB
1119	1128	NTFVSGNCDV	10	IEDB
1136	1146	TVYDPLQPELD	11	IEDB
1155	1173	YFKNHTSPDVDLGDISGIN	19	IEDB
1237	1264	MTSCCSCLKGCCSCGSCCKFDEDDSEPV	28	IEDB
19	34	TTRTQLPPAYTNSFTR	16	IEDB
39	47	PDKVFRSSV	9	IEDB
49	54	HSTQDL	6	IEDB
71	83	SGTNGTKRFDNPV	13	IEDB
94	99	STEKSN	6	IEDB
108	116	TTLDSKTQS	9	IEDB
147	152	KNNKSW	6	IEDB
180	190	EGKQGNFKNLR	11	IEDB
205	210	SKHTPI	6	IEDB
214	220	RDLPQGF	7	IEDB
248	258	YLTPGDSSSGW	11	IEDB
279	287	YNENGTITD	9	IEDB
294	305	DPLSETKCTLKS	12	IEDB
383	389	SPTKLND	7	IEDB
403	408	RGDEVR	6	IEDB
411	417	APGQTGK	7	IEDB

424	430	KLPDDFT	7	IEDB
437	448	NSNNLDSKVGGN	12	IEDB
457	471	RKSNLKPFERDISTE	15	IEDB
475	484	AGSTPCNGVE	10	IEDB
498	503	QPTNGV	6	IEDB
526	537	GPKKSTNLVKNK	12	IEDB
545	558	GLTGTGVLTESNKK	14	IEDB
564	582	QFGRDIADTTDAVRDPQTL	19	IEDB
614	619	DVNCTE	6	IEDB
627	632	DQLTPT	6	IEDB
637	642	STGSNV	6	IEDB
675	691	QTQTNSPRRARSVASQS	17	IEDB
744	750	GDSTECS	7	IEDB
773	780	EQDKNTQE	8	IEDB
789	798	YKTPPIKDFG	10	IEDB
806	816	LPDPSKPSKRS	11	IEDB
836	841	QYGDCL	6	IEDB
880	885	GTITSG	6	IEDB
932	943	GKIQDSLSSTAS	12	IEDB
946	956	GKLQDVVNQNA	11	IEDB
961	969	TLVKQLSSN	9	IEDB
982	988	SRLDKVE	7	IEDB
994	1011	DRLITGRLQSLQTYVTQQ	18	IEDB
1034	1039	LGQSKR	6	IEDB
1070	1078	AQEKNFTTA	9	IEDB
1116	1125	TTDNTFVSGN	10	IEDB
1134	1172	NNTVYDPLQPELDSFKEELDKYFKNHTSPDVDLGDISGI	39	IEDB
1179	1187	IQKEIDRLN	9	IEDB
1191	1196	KNLNES	6	IEDB
1202	1207	ELGKYE	6	IEDB
1256	1269	FDEDDSEPVLKGVK	14	IEDB

Supplementary Table 2. Predicted B-cell epitopes with > 7 AA and antigenicity > 0.9.

start	end	peptide	Antigenicity (VaxiJen)	Peptide Length	methods	transmembrane topology (TMHMM v2.0)
4	23	FLVLLPLVSSQCVNLTTRTQ	1.0702	20	ABCPRED	outside
5	22	LVLLPLVSSQCVNLTTRT	1.1125	18	ABCPRED	outside
7	24	LLPLVSSQCVNLTTRTQL	1.1517	18	ABCPRED	outside
7	18	LLPLVSSQCVNL	1.0673	12	ABCPRED	outside
8	19	LPLVSSQCVNLT	1.4488	12	ABCPRED	outside
8	21	LPLVSSQCVNLTTR	1.3565	14	ABCPRED	outside
8	23	LPLVSSQCVNLTTRTQ	1.3416	16	ABCPRED	outside
8	23	LPLVSSQCVNLTTRTQ	1.3416	16	FBCPred	outside
8	25	LPLVSSQCVNLTTRTQLP	1.0804	18	ABCPRED	outside
9	22	PLVSSQCVNLTTRT	1.2427	14	ABCPRED	outside
9	22	PLVSSQCVNLTTRT	1.2427	14	FBCPred	outside
9	28	PLVSSQCVNLTTRTQLPPAY	1.2186	20	ABCPRED	outside
9	18	PLVSSQCVNL	1.0958	10	ABCPRED	outside
10	19	LVSSQCVNLT	1.3797	10	ABCPRED	outside
10	21	LVSSQCVNLTTR	1.2957	12	ABCPRED	outside
10	23	LVSSQCVNLTTRTQ	1.2893	14	ABCPRED	outside
10	27	LVSSQCVNLTTRTQLPPA	1.2068	18	ABCPRED	outside
10	29	LVSSQCVNLTTRTQLPPAYT	1.1791	20	ABCPRED	outside
10	25	LVSSQCVNLTTRTQLP	0.999	16	ABCPRED	outside
11	26	VSSQCVNLTTRTQLPP	1.409	16	ABCPRED	outside
11	28	VSSQCVNLTTRTQLPPAY	1.3205	18	ABCPRED	outside
11	20	VSSQCVNLTT	1.2691	10	ABCPRED	outside
12	23	SSQCVNLTTRTQ	1.5168	12	ABCPRED	outside
12	31	SSQCVNLTTRTQLPPAYTNS	1.1345	20	ABCPRED	outside
13	30	SQCVNLTTRTQLPPAYTN	1.2205	18	ABCPRED	outside
13	32	SQCVNLTTRTQLPPAYTNSF	0.9351	20	ABCPRED	outside
14	23	QCVNLTTRTQ	1.7824	10	ABCPRED	outside
14	27	QCVNLTTRTQLPPA	1.4983	14	BCPREDS	outside
14	29	QCVNLTTRTQLPPAYT	1.4165	16	ABCPRED	outside
14	25	QCVNLTTRTQLP	1.2589	12	ABCPRED	outside
14	33	QCVNLTTRTQLPPAYTNSFT	0.9669	20	ABCPRED	outside
15	26	CVNLTTRTQLPP	1.649	12	BCPREDS	outside
15	26	CVNLTTRTQLPP	1.649	12	FBCPred	outside
15	28	CVNLTTRTQLPPAY	1.483	14	ABCPRED	outside
16	27	VNLTTRTQLPPA	1.4268	12	ABCPRED	outside
16	31	VNLTTRTQLPPAYTNS	1.1248	16	ABCPRED	outside
16	31	VNLTTRTQLPPAYTNS	1.1248	16	AAP	outside
17	26	NLTTRTQLPP	1.2832	10	ABCPRED	outside
17	30	NLTTRTQLPPAYTN	1.1058	14	ABCPRED	outside
18	27	LTTRTQLPPA	1.3398	10	ABCPRED	outside
19	28	TTRTQLPPAY	1.2001	10	ABCPRED	outside
20	29	TRTQLPPAYT	1.2416	10	ABCPRED	outside
20	31	TRTQLPPAYTNS	0.9332	12	ABCPRED	outside
21	30	RTQLPPAYTN	1.1291	10	ABCPRED	outside
34	41	RGVYYPDK	1.0191	8	IEDB	outside
61	70	NVTWFHAIHV	0.9051	10	ABCPRED	outside
70	78	VSGTNGTKR	0.9855	9	BCEPRED	outside
90	97	VYFASTEK	0.9206	8	BCEPRED	outside
108	115	TTLDSKTQ	1.0912	8	BCEPRED	outside
108	116	TTLDSKTQS	1.0106	9	IEDB	outside
108	117	TTLDSKTQSL	0.99	10	BCEPRED	outside
110	121	LDSKTQSLLIVN	0.9139	12	ABCPRED	outside
137	148	NDPFLGVYYHKN	0.9849	12	ABCPRED	outside
138	147	DPFLGVYYHK	0.9542	10	ABCPRED	outside
141	150	LGVYYHKNNK	0.9727	10	ABCPRED	outside
167	186	TFEYVSQPFLMDLEGKQGNF	1.009	20	ABCPRED	outside
168	185	FEYVSQPFLMDLEGKQGN	0.9891	18	ABCPRED	outside
171	186	VSQPFLMDLEGKQGNF	0.9279	16	ABCPRED	outside
172	185	SQPFLMDLEGKQGN	0.9603	14	ABCPRED	outside
172	187	SQPFLMDLEGKQGNFK	0.9284	16	ABCPRED	outside
174	185	PFLMDLEGKQGN	1.2067	12	ABCPRED	outside
174	187	PFLMDLEGKQGNFK	1.1174	14	ABCPRED	outside
174	189	PFLMDLEGKQGNFKNL	1.0418	16	ABCPRED	outside
174	183	PFLMDLEGKQ	0.915	10	ABCPRED	outside
177	186	MDLEGKQGNF	1.7852	10	ABCPRED	outside
177	189	MDLEGKQGNFKNL	1.2592	13	IEDB	outside
178	187	DLEGKQGNFK	1.3065	10	ABCPRED	outside
180	188	EGKQGNFKN	1.1232	9	IEDB	outside
180	190	EGKQGNFKNLR	1.0042	11	IEDB	outside
181	188	GKQGNFKN	1.0999	8	IEDB	outside
199	212	GYFKIYSKHTPINL	0.9308	14	ABCPRED	outside
201	208	FKIYSKHT	1.1661	8	BCEPRED	outside
201	212	FKIYSKHTPINL	1.1557	12	ABCPRED	outside
201	214	FKIYSKHTPINLVR	0.9156	14	ABCPRED	outside
203	212	IYSKHTPINL	1.2064	10	ABCPRED	outside
204	213	YSKHTPINLV	1.0391	10	ABCPRED	outside
213	226	VRDLPQGFSALEPL	0.9407	14	ABCPRED	outside
223	236	LEPLVDLPIGINIT	1.1627	14	ABCPRED	outside
224	237	EPLVDLPIGINITR	0.9556	14	ABCPRED	outside
225	236	PLVDLPIGINIT	1.3843	12	ABCPRED	outside
227	240	VDLPIGINITRFQT	0.9138	14	ABCPRED	outside
228	239	DLPIGINITRFQ	1.3916	12	ABCPRED	outside

228	241	DLPIGINITRFQTL	1.1058	14	ABCPRED	outside
228	245	DLPIGINITRFQTLLALH	0.9638	18	ABCPRED	outside
229	240	LPIGINITRFQT	0.9919	12	ABCPRED	outside
230	241	PIGINITRFQTL	1.0169	12	ABCPRED	outside
258	273	WTAGAAAYYVGYLQPR	0.9545	16	ABCPRED	outside
260	273	AGAAAYYVGYLQPR	1.0663	14	ABCPRED	outside
262	273	AAAYYVGYLQPR	1.0262	12	ABCPRED	outside
263	274	AAYYVGYLQPRT	0.9255	12	BCPREDS	outside
264	273	AYYVGYLQPR	1.3309	10	ABCPRED	outside
265	273	YYVGYLQPR	1.4692	9	BCEPRED	outside
278	287	KYNENGTITD	0.9589	10	BCEPRED	outside
291	300	CALDPLSETK	0.9226	10	ABCPRED	outside
292	305	ALDPLSETKCTLKS	1.017	14	ABCPRED	outside
293	302	LDPLSETKCT	1.2586	10	ABCPRED	outside
293	304	LDPLSETKCTLK	0.9712	12	ABCPRED	outside
294	305	DPLSETKCTLKS	0.9204	12	FBCPred	outside
294	305	DPLSETKCTLKS	0.9204	12	IEDB	outside
295	302	PLSETKCT	1.2573	8	IEDB	outside
296	305	LSETKCTLKS	0.9434	10	ABCPRED	outside
310	323	KGIYQTSNFRVQPT	0.962	14	ABCPRED	outside
311	322	GIYQTSNFRVQP	0.9986	12	ABCPRED	outside
312	323	IYQTSNFRVQPT	1.1283	12	ABCPRED	outside
312	323	IYQTSNFRVQPT	1.1283	12	AAP	outside
313	322	YQTSNFRVQP	1.1866	10	ABCPRED	outside
314	323	QTSNFRVQPT	1.405	10	ABCPRED	outside
314	323	QTSNFRVQPT	1.405	10	IEDB	outside
315	324	TSNFRVQPTE	1.3571	10	ABCPRED	outside
315	326	TSNFRVQPTESI	0.9265	12	ABCPRED	outside
316	325	SNFRVQPTES	1.2078	10	IEDB	outside
317	326	NFRVQPTESI	1.2412	10	ABCPRED	outside
326	337	IVRFPNITNLCP	0.9596	12	ABCPRED	outside
327	338	VRFPNITNLCPF	1.2901	12	ABCPRED	outside
327	336	VRFPNITNLC	1.1924	10	ABCPRED	outside
327	340	VRFPNITNLCPFGE	1.0597	14	ABCPRED	outside
328	337	RFPNITNLCP	1.4779	10	ABCPRED	outside
329	340	FPNITNLCPFGE	1.0991	12	ABCPRED	outside
330	339	PNITNLCPFG	1.4887	10	ABCPRED	outside
331	340	NITNLCPFGE	1.237	10	ABCPRED	outside
369	393	YNSASFSTFKCYGVSPTKLNDLCFT	1.4031	25	IEDB	outside
369	390	YNSASFSTFKCYGVSPTKLNDL	0.9508	22	AAP	outside
371	388	SASFSTFKCYGVSPTKLN	0.9891	18	ABCPRED	outside
371	386	SASFSTFKCYGVSPTK	0.908	16	ABCPRED	outside
372	391	ASFSTFKCYGVSPTKLNDLC	1.227	20	ABCPRED	outside
372	387	ASFSTFKCYGVSPTKL	0.912	16	ABCPRED	outside
373	386	SFSTFKCYGVSPTK	0.9678	14	ABCPRED	outside
374	391	FSTFKCYGVSPTKLNDLC	1.2396	18	ABCPRED	outside
374	389	FSTFKCYGVSPTKLND	0.965	16	ABCPRED	outside
375	392	STFKCYGVSPTKLNDLCF	1.7606	18	ABCPRED	outside
375	388	STFKCYGVSPTKLN	1.2695	14	AAP	outside
375	390	STFKCYGVSPTKLNDL	1.2492	16	ABCPRED	outside
375	384	STFKCYGVSP	1.104	10	ABCPRED	outside
376	385	TFKCYGVSPT	1.5059	10	ABCPRED	outside
376	385	TFKCYGVSPT	1.5059	10	IEDB	outside
376	387	TFKCYGVSPTKL	1.4293	12	ABCPRED	outside
377	392	FKCYGVSPTKLNDLCF	2.0332	16	ABCPRED	outside
377	394	FKCYGVSPTKLNDLCFTN	1.9296	18	ABCPRED	outside
377	390	FKCYGVSPTKLNDL	1.4748	14	ABCPRED	outside
378	393	KCYGVSPTKLNDLCFT	2.0395	16	ABCPRED	outside
378	395	KCYGVSPTKLNDLCFTNV	1.8687	18	ABCPRED	outside
378	397	KCYGVSPTKLNDLCFTNVYA	1.5664	20	ABCPRED	outside
378	397	KCYGVSPTKLNDLCFTNVYA	1.5664	20	AAP	outside
378	389	KCYGVSPTKLND	1.3195	12	ABCPRED	outside
378	389	KCYGVSPTKLND	1.3195	12	BCPREDS	outside
379	394	CYGVSPTKLNDLCFTN	2.0134	16	ABCPRED	outside
379	396	CYGVSPTKLNDLCFTNVY	1.7815	18	ABCPRED	outside
379	388	CYGVSPTKLN	1.5759	10	ABCPRED	outside
379	398	CYGVSPTKLNDLCFTNVYAD	1.5222	20	ABCPRED	outside
379	390	CYGVSPTKLNDL	1.4808	12	ABCPRED	outside
380	395	YGVSPTKLNDLCFTNV	2.0255	16	ABCPRED	outside
380	391	YGVSPTKLNDLC	1.8347	12	ABCPRED	outside
380	391	YGVSPTKLNDLC	1.8347	12	AAP	outside
380	389	YGVSPTKLND	1.4531	10	IEDB	outside
380	399	YGVSPTKLNDLCFTNVYADS	1.361	20	ABCPRED	outside
380	401	YGVSPTKLNDLCFTNVYADSFV	1.1404	22	BCPREDS	outside
381	388	GVSPTKLN	1.9197	8	IEDB	outside
381	396	GVSPTKLNDLCFTNVY	1.9127	16	ABCPRED	outside
381	390	GVSPTKLNDL	1.6525	10	ABCPRED	outside
381	400	GVSPTKLNDLCFTNVYADSF	1.2617	20	ABCPRED	outside
382	393	VSPTKLNDLCFT	2.4268	12	ABCPRED	outside
382	391	VSPTKLNDLC	1.9573	10	ABCPRED	outside
383	392	SPTKLNDLCF	2.2412	10	ABCPRED	outside
383	396	SPTKLNDLCFTNVY	1.6982	14	ABCPRED	outside
383	398	SPTKLNDLCFTNVYAD	1.3666	16	ABCPRED	outside
383	390	SPTKLNDL	1.0358	8	BCEPRED	outside

383	402	SPTKLNDLCFTNVYADSFVI	0.9482	20	ABCPRED	outside
384	393	PTKLNDLCFT	2.5246	10	ABCPRED	outside
384	399	PTKLNDLCFTNVYADS	1.2303	16	ABCPRED	outside
384	403	PTKLNDLCFTNVYADSFVIR	0.9772	20	ABCPRED	outside
385	396	TKLNDLCFTNVY	2.0035	12	ABCPRED	outside
385	404	TKLNDLCFTNVYADSFVIRG	1.049	20	ABCPRED	outside
385	402	TKLNDLCFTNVYADSFVI	1.0271	18	ABCPRED	outside
386	395	KLNDLCFTNV	2.6927	10	ABCPRED	outside
386	403	KLNDLCFTNVYADSFVIR	1.0694	18	ABCPRED	outside
386	405	KLNDLCFTNVYADSFVIRGD	0.932	20	ABCPRED	outside
387	396	LNDLCFTNVY	1.6134	10	ABCPRED	outside
389	396	DLCFTNVY	1.8569	8	BCEPRED	outside
404	423	GDEVRQIAPGQTGKIADYNY	1.219	20	ABCPRED	outside
404	417	GDEVRQIAPGQTGK	1.1397	14	ABCPRED	outside
404	417	GDEVRQIAPGQTGK	1.1397	14	AAP	outside
404	417	GDEVRQIAPGQTGK	1.1397	14	FBCPred	outside
404	426	GDEVRQIAPGQTGKIADYNYKLP	1.1017	23	IEDB	outside
405	416	DEVRQIAPGQTG	1.0397	12	ABCPRED	outside
405	420	DEVRQIAPGQTGKIAD	1.0388	16	ABCPRED	outside
406	423	EVRQIAPGQTGKIADYNY	1.4691	18	BCPREDS	outside
406	421	EVRQIAPGQTGKIADY	1.3837	16	ABCPRED	outside
406	419	EVRQIAPGQTGKIA	1.3182	14	ABCPRED	outside
406	415	EVRQIAPGQT	1.0655	10	ABCPRED	outside
407	416	VRQIAPGQTG	1.3856	10	ABCPRED	outside
407	422	VRQIAPGQTGKIADYN	1.3372	16	ABCPRED	outside
407	420	VRQIAPGQTGKIAD	1.2606	14	IEDB	outside
407	426	VRQIAPGQTGKIADYNYKLP	1.2386	20	ABCPRED	outside
408	423	RQIAPGQTGKIADYNY	1.5799	16	ABCPRED	outside
408	421	RQIAPGQTGKIADY	1.504	14	ABCPRED	outside
408	419	RQIAPGQTGKIA	1.4558	12	BCPREDS	outside
408	419	RQIAPGQTGKIA	1.4558	12	FBCPred	outside
408	427	RQIAPGQTGKIADYNYKLPD	1.4102	20	ABCPRED	outside
408	427	RQIAPGQTGKIADYNYKLPD	1.4102	20	BCPREDS	outside
409	422	QIAPGQTGKIADYN	1.5448	14	ABCPRED	outside
409	418	QIAPGQTGKI	1.4416	10	ABCPRED	outside
410	423	IAPGQTGKIADYNY	1.724	14	ABCPRED	outside
410	423	IAPGQTGKIADYNY	1.724	14	BCPREDS	outside
410	425	IAPGQTGKIADYNYKL	1.6707	16	ABCPRED	outside
410	421	IAPGQTGKIADY	1.6626	12	ABCPRED	outside
410	431	IAPGQTGKIADYNYKLPDDFTG	1.1317	22	BCPREDS	outside
411	425	APGQTGKIADYNYKL	1.4441	15	IEDB	outside
411	420	APGQTGKIAD	1.2753	10	ABCPRED	outside
411	426	APGQTGKIADYNYKLP	1.2303	16	ABCPRED	outside
411	430	APGQTGKIADYNYKLPDDFT	1.0425	20	ABCPRED	outside
411	430	APGQTGKIADYNYKLPDDFT	1.0425	20	IEDB	outside
412	423	PGQTGKIADYNY	1.642	12	ABCPRED	outside
412	427	PGQTGKIADYNYKLPD	1.4039	16	ABCPRED	outside
412	431	PGQTGKIADYNYKLPDDFTG	1.0123	20	ABCPRED	outside
413	424	GQTGKIADYNYK	1.7308	12	ABCPRED	outside
413	426	GQTGKIADYNYKLP	1.2756	14	ABCPRED	outside
413	430	GQTGKIADYNYKLPDDFT	1.0564	18	ABCPRED	outside
413	428	GQTGKIADYNYKLPDD	1.0071	16	ABCPRED	outside
414	423	QTGKIADYNY	1.5116	10	ABCPRED	outside
414	425	QTGKIADYNYKL	1.4851	12	ABCPRED	outside
414	427	QTGKIADYNYKLPD	1.2934	14	ABCPRED	outside
414	429	QTGKIADYNYKLPDDF	0.986	16	ABCPRED	outside
415	426	TGKIADYNYKLP	1.1956	12	ABCPRED	outside
415	430	TGKIADYNYKLPDDFT	0.9642	16	ABCPRED	outside
416	425	GKIADYNYKL	1.6079	10	ABCPRED	outside
416	427	GKIADYNYKLPD	1.3553	12	ABCPRED	outside
416	429	GKIADYNYKLPDDF	0.9776	14	ABCPRED	outside
417	426	KIADYNYKLP	1.2565	10	ABCPRED	outside
417	430	KIADYNYKLPDDFT	0.9567	14	BCEPRED	outside
433	442	VIAWNSNNLD	0.9177	10	ABCPRED	outside
434	451	IAWNSNNLDSKVGGNYNY	1.0683	18	ABCPRED	outside
434	449	IAWNSNNLDSKVGGNY	0.906	16	ABCPRED	outside
435	444	AWNSNNLDSK	1.0198	10	BCEPRED	outside
439	446	NNLDSKVG	1.2952	8	BCEPRED	outside
441	450	LDSKVGGNYN	0.91	10	ABCPRED	outside
456	463	FRKSNLKP	1.1111	8	BCEPRED	outside
482	493	GVEGFNCYFPLQ	0.9537	12	ABCPRED	outside
482	501	GVEGFNCYFPLQSYGFQPTN	0.9121	20	ABCPRED	outside
485	500	GFNCYFPLQSYGFQPT	0.981	16	ABCPRED	outside
486	501	FNCYFPLQSYGFQPTN	1.0229	16	ABCPRED	outside
488	495	CYFPLQSY	0.9394	8	IEDB	outside
490	501	FPLQSYGFQPTN	0.9942	12	ABCPRED	outside
492	501	LQSYGFQPTN	1.043	10	ABCPRED	outside
493	502	QSYGFQPTNG	0.9097	10	ABCPRED	outside
494	503	SYGFQPTNGV	1.0549	10	ABCPRED	outside
501	516	NGVGYQPYRVVVLSFE	0.9736	16	ABCPRED	outside
501	516	NGVGYQPYRVVVLSFE	0.9736	16	BCPREDS	outside
501	520	NGVGYQPYRVVVLSFELLHA	0.9212	20	ABCPRED	outside
502	515	GVGYQPYRVVVLSF	1.2391	14	ABCPRED	outside
502	511	GVGYQPYRVV	1.1915	10	ABCPRED	outside

502	513	GVGYQPYRVVVL	1.1207	12	BCPREDS	outside
502	513	GVGYQPYRVVVL	1.1207	12	FBCPred	outside
503	511	VGYQPYRVV	1.4383	9	BCEPRED	outside
503	522	VGYQPYRVVVLSFELLHAPA	0.989	20	ABCPRED	outside
504	515	GYQPYRVVVLSF	1.1235	12	ABCPRED	outside
505	516	YQPYRVVVLSFE	0.9911	12	ABCPRED	outside
505	519	YQPYRVVVLSFELLH	0.9711	15	BCEPRED	outside
506	517	QPYRVVVLSFEL	0.9785	12	ABCPRED	outside
506	519	QPYRVVVLSFELLH	0.9675	14	ABCPRED	outside
507	520	PYRVVVLSFELLHA	1.0185	14	ABCPRED	outside
508	517	YRVVVLSFEL	1.1494	10	ABCPRED	outside
508	519	YRVVVLSFELLH	1.1085	12	ABCPRED	outside
513	524	LSFELLHAPATV	0.9969	12	ABCPRED	outside
526	543	GPKKSTNLVKNKCVNFNF	1.1626	18	ABCPRED	outside
527	546	PKKSTNLVKNKCVNFNFNGL	1.0582	20	ABCPRED	outside
528	541	KKSTNLVKNKCVNF	1.0894	14	ABCPRED	outside
528	547	KKSTNLVKNKCVNFNFNGLT	1.0818	20	ABCPRED	outside
529	544	KSTNLVKNKCVNFNFN	1.4412	16	ABCPRED	outside
529	546	KSTNLVKNKCVNFNFNGL	1.22	18	ABCPRED	outside
530	543	STNLVKNKCVNFNF	1.289	14	ABCPRED	outside
530	549	STNLVKNKCVNFNFNGLTGT	1.0756	20	ABCPRED	outside
530	551	STNLVKNKCVNFNFNGLTGTGV	1.0662	22	BCPREDS	outside
530	547	STNLVKNKCVNFNFNGLT	0.9604	18	ABCPRED	outside
531	544	TNLVKNKCVNFNFN	1.2489	14	ABCPRED	outside
531	544	TNLVKNKCVNFNFN	1.2489	14	BCPREDS	outside
532	543	NLVKNKCVNFNF	1.3432	12	ABCPRED	outside
532	551	NLVKNKCVNFNFNGLTGTGV	1.084	20	ABCPRED	outside
533	544	LVKNKCVNFNFN	1.6246	12	FBCPred	outside
533	546	LVKNKCVNFNFNGL	1.3128	14	FBCPred	outside
533	550	LVKNKCVNFNFNGLTGTG	1.2501	18	ABCPRED	outside
534	547	VKNKCVNFNFNGLT	1.5749	14	ABCPRED	outside
535	544	KNKCVNFNFN	2.2331	10	ABCPRED	outside
535	546	KNKCVNFNFNGL	1.7395	12	ABCPRED	outside
535	548	KNKCVNFNFNGLTG	1.6052	14	ABCPRED	outside
535	552	KNKCVNFNFNGLTGTGVL	1.3849	18	ABCPRED	outside
535	554	KNKCVNFNFNGLTGTGVLTE	1.2763	20	ABCPRED	outside
536	549	NKCVNFNFNGLTGT	1.3691	14	ABCPRED	outside
536	545	NKCVNFNFNG	1.3088	10	ABCPRED	outside
536	555	NKCVNFNFNGLTGTGVLTES	1.0131	20	ABCPRED	outside
537	548	KCVNFNFNGLTG	1.6969	12	IEDB	outside
537	550	KCVNFNFNGLTGTG	1.5894	14	ABCPRED	outside
537	552	KCVNFNFNGLTGTGVL	1.4136	16	AAP	outside
537	556	KCVNFNFNGLTGTGVLTESN	1.2355	20	ABCPRED	outside
538	546	CVNFNFNGL	1.7985	9	BCEPRED	outside
538	557	CVNFNFNGLTGTGVLTESNK	1.1352	20	ABCPRED	outside
538	555	CVNFNFNGLTGTGVLTES	1.1292	18	ABCPRED	outside
539	552	VNFNFNGLTGTGVL	1.2915	14	ABCPRED	outside
539	554	VNFNFNGLTGTGVLTE	1.1691	16	ABCPRED	outside
539	558	VNFNFNGLTGTGVLTESNKK	1.1457	20	ABCPRED	outside
540	551	NFNFNGLTGTGV	1.3547	12	ABCPRED	outside
540	551	NFNFNGLTGTGV	1.3547	12	BCPREDS	outside
540	553	NFNFNGLTGTGVLT	1.1216	14	ABCPRED	outside
540	559	NFNFNGLTGTGVLTESNKKF	0.9432	20	ABCPRED	outside
541	550	FNFNGLTGTG	1.0567	10	ABCPRED	outside
541	558	FNFNGLTGTGVLTESNKK	0.9066	18	ABCPRED	outside
545	558	GLTGTGVLTESNKK	1.0227	14	IEDB	outside
546	563	LTGTGVLTESNKKFLPFQ	0.941	18	ABCPRED	outside
547	562	TGTGVLTESNKKFLPF	0.9925	16	ABCPRED	outside
553	563	TESNKKFLPFQ	0.9243	11	ellipro	outside
555	562	SNKKFLPF	1.3952	8	IEDB	outside
555	564	SNKKFLPFQQ	0.9488	10	ABCPRED	outside
568	589	DIADTTDAVRDPQTLEILDITP	1.191	22	FBCPred	outside
572	589	TTDAVRDPQTLEILDITP	1.1067	18	AAP	outside
573	588	TDAVRDPQTLEILDIT	0.9832	16	ABCPRED	outside
574	589	DAVRDPQTLEILDITP	1.1133	16	ABCPRED	outside
574	595	DAVRDPQTLEILDITPCSFGGV	1.0597	22	AAP	outside
575	594	AVRDPQTLEILDITPCSFGG	1.1985	20	ABCPRED	outside
576	589	VRDPQTLEILDITP	1.4571	14	ABCPRED	outside
577	596	RDPQTLEILDITPCSFGGVS	1.4479	20	ABCPRED	outside
577	590	RDPQTLEILDITPC	1.2642	14	ABCPRED	outside
577	586	RDPQTLEILD	1.0339	10	ABCPRED	outside
577	584	RDPQTLEI	0.9248	8	ellipro	outside
578	587	DPQTLEILDI	1.5757	10	ABCPRED	outside
578	597	DPQTLEILDITPCSFGGVSV	1.5468	20	ABCPRED	outside
578	595	DPQTLEILDITPCSFGGV	1.4948	18	ABCPRED	outside
578	591	DPQTLEILDITPCS	1.252	14	ABCPRED	outside
579	590	PQTLEILDITPC	1.4158	12	ABCPRED	outside
579	598	PQTLEILDITPCSFGGVSVI	1.3978	20	ABCPRED	outside
579	592	PQTLEILDITPCSF	1.3489	14	ABCPRED	outside
580	589	QTLEILDITP	2.027	10	ABCPRED	outside
580	597	QTLEILDITPCSFGGVSV	1.5916	18	ABCPRED	outside
580	595	QTLEILDITPCSFGGV	1.5382	16	ABCPRED	outside
580	599	QTLEILDITPCSFGGVSVIT	1.3356	20	ABCPRED	outside
580	591	QTLEILDITPCS	1.2575	12	ABCPRED	outside

581	596	TLEILDITPCSFGGVS	1.6878	16	ABCPRED	outside
581	594	TLEILDITPCSFGG	1.5811	14	ABCPRED	outside
581	598	TLEILDITPCSFGGVSVI	1.4847	18	ABCPRED	outside
581	600	TLEILDITPCSFGGVSVITP	1.2987	20	ABCPRED	outside
582	593	LEILDITPCSFG	1.759	12	ABCPRED	outside
582	597	LEILDITPCSFGGVSV	1.7357	16	ABCPRED	outside
582	599	LEILDITPCSFGGVSVIT	1.428	18	ABCPRED	outside
582	601	LEILDITPCSFGGVSVITPG	1.2631	20	ABCPRED	outside
583	596	EILDITPCSFGGVS	1.6193	14	ABCPRED	outside
583	598	EILDITPCSFGGVSVI	1.3971	16	ABCPRED	outside
583	592	EILDITPCSF	1.2698	10	ABCPRED	outside
583	602	EILDITPCSFGGVSVITPGT	1.0825	20	ABCPRED	outside
584	597	ILDITPCSFGGVSV	1.6035	14	ABCPRED	outside
584	593	ILDITPCSFG	1.5391	10	ABCPRED	outside
584	595	ILDITPCSFGGV	1.5192	12	ABCPRED	outside
584	599	ILDITPCSFGGVSVIT	1.2689	16	ABCPRED	outside
584	601	ILDITPCSFGGVSVITPG	1.1031	18	BCEPRED	outside
584	603	ILDITPCSFGGVSVITPGTN	0.9838	20	ABCPRED	outside
585	596	LDITPCSFGGVS	1.9283	12	ABCPRED	outside
585	598	LDITPCSFGGVSVI	1.6124	14	ABCPRED	outside
585	600	LDITPCSFGGVSVITP	1.3502	16	ABCPRED	outside
585	602	LDITPCSFGGVSVITPGT	1.2006	18	ABCPRED	outside
585	604	LDITPCSFGGVSVITPGTNT	1.0966	20	ABCPRED	outside
586	603	DITPCSFGGVSVITPGTN	1.0022	18	ABCPRED	outside
586	605	DITPCSFGGVSVITPGTNTS	0.9823	20	ABCPRED	outside
587	600	ITPCSFGGVSVITP	0.9725	14	ABCPRED	outside
588	599	TPCSFGGVSVIT	1.1714	12	ABCPRED	outside
588	601	TPCSFGGVSVITPG	0.9486	14	ABCPRED	outside
590	599	CSFGGVSVIT	1.0615	10	ABCPRED	outside
598	607	ITPGTNTSNQ	0.941	10	ABCPRED	outside
607	620	QVAVLYQDVNCTEV	0.9497	14	ABCPRED	outside
609	620	AVLYQDVNCTEV	0.9218	12	ABCPRED	outside
612	623	YQDVNCTEVPVA	1.0646	12	AAP	outside
612	623	YQDVNCTEVPVA	1.0646	12	FBCPred	outside
612	625	YQDVNCTEVPVAIH	1.0383	14	ABCPRED	outside
614	623	DVNCTEVPVA	1.237	10	ABCPRED	outside
615	624	VNCTEVPVAI	0.9137	10	ABCPRED	outside
628	639	QLTPTWRVYSTG	1.1857	12	ABCPRED	outside
628	641	QLTPTWRVYSTGSN	0.9924	14	ABCPRED	outside
629	638	LTPTWRVYST	1.1346	10	ABCPRED	outside
637	650	STGSNVFQTRAGCL	0.9742	14	ABCPRED	outside
638	655	TGSNVFQTRAGCLIGAEH	0.9162	18	ABCPRED	outside
639	650	GSNVFQTRAGCL	1.0436	12	ABCPRED	outside
639	654	GSNVFQTRAGCLIGAE	0.9074	16	ABCPRED	outside
640	657	SNVFQTRAGCLIGAEHVN	0.9399	18	ABCPRED	outside
641	660	NVFQTRAGCLIGAEHVNNSY	0.9383	20	ABCPRED	outside
642	657	VFQTRAGCLIGAEHVN	1.2576	16	ABCPRED	outside
642	655	VFQTRAGCLIGAEH	1.2294	14	ABCPRED	outside
642	659	VFQTRAGCLIGAEHVNNS	1.1368	18	ABCPRED	outside
643	656	FQTRAGCLIGAEHV	1.27	14	ABCPRED	outside
643	658	FQTRAGCLIGAEHVNN	1.2294	16	ABCPRED	outside
644	657	QTRAGCLIGAEHVN	1.3917	14	ABCPRED	outside
645	662	TRAGCLIGAEHVNNSYEC	1.1982	18	ABCPRED	outside
645	664	TRAGCLIGAEHVNNSYECDI	1.0613	20	ABCPRED	outside
646	657	RAGCLIGAEHVN	1.3707	12	ABCPRED	outside
646	659	RAGCLIGAEHVNNS	1.1927	14	ABCPRED	outside
646	661	RAGCLIGAEHVNNSYE	1.1613	16	ABCPRED	outside
647	662	AGCLIGAEHVNNSYEC	0.914	16	ABCPRED	outside
648	667	GCLIGAEHVNNSYECDIPIG	0.9157	20	ABCPRED	outside
649	658	CLIGAEHVNN	1.1765	10	ABCPRED	outside
649	664	CLIGAEHVNNSYECDI	1.0413	16	ABCPRED	outside
649	668	CLIGAEHVNNSYECDIPIGA	1.0187	20	ABCPRED	outside
649	666	CLIGAEHVNNSYECDIPI	0.9191	18	ABCPRED	outside
649	666	CLIGAEHVNNSYECDIPI	0.9191	18	FBCPred	outside
650	659	LIGAEHVNNS	0.9735	10	ABCPRED	outside
650	667	LIGAEHVNNSYECDIPIG	0.9717	18	ABCPRED	outside
650	663	LIGAEHVNNSYECD	0.9242	14	ABCPRED	outside
651	670	IGAEHVNNSYECDIPIGAGI	1.1141	20	ABCPRED	outside
651	670	IGAEHVNNSYECDIPIGAGI	1.1141	20	BCPREDS	outside
651	668	IGAEHVNNSYECDIPIGA	1.0231	18	ABCPRED	outside
651	666	IGAEHVNNSYECDIPI	0.9115	16	FBCPred	outside
652	669	GAEHVNNSYECDIPIGAG	0.9847	18	ABCPRED	outside
652	669	GAEHVNNSYECDIPIGAG	0.9847	18	AAP	outside
652	669	GAEHVNNSYECDIPIGAG	0.9847	18	BCPREDS	outside
654	662	EHVNNSYEC	1.068	9	IEDB	outside
663	678	DIPIGAGICASYQTQT	1.0235	16	ABCPRED	outside
664	679	IPIGAGICASYQTQTN	0.9249	16	ABCPRED	outside
669	678	GICASYQTQT	0.9671	10	ABCPRED	outside
697	714	MSLGAENSVAYSNNSIAI	0.9071	18	ABCPRED	outside
705	714	VAYSNNSIAI	1.0545	10	IEDB	outside
712	723	IAIPTNFTISVT	1.0108	12	ABCPRED	outside
714	733	IPTNFTISVTTEILPVSMTK	1.0688	20	ABCPRED	outside
714	731	IPTNFTISVTTEILPVSM	0.9956	18	ABCPRED	outside
715	732	PTNFTISVTTEILPVSMT	1.3036	18	ABCPRED	outside

715	734	PTNFTISVTTEILPVSMTKT	1.2666	20	ABCPRED	outside
715	730	PTNFTISVTTEILPVS	1.1529	16	FBCPred	outside
715	726	PTNFTISVTTEI	1.0904	12	BCPREDS	outside
716	729	TNFTISVTTEILPV	1.1495	14	FBCPred	outside
717	732	NFTISVTTEILPVSMT	1.3573	16	ABCPRED	outside
717	734	NFTISVTTEILPVSMTKT	1.3127	18	ABCPRED	outside
719	738	TISVTTEILPVSMTKTSVDC	1.3041	20	ABCPRED	outside
719	738	TISVTTEILPVSMTKTSVDC	1.3041	20	AAP	outside
719	734	TISVTTEILPVSMTKT	1.2262	16	ABCPRED	outside
719	736	TISVTTEILPVSMTKTSV	1.1598	18	ABCPRED	outside
719	730	TISVTTEILPVS	1.036	12	ABCPRED	outside
719	730	TISVTTEILPVS	1.036	12	FBCPred	outside
720	739	ISVTTEILPVSMTKTSVDCT	1.4126	20	ABCPRED	outside
720	739	ISVTTEILPVSMTKTSVDCT	1.4126	20	FBCPred	outside
720	733	ISVTTEILPVSMTK	1.286	14	ABCPRED	outside
720	741	ISVTTEILPVSMTKTSVDCTMY	1.2581	22	FBCPred	outside
720	737	ISVTTEILPVSMTKTSVD	1.1887	18	ABCPRED	outside
720	729	ISVTTEILPV	1.1242	10	ABCPRED	outside
721	738	SVTTEILPVSMTKTSVDC	1.2841	18	ABCPRED	outside
721	732	SVTTEILPVSMT	1.2175	12	ABCPRED	outside
721	734	SVTTEILPVSMTKT	1.1868	14	ABCPRED	outside
721	730	SVTTEILPVS	0.9195	10	ABCPRED	outside
722	739	VTTEILPVSMTKTSVDCT	1.3791	18	ABCPRED	outside
722	739	VTTEILPVSMTKTSVDCT	1.3791	18	AAP	outside
722	739	VTTEILPVSMTKTSVDCT	1.3791	18	FBCPred	outside
722	741	VTTEILPVSMTKTSVDCTMY	1.2085	20	ABCPRED	outside
722	737	VTTEILPVSMTKTSVD	1.1143	16	ABCPRED	outside
722	737	VTTEILPVSMTKTSVD	1.1143	16	AAP	outside
722	731	VTTEILPVSM	1.0712	10	ABCPRED	outside
723	732	TTEILPVSMT	1.484	10	ABCPRED	outside
723	740	TTEILPVSMTKTSVDCTM	1.3906	18	ABCPRED	outside
723	736	TTEILPVSMTKTSV	1.2571	14	AAP	outside
723	730	TTEILPVS	1.2071	8	IEDB	outside
723	742	TTEILPVSMTKTSVDCTMYI	1.1726	20	ABCPRED	outside
723	742	TTEILPVSMTKTSVDCTMYI	1.1726	20	BCPREDS	outside
724	739	TEILPVSMTKTSVDCT	1.5042	16	ABCPRED	outside
724	735	TEILPVSMTKTS	1.4845	12	ABCPRED	outside
724	741	TEILPVSMTKTSVDCTMY	1.2922	18	ABCPRED	outside
725	738	EILPVSMTKTSVDC	1.617	14	ABCPRED	outside
725	740	EILPVSMTKTSVDCTM	1.5372	16	ABCPRED	outside
725	742	EILPVSMTKTSVDCTMYI	1.2689	18	ABCPRED	outside
726	739	ILPVSMTKTSVDCT	1.606	14	ABCPRED	outside
727	740	LPVSMTKTSVDCTM	1.4435	14	BCPREDS	outside
727	736	LPVSMTKTSV	1.2574	10	ABCPRED	outside
727	742	LPVSMTKTSVDCTMYI	1.1469	16	ABCPRED	outside
728	741	PVSMTKTSVDCTMY	1.3667	14	ABCPRED	outside
729	738	VSMTKTSVDC	1.6231	10	ABCPRED	outside
730	739	SMTKTSVDCT	1.4047	10	ABCPRED	outside
730	741	SMTKTSVDCTMY	1.0622	12	ABCPRED	outside
731	738	MTKTSVDC	1.5932	8	IEDB	outside
731	740	MTKTSVDCTM	1.3958	10	ABCPRED	outside
732	741	TKTSVDCTMY	1.2485	10	ABCPRED	outside
733	742	KTSVDCTMYI	0.9345	10	ABCPRED	outside
751	760	NLLLQYGSFC	1.1023	10	ABCPRED	outside
753	764	LLQYGSFCTQLN	0.9501	12	AAP	outside
753	766	LLQYGSFCTQLNRA	0.9077	14	ABCPRED	outside
753	766	LLQYGSFCTQLNRA	0.9077	14	AAP	outside
754	763	LQYGSFCTQL	1.4443	10	ABCPRED	outside
754	765	LQYGSFCTQLNR	1.383	12	BCPREDS	outside
754	767	LQYGSFCTQLNRAL	1.0734	14	ABCPRED	outside
754	771	LQYGSFCTQLNRALTGIA	0.9721	18	ABCPRED	outside
755	764	QYGSFCTQLN	1.3057	10	ABCPRED	outside
755	766	QYGSFCTQLNRA	1.1892	12	ABCPRED	outside
756	765	YGSFCTQLNR	0.9565	10	ABCPRED	outside
766	777	ALTGIAVEQDKN	0.9459	12	ABCPRED	outside
768	777	TGIAVEQDKN	1.0371	10	ABCPRED	outside
841	852	LGDIAARDLICA	1.1914	12	ABCPRED	outside
841	854	LGDIAARDLICAQK	1.1817	14	ABCPRED	outside
841	856	LGDIAARDLICAQKFN	0.9412	16	ABCPRED	outside
842	853	GDIAARDLICAQ	1.1569	12	ABCPRED	outside
890	905	AGAALQIPFAMQMAYR	0.9036	16	ABCPRED	outside
892	905	AALQIPFAMQMAYR	1.0216	14	ABCPRED	outside
893	912	ALQIPFAMQMAYRFNGIGVT	1.2262	20	ABCPRED	outside
894	913	LQIPFAMQMAYRFNGIGVTQ	1.2425	20	ABCPRED	outside
894	911	LQIPFAMQMAYRFNGIGV	1.1951	18	ABCPRED	outside
894	907	LQIPFAMQMAYRFN	1.0747	14	ABCPRED	outside
895	906	QIPFAMQMAYRF	1.1307	12	ABCPRED	outside
896	915	IPFAMQMAYRFNGIGVTQNV	1.2009	20	ABCPRED	outside
896	909	IPFAMQMAYRFNGI	1.0623	14	ABCPRED	outside
897	912	PFAMQMAYRFNGIGVT	1.3871	16	ABCPRED	outside
897	913	PFAMQMAYRFNGIGVTQ	1.3306	17	COVIDep	outside
897	914	PFAMQMAYRFNGIGVTQN	1.2715	18	ABCPRED	outside
898	913	FAMQMAYRFNGIGVTQ	1.3096	16	ABCPRED	outside
898	917	FAMQMAYRFNGIGVTQNVLY	0.9281	20	ABCPRED	outside

899	906	AMQMAYRF	0.9776	8	COVIDep	outside
900	911	MQMAYRFNGIGV	1.3524	12	ABCPRED	outside
900	915	MQMAYRFNGIGVTQNV	1.1008	16	ABCPRED	outside
901	912	QMAYRFNGIGVT	1.4219	12	ABCPRED	outside
902	913	MAYRFNGIGVTQ	1.3822	12	ABCPRED	outside
903	914	AYRFNGIGVTQN	1.2149	12	ABCPRED	outside
904	915	YRFNGIGVTQNV	1.1043	12	ABCPRED	outside
904	915	YRFNGIGVTQNV	1.1043	12	FBCPred	outside
1027	1042	TKMSECVLGQSKRVDF	1.0151	16	ABCPRED	outside
1029	1042	MSECVLGQSKRVDF	1.0345	14	ABCPRED	outside
1030	1045	SECVLGQSKRVDFCGK	1.0135	16	ABCPRED	outside
1031	1048	ECVLGQSKRVDFCGKGYH	1.009	18	ABCPRED	outside
1031	1044	ECVLGQSKRVDFCG	0.9433	14	ABCPRED	outside
1031	1039	ECVLGQSKR	0.9377	9	BCEPRED	outside
1032	1049	CVLGQSKRVDFCGKGYHL	1.1216	18	ABCPRED	outside
1032	1047	CVLGQSKRVDFCGKGY	1.0648	16	ABCPRED	outside
1033	1046	VLGQSKRVDFCGKG	1.3582	14	ABCPRED	outside
1033	1046	VLGQSKRVDFCGKG	1.3582	14	IEDB	outside
1033	1050	VLGQSKRVDFCGKGYHLM	1.0561	18	ABCPRED	outside
1034	1049	LGQSKRVDFCGKGYHL	1.2157	16	ABCPRED	outside
1035	1042	GQSKRVDF	1.9298	8	BCEPRED	outside
1035	1043	GQSKRVDFC	1.779	9	IEDB	outside
1035	1048	GQSKRVDFCGKGYH	1.1237	14	ABCPRED	outside
1035	1052	GQSKRVDFCGKGYHLMSF	0.9773	18	ABCPRED	outside
1037	1052	SKRVDFCGKGYHLMSF	1.0195	16	ABCPRED	outside
1038	1049	KRVDFCGKGYHL	1.3047	12	BCPREDS	outside
1057	1069	PHGVVFLHVTYVP	0.9305	13	BCEPRED	outside
1057	1074	PHGVVFLHVTYVPAQEKN	0.9072	18	ABCPRED	outside
1059	1076	GVVFLHVTYVPAQEKNFT	1.1197	18	ABCPRED	outside
1059	1072	GVVFLHVTYVPAQE	1.0085	14	ABCPRED	outside
1060	1075	VVFLHVTYVPAQEKNF	1.1471	16	ABCPRED	outside
1060	1079	VVFLHVTYVPAQEKNFTTAP	0.9571	20	ABCPRED	outside
1060	1077	VVFLHVTYVPAQEKNFTT	0.9224	18	ABCPRED	outside
1062	1077	FLHVTYVPAQEKNFTT	0.9556	16	ABCPRED	outside
1062	1081	FLHVTYVPAQEKNFTTAPAI	0.9351	20	AAP	outside
1063	1076	LHVTYVPAQEKNFT	1.2382	14	ABCPRED	outside
1063	1078	LHVTYVPAQEKNFTTA	1.0276	16	ABCPRED	outside
1063	1080	LHVTYVPAQEKNFTTAPA	0.9567	18	BCPREDS	outside
1063	1080	LHVTYVPAQEKNFTTAPA	0.9567	18	FBCPred	outside
1063	1082	LHVTYVPAQEKNFTTAPAIC	0.9251	20	BCPREDS	outside
1064	1075	HVTYVPAQEKNF	1.1353	12	AAP	outside
1064	1075	HVTYVPAQEKNF	1.1353	12	BCPREDS	outside
1064	1075	HVTYVPAQEKNF	1.1353	12	FBCPred	outside
1068	1076	VPAQEKNFT	1.0107	9	IEDB	outside
1155	1170	YFKNHTSPDVDLGDIS	1.1715	16	ABCPRED	outside
1156	1168	FKNHTSPDVDLGD	1.0616	13	IEDB	outside
1156	1173	FKNHTSPDVDLGDISGIN	0.9468	18	ABCPRED	outside
1156	1175	FKNHTSPDVDLGDISGINAS	0.9142	20	ABCPRED	outside
1157	1167	KNHTSPDVDLG	1.4039	11	IEDB	outside
1157	1173	KNHTSPDVDLGDISGIN	1.1116	17	COVIDep	outside
1157	1174	KNHTSPDVDLGDISGINA	1.0631	18	ABCPRED	outside
1158	1173	NHTSPDVDLGDISGIN	1.1399	16	ABCPRED	outside
1158	1171	NHTSPDVDLGDISG	1.0939	14	FBCPred	outside
1159	1180	HTSPDVDLGDISGINASVVNIQ	1.0856	22	FBCPred	outside
1159	1174	HTSPDVDLGDISGINA	1.0848	16	ABCPRED	outside
1159	1172	HTSPDVDLGDISGI	1.0779	14	AAP	outside
1159	1178	HTSPDVDLGDISGINASVVN	0.9177	20	AAP	outside
1159	1176	HTSPDVDLGDISGINASV	0.9036	18	ABCPRED	outside
1160	1175	TSPDVDLGDISGINAS	1.0756	16	AAP	outside
1160	1177	TSPDVDLGDISGINASVV	0.9371	18	ABCPRED	outside
1161	1180	SPDVDLGDISGINASVVNIQ	1.0657	20	ABCPRED	outside
1162	1173	PDVDLGDISGIN	1.2343	12	FBCPred	outside
1162	1179	PDVDLGDISGINASVVNI	1.0023	18	ABCPRED	outside
1162	1181	PDVDLGDISGINASVVNIQK	0.9428	20	FBCPred	outside
1163	1180	DVDLGDISGINASVVNIQ	1.3153	18	FBCPred	outside
1163	1178	DVDLGDISGINASVVN	1.1317	16	ABCPRED	outside
1164	1179	VDLGDISGINASVVNI	1.0784	16	ABCPRED	outside
1165	1180	DLGDISGINASVVNIQ	1.0543	16	FBCPred	outside
1165	1182	DLGDISGINASVVNIQKE	0.9087	18	ABCPRED	outside
1196	1215	SLIDLQELGKYEQYIKWPWY	1.0282	20	ABCPRED	transmembrane
1196	1215	SLIDLQELGKYEQYIKWPWY	1.0282	20	AAP	transmembrane
1197	1216	LIDLQELGKYEQYIKWPWYI	0.9949	20	ABCPRED	transmembrane
1198	1215	IDLQELGKYEQYIKWPWY	1.0558	18	ABCPRED	transmembrane
1198	1219	IDLQELGKYEQYIKWPWYIWLG	0.9637	22	BCPREDS	transmembrane
1199	1220	DLQELGKYEQYIKWPWYIWLGF	0.9678	22	FBCPred	transmembrane
1201	1220	QELGKYEQYIKWPWYIWLGF	0.9359	20	ABCPRED	transmembrane
1203	1222	LGKYEQYIKWPWYIWLGFIA	0.9901	20	ABCPRED	transmembrane
1204	1219	GKYEQYIKWPWYIWLG	0.9643	16	ABCPRED	transmembrane
1205	1220	KYEQYIKWPWYIWLGF	1.1402	16	ABCPRED	transmembrane
1205	1222	KYEQYIKWPWYIWLGFIA	1.0289	18	ABCPRED	transmembrane
1206	1221	YEQYIKWPWYIWLGFI	0.951	16	ABCPRED	transmembrane
1207	1222	EQYIKWPWYIWLGFIA	1.0127	16	ABCPRED	transmembrane
1210	1227	IKWPWYIWLGFIAGLIAI	0.9041	18	ABCPRED	transmembrane
1256	1273	FDEDDSEPVLKGVKLHYT	0.9755	18	AAP	inside

1257	1272	DEDDSEPVLKGVKLHY	1.0101	16	ABCPRED	inside
1258	1273	EDDSEPVLKGVKLHYT	1.125	16	ABCPRED	inside
1258	1273	EDDSEPVLKGVKLHYT	1.125	16	AAP	inside
1259	1273	DDSEPVLKGVKLHYT	1.1849	15	COVIDep	inside
1262	1273	EPVLKGVKLHYT	1.4118	12	BCEPRED	inside
1262	1270	EPVLKGVKL	1.2301	9	IEDB	inside

Supplementary Table 3. Unique list of predicted B-cell epitopes.

start	end	peptide	Antigenicity (VaxiJen)	Peptide Length	methods	transmembrane topology (TMHMM v2.0)	Toxicity (ToxinPred)	Peaks overlapped with RBD and Discontinuous epitope enriched regions
7	18	LLPLVSSQCVNL	1.0673	12	ABCPRED	outside	Non-toxin	No
9	18	PLVSSQCVNL	1.0958	10	ABCPRED	outside	Non-toxin	No
8	19	LPLVSSQCVNLT	1.4488	12	ABCPRED	outside	Non-toxin	No
10	19	LVSSQCVNLT	1.3797	10	ABCPRED	outside	Non-toxin	No
11	20	VSSQCVNLTT	1.2691	10	ABCPRED	outside	Non-toxin	No
8	21	LPLVSSQCVNLTTR	1.3565	14	ABCPRED	outside	Non-toxin	No
10	21	LVSSQCVNLTTR	1.2957	12	ABCPRED	outside	Non-toxin	No
5	22	LVLLPLVSSQCVNLTTRT	1.1125	18	ABCPRED	outside	Non-toxin	No
9	22	PLVSSQCVNLTTRT	1.2427	14	ABCPRED,FBCPred	outside	Non-toxin	No
4	23	FLVLLPLVSSQCVNLTTRTQ	1.0702	20	ABCPRED	outside	Non-toxin	No
8	23	LPLVSSQCVNLTTRTQ	1.3416	16	ABCPRED,FBCPred	outside	Non-toxin	No
10	23	LVSSQCVNLTTRTQ	1.2893	14	ABCPRED	outside	Non-toxin	No
12	23	SSQCVNLTTRTQ	1.5168	12	ABCPRED	outside	Non-toxin	No
14	23	QCVNLTTRTQ	1.7824	10	ABCPRED	outside	Non-toxin	No
7	24	LLPLVSSQCVNLTTRTQL	1.1517	18	ABCPRED	outside	Non-toxin	No
8	25	LPLVSSQCVNLTTRTQLP	1.0804	18	ABCPRED	outside	Non-toxin	No
10	25	LVSSQCVNLTTRTQLP	0.999	16	ABCPRED	outside	Non-toxin	No
14	25	QCVNLTTRTQLP	1.2589	12	ABCPRED	outside	Non-toxin	No
11	26	VSSQCVNLTTRTQLPP	1.409	16	ABCPRED	outside	Non-toxin	No
15	26	CVNLTTRTQLPP	1.649	12	BCPREDS,FBCPred	outside	Non-toxin	No
17	26	NLTTRTQLPP	1.2832	10	ABCPRED	outside	Non-toxin	No
10	27	LVSSQCVNLTTRTQLPPA	1.2068	18	ABCPRED	outside	Non-toxin	No
14	27	QCVNLTTRTQLPPA	1.4983	14	BCPREDS	outside	Non-toxin	No
13	30	SQCVNLTTRTQLPPAYTN	1.2205	18	ABCPRED	outside	Toxin	NA
16	27	VNLTTRTQLPPA	1.4268	12	ABCPRED	outside	Non-toxin	No
18	27	LTTRTQLPPA	1.3398	10	ABCPRED	outside	Non-toxin	No
9	28	PLVSSQCVNLTTRTQLPPAY	1.2186	20	ABCPRED	outside	Non-toxin	No
11	28	VSSQCVNLTTRTQLPPAY	1.3205	18	ABCPRED	outside	Non-toxin	No
15	28	CVNLTTRTQLPPAY	1.483	14	ABCPRED	outside	Non-toxin	No
19	28	TTRTQLPPAY	1.2001	10	ABCPRED	outside	Non-toxin	No
10	29	LVSSQCVNLTTRTQLPPAYT	1.1791	20	ABCPRED	outside	Non-toxin	No
14	29	QCVNLTTRTQLPPAYT	1.4165	16	ABCPRED	outside	Non-toxin	No
20	29	TRTQLPPAYT	1.2416	10	ABCPRED	outside	Non-toxin	No
17	30	NLTTRTQLPPAYTN	1.1058	14	ABCPRED	outside	Non-toxin	No
12	31	SSQCVNLTTRTQLPPAYTNS	1.1345	20	ABCPRED	outside	Non-toxin	No
16	31	VNLTTRTQLPPAYTNS	1.1248	16	ABCPRED	outside	Non-toxin	No
20	31	TRTQLPPAYTNS	0.9332	12	ABCPRED	outside	Non-toxin	No
13	32	SQCVNLTTRTQLPPAYTNSF	0.9351	20	ABCPRED	outside	Non-toxin	No
14	33	QCVNLTTRTQLPPAYTNSFT	0.9669	20	ABCPRED	outside	Non-toxin	No
21	30	RTQLPPAYTN	1.1291	10	ABCPRED	outside	Toxin	NA
34	41	RGVYYPDK	1.0191	8	IEDB	outside	Non-toxin	No
61	70	NVTWFHAIHV	0.9051	10	ABCPRED	outside	Non-toxin	No
70	78	VSGTNGTKR	0.9855	9	BCEPRED	outside	Non-toxin	No
90	97	VYFASTEK	0.9206	8	BCEPRED	outside	Non-toxin	No
108	115	TTLDSKTQ	1.0912	8	BCEPRED	outside	Non-toxin	No
108	116	TTLDSKTQS	1.0106	9	IEDB	outside	Non-toxin	No
108	117	TTLDSKTQSL	0.99	10	BCEPRED	outside	Non-toxin	No
110	121	LDSKTQSLLIVN	0.9139	12	ABCPRED	outside	Non-toxin	No
138	147	DPFLGVYYHK	0.9542	10	ABCPRED	outside	Non-toxin	No
137	148	NDPFLGVYYHKN	0.9849	12	ABCPRED	outside	Non-toxin	No
141	150	LGVYYHKNNK	0.9727	10	ABCPRED	outside	Non-toxin	No
174	183	PFLMDLEGKQ	0.915	10	ABCPRED	outside	Non-toxin	No
168	185	FEYVSQPFLMDLEGKQGN	0.9891	18	ABCPRED	outside	Non-toxin	No
174	185	PFLMDLEGKQGN	1.2067	12	ABCPRED	outside	Non-toxin	No
172	185	SQPFLMDLEGKQGN	0.9603	14	ABCPRED	outside	Toxin	NA
172	187	SQPFLMDLEGKQGNFK	0.9284	16	ABCPRED	outside	Toxin	NA
167	186	TFEYVSQPFLMDLEGKQGNF	1.009	20	ABCPRED	outside	Non-toxin	No
171	186	VSQPFLMDLEGKQGNF	0.9279	16	ABCPRED	outside	Non-toxin	No
177	186	MDLEGKQGNF	1.7852	10	ABCPRED	outside	Non-toxin	No
174	189	PFLMDLEGKQGNFKNL	1.0418	16	ABCPRED	outside	Toxin	NA
174	187	PFLMDLEGKQGNFK	1.1174	14	ABCPRED	outside	Non-toxin	No
178	187	DLEGKQGNFK	1.3065	10	ABCPRED	outside	Non-toxin	No
180	188	EGKQGNFKN	1.1232	9	IEDB	outside	Non-toxin	No
181	188	GKQGNFKN	1.0999	8	IEDB	outside	Non-toxin	No
177	189	MDLEGKQGNFKNL	1.2592	13	IEDB	outside	Non-toxin	No
180	190	EGKQGNFKNLR	1.0042	11	IEDB	outside	Non-toxin	No
201	208	FKIYSKHT	1.1661	8	BCEPRED	outside	Non-toxin	No
199	212	GYFKIYSKHTPINL	0.9308	14	ABCPRED	outside	Non-toxin	No
201	212	FKIYSKHTPINL	1.1557	12	ABCPRED	outside	Non-toxin	No
203	212	IYSKHTPINL	1.2064	10	ABCPRED	outside	Non-toxin	No
204	213	YSKHTPINLV	1.0391	10	ABCPRED	outside	Non-toxin	No
201	214	FKIYSKHTPINLVR	0.9156	14	ABCPRED	outside	Non-toxin	No
213	226	VRDLPQGFSALEPL	0.9407	14	ABCPRED	outside	Non-toxin	No
223	236	LEPLVDLPIGINIT	1.1627	14	ABCPRED	outside	Non-toxin	No
225	236	PLVDLPIGINIT	1.3843	12	ABCPRED	outside	Non-toxin	No
224	237	EPLVDLPIGINITR	0.9556	14	ABCPRED	outside	Non-toxin	No
228	239	DLPIGINITRFQ	1.3916	12	ABCPRED	outside	Non-toxin	No
227	240	VDLPIGINITRFQT	0.9138	14	ABCPRED	outside	Non-toxin	No
229	240	LPIGINITRFQT	0.9919	12	ABCPRED	outside	Non-toxin	No
228	245	DLPIGINITRFQTLLALH	0.9638	18	ABCPRED	outside	Toxin	NA
228	241	DLPIGINITRFQTL	1.1058	14	ABCPRED	outside	Non-toxin	No
230	241	PIGINITRFQTL	1.0169	12	ABCPRED	outside	Non-toxin	No
258	273	WTAGAAAYYVGYLQPR	0.9545	16	ABCPRED	outside	Non-toxin	No
260	273	AGAAAYYVGYLQPR	1.0663	14	ABCPRED	outside	Non-toxin	No
262	273	AAAYYVGYLQPR	1.0262	12	ABCPRED	outside	Non-toxin	No

264	273	AYYVGYLQPR	1.3309	10	ABCPRED	outside	Non-toxin	No
265	273	YYVGYLQPR	1.4692	9	BCEPRED	outside	Non-toxin	No
263	274	AAYYVGYLQPRT	0.9255	12	BCPREDS	outside	Non-toxin	No
278	287	KYNENGTITD	0.9589	10	BCEPRED	outside	Non-toxin	No
291	300	CALDPLSETK	0.9226	10	ABCPRED	outside	Non-toxin	No
293	302	LDPLSETKCT	1.2586	10	ABCPRED	outside	Non-toxin	No
295	302	PLSETKCT	1.2573	8	IEDB	outside	Non-toxin	No
293	304	LDPLSETKCTLK	0.9712	12	ABCPRED	outside	Non-toxin	No
292	305	ALDPLSETKCTLKS	1.017	14	ABCPRED	outside	Non-toxin	No
294	305	DPLSETKCTLKS	0.9204	12	FBCPred,IEDB	outside	Non-toxin	No
296	305	LSETKCTLKS	0.9434	10	ABCPRED	outside	Non-toxin	No
311	322	GIYQTSNFRVQP	0.9986	12	ABCPRED	outside	Non-toxin	No
313	322	YQTSNFRVQP	1.1866	10	ABCPRED	outside	Non-toxin	No
310	323	KGIYQTSNFRVQPT	0.962	14	ABCPRED	outside	Non-toxin	No
312	323	IYQTSNFRVQPT	1.1283	12	ABCPRED	outside	Non-toxin	No
314	323	QTSNFRVQPT	1.405	10	ABCPRED,IEDB	outside	Non-toxin	No
315	324	TSNFRVQPTE	1.3571	10	ABCPRED	outside	Non-toxin	No
316	325	SNFRVQPTES	1.2078	10	IEDB	outside	Non-toxin	No
315	326	TSNFRVQPTESI	0.9265	12	ABCPRED	outside	Non-toxin	No
317	326	NFRVQPTESI	1.2412	10	ABCPRED	outside	Non-toxin	No
327	336	VRFPNITNLC	1.1924	10	ABCPRED	outside	Non-toxin	No
326	337	IVRFPNITNLCP	0.9596	12	ABCPRED	outside	Non-toxin	No
328	337	RFPNITNLCP	1.4779	10	ABCPRED	outside	Non-toxin	No
327	338	VRFPNITNLCPF	1.2901	12	ABCPRED	outside	Non-toxin	No
330	339	PNITNLCPFG	1.4887	10	ABCPRED	outside	Non-toxin	No
327	340	VRFPNITNLCPFGE	1.0597	14	ABCPRED	outside	Non-toxin	No
329	340	FPNITNLCPFGE	1.0991	12	ABCPRED	outside	Non-toxin	No
331	340	NITNLCPFGE	1.237	10	ABCPRED	outside	Non-toxin	No
375	384	STFKCYGVSP	1.104	10	ABCPRED	outside	Non-toxin	Peak1
376	385	TFKCYGVSPT	1.5059	10	ABCPRED,IEDB	outside	Non-toxin	Peak1
371	386	SASFSTFKCYGVSPTK	0.908	16	ABCPRED	outside	Non-toxin	Peak1
373	386	SFSTFKCYGVSPTK	0.9678	14	ABCPRED	outside	Non-toxin	Peak1
372	387	ASFSTFKCYGVSPTKL	0.912	16	ABCPRED	outside	Non-toxin	Peak1
376	387	TFKCYGVSPTKL	1.4293	12	ABCPRED	outside	Non-toxin	Peak1
371	388	SASFSTFKCYGVSPTKLN	0.9891	18	ABCPRED	outside	Non-toxin	Peak1
375	388	STFKCYGVSPTKLN	1.2695	14	AAP	outside	Non-toxin	Peak1
379	388	CYGVSPTKLN	1.5759	10	ABCPRED	outside	Non-toxin	Peak1
381	388	GVSPTKLN	1.9197	8	IEDB	outside	Non-toxin	Peak1
374	389	FSTFKCYGVSPTKLND	0.965	16	ABCPRED	outside	Non-toxin	Peak1
378	389	KCYGVSPTKLND	1.3195	12	ABCPRED,BCPREDS	outside	Non-toxin	Peak1
380	389	YGVSPTKLND	1.4531	10	IEDB	outside	Non-toxin	Peak1
369	390	YNSASFSTFKCYGVSPTKLNDL	0.9508	22	AAP	outside	Non-toxin	Peak1
375	390	STFKCYGVSPTKLNDL	1.2492	16	ABCPRED	outside	Non-toxin	Peak1
377	390	FKCYGVSPTKLNDL	1.4748	14	ABCPRED	outside	Non-toxin	Peak1
379	390	CYGVSPTKLNDL	1.4808	12	ABCPRED	outside	Non-toxin	Peak1
381	390	GVSPTKLNDL	1.6525	10	ABCPRED	outside	Non-toxin	Peak1
383	390	SPTKLNDL	1.0358	8	BCEPRED	outside	Non-toxin	Peak1
372	391	ASFSTFKCYGVSPTKLNDLC	1.227	20	ABCPRED	outside	Non-toxin	Peak1
374	391	FSTFKCYGVSPTKLNDLC	1.2396	18	ABCPRED	outside	Non-toxin	Peak1
380	391	YGVSPTKLNDLC	1.8347	12	ABCPRED,AAP	outside	Non-toxin	Peak1
382	391	VSPTKLNDLC	1.9573	10	ABCPRED	outside	Non-toxin	Peak1
375	392	STFKCYGVSPTKLNDLCF	1.7606	18	ABCPRED	outside	Non-toxin	Peak1
377	392	FKCYGVSPTKLNDLCF	2.0332	16	ABCPRED	outside	Non-toxin	Peak1
383	392	SPTKLNDLCF	2.2412	10	ABCPRED	outside	Non-toxin	Peak1
369	393	YNSASFSTFKCYGVSPTKLNDLCFT	1.4031	25	IEDB	outside	Non-toxin	Peak1
378	393	KCYGVSPTKLNDLCFT	2.0395	16	ABCPRED	outside	Non-toxin	Peak1
382	393	VSPTKLNDLCFT	2.4268	12	ABCPRED	outside	Non-toxin	Peak1
384	393	PTKLNDLCFT	2.5246	10	ABCPRED	outside	Non-toxin	Peak1
377	394	FKCYGVSPTKLNDLCFTN	1.9296	18	ABCPRED	outside	Non-toxin	Peak1
379	394	CYGVSPTKLNDLCFTN	2.0134	16	ABCPRED	outside	Non-toxin	Peak1
378	395	KCYGVSPTKLNDLCFTNV	1.8687	18	ABCPRED	outside	Non-toxin	Peak1
380	395	YGVSPTKLNDLCFTNV	2.0255	16	ABCPRED	outside	Non-toxin	Peak1
386	395	KLNDLCFTNV	2.6927	10	ABCPRED	outside	Non-toxin	Peak1
379	396	CYGVSPTKLNDLCFTNVY	1.7815	18	ABCPRED	outside	Non-toxin	Peak1
381	396	GVSPTKLNDLCFTNVY	1.9127	16	ABCPRED	outside	Non-toxin	Peak1
383	396	SPTKLNDLCFTNVY	1.6982	14	ABCPRED	outside	Non-toxin	Peak1
385	396	TKLNDLCFTNVY	2.0035	12	ABCPRED	outside	Non-toxin	Peak1
387	396	LNDLCFTNVY	1.6134	10	ABCPRED	outside	Non-toxin	Peak1
389	396	DLCFTNVY	1.8569	8	BCEPRED	outside	Non-toxin	Peak1
378	397	KCYGVSPTKLNDLCFTNVYA	1.5664	20	ABCPRED,AAP	outside	Non-toxin	Peak1
379	398	CYGVSPTKLNDLCFTNVYAD	1.5222	20	ABCPRED	outside	Non-toxin	Peak1
383	398	SPTKLNDLCFTNVYAD	1.3666	16	ABCPRED	outside	Non-toxin	Peak1
380	399	YGVSPTKLNDLCFTNVYADS	1.361	20	ABCPRED	outside	Non-toxin	Peak1
384	399	PTKLNDLCFTNVYADS	1.2303	16	ABCPRED	outside	Non-toxin	Peak1
381	400	GVSPTKLNDLCFTNVYADSF	1.2617	20	ABCPRED	outside	Non-toxin	Peak2
380	401	YGVSPTKLNDLCFTNVYADSFV	1.1404	22	BCPREDS	outside	Non-toxin	Peak2
383	402	SPTKLNDLCFTNVYADSFVI	0.9482	20	ABCPRED	outside	Non-toxin	Peak2
385	402	TKLNDLCFTNVYADSFVI	1.0271	18	ABCPRED	outside	Non-toxin	Peak2
384	403	PTKLNDLCFTNVYADSFVIR	0.9772	20	ABCPRED	outside	Non-toxin	Peak2
386	403	KLNDLCFTNVYADSFVIR	1.0694	18	ABCPRED	outside	Non-toxin	Peak2
385	404	TKLNDLCFTNVYADSFVIRG	1.049	20	ABCPRED	outside	Non-toxin	Peak2
386	405	KLNDLCFTNVYADSFVIRGD	0.932	20	ABCPRED	outside	Non-toxin	Peak2
406	415	EVRQIAPGQT	1.0655	10	ABCPRED	outside	Non-toxin	Peak2
405	416	DEVRQIAPGQTG	1.0397	12	ABCPRED	outside	Non-toxin	Peak2
407	416	VRQIAPGQTG	1.3856	10	ABCPRED	outside	Non-toxin	Peak2
404	417	GDEVRQIAPGQTGK	1.1397	14	BCPRED,AAP,FBCPr	outside	Non-toxin	Peak2
409	418	QIAPGQTGKI	1.4416	10	ABCPRED	outside	Non-toxin	Peak2
406	419	EVRQIAPGQTGKIA	1.3182	14	ABCPRED	outside	Non-toxin	Peak2
408	419	RQIAPGQTGKIA	1.4558	12	BCPREDS,FBCPred	outside	Non-toxin	Peak2

405	420	DEVRQIAPGQTGKIAD	1.0388	16	ABCPRED	outside	Non-toxin	Peak2
407	420	VRQIAPGQTGKIAD	1.2606	14	IEDB	outside	Non-toxin	Peak2
411	420	APGQTGKIAD	1.2753	10	ABCPRED	outside	Non-toxin	Peak2
406	421	EVRQIAPGQTGKIADY	1.3837	16	ABCPRED	outside	Non-toxin	Peak2
408	421	RQIAPGQTGKIADY	1.504	14	ABCPRED	outside	Non-toxin	Peak2
410	421	IAPGQTGKIADY	1.6626	12	ABCPRED	outside	Non-toxin	Peak2
407	422	VRQIAPGQTGKIADYN	1.3372	16	ABCPRED	outside	Non-toxin	Peak2
409	422	QIAPGQTGKIADYN	1.5448	14	ABCPRED	outside	Non-toxin	Peak2
404	423	GDEVRQIAPGQTGKIADYNY	1.219	20	ABCPRED	outside	Non-toxin	Peak2
406	423	EVRQIAPGQTGKIADYNY	1.4691	18	BCPREDS	outside	Non-toxin	Peak2
408	423	RQIAPGQTGKIADYNY	1.5799	16	ABCPRED	outside	Non-toxin	Peak2
410	423	IAPGQTGKIADYNY	1.724	14	ABCPRED,BCPREDS	outside	Non-toxin	Peak2
412	423	PGQTGKIADYNY	1.642	12	ABCPRED	outside	Non-toxin	Peak2
414	423	QTGKIADYNY	1.5116	10	ABCPRED	outside	Non-toxin	Peak2
413	424	GQTGKIADYNYK	1.7308	12	ABCPRED	outside	Non-toxin	Peak2
410	431	IAPGQTGKIADYNYKLPDDFTG	1.1317	22	BCPREDS	outside	Toxin	NA
410	425	IAPGQTGKIADYNYKL	1.6707	16	ABCPRED	outside	Non-toxin	Peak2
411	425	APGQTGKIADYNYKL	1.4441	15	IEDB	outside	Non-toxin	Peak2
414	425	QTGKIADYNYKL	1.4851	12	ABCPRED	outside	Non-toxin	Peak2
416	425	GKIADYNYKL	1.6079	10	ABCPRED	outside	Non-toxin	Peak2
404	426	GDEVRQIAPGQTGKIADYNYKLP	1.1017	23	IEDB	outside	Non-toxin	Peak2
407	426	VRQIAPGQTGKIADYNYKLP	1.2386	20	ABCPRED	outside	Non-toxin	Peak2
411	426	APGQTGKIADYNYKLP	1.2303	16	ABCPRED	outside	Non-toxin	Peak2
413	426	GQTGKIADYNYKLP	1.2756	14	ABCPRED	outside	Non-toxin	Peak2
415	426	TGKIADYNYKLP	1.1956	12	ABCPRED	outside	Non-toxin	Peak2
417	426	KIADYNYKLP	1.2565	10	ABCPRED	outside	Non-toxin	Peak2
408	427	RQIAPGQTGKIADYNYKLPD	1.4102	20	ABCPRED,BCPREDS	outside	Non-toxin	Peak2
412	427	PGQTGKIADYNYKLPD	1.4039	16	ABCPRED	outside	Non-toxin	Peak2
414	427	QTGKIADYNYKLPD	1.2934	14	ABCPRED	outside	Non-toxin	Peak2
416	427	GKIADYNYKLPD	1.3553	12	ABCPRED	outside	Non-toxin	Peak2
413	428	GQTGKIADYNYKLPDD	1.0071	16	ABCPRED	outside	Non-toxin	Peak2
414	429	QTGKIADYNYKLPDDF	0.986	16	ABCPRED	outside	Non-toxin	Peak2
416	429	GKIADYNYKLPDDF	0.9776	14	ABCPRED	outside	Non-toxin	Peak2
411	430	APGQTGKIADYNYKLPDDFT	1.0425	20	ABCPRED,IEDB	outside	Non-toxin	Peak2
413	430	GQTGKIADYNYKLPDDFT	1.0564	18	ABCPRED	outside	Non-toxin	Peak2
415	430	TGKIADYNYKLPDDFT	0.9642	16	ABCPRED	outside	Non-toxin	Peak2
417	430	KIADYNYKLPDDFT	0.9567	14	BCEPRED	outside	Non-toxin	Peak2
412	431	PGQTGKIADYNYKLPDDFTG	1.0123	20	ABCPRED	outside	Non-toxin	Peak2
433	442	VIAWNSNNLD	0.9177	10	ABCPRED	outside	Non-toxin	Peak2
435	444	AWNSNNLDSK	1.0198	10	BCEPRED	outside	Non-toxin	Peak2
439	446	NNLDSKVG	1.2952	8	BCEPRED	outside	Non-toxin	Peak2
434	449	IAWNSNNLDSKVGGNY	0.906	16	ABCPRED	outside	Non-toxin	Peak2
441	450	LDSKVGGNYN	0.91	10	ABCPRED	outside	Non-toxin	Peak2
434	451	IAWNSNNLDSKVGGNYNY	1.0683	18	ABCPRED	outside	Non-toxin	Peak2
456	463	FRKSNLKP	1.1111	8	BCEPRED	outside	Non-toxin	Peak3
482	493	GVEGFNCYFPLQ	0.9537	12	ABCPRED	outside	Non-toxin	Peak3
488	495	CYFPLQSY	0.9394	8	IEDB	outside	Non-toxin	Peak3
485	500	GFNCYFPLQSYGFQPT	0.981	16	ABCPRED	outside	Non-toxin	Peak3
482	501	GVEGFNCYFPLQSYGFQPTN	0.9121	20	ABCPRED	outside	Non-toxin	Peak3
486	501	FNCYFPLQSYGFQPTN	1.0229	16	ABCPRED	outside	Non-toxin	Peak3
490	501	FPLQSYGFQPTN	0.9942	12	ABCPRED	outside	Non-toxin	Peak3
492	501	LQSYGFQPTN	1.043	10	ABCPRED	outside	Non-toxin	Peak3
493	502	QSYGFQPTNG	0.9097	10	ABCPRED	outside	Non-toxin	Peak3
494	503	SYGFQPTNGV	1.0549	10	ABCPRED	outside	Non-toxin	Peak3
502	511	GVGYQPYRVV	1.1915	10	ABCPRED	outside	Non-toxin	Peak3
503	511	VGYQPYRVV	1.4383	9	BCEPRED	outside	Non-toxin	Peak3
502	513	GVGYQPYRVVVL	1.1207	12	BCPREDS,FBCPred	outside	Non-toxin	Peak3
502	515	GVGYQPYRVVVLSF	1.2391	14	ABCPRED	outside	Non-toxin	Peak3
504	515	GYQPYRVVVLSF	1.1235	12	ABCPRED	outside	Non-toxin	Peak3
501	516	NGVGYQPYRVVVLSFE	0.9736	16	ABCPRED,BCPREDS	outside	Non-toxin	Peak3
505	516	YQPYRVVVLSFE	0.9911	12	ABCPRED	outside	Non-toxin	Peak3
506	517	QPYRVVVLSFEL	0.9785	12	ABCPRED	outside	Non-toxin	Peak3
508	517	YRVVVLSFEL	1.1494	10	ABCPRED	outside	Non-toxin	Peak3
505	519	YQPYRVVVLSFELLH	0.9711	15	BCEPRED	outside	Non-toxin	Peak3
506	519	QPYRVVVLSFELLH	0.9675	14	ABCPRED	outside	Non-toxin	Peak3
508	519	YRVVVLSFELLH	1.1085	12	ABCPRED	outside	Non-toxin	Peak3
501	520	NGVGYQPYRVVVLSFELLHA	0.9212	20	ABCPRED	outside	Non-toxin	Peak3
507	520	PYRVVVLSFELLHA	1.0185	14	ABCPRED	outside	Non-toxin	Peak3
503	522	VGYQPYRVVVLSFELLHAPA	0.989	20	ABCPRED	outside	Non-toxin	Peak3
513	524	LSFELLHAPATV	0.9969	12	ABCPRED	outside	Non-toxin	Peak4
528	541	KKSTNLVKNKCVNF	1.0894	14	ABCPRED	outside	Non-toxin	Peak4
526	543	GPKKSTNLVKNKCVNFNF	1.1626	18	ABCPRED	outside	Non-toxin	Peak4
530	543	STNLVKNKCVNFNF	1.289	14	ABCPRED	outside	Non-toxin	Peak4
532	543	NLVKNKCVNFNF	1.3432	12	ABCPRED	outside	Non-toxin	Peak4
529	544	KSTNLVKNKCVNFNFN	1.4412	16	ABCPRED	outside	Non-toxin	Peak4
531	544	TNLVKNKCVNFNFN	1.2489	14	ABCPRED,BCPREDS	outside	Non-toxin	Peak4
533	544	LVKNKCVNFNFN	1.6246	12	FBCPred	outside	Non-toxin	Peak4
535	544	KNKCVNFNFN	2.2331	10	ABCPRED	outside	Non-toxin	Peak4
536	545	NKCVNFNFNG	1.3088	10	ABCPRED	outside	Non-toxin	Peak4
527	546	PKKSTNLVKNKCVNFNFNGL	1.0582	20	ABCPRED	outside	Non-toxin	Peak4
529	546	KSTNLVKNKCVNFNFNGL	1.22	18	ABCPRED	outside	Non-toxin	Peak4
533	546	LVKNKCVNFNFNGL	1.3128	14	FBCPred	outside	Non-toxin	Peak4
535	546	KNKCVNFNFNGL	1.7395	12	ABCPRED	outside	Non-toxin	Peak4
538	546	CVNFNFNGL	1.7985	9	BCEPRED	outside	Non-toxin	Peak4
528	547	KKSTNLVKNKCVNFNFNGLT	1.0818	20	ABCPRED	outside	Non-toxin	Peak4
530	547	STNLVKNKCVNFNFNGLT	0.9604	18	ABCPRED	outside	Non-toxin	Peak4
534	547	VKNKCVNFNFNGLT	1.5749	14	ABCPRED	outside	Non-toxin	Peak4
535	548	KNKCVNFNFNGLTG	1.6052	14	ABCPRED	outside	Non-toxin	Peak4
537	548	KCVNFNFNGLTG	1.6969	12	IEDB	outside	Non-toxin	Peak4

530	549	STNLVKNKCVNFNFNGLTGT	1.0756	20	ABCPRED	outside	Non-toxin	Peak4
536	549	NKCVNFNFNGLTGT	1.3691	14	ABCPRED	outside	Non-toxin	Peak4
533	550	LVKNKCVNFNFNGLTGTG	1.2501	18	ABCPRED	outside	Non-toxin	Peak4
537	550	KCVNFNFNGLTGTG	1.5894	14	ABCPRED	outside	Non-toxin	Peak4
541	550	FNFNGLTGTG	1.0567	10	ABCPRED	outside	Non-toxin	Peak4
530	551	STNLVKNKCVNFNFNGLTGTGV	1.0662	22	BCPREDS	outside	Non-toxin	Peak4
532	551	NLVKNKCVNFNFNGLTGTGV	1.084	20	ABCPRED	outside	Non-toxin	Peak4
540	551	NFNFNGLTGTGV	1.3547	12	ABCPRED,BCPREDS	outside	Non-toxin	Peak4
535	552	KNKCVNFNFNGLTGTGVL	1.3849	18	ABCPRED	outside	Non-toxin	Peak4
537	552	KCVNFNFNGLTGTGVL	1.4136	16	AAP	outside	Non-toxin	Peak4
539	552	VNFNFNGLTGTGVL	1.2915	14	ABCPRED	outside	Non-toxin	Peak4
540	553	NFNFNGLTGTGVLT	1.1216	14	ABCPRED	outside	Non-toxin	Peak4
535	554	KNKCVNFNFNGLTGTGVLTE	1.2763	20	ABCPRED	outside	Non-toxin	Peak4
539	554	VNFNFNGLTGTGVLTE	1.1691	16	ABCPRED	outside	Non-toxin	Peak4
536	555	NKCVNFNFNGLTGTGVLTES	1.0131	20	ABCPRED	outside	Non-toxin	Peak4
538	555	CVNFNFNGLTGTGVLTES	1.1292	18	ABCPRED	outside	Non-toxin	Peak4
537	556	KCVNFNFNGLTGTGVLTESN	1.2355	20	ABCPRED	outside	Non-toxin	Peak4
538	557	CVNFNFNGLTGTGVLTESNK	1.1352	20	ABCPRED	outside	Non-toxin	Peak4
539	558	VNFNFNGLTGTGVLTESNKK	1.1457	20	ABCPRED	outside	Non-toxin	Peak4
541	558	FNFNGLTGTGVLTESNKK	0.9066	18	ABCPRED	outside	Non-toxin	Peak4
545	558	GLTGTGVLTESNKK	1.0227	14	IEDB	outside	Non-toxin	Peak4
540	559	NFNFNGLTGTGVLTESNKKF	0.9432	20	ABCPRED	outside	Non-toxin	Peak4
547	562	TGTGVLTESNKKFLPF	0.9925	16	ABCPRED	outside	Non-toxin	No
555	562	SNKKFLPF	1.3952	8	IEDB	outside	Non-toxin	No
546	563	LTGTGVLTESNKKFLPFQ	0.941	18	ABCPRED	outside	Non-toxin	No
553	563	TESNKKFLPFQ	0.9243	11	ellipro	outside	Non-toxin	No
555	564	SNKKFLPFQQ	0.9488	10	ABCPRED	outside	Non-toxin	No
577	584	RDPQTLEI	0.9248	8	ellipro	outside	Non-toxin	No
577	586	RDPQTLEILD	1.0339	10	ABCPRED	outside	Non-toxin	No
578	587	DPQTLEILDI	1.5757	10	ABCPRED	outside	Non-toxin	No
573	588	TDAVRDPQTLEILDIT	0.9832	16	ABCPRED	outside	Non-toxin	No
568	589	DIADTTDAVRDPQTLEILDITP	1.191	22	FBCPred	outside	Non-toxin	No
572	589	TTDAVRDPQTLEILDITP	1.1067	18	AAP	outside	Non-toxin	No
574	589	DAVRDPQTLEILDITP	1.1133	16	ABCPRED	outside	Non-toxin	No
576	589	VRDPQTLEILDITP	1.4571	14	ABCPRED	outside	Non-toxin	No
580	589	QTLEILDITP	2.027	10	ABCPRED	outside	Non-toxin	No
577	590	RDPQTLEILDITPC	1.2642	14	ABCPRED	outside	Non-toxin	No
579	590	PQTLEILDITPC	1.4158	12	ABCPRED	outside	Non-toxin	No
578	591	DPQTLEILDITPCS	1.252	14	ABCPRED	outside	Non-toxin	No
580	591	QTLEILDITPCS	1.2575	12	ABCPRED	outside	Non-toxin	No
579	592	PQTLEILDITPCSF	1.3489	14	ABCPRED	outside	Non-toxin	No
583	592	EILDITPCSF	1.2698	10	ABCPRED	outside	Non-toxin	No
582	593	LEILDITPCSFG	1.759	12	ABCPRED	outside	Non-toxin	No
584	593	ILDITPCSFG	1.5391	10	ABCPRED	outside	Non-toxin	No
575	594	AVRDPQTLEILDITPCSFGG	1.1985	20	ABCPRED	outside	Non-toxin	No
581	594	TLEILDITPCSFGG	1.5811	14	ABCPRED	outside	Non-toxin	No
574	595	DAVRDPQTLEILDITPCSFGGV	1.0597	22	AAP	outside	Non-toxin	No
578	595	DPQTLEILDITPCSFGGV	1.4948	18	ABCPRED	outside	Non-toxin	No
580	595	QTLEILDITPCSFGGV	1.5382	16	ABCPRED	outside	Non-toxin	No
584	595	ILDITPCSFGGV	1.5192	12	ABCPRED	outside	Non-toxin	No
577	596	RDPQTLEILDITPCSFGGVS	1.4479	20	ABCPRED	outside	Non-toxin	No
581	596	TLEILDITPCSFGGVS	1.6878	16	ABCPRED	outside	Non-toxin	No
583	596	EILDITPCSFGGVS	1.6193	14	ABCPRED	outside	Non-toxin	No
585	596	LDITPCSFGGVS	1.9283	12	ABCPRED	outside	Non-toxin	No
578	597	DPQTLEILDITPCSFGGVSV	1.5468	20	ABCPRED	outside	Non-toxin	No
580	597	QTLEILDITPCSFGGVSV	1.5916	18	ABCPRED	outside	Non-toxin	No
582	597	LEILDITPCSFGGVSV	1.7357	16	ABCPRED	outside	Non-toxin	No
584	597	ILDITPCSFGGVSV	1.6035	14	ABCPRED	outside	Non-toxin	No
579	598	PQTLEILDITPCSFGGVSVI	1.3978	20	ABCPRED	outside	Non-toxin	No
581	598	TLEILDITPCSFGGVSVI	1.4847	18	ABCPRED	outside	Non-toxin	No
583	598	EILDITPCSFGGVSVI	1.3971	16	ABCPRED	outside	Non-toxin	No
585	598	LDITPCSFGGVSVI	1.6124	14	ABCPRED	outside	Non-toxin	No
580	599	QTLEILDITPCSFGGVSVIT	1.3356	20	ABCPRED	outside	Non-toxin	No
582	599	LEILDITPCSFGGVSVIT	1.428	18	ABCPRED	outside	Non-toxin	No
584	599	ILDITPCSFGGVSVIT	1.2689	16	ABCPRED	outside	Non-toxin	No
588	599	TPCSFGGVSVIT	1.1714	12	ABCPRED	outside	Non-toxin	No
590	599	CSFGGVSVIT	1.0615	10	ABCPRED	outside	Non-toxin	No
581	600	TLEILDITPCSFGGVSVITP	1.2987	20	ABCPRED	outside	Non-toxin	No
585	600	LDITPCSFGGVSVITP	1.3502	16	ABCPRED	outside	Non-toxin	No
587	600	ITPCSFGGVSVITP	0.9725	14	ABCPRED	outside	Non-toxin	No
582	601	LEILDITPCSFGGVSVITPG	1.2631	20	ABCPRED	outside	Non-toxin	No
584	601	ILDITPCSFGGVSVITPG	1.1031	18	BCEPRED	outside	Non-toxin	No
588	601	TPCSFGGVSVITPG	0.9486	14	ABCPRED	outside	Non-toxin	No
583	602	EILDITPCSFGGVSVITPGT	1.0825	20	ABCPRED	outside	Non-toxin	No
585	602	LDITPCSFGGVSVITPGT	1.2006	18	ABCPRED	outside	Non-toxin	No
584	603	ILDITPCSFGGVSVITPGTN	0.9838	20	ABCPRED	outside	Non-toxin	No
586	603	DITPCSFGGVSVITPGTN	1.0022	18	ABCPRED	outside	Non-toxin	No
585	604	LDITPCSFGGVSVITPGTNT	1.0966	20	ABCPRED	outside	Non-toxin	No
586	605	DITPCSFGGVSVITPGTNTS	0.9823	20	ABCPRED	outside	Non-toxin	No
598	607	ITPGTNTSNQ	0.941	10	ABCPRED	outside	Non-toxin	No
607	620	QVAVLYQDVNCTEV	0.9497	14	ABCPRED	outside	Non-toxin	No
609	620	AVLYQDVNCTEV	0.9218	12	ABCPRED	outside	Non-toxin	No
612	623	YQDVNCTEVPVA	1.0646	12	AAP,FBCPred	outside	Non-toxin	No
614	623	DVNCTEVPVA	1.237	10	ABCPRED	outside	Non-toxin	No
615	624	VNCTEVPVAI	0.9137	10	ABCPRED	outside	Non-toxin	No
612	625	YQDVNCTEVPVAIH	1.0383	14	ABCPRED	outside	Non-toxin	No
629	638	LTPTWRVYST	1.1346	10	ABCPRED	outside	Non-toxin	No
628	639	QLTPTWRVYSTG	1.1857	12	ABCPRED	outside	Non-toxin	No
628	641	QLTPTWRVYSTGSN	0.9924	14	ABCPRED	outside	Non-toxin	No

637	650	STGSNVFQTRAGCL	0.9742	14	ABCPRED	outside	Non-toxin	No
639	650	GSNVFQTRAGCL	1.0436	12	ABCPRED	outside	Non-toxin	No
639	654	GSNVFQTRAGCLIGAE	0.9074	16	ABCPRED	outside	Non-toxin	No
638	655	TGSNVFQTRAGCLIGAEH	0.9162	18	ABCPRED	outside	Non-toxin	No
642	655	VFQTRAGCLIGAEH	1.2294	14	ABCPRED	outside	Non-toxin	No
643	656	FQTRAGCLIGAEHV	1.27	14	ABCPRED	outside	Non-toxin	No
640	657	SNVFQTRAGCLIGAEHVN	0.9399	18	ABCPRED	outside	Non-toxin	No
642	657	VFQTRAGCLIGAEHVN	1.2576	16	ABCPRED	outside	Non-toxin	No
644	657	QTRAGCLIGAEHVN	1.3917	14	ABCPRED	outside	Non-toxin	No
646	657	RAGCLIGAEHVN	1.3707	12	ABCPRED	outside	Non-toxin	No
643	658	FQTRAGCLIGAEHVNN	1.2294	16	ABCPRED	outside	Non-toxin	No
649	658	CLIGAEHVNN	1.1765	10	ABCPRED	outside	Non-toxin	No
642	659	VFQTRAGCLIGAEHVNNS	1.1368	18	ABCPRED	outside	Non-toxin	No
646	659	RAGCLIGAEHVNNS	1.1927	14	ABCPRED	outside	Non-toxin	No
650	659	LIGAEHVNNS	0.9735	10	ABCPRED	outside	Non-toxin	No
641	660	NVFQTRAGCLIGAEHVNNSY	0.9383	20	ABCPRED	outside	Non-toxin	No
646	661	RAGCLIGAEHVNNSYE	1.1613	16	ABCPRED	outside	Non-toxin	No
645	662	TRAGCLIGAEHVNNSYEC	1.1982	18	ABCPRED	outside	Non-toxin	No
647	662	AGCLIGAEHVNNSYEC	0.914	16	ABCPRED	outside	Non-toxin	No
654	662	EHVNNSYEC	1.068	9	IEDB	outside	Non-toxin	No
650	663	LIGAEHVNNSYECD	0.9242	14	ABCPRED	outside	Non-toxin	No
645	664	TRAGCLIGAEHVNNSYECDI	1.0613	20	ABCPRED	outside	Non-toxin	No
649	664	CLIGAEHVNNSYECDI	1.0413	16	ABCPRED	outside	Non-toxin	No
649	666	CLIGAEHVNNSYECDIPI	0.9191	18	ABCPRED,FBCPred	outside	Non-toxin	No
651	666	IGAEHVNNSYECDIPI	0.9115	16	FBCPred	outside	Non-toxin	No
648	667	GCLIGAEHVNNSYECDIPIG	0.9157	20	ABCPRED	outside	Non-toxin	No
650	667	LIGAEHVNNSYECDIPIG	0.9717	18	ABCPRED	outside	Non-toxin	No
649	668	CLIGAEHVNNSYECDIPIGA	1.0187	20	ABCPRED	outside	Non-toxin	No
651	668	IGAEHVNNSYECDIPIGA	1.0231	18	ABCPRED	outside	Non-toxin	No
652	669	GAEHVNNSYECDIPIGAG	0.9847	18	CPRED,AAP,BCPRE	outside	Non-toxin	No
651	670	IGAEHVNNSYECDIPIGAGI	1.1141	20	ABCPRED,BCPREDS	outside	Non-toxin	No
663	678	DIPIGAGICASYQTQT	1.0235	16	ABCPRED	outside	Non-toxin	No
669	678	GICASYQTQT	0.9671	10	ABCPRED	outside	Non-toxin	No
664	679	IPIGAGICASYQTQTN	0.9249	16	ABCPRED	outside	Non-toxin	No
697	714	MSLGAENSVAYSNNSIAI	0.9071	18	ABCPRED	outside	Non-toxin	No
705	714	VAYSNNSIAI	1.0545	10	IEDB	outside	Non-toxin	No
712	723	IAIPTNFTISVT	1.0108	12	ABCPRED	outside	Non-toxin	No
715	726	PTNFTISVTTEI	1.0904	12	BCPREDS	outside	Non-toxin	No
716	729	TNFTISVTTEILPV	1.1495	14	FBCPred	outside	Non-toxin	No
720	729	ISVTTEILPV	1.1242	10	ABCPRED	outside	Non-toxin	No
715	730	PTNFTISVTTEILPVS	1.1529	16	FBCPred	outside	Non-toxin	No
719	730	TISVTTEILPVS	1.036	12	ABCPRED,FBCPred	outside	Non-toxin	No
721	730	SVTTEILPVS	0.9195	10	ABCPRED	outside	Non-toxin	No
723	730	TTEILPVS	1.2071	8	IEDB	outside	Non-toxin	No
714	731	IPTNFTISVTTEILPVSM	0.9956	18	ABCPRED	outside	Non-toxin	No
722	731	VTTEILPVSM	1.0712	10	ABCPRED	outside	Non-toxin	No
715	732	PTNFTISVTTEILPVSMT	1.3036	18	ABCPRED	outside	Non-toxin	No
717	732	NFTISVTTEILPVSMT	1.3573	16	ABCPRED	outside	Non-toxin	No
721	732	SVTTEILPVSMT	1.2175	12	ABCPRED	outside	Non-toxin	No
723	732	TTEILPVSMT	1.484	10	ABCPRED	outside	Non-toxin	No
714	733	IPTNFTISVTTEILPVSMTK	1.0688	20	ABCPRED	outside	Non-toxin	No
720	733	ISVTTEILPVSMTK	1.286	14	ABCPRED	outside	Non-toxin	No
715	734	PTNFTISVTTEILPVSMTKT	1.2666	20	ABCPRED	outside	Non-toxin	No
717	734	NFTISVTTEILPVSMTKT	1.3127	18	ABCPRED	outside	Non-toxin	No
719	734	TISVTTEILPVSMTKT	1.2262	16	ABCPRED	outside	Non-toxin	No
721	734	SVTTEILPVSMTKT	1.1868	14	ABCPRED	outside	Non-toxin	No
724	735	TEILPVSMTKTS	1.4845	12	ABCPRED	outside	Non-toxin	No
719	736	TISVTTEILPVSMTKTSV	1.1598	18	ABCPRED	outside	Non-toxin	No
723	736	TTEILPVSMTKTSV	1.2571	14	AAP	outside	Non-toxin	No
727	736	LPVSMTKTSV	1.2574	10	ABCPRED	outside	Non-toxin	No
720	737	ISVTTEILPVSMTKTSVD	1.1887	18	ABCPRED	outside	Non-toxin	No
722	737	VTTEILPVSMTKTSVD	1.1143	16	ABCPRED,AAP	outside	Non-toxin	No
719	738	TISVTTEILPVSMTKTSVDC	1.3041	20	ABCPRED,AAP	outside	Non-toxin	No
721	738	SVTTEILPVSMTKTSVDC	1.2841	18	ABCPRED	outside	Non-toxin	No
725	738	EILPVSMTKTSVDC	1.617	14	ABCPRED	outside	Non-toxin	No
729	738	VSMTKTSVDC	1.6231	10	ABCPRED	outside	Non-toxin	No
731	738	MTKTSVDC	1.5932	8	IEDB	outside	Non-toxin	No
720	739	ISVTTEILPVSMTKTSVDCT	1.4126	20	ABCPRED,FBCPred	outside	Non-toxin	No
722	739	VTTEILPVSMTKTSVDCT	1.3791	18	3CPRED,AAP,FBCPı	outside	Non-toxin	No
724	739	TEILPVSMTKTSVDCT	1.5042	16	ABCPRED	outside	Non-toxin	No
726	739	ILPVSMTKTSVDCT	1.606	14	ABCPRED	outside	Non-toxin	No
730	739	SMTKTSVDCT	1.4047	10	ABCPRED	outside	Non-toxin	No
723	740	TTEILPVSMTKTSVDCTM	1.3906	18	ABCPRED	outside	Non-toxin	No
725	740	EILPVSMTKTSVDCTM	1.5372	16	ABCPRED	outside	Non-toxin	No
727	740	LPVSMTKTSVDCTM	1.4435	14	BCPREDS	outside	Non-toxin	No
731	740	MTKTSVDCTM	1.3958	10	ABCPRED	outside	Non-toxin	No
720	741	ISVTTEILPVSMTKTSVDCTMY	1.2581	22	FBCPred	outside	Non-toxin	No
722	741	VTTEILPVSMTKTSVDCTMY	1.2085	20	ABCPRED	outside	Non-toxin	No
724	741	TEILPVSMTKTSVDCTMY	1.2922	18	ABCPRED	outside	Non-toxin	No
728	741	PVSMTKTSVDCTMY	1.3667	14	ABCPRED	outside	Non-toxin	No
730	741	SMTKTSVDCTMY	1.0622	12	ABCPRED	outside	Non-toxin	No
732	741	TKTSVDCTMY	1.2485	10	ABCPRED	outside	Non-toxin	No
723	742	TTEILPVSMTKTSVDCTMYI	1.1726	20	ABCPRED,BCPREDS	outside	Non-toxin	No
725	742	EILPVSMTKTSVDCTMYI	1.2689	18	ABCPRED	outside	Non-toxin	No
727	742	LPVSMTKTSVDCTMYI	1.1469	16	ABCPRED	outside	Non-toxin	No
733	742	KTSVDCTMYI	0.9345	10	ABCPRED	outside	Non-toxin	No
751	760	NLLLQYGSFC	1.1023	10	ABCPRED	outside	Non-toxin	No
754	763	LQYGSFCTQL	1.4443	10	ABCPRED	outside	Non-toxin	No
753	764	LLQYGSFCTQLN	0.9501	12	AAP	outside	Non-toxin	No

755	764	QYGSFCTQLN	1.3057	10	ABCPRED	outside	Non-toxin	No
754	765	LQYGSFCTQLNR	1.383	12	BCPREDS	outside	Non-toxin	No
756	765	YGSFCTQLNR	0.9565	10	ABCPRED	outside	Non-toxin	No
753	766	LLQYGSFCTQLNRA	0.9077	14	ABCPRED,AAP	outside	Non-toxin	No
755	766	QYGSFCTQLNRA	1.1892	12	ABCPRED	outside	Non-toxin	No
754	767	LQYGSFCTQLNRAL	1.0734	14	ABCPRED	outside	Non-toxin	No
754	771	LQYGSFCTQLNRALTGIA	0.9721	18	ABCPRED	outside	Non-toxin	No
766	777	ALTGIAVEQDKN	0.9459	12	ABCPRED	outside	Non-toxin	No
768	777	TGIAVEQDKN	1.0371	10	ABCPRED	outside	Non-toxin	No
841	852	LGDIAARDLICA	1.1914	12	ABCPRED	outside	Non-toxin	No
842	853	GDIAARDLICAQ	1.1569	12	ABCPRED	outside	Non-toxin	No
841	854	LGDIAARDLICAQK	1.1817	14	ABCPRED	outside	Non-toxin	No
841	856	LGDIAARDLICAQKFN	0.9412	16	ABCPRED	outside	Non-toxin	No
890	905	AGAALQIPFAMQMAYR	0.9036	16	ABCPRED	outside	Non-toxin	No
892	905	AALQIPFAMQMAYR	1.0216	14	ABCPRED	outside	Non-toxin	No
895	906	QIPFAMQMAYRF	1.1307	12	ABCPRED	outside	Non-toxin	No
899	906	AMQMAYRF	0.9776	8	COVIDep	outside	Non-toxin	No
894	907	LQIPFAMQMAYRFN	1.0747	14	ABCPRED	outside	Non-toxin	No
896	909	IPFAMQMAYRFNGI	1.0623	14	ABCPRED	outside	Non-toxin	No
894	911	LQIPFAMQMAYRFNGIGV	1.1951	18	ABCPRED	outside	Non-toxin	No
900	911	MQMAYRFNGIGV	1.3524	12	ABCPRED	outside	Non-toxin	No
893	912	ALQIPFAMQMAYRFNGIGVT	1.2262	20	ABCPRED	outside	Non-toxin	No
897	912	PFAMQMAYRFNGIGVT	1.3871	16	ABCPRED	outside	Non-toxin	No
901	912	QMAYRFNGIGVT	1.4219	12	ABCPRED	outside	Non-toxin	No
894	913	LQIPFAMQMAYRFNGIGVTQ	1.2425	20	ABCPRED	outside	Non-toxin	No
897	913	PFAMQMAYRFNGIGVTQ	1.3306	17	COVIDep	outside	Non-toxin	No
898	913	FAMQMAYRFNGIGVTQ	1.3096	16	ABCPRED	outside	Non-toxin	No
902	913	MAYRFNGIGVTQ	1.3822	12	ABCPRED	outside	Non-toxin	No
897	914	PFAMQMAYRFNGIGVTQN	1.2715	18	ABCPRED	outside	Non-toxin	No
903	914	AYRFNGIGVTQN	1.2149	12	ABCPRED	outside	Non-toxin	No
896	915	IPFAMQMAYRFNGIGVTQNV	1.2009	20	ABCPRED	outside	Non-toxin	No
900	915	MQMAYRFNGIGVTQNV	1.1008	16	ABCPRED	outside	Non-toxin	No
904	915	YRFNGIGVTQNV	1.1043	12	ABCPRED,FBCPred	outside	Non-toxin	No
898	917	FAMQMAYRFNGIGVTQNVLY	0.9281	20	ABCPRED	outside	Non-toxin	No
1031	1039	ECVLGQSKR	0.9377	9	BCEPRED	outside	Non-toxin	No
1027	1042	TKMSECVLGQSKRVDF	1.0151	16	ABCPRED	outside	Non-toxin	No
1029	1042	MSECVLGQSKRVDF	1.0345	14	ABCPRED	outside	Non-toxin	No
1035	1042	GQSKRVDF	1.9298	8	BCEPRED	outside	Non-toxin	No
1035	1043	GQSKRVDFC	1.779	9	IEDB	outside	Non-toxin	No
1031	1044	ECVLGQSKRVDFCG	0.9433	14	ABCPRED	outside	Non-toxin	No
1030	1045	SECVLGQSKRVDFCGK	1.0135	16	ABCPRED	outside	Non-toxin	No
1033	1046	VLGQSKRVDFCGKG	1.3582	14	ABCPRED,IEDB	outside	Non-toxin	No
1032	1047	CVLGQSKRVDFCGKGY	1.0648	16	ABCPRED	outside	Non-toxin	No
1031	1048	ECVLGQSKRVDFCGKGYH	1.009	18	ABCPRED	outside	Non-toxin	No
1035	1048	GQSKRVDFCGKGYH	1.1237	14	ABCPRED	outside	Non-toxin	No
1032	1049	CVLGQSKRVDFCGKGYHL	1.1216	18	ABCPRED	outside	Non-toxin	No
1034	1049	LGQSKRVDFCGKGYHL	1.2157	16	ABCPRED	outside	Non-toxin	No
1038	1049	KRVDFCGKGYHL	1.3047	12	BCPREDS	outside	Non-toxin	No
1033	1050	VLGQSKRVDFCGKGYHLM	1.0561	18	ABCPRED	outside	Non-toxin	No
1035	1052	GQSKRVDFCGKGYHLMSF	0.9773	18	ABCPRED	outside	Non-toxin	No
1037	1052	SKRVDFCGKGYHLMSF	1.0195	16	ABCPRED	outside	Non-toxin	No
1057	1069	PHGVVFLHVTYVP	0.9305	13	BCEPRED	outside	Non-toxin	No
1059	1072	GVVFLHVTYVPAQE	1.0085	14	ABCPRED	outside	Non-toxin	No
1057	1074	PHGVVFLHVTYVPAQEKN	0.9072	18	ABCPRED	outside	Non-toxin	No
1060	1075	VVFLHVTYVPAQEKNF	1.1471	16	ABCPRED	outside	Non-toxin	No
1064	1075	HVTYVPAQEKNF	1.1353	12	AP,BCPREDS,FBCPr	outside	Non-toxin	No
1059	1076	GVVFLHVTYVPAQEKNFT	1.1197	18	ABCPRED	outside	Non-toxin	No
1063	1076	LHVTYVPAQEKNFT	1.2382	14	ABCPRED	outside	Non-toxin	No
1068	1076	VPAQEKNFT	1.0107	9	IEDB	outside	Non-toxin	No
1060	1077	VVFLHVTYVPAQEKNFTT	0.9224	18	ABCPRED	outside	Non-toxin	No
1062	1077	FLHVTYVPAQEKNFTT	0.9556	16	ABCPRED	outside	Non-toxin	No
1063	1078	LHVTYVPAQEKNFTTA	1.0276	16	ABCPRED	outside	Non-toxin	No
1060	1079	VVFLHVTYVPAQEKNFTTAP	0.9571	20	ABCPRED	outside	Non-toxin	No
1063	1080	LHVTYVPAQEKNFTTAPA	0.9567	18	BCPREDS,FBCPred	outside	Non-toxin	No
1062	1081	FLHVTYVPAQEKNFTTAPAI	0.9351	20	AAP	outside	Non-toxin	No
1063	1082	LHVTYVPAQEKNFTTAPAIC	0.9251	20	BCPREDS	outside	Non-toxin	No
1157	1167	KNHTSPDVDLG	1.4039	11	IEDB	outside	Non-toxin	No
1156	1168	FKNHTSPDVDLGD	1.0616	13	IEDB	outside	Non-toxin	No
1155	1170	YFKNHTSPDVDLGDIS	1.1715	16	ABCPRED	outside	Non-toxin	No
1158	1171	NHTSPDVDLGDISG	1.0939	14	FBCPred	outside	Non-toxin	No
1159	1172	HTSPDVDLGDISGI	1.0779	14	AAP	outside	Non-toxin	No
1156	1173	FKNHTSPDVDLGDISGIN	0.9468	18	ABCPRED	outside	Non-toxin	No
1157	1173	KNHTSPDVDLGDISGIN	1.1116	17	COVIDep	outside	Non-toxin	No
1158	1173	NHTSPDVDLGDISGIN	1.1399	16	ABCPRED	outside	Non-toxin	No
1162	1173	PDVDLGDISGIN	1.2343	12	FBCPred	outside	Non-toxin	No
1157	1174	KNHTSPDVDLGDISGINA	1.0631	18	ABCPRED	outside	Non-toxin	No
1159	1174	HTSPDVDLGDISGINA	1.0848	16	ABCPRED	outside	Non-toxin	No
1156	1175	FKNHTSPDVDLGDISGINAS	0.9142	20	ABCPRED	outside	Non-toxin	No
1160	1175	TSPDVDLGDISGINAS	1.0756	16	AAP	outside	Non-toxin	No
1159	1176	HTSPDVDLGDISGINASV	0.9036	18	ABCPRED	outside	Non-toxin	No
1160	1177	TSPDVDLGDISGINASVV	0.9371	18	ABCPRED	outside	Non-toxin	No
1159	1178	HTSPDVDLGDISGINASVVN	0.9177	20	AAP	outside	Non-toxin	No
1163	1178	DVDLGDISGINASVVN	1.1317	16	ABCPRED	outside	Non-toxin	No
1162	1179	PDVDLGDISGINASVVNI	1.0023	18	ABCPRED	outside	Non-toxin	No
1164	1179	VDLGDISGINASVVNI	1.0784	16	ABCPRED	outside	Non-toxin	No
1159	1180	HTSPDVDLGDISGINASVVNIQ	1.0856	22	FBCPred	outside	Non-toxin	No
1161	1180	SPDVDLGDISGINASVVNIQ	1.0657	20	ABCPRED	outside	Non-toxin	No
1163	1180	DVDLGDISGINASVVNIQ	1.3153	18	FBCPred	outside	Non-toxin	No
1165	1180	DLGDISGINASVVNIQ	1.0543	16	FBCPred	outside	Non-toxin	No

1162	1181	PDVDLGDISGINASVVNIQK	0.9428	20	FBCPred	outside	Non-toxin	No
1165	1182	DLGDISGINASVVNIQKE	0.9087	18	ABCPRED	outside	Non-toxin	No

Supplementary Table 4. Discontinuous B-cell epitopes predicted by DiscoTope 2 with the discotope score threshold of -3.7 (default setting).

chain_id	residue_id	residue_name	contact_number	propensity_score	discotope_score	locations on the 3D structure of SARS-CoV-2 (PDB ID: 6VSB)
A	281	GLU	0	-3.366	-2.979	stem
A	282	ASN	7	-2.664	-3.162	stem
A	415	THR	0	-3.819	-3.38	head
A	420	ASP	4	-3.618	-3.662	head
A	449	TYR	4	-0.567	-0.962	head
A	450	ASN	11	-1.78	-2.841	head
A	454	ARG	14	-1.224	-2.694	head
A	491	PRO	7	-0.72	-1.442	head
A	492	LEU	15	-0.95	-2.565	head
A	493	GLN	9	-0.572	-1.541	head
A	494	SER	7	-0.846	-1.553	head
A	496	GLY	3	0.041	-0.309	head
A	498	GLN	4	0.68	0.142	head
A	499	PRO	5	0.178	-0.417	head
A	500	THR	0	1.907	1.688	head
A	503	VAL	5	-1.856	-2.218	head
A	505	TYR	8	-1.528	-2.272	head
A	556	ASN	2	-3.79	-3.584	stem
A	558	LYS	2	-1.479	-1.539	head
A	560	LEU	2	-1.137	-1.236	stem
A	561	PRO	0	-0.961	-0.851	head
A	562	PHE	0	-2.061	-1.824	head
A	703	ASN	4	-2.02	-2.248	stem
A	704	SER	3	-1.469	-1.645	stem
A	705	VAL	10	-2.821	-3.646	stem
A	793	PRO	1	-2.278	-2.131	stem
A	794	ILE	1	-2.5	-2.327	stem
A	809	PRO	4	-2.691	-2.841	stem
A	810	SER	9	-0.669	-1.627	stem
A	914	ASN	7	-1.117	-1.794	stem
A	917	TYR	9	-2.702	-3.426	stem
A	918	GLU	13	-2.285	-3.517	stem
A	1071	GLN	9	-2.775	-3.491	stem
A	1099	GLY	1	-3.789	-3.468	stem
A	1100	THR	0	-3.877	-3.431	stem
A	1101	HIS	8	-2.903	-3.489	stem
A	1111	GLU	19	-1.693	-3.684	stem
A	1118	ASP	4	-3.016	-3.129	stem
A	1140	PRO	7	-0.961	-1.656	root
A	1141	LEU	5	-0.257	-0.802	root
A	1142	GLN	7	0.318	-0.523	root
A	1143	PRO	6	1.067	0.255	root
A	1144	GLU	6	0.716	-0.056	root
A	1145	LEU	5	0.162	-0.431	root
A	1146	ASP	5	0.731	0.072	root
B	187	LYS	9	-2.398	-3.158	stem
B	209	PRO	1	-3.243	-2.985	head
B	281	GLU	0	-4.065	-3.597	stem
B	282	ASN	6	-2.337	-2.758	stem
B	415	THR	0	-2.448	-2.167	head
B	417	LYS	15	-2.055	-3.544	head
B	420	ASP	5	-3.2	-3.407	head
B	449	TYR	3	-1.991	-2.107	head
B	460	ASN	15	-0.073	-1.79	head
B	462	LYS	2	-3.079	-2.955	head
B	469	SER	1	-2.505	-2.332	head
B	471	GLU	1	-2.616	-2.43	head
B	472	ILE	3	-3.202	-3.179	head
B	473	TYR	11	-2.078	-3.104	head
B	487	ASN	0	-1.605	-1.42	head
B	488	CYS	3	-2.718	-2.75	head
B	489	TYR	6	-3.009	-3.353	head
B	493	GLN	12	-2.368	-3.476	head
B	494	SER	8	-2.189	-2.858	head
B	496	GLY	1	-0.693	-0.728	head
B	497	PHE	18	-1.153	-3.091	head
B	498	GLN	7	1.188	0.246	head
B	499	PRO	5	0.294	-0.315	head
B	500	THR	1	2.231	1.86	head
B	503	VAL	6	-1.621	-2.124	head

B	504	GLY	0	-2.702	-2.392	head
B	505	TYR	9	-1.67	-2.513	head
B	556	ASN	2	-3.692	-3.497	stem
B	558	LYS	2	-2.13	-2.115	stem
B	560	LEU	1	-3.858	-3.529	stem
B	561	PRO	0	-3.986	-3.528	stem
B	703	ASN	4	-2.12	-2.336	stem
B	704	SER	3	-1.967	-2.086	stem
B	705	VAL	10	-2.857	-3.678	stem
B	716	THR	10	-2.78	-3.61	stem
B	793	PRO	0	-2.542	-2.25	stem
B	794	ILE	3	-2.633	-2.675	stem
B	809	PRO	4	-3.089	-3.193	stem
B	810	SER	9	-1.014	-1.932	stem
B	914	ASN	6	-1.369	-1.901	stem
B	917	TYR	9	-2.268	-3.042	stem
B	918	GLU	11	-2.251	-3.257	stem
B	1071	GLN	7	-3.08	-3.53	stem
B	1111	GLU	19	-1.343	-3.373	stem
B	1114	ILE	8	-2.852	-3.444	stem
B	1118	ASP	5	-2.997	-3.228	stem
B	1140	PRO	8	-0.677	-1.519	root
B	1141	LEU	5	-0.017	-0.59	root
B	1142	GLN	7	0.372	-0.476	root
B	1143	PRO	6	0.629	-0.134	root
B	1144	GLU	4	0.704	0.163	root
B	1145	LEU	5	0.171	-0.424	root
B	1146	ASP	4	0.724	0.181	root
C	281	GLU	0	-3.898	-3.45	stem
C	282	ASN	3	-2.566	-2.616	stem
C	415	THR	0	-3.378	-2.989	head
C	449	TYR	3	-1.844	-1.977	head
C	460	ASN	11	-0.126	-1.376	head
C	462	LYS	1	-2.996	-2.767	head
C	469	SER	5	-2.411	-2.708	head
C	470	THR	12	-2.535	-3.624	head
C	471	GLU	2	-1.882	-1.896	head
C	487	ASN	0	-1.487	-1.316	head
C	488	CYS	0	-2.512	-2.223	head
C	489	TYR	7	-2.629	-3.132	head
C	493	GLN	9	-2.288	-3.06	head
C	494	SER	7	-2.316	-2.854	head
C	496	GLY	1	-1.754	-1.668	head
C	498	GLN	3	-0.804	-1.057	head
C	503	VAL	10	-2.855	-3.677	head
C	504	GLY	0	-2.423	-2.145	head
C	505	TYR	9	-2.339	-3.105	head
C	556	ASN	0	-3.139	-2.778	stem
C	558	LYS	1	-1.819	-1.725	stem
C	560	LEU	1	-2.713	-2.516	stem
C	561	PRO	0	-3.804	-3.367	stem
C	703	ASN	3	-2.135	-2.235	stem
C	704	SER	3	-1.544	-1.711	stem
C	793	PRO	0	-2.671	-2.364	stem
C	794	ILE	4	-2.452	-2.63	stem
C	809	PRO	4	-3.118	-3.22	stem
C	810	SER	9	-1.295	-2.181	stem
C	914	ASN	9	-0.693	-1.648	stem
C	917	TYR	9	-2.612	-3.347	stem
C	918	GLU	12	-1.782	-2.957	stem
C	1071	GLN	7	-2.99	-3.451	stem
C	1074	ASN	7	-2.785	-3.27	stem
C	1100	THR	0	-3.348	-2.963	stem
C	1111	GLU	18	-1.566	-3.456	stem
C	1114	ILE	8	-2.926	-3.51	stem
C	1118	ASP	4	-3.023	-3.136	stem
C	1140	PRO	8	-1.41	-2.168	root
C	1141	LEU	3	-0.304	-0.614	root
C	1142	GLN	6	0.278	-0.444	root
C	1143	PRO	6	0.79	0.009	root
C	1144	GLU	4	0.433	-0.077	root
C	1145	LEU	4	0.405	-0.101	root
C	1146	ASP	4	0.234	-0.253	root

Supplementary Table 5. Predicted B-cell epitopes in peak 1, 2, 3 and 4.

start	end	peptide	Antigenicity (VaxiJen)	Peptide Length	methods	transmembrane topology (TMHMM v2.0)	Toxicity (ToxinPred)	Peaks overlapped with RBD and Discontinuous epitope enriched regions	Number of mutation sites	Number of virus strains	Number of mutation sites in key amino acids critical for protein interactions	Number of virus strains having mutations in key amino acids critical for protein interactions
375	384	STFKCYGVSP	1.104	10	ABCPRED	outside	Non-toxin	Peak1	10	13852193	0	0
376	385	TFKCYGVSPT	1.5059	10	ABCPRED,IEDB	outside	Non-toxin	Peak1	10	5634212	0	0
371	386	SASFSTFKCYGVSPTK	0.908	16	ABCPRED	outside	Non-toxin	Peak1	16	32917064	0	0
373	386	SFSTFKCYGVSPTK	0.9678	14	ABCPRED	outside	Non-toxin	Peak1	14	22094636	0	0
372	387	ASFSTFKCYGVSPTKL	0.912	16	ABCPRED	outside	Non-toxin	Peak1	16	22096895	0	0
376	387	TFKCYGVSPTKL	1.4293	12	ABCPRED	outside	Non-toxin	Peak1	12	5634815	0	0
371	388	SASFSTFKCYGVSPTKLN	0.9891	18	ABCPRED	outside	Non-toxin	Peak1	18	32918210	0	0
375	388	STFKCYGVSPTKLN	1.2695	14	AAP	outside	Non-toxin	Peak1	14	13863122	0	0
379	388	CYGVSPTKLN	1.5759	10	ABCPRED	outside	Non-toxin	Peak1	10	27529	0	0
381	388	GVSPTKLN	1.9197	8	IEDB	outside	Non-toxin	Peak1	8	27161	0	0
374	389	FSTFKCYGVSPTKLND	0.965	16	ABCPRED	outside	Non-toxin	Peak1	16	13867657	0	0
378	389	KCYGVSPTKLND	1.3195	12	BCPRED,BCPRED	outside	Non-toxin	Peak1	12	31301	0	0
380	389	YGVSPTKLND	1.4531	10	IEDB	outside	Non-toxin	Peak1	10	30038	0	0
369	390	YNSASFSTFKCYGVSPTKLNDL	0.9508	22	AAP	outside	Non-toxin	Peak1	22	32925685	0	0
375	390	STFKCYGVSPTKLNDL	1.2492	16	ABCPRED	outside	Non-toxin	Peak1	16	13866301	0	0
377	390	FKCYGVSPTKLNDL	1.4748	14	ABCPRED	outside	Non-toxin	Peak1	14	33679	0	0
379	390	CYGVSPTKLNDL	1.4808	12	ABCPRED	outside	Non-toxin	Peak1	12	30708	0	0
381	390	GVSPTKLNDL	1.6525	10	ABCPRED	outside	Non-toxin	Peak1	10	30340	0	0
383	390	SPTKLNDL	1.0358	8	BCEPRED	outside	Non-toxin	Peak1	8	25400	0	0
372	391	ASFSTFKCYGVSPTKLNDLC	1.227	20	ABCPRED	outside	Non-toxin	Peak1	20	22101847	0	0
374	391	FSTFKCYGVSPTKLNDLC	1.2396	18	ABCPRED	outside	Non-toxin	Peak1	18	13869013	0	0
380	391	YGVSPTKLNDLC	1.8347	12	ABCPRED,AAP	outside	Non-toxin	Peak1	12	31394	0	0
382	391	VSPTKLNDLC	1.9573	10	ABCPRED	outside	Non-toxin	Peak1	10	31121	0	0
375	392	STFKCYGVSPTKLNDLCF	1.7606	18	ABCPRED	outside	Non-toxin	Peak1	18	13872510	0	0
377	392	FKCYGVSPTKLNDLCF	2.0332	16	ABCPRED	outside	Non-toxin	Peak1	16	39888	0	0
383	392	SPTKLNDLCF	2.2412	10	ABCPRED	outside	Non-toxin	Peak1	10	31609	0	0
369	393	NSASFSTFKCYGVSPTKLNDLCF	1.4031	25	IEDB	outside	Non-toxin	Peak1	25	32932156	0	0
378	393	KCYGVSPTKLNDLCFT	2.0395	16	ABCPRED	outside	Non-toxin	Peak1	16	38210	0	0
382	393	VSPTKLNDLCFT	2.4268	12	ABCPRED	outside	Non-toxin	Peak1	12	36674	0	0
384	393	PTKLNDLCFT	2.5246	10	ABCPRED	outside	Non-toxin	Peak1	10	31712	0	0
377	394	FKCYGVSPTKLNDLCFTN	1.9296	18	ABCPRED	outside	Non-toxin	Peak1	18	44177	0	0
379	394	CYGVSPTKLNDLCFTN	2.0134	16	ABCPRED	outside	Non-toxin	Peak1	16	41206	0	0
378	395	KCYGVSPTKLNDLCFTNV	1.8687	18	ABCPRED	outside	Non-toxin	Peak1	18	42615	0	0
380	395	YGVSPTKLNDLCFTNV	2.0255	16	ABCPRED	outside	Non-toxin	Peak1	16	41352	0	0
386	395	KLNDLCFTNV	2.6927	10	ABCPRED	outside	Non-toxin	Peak1	10	15513	0	0
379	396	CYGVSPTKLNDLCFTNVY	1.7815	18	ABCPRED	outside	Non-toxin	Peak1	18	41804	0	0
381	396	GVSPTKLNDLCFTNVY	1.9127	16	ABCPRED	outside	Non-toxin	Peak1	16	41436	0	0
383	396	SPTKLNDLCFTNVY	1.6982	14	ABCPRED	outside	Non-toxin	Peak1	14	36496	0	0
385	396	TKLNDLCFTNVY	2.0035	12	ABCPRED	outside	Non-toxin	Peak1	12	25204	0	0
387	396	LNDLCFTNVY	1.6134	10	ABCPRED	outside	Non-toxin	Peak1	10	15421	0	0
389	396	DLCFTNVY	1.8569	8	BCEPRED	outside	Non-toxin	Peak1	8	14275	0	0
378	397	KCYGVSPTKLNDLCFTNVYA	1.5664	20	ABCPRED,AAP	outside	Non-toxin	Peak1	20	43298	0	0
379	398	CYGVSPTKLNDLCFTNVYAD	1.5222	20	ABCPRED	outside	Non-toxin	Peak1	20	42815	0	0
383	398	SPTKLNDLCFTNVYAD	1.3666	16	ABCPRED	outside	Non-toxin	Peak1	16	37507	0	0
380	399	YGVSPTKLNDLCFTNVYADS	1.361	20	ABCPRED	outside	Non-toxin	Peak1	20	42653	0	0
384	399	PTKLNDLCFTNVYADS	1.2303	16	ABCPRED	outside	Non-toxin	Peak1	16	37418	0	0
381	400	GVSPTKLNDLCFTNVYADSF	1.2617	20	ABCPRED	outside	Non-toxin	Peak2	20	43732	0	0
380	401	YGVSPTKLNDLCFTNVYADSFV	1.1404	22	BCPREDS	outside	Non-toxin	Peak2	22	45395	0	0
383	402	SPTKLNDLCFTNVYADSFVI	0.9482	20	ABCPRED	outside	Non-toxin	Peak2	20	41112	0	0
385	402	TKLNDLCFTNVYADSFVI	1.0271	18	ABCPRED	outside	Non-toxin	Peak2	18	29820	0	0
384	403	PTKLNDLCFTNVYADSFVIR	0.9772	20	ABCPRED	outside	Non-toxin	Peak2	20	42874	0	0
386	403	KLNDLCFTNVYADSFVIR	1.0694	18	ABCPRED	outside	Non-toxin	Peak2	18	22270	0	0
385	404	TKLNDLCFTNVYADSFVIRG	1.049	20	ABCPRED	outside	Non-toxin	Peak2	20	32071	0	0
386	405	KLNDLCFTNVYADSFVIRGD	0.932	20	ABCPRED	outside	Non-toxin	Peak2	20	5619667	0	0
406	415	EVRQIAPGQT	1.0655	10	ABCPRED	outside	Non-toxin	Peak2	10	4109122	0	0
405	416	DEVRQIAPGQTG	1.0397	12	ABCPRED	outside	Non-toxin	Peak2	12	9706300	0	0
407	416	VRQIAPGQTG	1.3856	10	ABCPRED	outside	Non-toxin	Peak2	10	4108153	0	0
404	417	GDEVRQIAPGQTGK	1.1397	14	CPRED,AAP,FBCP	outside	Non-toxin	Peak2	14	15267070	1	5560440
409	418	QIAPGQTGKI	1.4416	10	ABCPRED	outside	Non-toxin	Peak2	10	5974336	1	5560440
406	419	EVRQIAPGQTGKIA	1.3182	14	ABCPRED	outside	Non-toxin	Peak2	14	9672837	1	5560440
408	419	RQIAPGQTGKIA	1.4558	12	BCPREDS,FBCPred	outside	Non-toxin	Peak2	12	9671003	1	5560440
405	420	DEVRQIAPGQTGKIAD	1.0388	16	ABCPRED	outside	Non-toxin	Peak2	16	15271342	1	5560440
407	420	VRQIAPGQTGKIAD	1.2606	14	IEDB	outside	Non-toxin	Peak2	14	9673195	1	5560440
411	420	APGQTGKIAD	1.2753	10	ABCPRED	outside	Non-toxin	Peak2	10	5594288	1	5560440
406	421	EVRQIAPGQTGKIADY	1.3837	16	ABCPRED	outside	Non-toxin	Peak2	16	9674469	1	5560440
408	421	RQIAPGQTGKIADY	1.504	14	ABCPRED	outside	Non-toxin	Peak2	14	9672635	1	5560440
410	421	IAPGQTGKIADY	1.6626	12	ABCPRED	outside	Non-toxin	Peak2	12	5978685	1	5560440
407	422	VRQIAPGQTGKIADYN	1.3372	16	ABCPRED	outside	Non-toxin	Peak2	16	9673636	1	5560440
409	422	QIAPGQTGKIADYN	1.5448	14	ABCPRED	outside	Non-toxin	Peak2	14	5979312	1	5560440
404	423	GDEVRQIAPGQTGKIADYNY	1.219	20	ABCPRED	outside	Non-toxin	Peak2	20	15272116	1	5560440
406	423	EVRQIAPGQTGKIADYNY	1.4691	18	BCPREDS	outside	Non-toxin	Peak2	18	9674719	1	5560440
408	423	RQIAPGQTGKIADYNY	1.5799	16	ABCPRED	outside	Non-toxin	Peak2	16	9672885	1	5560440
410	423	IAPGQTGKIADYNY	1.724	14	BCPRED,BCPRED	outside	Non-toxin	Peak2	14	5978935	1	5560440
412	423	PGQTGKIADYNY	1.642	12	ABCPRED	outside	Non-toxin	Peak2	12	5585495	1	5560440
414	423	QTGKIADYNY	1.5116	10	ABCPRED	outside	Non-toxin	Peak2	10	5583961	1	5560440
413	424	GQTGKIADYNYK	1.7308	12	ABCPRED	outside	Non-toxin	Peak2	12	5587081	1	5560440
410	425	IAPGQTGKIADYNYKL	1.6707	16	ABCPRED	outside	Non-toxin	Peak2	16	5981766	1	5560440
411	425	APGQTGKIADYNYKL	1.4441	15	IEDB	outside	Non-toxin	Peak2	15	5597563	1	5560440
414	425	QTGKIADYNYKL	1.4851	12	ABCPRED	outside	Non-toxin	Peak2	12	5586792	1	5560440
416	425	GKIADYNYKL	1.6079	10	ABCPRED	outside	Non-toxin	Peak2	10	5568428	1	5560440
404	426	GDEVRQIAPGQTGKIADYNYKLP	1.1017	23	IEDB	outside	Non-toxin	Peak2	23	15275690	1	5560440
407	426	VRQIAPGQTGKIADYNYKLP	1.2386	20	ABCPRED	outside	Non-toxin	Peak2	20	9677213	1	5560440
411	426	APGQTGKIADYNYKLP	1.2303	16	ABCPRED	outside	Non-toxin	Peak2	16	5598306	1	5560440
413	426	GQTGKIADYNYKLP	1.2756	14	ABCPRED	outside	Non-toxin	Peak2	14	5588585	1	5560440
415	426	TGKIADYNYKLP	1.1956	12	ABCPRED	outside	Non-toxin	Peak2	12	5570596	1	5560440
417	426	KIADYNYKLP	1.2565	10	ABCPRED	outside	Non-toxin	Peak2	10	5569060	1	5560440
408	427	RQIAPGQTGKIADYNYKLPD	1.4102	20	BCPRED,BCPRED	outside	Non-toxin	Peak2	20	9688554	1	5560440
412	427	PGQTGKIADYNYKLPD	1.4039	16	ABCPRED	outside	Non-toxin	Peak2	16	5601164	1	5560440
414	427	QTGKIADYNYKLPD	1.2934	14	ABCPRED	outside	Non-toxin	Peak2	14	5599630	1	5560440
416	427	GKIADYNYKLPD	1.3553	12	ABCPRED	outside	Non-toxin	Peak2	12	5581266	1	5560440
413	428	GQTGKIADYNYKLPDD	1.0071	16	ABCPRED	outside	Non-toxin	Peak2	16	5603337	1	5560440
414	429	QTGKIADYNYKLPDDF	0.986	16	ABCPRED	outside	Non-toxin	Peak2	16	5602616	1	5560440
416	429	GKIADYNYKLPDDF	0.9776	14	ABCPRED	outside	Non-toxin	Peak2	14	5584252	1	5560440
411	430	APGQTGKIADYNYKLPDDFT	1.0425	20	ABCPRED,IEDB	outside	Non-toxin	Peak2	20	5616221	1	5560440
413	430	GQTGKIADYNYKLPDDFT	1.0564	18	ABCPRED	outside	Non-toxin	Peak2	18	5606500	1	5560440
415	430	TGKIADYNYKLPDDFT	0.9642	16	ABCPRED	outside	Non-toxin	Peak2	16	5588511	1	5560440
417	430	KIADYNYKLPDDFT	0.9567	14	BCEPRED	outside	Non-toxin	Peak2	14	5586975	1	5560440
412	431	PGQTGKIADYNYKLPDDFTG	1.0123	20	ABCPRED	outside	Non-toxin	Peak2	20	5607036	1	5560440
433	442	VIAWNSNNLD	0.9177	10	ABCPRED	outside	Non-toxin	Peak2	10	5348416	0	0
435	444	AWNSNNLDSK	1.0198	10	BCEPRED	outside	Non-toxin	Peak2	10	5405247	0	0
439	446	NNLDSKVG	1.2952	8	BCEPRED	outside	Non-toxin	Peak2	8	8033647	1	2607609
434	449	IAWNSNNLDSKVGGNY	0.906	16	ABCPRED	outside	Non-toxin	Peak2	16	8049651	2	2615877
441	450	LDSKVGGNYN	0.91	10	ABCPRED	outside	Non-toxin	Peak2	10	2724108	2	2615877
434	451	IAWNSNNLDSKVGGNYNY	1.0683	18	ABCPRED	outside	Non-toxin	Peak2	18	8068630	2	2615877
456	463	FRKSNLKP	1.1111	8	BCEPRED	outside	Non-toxin	Peak3	8	73052	1	2490
482	493	GVEGFNCYFPLQ	0.9537	12	ABCPRED	outside	Non-toxin	Peak3	12	16813308	4	8218348
488	495	CYFPLQSY	0.9394	8	IEDB	outside	Non-toxin	Peak3	8	5909922	2	5829624
485	500	GFNCYFPLQSYGFQPT	0.981	16	ABCPRED	outside	Non-toxin	Peak3	16	19150957	7	19061793
482	501	GVEGFNCYFPLQSYGFQPTN	0.9121	20	ABCPRED	outside	Non-toxin	Peak3	20	38658947	8	30025060
486	501	FNCYFPLQSYGFQPTN	1.0229	16	ABCPRED	outside	Non-toxin	Peak3	16	30112373	8	30025060
490	501	FPLQSYGFQPTN	0.9942	12	ABCPRED	outside	Non-toxin	Peak3	12	27722579	5	27635850
492	501	LQSYGFQPTN	1.043	10	ABCPRED	outside	Non-toxin	Peak3	10	27676009	5	27635850
493	502	QSYGFQPTNG	0.9097	10	ABCPRED	outside	Non-toxin	Peak3	10	27675320	6	27636393
494	503	SYGFQPTNGV	1.0549	10	ABCPRED	outside	Non-toxin	Peak3	10	21849389	5	21807255

502	511	GVGYQPYRVV	1.1915	10	ABCPRED	outside	Non-toxin	Peak3	10	8231684	2	8221878
503	511	VGYQPYRVV	1.4383	9	BCEPRED	outside	Non-toxin	Peak3	9	8231141	1	8221335
502	513	GVGYQPYRVVVL	1.1207	12	BCPREDS,FBCPred	outside	Non-toxin	Peak3	12	8232931	2	8221878
502	515	GVGYQPYRVVVLSF	1.2391	14	ABCPRED	outside	Non-toxin	Peak3	14	8235843	2	8221878
504	515	GYQPYRVVVLSF	1.1235	12	ABCPRED	outside	Non-toxin	Peak3	12	8232093	1	8221335
501	516	NGVGYQPYRVVVLSFE	0.9736	16	\BCPRED,BCPRED	outside	Non-toxin	Peak3	16	19201321	3	19185145
505	516	YQPYRVVVLSFE	0.9911	12	ABCPRED	outside	Non-toxin	Peak3	12	8232570	1	8221335
506	517	QPYRVVVLSFEL	0.9785	12	ABCPRED	outside	Non-toxin	Peak3	12	13791	0	0
508	517	YRVVVLSFEL	1.1494	10	ABCPRED	outside	Non-toxin	Peak3	10	13052	0	0
505	519	YQPYRVVVLSFELLH	0.9711	15	BCEPRED	outside	Non-toxin	Peak3	15	8251534	1	8221335
506	519	QPYRVVVLSFELLH	0.9675	14	ABCPRED	outside	Non-toxin	Peak3	14	30199	0	0
508	519	YRVVVLSFELLH	1.1085	12	ABCPRED	outside	Non-toxin	Peak3	12	29460	0	0
501	520	NGVGYQPYRVVVLSFELLHA	0.9212	20	ABCPRED	outside	Non-toxin	Peak3	20	19240199	3	19185145
507	520	PYRVVVLSFELLHA	1.0185	14	ABCPRED	outside	Non-toxin	Peak3	14	49869	0	0
503	522	VGYQPYRVVVLSFELLHAPA	0.989	20	ABCPRED	outside	Non-toxin	Peak3	20	8309497	1	8221335
513	524	LSFELLHAPATV	0.9969	12	ABCPRED	outside	Non-toxin	Peak4	12	78993	0	0
528	541	KKSTNLVKNKCVNF	1.0894	14	ABCPRED	outside	Non-toxin	Peak4	14	28417	0	0
526	543	GPKKSTNLVKNKCVNFNF	1.1626	18	ABCPRED	outside	Non-toxin	Peak4	18	66131	0	0
530	543	STNLVKNKCVNFNF	1.289	14	ABCPRED	outside	Non-toxin	Peak4	14	63711	0	0
532	543	NLVKNKCVNFNF	1.3432	12	ABCPRED	outside	Non-toxin	Peak4	12	63643	0	0
529	544	KSTNLVKNKCVNFNFN	1.4412	16	ABCPRED	outside	Non-toxin	Peak4	16	66104	0	0
531	544	TNLVKNKCVNFNFN	1.2489	14	\BCPRED,BCPRED	outside	Non-toxin	Peak4	14	64364	0	0
533	544	LVKNKCVNFNFN	1.6246	12	FBCPred	outside	Non-toxin	Peak4	12	60684	0	0
535	544	KNKCVNFNFN	2.2331	10	ABCPRED	outside	Non-toxin	Peak4	10	58370	0	0
536	545	NKCVNFNFNG	1.3088	10	ABCPRED	outside	Non-toxin	Peak4	10	58304	0	0
527	546	PKKSTNLVKNKCVNFNFNGL	1.0582	20	ABCPRED	outside	Non-toxin	Peak4	20	68269	0	0
529	546	KSTNLVKNKCVNFNFNGL	1.22	18	ABCPRED	outside	Non-toxin	Peak4	18	67604	0	0
533	546	LVKNKCVNFNFNGL	1.3128	14	FBCPred	outside	Non-toxin	Peak4	14	62184	0	0
535	546	KNKCVNFNFNGL	1.7395	12	ABCPRED	outside	Non-toxin	Peak4	12	59870	0	0
538	546	CVNFNFNGL	1.7985	9	BCEPRED	outside	Non-toxin	Peak4	9	55207	0	0
528	547	KKSTNLVKNKCVNFNFNGLT	1.0818	20	ABCPRED	outside	Non-toxin	Peak4	20	2737898	0	0
530	547	STNLVKNKCVNFNFNGLT	0.9604	18	ABCPRED	outside	Non-toxin	Peak4	18	2735698	0	0
534	547	VKNKCVNFNFNGLT	1.5749	14	ABCPRED	outside	Non-toxin	Peak4	14	2731068	0	0
535	548	KNKCVNFNFNGLTG	1.6052	14	ABCPRED	outside	Non-toxin	Peak4	14	2729748	0	0
537	548	KCVNFNFNGLTG	1.6969	12	IEDB	outside	Non-toxin	Peak4	12	2725517	0	0
530	549	STNLVKNKCVNFNFNGLTGT	1.0756	20	ABCPRED	outside	Non-toxin	Peak4	20	2739223	0	0
536	549	NKCVNFNFNGLTGT	1.3691	14	ABCPRED	outside	Non-toxin	Peak4	14	2733014	0	0
533	550	LVKNKCVNFNFNGLTGTG	1.2501	18	ABCPRED	outside	Non-toxin	Peak4	18	2735520	0	0
537	550	KCVNFNFNGLTGTG	1.5894	14	ABCPRED	outside	Non-toxin	Peak4	14	2728975	0	0
541	550	FNFNGLTGTG	1.0567	10	ABCPRED	outside	Non-toxin	Peak4	10	2721751	0	0
530	551	STNLVKNKCVNFNFNGLTGTGV	1.0662	22	BCPREDS	outside	Non-toxin	Peak4	22	2742365	0	0
532	551	NLVKNKCVNFNFNGLTGTGV	1.084	20	ABCPRED	outside	Non-toxin	Peak4	20	2742297	0	0
540	551	NFNFNGLTGTGV	1.3547	12	\BCPRED,BCPRED	outside	Non-toxin	Peak4	12	2731133	0	0
535	552	KNKCVNFNFNGLTGTGVL	1.3849	18	ABCPRED	outside	Non-toxin	Peak4	18	2738751	0	0
537	552	KCVNFNFNGLTGTGVL	1.4136	16	AAP	outside	Non-toxin	Peak4	16	2734520	0	0
539	552	VNFNFNGLTGTGVL	1.2915	14	ABCPRED	outside	Non-toxin	Peak4	14	2734081	0	0
540	553	NFNFNGLTGTGVLT	1.1216	14	ABCPRED	outside	Non-toxin	Peak4	14	2737202	0	0
535	554	KNKCVNFNFNGLTGTGVLTE	1.2763	20	ABCPRED	outside	Non-toxin	Peak4	20	2756626	0	0
539	554	VNFNFNGLTGTGVLTE	1.1691	16	ABCPRED	outside	Non-toxin	Peak4	16	2751956	0	0
536	555	NKCVNFNFNGLTGTGVLTES	1.0131	20	ABCPRED	outside	Non-toxin	Peak4	20	2756530	0	0
538	555	CVNFNFNGLTGTGVLTES	1.1292	18	ABCPRED	outside	Non-toxin	Peak4	18	2752026	0	0
537	556	KCVNFNFNGLTGTGVLTESN	1.2355	20	ABCPRED	outside	Non-toxin	Peak4	20	2765767	0	0
538	557	CVNFNFNGLTGTGVLTESNK	1.1352	20	ABCPRED	outside	Non-toxin	Peak4	20	2765733	0	0
539	558	VNFNFNGLTGTGVLTESNKK	1.1457	20	ABCPRED	outside	Non-toxin	Peak4	20	2774117	0	0
541	558	FNFNGLTGTGVLTESNKK	0.9066	18	ABCPRED	outside	Non-toxin	Peak4	18	2767332	0	0
545	558	GLTGTGVLTESNKK	1.0227	14	IEDB	outside	Non-toxin	Peak4	14	2720417	0	0
540	559	NFNFNGLTGTGVLTESNKKF	0.9432	20	ABCPRED	outside	Non-toxin	Peak4	20	2774224	0	0

Supplementary Table 6. Differentially expressed genes in PBMCs of mice immunized with Epitope25-DMNA compared with those with Adjuvant-DMNA.

gene_id	baseMean	log2FoldChange	lfcSE	stat	pvalue	padj	Readcounts (Adjuvant-DMNA)	Readcounts (Adjuvant-DMNA)	Readcounts (Adjuvant-DMNA)	Readcounts (Adjuvant-DMNA)	Readcounts (Adjuvant-DMNA)	Readcounts (Epitope25-DMNA)	Readcounts (Epitope25-DMNA)	Readcounts (Epitope25-DMNA)	Readcounts (Epitope25-DMNA)	Readcounts (Epitope25-DMNA)
Ighv2-3	5.925269455	3.702046646	0.8783336	4.214852	2.50E-05	0.0061075	2	0	0	0	2	11	19	23	6	6
Maco1	4.908593218	3.427377289	0.8154801	4.202895	2.64E-05	0.0061075	0	0	1	2	1	11	7	16	11	6
Thy1	173.9937991	0.588499683	0.1477078	3.984216	6.77E-05	0.0111146	117	125	178	115	132	209	251	352	160	225
Tasp1	4.560348372	2.125987241	0.6118292	3.474805	0.0005112	0.0394946	2	2	1	2	1	8	9	9	7	8
Marcks	45.60181098	-1.403788835	0.2697103	-5.2048	1.94E-07	0.0007652	48	73	48	89	60	34	22	45	22	20
Cd33	123.9333717	-1.417876825	0.2894963	-4.89774	9.69E-07	0.0015188	144	216	121	272	115	67	89	112	63	54
D030028A08Rik	13.18604952	-2.583156823	0.5394567	-4.78844	1.68E-06	0.0015188	9	21	16	40	24	3	7	7	1	4
Itsn1	205.3370531	-0.948269284	0.1986294	-4.77406	1.81E-06	0.0015188	195	267	356	252	234	171	142	181	153	127
Nudt4	105.6286686	-0.874938951	0.1837765	-4.76089	1.93E-06	0.0015188	115	147	116	151	124	96	78	98	62	81
Ppp1r3d	74.47526773	-1.339514906	0.307578	-4.35504	1.33E-05	0.0061075	62	114	87	178	77	36	58	75	40	34
Trim10	341.2326876	-1.565341148	0.3602723	-4.34488	1.39E-05	0.0061075	411	412	415	752	454	293	125	178	129	206
Trem3	15.48415869	-1.465814004	0.341867	-4.28767	1.81E-05	0.0061075	17	31	17	27	18	6	12	17	7	6
Antxr2	67.7162512	-1.045642787	0.2439686	-4.28597	1.82E-05	0.0061075	90	109	59	104	73	43	60	73	31	46
Vopp1	547.1061779	-0.959082176	0.2243825	-4.27432	1.92E-05	0.0061075	618	642	723	815	660	519	318	432	376	380
Cdr2	5224.260825	-0.938108512	0.2199612	-4.26488	2.00E-05	0.0061075	5303	6964	7504	7088	6159	5015	3127	4221	3771	3375
Ybx3	179.5890375	-0.760540973	0.1786454	-4.25727	2.07E-05	0.0061075	205	221	192	235	222	160	139	163	138	134
Rab11fip1	52.12756111	-0.948054508	0.2232629	-4.24636	2.17E-05	0.0061075	72	80	53	75	48	30	40	69	33	34
Slc40a1	16.41731366	-1.688943748	0.3995694	-4.22691	2.37E-05	0.0061075	26	27	16	38	13	7	10	15	6	7
Metrnl	21.92712667	-1.327738074	0.3208802	-4.1378	3.51E-05	0.0074913	27	26	26	43	29	11	16	30	10	7
S100a11	168.6651549	-1.209630611	0.2928209	-4.13096	3.61E-05	0.0074913	234	262	156	339	135	106	126	188	86	82
Mxd1	729.0976508	-1.368546848	0.3337588	-4.10041	4.12E-05	0.0081247	912	1010	599	1879	642	351	497	722	402	367
Fech	207.0950133	-0.815972151	0.1997031	-4.08593	4.39E-05	0.0082367	190	353	206	238	280	158	191	217	132	146
Tmcc2	16.94246451	-1.443613024	0.3561551	-4.05333	5.05E-05	0.0090431	21	21	36	27	14	10	8	13	13	7
Rfk	157.7766973	-0.809178864	0.2020166	-4.00551	6.19E-05	0.0106011	184	221	215	173	168	163	117	173	84	108
Tlr4	24.75716243	-1.423413801	0.3608801	-3.94428	8.00E-05	0.0123612	29	45	23	56	21	10	17	25	10	16
AK157302	3130.184206	-0.647399719	0.1647817	-3.92883	8.54E-05	0.0123612	2878	4239	4560	3270	3456	3118	2572	3503	2092	2350
Dcaf12	483.5215403	-1.131260304	0.2885342	-3.92071	8.83E-05	0.0123612	559	646	776	614	591	480	379	460	171	220
Fos	203.2236792	-0.914572534	0.2336993	-3.91346	9.10E-05	0.0123612	263	224	213	342	223	147	182	257	131	94
Cmas	54.91925623	-1.272729458	0.3297447	-3.85974	0.0001135	0.0149072	75	54	56	132	54	36	33	55	23	37
Cers6	89.22704892	-0.816719813	0.2126167	-3.84128	0.0001224	0.0155561	89	113	94	163	88	61	58	110	68	71
Mpeg1	546.3548753	-0.82673939	0.217173	-3.80682	0.0001408	0.0173314	643	839	597	609	648	437	334	859	389	290
Mxi1	26.15455536	-0.988924134	0.2618442	-3.77676	0.0001589	0.0189691	28	26	43	36	33	19	20	24	22	13
Hebp1	27.43499048	-1.376501635	0.366681	-3.75395	0.0001741	0.0201717	36	28	41	46	37	22	15	14	22	9
Ifit1	70.25691319	-0.897680823	0.2420293	-3.70898	0.0002081	0.0229544	88	75	86	93	92	67	36	69	55	45
Lrrc25	48.65752594	-1.076445758	0.2929677	-3.67428	0.0002385	0.0253991	63	74	46	78	55	34	30	78	25	20
Slc25a39	425.7493268	-0.680310421	0.1871555	-3.635	0.000278	0.0288221	393	510	489	482	633	375	419	416	276	331
Tma7-ps	52.69905118	-0.633962837	0.175845	-3.60524	0.0003119	0.0315066	44	65	78	71	51	43	47	65	37	43
Rnf11	306.6570165	-0.712575767	0.1985813	-3.58833	0.0003328	0.0327807	316	371	431	372	337	334	243	307	183	222
Psme3	2609.865356	-0.712316646	0.2000608	-3.5605	0.0003701	0.0354687	2693	3417	3516	2995	2929	2464	1833	2337	2300	1844
Ctsb	126.7880868	-0.998223335	0.2810709	-3.5515	0.000383	0.0354687	115	191	145	234	131	114	113	133	50	71
Zfand5	68.76049081	-0.633057169	0.1783898	-3.54873	0.0003871	0.0354687	74	109	79	75	64	50	66	91	51	50
Ccr1	35.20844777	-1.258674124	0.3558944	-3.53665	0.0004052	0.0362871	31	71	36	68	35	23	28	44	15	12
Adipor1	72.65431586	-0.782359982	0.221842	-3.52665	0.0004208	0.0366287	57	111	76	106	93	51	76	84	38	56
Lmo2	4.275774032	-2.306838276	0.6563193	-3.51481	0.0004401	0.0366287	3	10	6	6	9	2	2	1	2	1
Rgs2	215.9134966	-0.729406909	0.2075649	-3.51412	0.0004412	0.0366287	238	251	203	375	222	128	197	308	156	153
Cript	82.8562532	-0.756290345	0.2154219	-3.51074	0.0004469	0.0366287	78	98	128	96	99	64	59	76	81	59
Slc7a11	27.39684776	-1.350476713	0.3852313	-3.50563	0.0004555	0.0366287	36	42	33	57	22	9	12	44	16	13
Msrb1	60.45743333	-1.231867745	0.3527427	-3.49226	0.000479	0.0377421	76	102	34	121	72	35	37	76	27	37
Pla2g7	33.94854817	-0.800148567	0.2316167	-3.45462	0.0005511	0.0412038	38	36	38	59	36	22	24	47	21	29
Gpr65	20.75655275	-0.889907038	0.2577155	-3.45306	0.0005543	0.0412038	22	28	25	34	21	10	17	27	13	17
Gm15707	6.162447648	-1.812424911	0.5265786	-3.44189	0.0005777	0.0418443	8	12	5	8	13	3	2	7	2	2
Alas2	3832.734089	-0.835004936	0.2428129	-3.43888	0.0005841	0.0418443	3587	5379	5551	4316	4808	3896	3538	3646	1710	2563
Cxcr2	146.7366145	-1.283871876	0.37812	-3.39541	0.0006853	0.0482132	164	250	138	341	110	81	106	128	112	51

Supplementary Table 7. Enriched GO termed for up-regualted genes in PBMCs of mice immunized with Epitope25-DMNA compared with those having Adjuvant-DMNA.

	ID	Description	pvalue	p.adjust	qvalue	Count	geneID
	GO:0050858	negative regulation of antigen receptor-mediated signaling pathway	0.004277626	0.038231282	0.007598415	1	Thy1
	GO:0050854	regulation of antigen receptor-mediated signaling pathway	0.00895342	0.039966098	0.007943207	1	Thy1
	GO:0050851	antigen receptor-mediated signaling pathway	0.000663464	0.032550912	0.006469449	2	Ighv2-3,Thy1
	GO:0042571	immunoglobulin complex, circulating	0.019164589	0.042978819	0.011310216	1	Ighv2-3
	GO:0019814	immunoglobulin complex	0.020669357	0.042978819	0.011310216	1	Ighv2-3
	GO:0002429	immune response-activating cell surface receptor signaling pathway	0.000856084	0.032550912	0.006469449	2	Ighv2-3,Thy1
	GO:0002757	immune response-activating signal transduction	0.000860964	0.032550912	0.006469449	2	Ighv2-3,Thy1
Activation of immune response	GO:0002768	immune response-regulating cell surface receptor signaling pathway	0.000910515	0.032550912	0.006469449	2	Ighv2-3,Thy1
	GO:0002253	activation of immune response	0.001405079	0.036051845	0.007165254	2	Ighv2-3,Thy1
	GO:0051251	positive regulation of lymphocyte activation	0.001512665	0.036051845	0.007165254	2	Ighv2-3,Thy1
	GO:0050856	regulation of T cell receptor signaling pathway	0.006617586	0.039966098	0.007943207	1	Thy1
	GO:0050860	negative regulation of T cell receptor signaling pathway	0.003450771	0.037958486	0.007544197	1	Thy1
	GO:0051894	positive regulation of focal adhesion assembly	0.003864263	0.038231282	0.007598415	1	Thy1
	GO:0090109	regulation of cell-substrate junction assembly	0.008816132	0.039966098	0.007943207	1	Thy1
	GO:0150116	regulation of cell-substrate junction organization	0.009502429	0.039966098	0.007943207	1	Thy1
	GO:0007044	cell-substrate junction assembly	0.013202274	0.043905236	0.008726105	1	Thy1
	GO:0150115	cell-substrate junction organization	0.014023052	0.045431198	0.009029388	1	Thy1
Positive regulation of cell matrix adhesion	GO:0150117	positive regulation of cell-substrate junction organization	0.00455313	0.038299857	0.007612044	1	Thy1
	GO:0001952	regulation of cell-matrix adhesion	0.017573842	0.048328067	0.009605137	1	Thy1
	GO:0048041	focal adhesion assembly	0.011833172	0.040755497	0.008100099	1	Thy1
	GO:0001954	positive regulation of cell-matrix adhesion	0.008816132	0.039966098	0.007943207	1	Thy1
	GO:0051893	regulation of focal adhesion assembly	0.008816132	0.039966098	0.007943207	1	Thy1
	GO:1901890	positive regulation of cell junction assembly	0.017164626	0.048128265	0.009565426	1	Thy1
	GO:0051281	positive regulation of release of sequestered calcium ion into cytosol	0.005929791	0.039966098	0.007943207	1	Thy1
	GO:0051279	regulation of release of sequestered calcium ion into cytosol	0.011285132	0.040755497	0.008100099	1	Thy1
	GO:1904427	positive regulation of calcium ion transmembrane transport	0.014706643	0.045718476	0.009086484	1	Thy1
Positive regulation of calcium ion	GO:0051209	release of sequestered calcium ion into cytosol	0.016482315	0.048128265	0.009565426	1	Thy1
	GO:0051283	negative regulation of sequestering of calcium ion	0.016618805	0.048128265	0.009565426	1	Thy1
	GO:0051282	regulation of sequestering of calcium ion	0.016891744	0.048128265	0.009565426	1	Thy1
	GO:0051208	sequestering of calcium ion	0.017164626	0.048128265	0.009565426	1	Thy1
	GO:0001754	eye photoreceptor cell differentiation	0.008541514	0.039966098	0.007943207	1	Thy1
	GO:0046549	retinal cone cell development	0.002485457	0.037958486	0.007544197	1	Thy1
	GO:0042670	retinal cone cell differentiation	0.002761333	0.037958486	0.007544197	1	Thy1
	GO:0060042	retina morphogenesis in camera-type eye	0.01155918	0.040755497	0.008100099	1	Thy1
Photoreceptor cell differentiation	GO:0060219	camera-type eye photoreceptor cell differentiation	0.005792189	0.039966098	0.007943207	1	Thy1
	GO:0042462	eye photoreceptor cell development	0.006342511	0.039966098	0.007943207	1	Thy1
	GO:0042461	photoreceptor cell development	0.009090694	0.039966098	0.007943207	1	Thy1
	GO:0046530	photoreceptor cell differentiation	0.011696183	0.040755497	0.008100099	1	Thy1
	GO:0003407	neural retina development	0.011970146	0.040755497	0.008100099	1	Thy1
	GO:0070571	negative regulation of neuron projection regeneration	0.002347498	0.037958486	0.007544197	1	Thy1
Neuron projection regeneration	GO:0070570	regulation of neuron projection regeneration	0.005379298	0.039966098	0.007943207	1	Thy1
	GO:0031102	neuron projection regeneration	0.008541514	0.039966098	0.007943207	1	Thy1
	GO:0002693	positive regulation of cellular extravasation	0.003175039	0.037958486	0.007544197	1	Thy1

Cellular extravasation	GO:0002691	regulation of cellular extravasation	0.006342511	0.039966098	0.007943207	1	Thy1
	GO:0045123	cellular extravasation	0.011148086	0.040755497	0.008100099	1	Thy1
Heterotypic cell-cell adhesion	GO:0034113	heterotypic cell-cell adhesion	0.007854718	0.039966098	0.007943207	1	Thy1
	GO:0034116	positive regulation of heterotypic cell-cell adhesion	0.001933534	0.037958486	0.007544197	1	Thy1
	GO:0034114	regulation of heterotypic cell-cell adhesion	0.003450771	0.037958486	0.007544197	1	Thy1
Protein tyrosine kinase activity	GO:0050732	negative regulation of peptidyl-tyrosine phosphorylation	0.007854718	0.039966098	0.007943207	1	Thy1
	GO:0061099	negative regulation of protein tyrosine kinase activity	0.004139853	0.038231282	0.007598415	1	Thy1
	GO:0061097	regulation of protein tyrosine kinase activity	0.010736864	0.040755497	0.008100099	1	Thy1
dendrite membrane	GO:0030673	axolemma	0.002774645	0.026515084	0.006977654	1	Thy1
	GO:0032590	dendrite membrane	0.006925781	0.027703125	0.007290296	1	Thy1
	GO:0032589	neuron projection membrane	0.009823856	0.031316389	0.008241155	1	Thy1
	GO:0044304	main axon	0.01161472	0.031316389	0.008241155	1	Thy1
	GO:0031256	leading edge membrane	0.02148941	0.042978819	0.011310216	1	Thy1
plasma membrane	GO:0031362	anchored component of external side of plasma membrane	0.003190343	0.026515084	0.006977654	1	Thy1
	GO:0031233	intrinsic component of external side of plasma membrane	0.003882885	0.026515084	0.006977654	1	Thy1
	GO:0046658	anchored component of plasma membrane	0.01120166	0.031316389	0.008241155	1	Thy1
	GO:0031225	anchored component of membrane	0.027215366	0.043511787	0.01145047	1	Thy1
other	GO:0032809	neuronal cell body membrane	0.005266885	0.026515084	0.006977654	1	Thy1
	GO:0044298	cell body membrane	0.005681804	0.026515084	0.006977654	1	Thy1
	GO:0030426	growth cone	0.027759381	0.043511787	0.01145047	1	Thy1
	GO:0030427	site of polarized growth	0.028710859	0.043511787	0.01145047	1	Thy1
	GO:0043209	myelin sheath	0.029525856	0.043511787	0.01145047	1	Thy1
	GO:0051861	glycolipid binding	0.004218291	0.021091455	0.007400511	1	Thy1
	GO:0030867	rough endoplasmic reticulum membrane	0.002358817	0.026515084	0.006977654	1	Maco1
	GO:0005791	rough endoplasmic reticulum	0.012302867	0.031316389	0.008241155	1	Maco1
	GO:0044306	neuron projection terminus	0.029525856	0.043511787	0.01145047	1	Maco1
	GO:0031965	nuclear membrane	0.033728504	0.047219905	0.012426291	1	Maco1
	GO:0004298	threonine-type endopeptidase activity	0.0019702	0.020048527	0.007034571	1	Tasp1
	GO:0070003	threonine-type peptidase activity	0.002673137	0.020048527	0.007034571	1	Tasp1
	GO:0050771	negative regulation of axonogenesis	0.009365198	0.039966098	0.007943207	1	Thy1
	GO:0043113	receptor clustering	0.010599762	0.040755497	0.008100099	1	Thy1
	GO:0007229	integrin-mediated signaling pathway	0.014296531	0.045431198	0.009029388	1	Thy1

Supplementary Table 8. Enriched GO termed for down-regualted genes in PBMCs of mice immunized with Epitope25-DMNA compared with those having Adjuvant-DMNA.

	ID	Description	pvalue	p.adjust	qvalue	Count	geneID
Pigment metabolism	GO:0006783	heme biosynthetic process	9.52E-06	0.0013555	0.000937	3	Fech,Slc25a39,Alas2
	GO:0006779	porphyrin-containing compound biosynthetic process	1.64E-05	0.0018644	0.001289	3	Fech,Slc25a39,Alas2
	GO:0033014	tetrapyrrole biosynthetic process	1.64E-05	0.0018644	0.001289	3	Fech,Slc25a39,Alas2
	GO:0046148	pigment biosynthetic process	1.43E-06	0.0002931	0.000203	4	Fech,Slc25a39,Slc7a11,Alas2
	GO:0042168	heme metabolic process	3.17E-07	0.0001804	0.000125	4	Fech,Hebp1,Slc25a39,Alas2
	GO:0006778	porphyrin-containing compound metabolic process	6.58E-07	0.00025	0.000173	4	Fech,Hebp1,Slc25a39,Alas2
	GO:0033013	tetrapyrrole metabolic process	1.54E-06	0.0002931	0.000203	4	Fech,Hebp1,Slc25a39,Alas2
	GO:0042440	pigment metabolic process	7.84E-08	8.93E-05	6.17E-05	5	Fech,Hebp1,Slc25a39,Slc7a11,Alas2
	GO:0042541	hemoglobin biosynthetic process	0.0002924	0.0175262	0.012116	2	Fech,Alas2
	GO:0020027	hemoglobin metabolic process	0.000612	0.026812	0.018535	2	Fech,Alas2
Cell homeostasis	GO:0001776	leukocyte homeostasis	0.0012196	0.0356171	0.024621	3	Slc40a1,Slc7a11,Cxcr2
	GO:0001781	neutrophil apoptotic process	0.0001615	0.0122595	0.008475	2	Slc7a11,Cxcr2
	GO:0006925	inflammatory cell apoptotic process	0.0009098	0.0324932	0.022462	2	Slc7a11,Cxcr2
	GO:0001780	neutrophil homeostasis	0.0009762	0.0336945	0.023292	2	Slc7a11,Cxcr2
	GO:0033032	regulation of myeloid cell apoptotic process	0.0017638	0.0434463	0.030034	2	Slc7a11,Cxcr2
	GO:0045672	positive regulation of osteoclast differentiation	0.001422	0.037667	0.026038	2	Fos,Ccr1
	GO:0030099	myeloid cell differentiation	7.11E-05	0.0073604	0.005088	6	Trim10,Fech,Fos,Ccr1,Lmo2,Alas2
	GO:0030218	erythrocyte differentiation	9.55E-05	0.0090624	0.006265	4	Trim10,Fech,Lmo2,Alas2
	GO:0034101	erythrocyte homeostasis	0.0001285	0.0109571	0.007574	4	Trim10,Fech,Lmo2,Alas2
	GO:0002262	myeloid cell homeostasis	8.93E-07	0.0002543	0.000176	6	Trim10,Fech,Lmo2,Slc7a11,Alas2,Cxcr2
	GO:0048872	homeostasis of number of cells	2.01E-06	0.0003268	0.000226	7	Trim10,Slc40a1,Fech,Lmo2,Slc7a11,Alas2,Cxcr2
	GO:0055072	iron ion homeostasis	0.0004411	0.0239229	0.016537	3	Slc40a1,Fech,Alas2
	GO:0055076	transition metal ion homeostasis	0.0013571	0.037667	0.026038	3	Slc40a1,Fech,Alas2
Cell chemotaxis	GO:0016493	C-C chemokine receptor activity	0.0006334	0.0189071	0.013477	2	Ccr1,Cxcr2
	GO:0001637	G protein-coupled chemoattractant receptor activity	0.0006878	0.0189071	0.013477	2	Ccr1,Cxcr2
	GO:0004950	chemokine receptor activity	0.0006878	0.0189071	0.013477	2	Ccr1,Cxcr2
	GO:0019957	C-C chemokine binding	0.0006878	0.0189071	0.013477	2	Ccr1,Cxcr2
	GO:0019956	chemokine binding	0.0012748	0.026984	0.019234	2	Ccr1,Cxcr2
	GO:0090026	positive regulation of monocyte chemotaxis	0.0005594	0.0265475	0.018352	2	Ccr1,Pla2g7
	GO:0090025	regulation of monocyte chemotaxis	0.0010449	0.033746	0.023328	2	Ccr1,Pla2g7
	GO:0002690	positive regulation of leukocyte chemotaxis	0.0005296	0.0262261	0.01813	3	Ccr1,Pla2g7,Cxcr2
	GO:0002688	regulation of leukocyte chemotaxis	0.0010666	0.033746	0.023328	3	Ccr1,Pla2g7,Cxcr2
	GO:0050921	positive regulation of chemotaxis	0.0017928	0.0434463	0.030034	3	Ccr1,Pla2g7,Cxcr2
	GO:0002687	positive regulation of leukocyte migration	0.0021141	0.0481587	0.033291	3	Ccr1,Pla2g7,Cxcr2
	GO:0030595	leukocyte chemotaxis	0.0004722	0.0244454	0.016899	4	Trem3,Ccr1,Pla2g7,Cxcr2
	GO:0097529	myeloid leukocyte migration	0.0005997	0.026812	0.018535	4	Trem3,Ccr1,Pla2g7,Cxcr2
	GO:0060326	cell chemotaxis	0.0015492	0.0401023	0.027722	4	Trem3,Ccr1,Pla2g7,Cxcr2
	GO:0032103	positive regulation of response to external stimulus	0.0007877	0.0309364	0.021386	5	Trem3,Tlr4,Ccr1,Pla2g7,Cxcr2
synapse organization	GO:0099159	regulation of modification of postsynaptic structure	0.0001906	0.0135692	0.00938	2	Marcks,Itsn1
	GO:0098885	modification of postsynaptic actin cytoskeleton	0.0002924	0.0175262	0.012116	2	Marcks,Itsn1
	GO:1905244	regulation of modification of synaptic structure	0.000372	0.0211857	0.014645	2	Marcks,Itsn1
	GO:0099010	modification of postsynaptic structure	0.0007838	0.0309364	0.021386	2	Marcks,Itsn1
	GO:0099563	modification of synaptic structure	0.0013421	0.037667	0.026038	2	Marcks,Itsn1
	GO:0050807	regulation of synapse organization	0.0008171	0.0310228	0.021445	4	Marcks,Itsn1,Cript,Slc7a11
	GO:0050803	regulation of synapse structure or activity	0.0009129	0.0324932	0.022462	4	Marcks,Itsn1,Cript,Slc7a11

	GO:1903203	regulation of oxidative stress-induced neuron death	0.001422	0.037667	0.026038	2	Tlr4,Slc7a11
cellular response to chemical st	GO:0036475	neuron death in response to oxidative stress	0.0015885	0.0402065	0.027794	2	Tlr4,Slc7a11
	GO:0062197	cellular response to chemical stress	0.0020697	0.0481105	0.033258	4	Ybx3,Tlr4,Fos,Slc7a11
	GO:0035672	oligopeptide transmembrane transport	0.0001347	0.0109571	0.007574	2	Slc25a39,Slc7a11
oligopeptide transport	GO:0006857	oligopeptide transport	0.0002924	0.0175262	0.012116	2	Slc25a39,Slc7a11
	GO:0072337	modified amino acid transport	0.001189	0.0356171	0.024621	2	Slc25a39,Slc7a11
	GO:0051537	2 iron, 2 sulfur cluster binding	0.0007444	0.0189071	0.013477	2	Fech,AK157302
	GO:0071827	plasma lipoprotein particle organization	0.0019479	0.0462223	0.031953	2	Fech,Pla2g7
Other	GO:0046628	positive regulation of insulin receptor signaling pathway	0.0007243	0.0305535	0.021121	2	Marcks,Adipor1
	GO:1900078	positive regulation of cellular response to insulin stimulus	0.0010449	0.033746	0.023328	2	Marcks,Adipor1
	GO:0099571	postsynaptic cytoskeleton	0.0004378	0.0446554	0.035485	2	Marcks,Itsn1
	GO:0050829	defense response to Gram-negative bacterium	0.0011932	0.0356171	0.024621	3	Trem3,Tlr4,Mpeg1

Supplementary Table 9. Differentially expressed genes in PBMCs of mice immunized with Epitope25-Sub-Q compared with those having Adjuvant-DMNA.

gene_id	baseMean	log2FoldChange	lfcSE	stat	pvalue	padj	Readcounts (Adjuvant-DMNA)	Readcounts (Adjuvant-DMNA)	Readcounts (Adjuvant-DMNA)	Readcounts (Adjuvant-DMNA)	Readcounts (Adjuvant-DMNA)	Readcounts (Epitope25-Sub-Q)	Readcounts (Epitope25-Sub-Q)	Readcounts (Epitope25-Sub-Q)	Readcounts (Epitope25-Sub-Q)	Readcounts (Epitope25-Sub-Q)
H1f0	33.46749897	1.202185995	0.234275	5.131512	2.87E-07	0.000166	10	19	13	16	17	48	80	52	72	101
Cul3	23.58236632	1.734638221	0.361498	4.798468	1.60E-06	0.000527	2	13	11	10	5	48	50	32	60	86
Il6st	67.64843687	0.871420446	0.184138	4.732439	2.22E-06	0.000668	28	27	46	39	37	98	130	92	167	172
Gm38244	52.30737652	1.380810467	0.299397	4.611966	3.99E-06	0.001023	15	27	35	17	15	70	158	51	161	162
Malat1	1022.307437	0.866274804	0.188703	4.59068	4.42E-06	0.001059	530	485	709	581	383	1348	2829	1074	2225	2785
Vasn	15.3006338	1.660374548	0.365685	4.540449	5.61E-06	0.001253	7	9	3	3	5	28	37	20	46	45
Gpcpd1	36.21334036	1.228437106	0.290973	4.221825	2.42E-05	0.003728	12	16	15	23	14	71	66	60	71	100
Zfp729a	26.60176421	1.094006445	0.265695	4.117525	3.83E-05	0.005218	12	9	15	15	12	39	67	30	78	64
Iws1	38.02030489	1.074686175	0.269732	3.984281	6.77E-05	0.008676	18	21	23	11	18	46	96	37	111	120
Runx1	40.7305909	1.03212123	0.262614	3.930182	8.49E-05	0.010308	24	15	25	13	21	53	83	60	117	100
Faf2	35.03694502	1.023831167	0.268188	3.817581	0.000135	0.015046	15	12	29	17	13	41	78	53	78	111
Gm43197	16.63582529	1.246675464	0.327957	3.801339	0.000144	0.015326	5	8	12	8	4	25	48	19	45	44
Zbp1	4.130228646	3.822831779	1.019597	3.749356	0.000177	0.016812	1	0	1	0	0	7	9	5	24	17
Uspl1	2.531587717	3.742355048	1.004568	3.725339	0.000195	0.017765	0	0	0	1	0	4	9	3	12	11
Ddx17	69.17225187	0.922961327	0.248106	3.720034	0.000199	0.017907	36	34	38	53	17	88	165	81	180	188
Zfp512	1.745525061	3.870369748	1.056215	3.664377	0.000248	0.020191	0	0	0	0	0	2	9	2	8	8
Vcan	3.261367643	4.043806125	1.128279	3.584049	0.000338	0.02602	1	0	0	0	0	7	3	10	6	19
Gimap6	86.28924599	0.861303253	0.24066	3.578921	0.000345	0.026196	43	42	47	47	48	118	195	63	239	275
Ccar2	6.274825691	2.060054111	0.580404	3.549344	0.000386	0.028744	3	1	4	1	0	9	17	11	15	26
Gm45223	12.97896669	2.022343983	0.571306	3.539861	0.0004	0.029349	2	5	5	5	2	18	28	44	20	32
Hps5	3.003737656	3.964020055	1.13792	3.483567	0.000495	0.034271	0	0	1	0	0	5	2	6	12	21
Gm7061	2.575915541	3.772621514	1.095062	3.445121	0.000571	0.037991	0	0	0	1	0	4	12	1	9	16
Zbtb37	32.18162528	1.04113536	0.303467	3.430804	0.000602	0.03929	19	13	17	16	13	41	102	26	79	97
Rnf166	9.8395114	1.427343344	0.417488	3.418888	0.000629	0.0403	1	4	5	5	5	11	24	16	32	28
Ptar1	50.61043598	0.806420291	0.236608	3.408253	0.000654	0.041519	23	28	28	26	32	66	110	41	136	159
Kcnq1ot1	9.916495338	1.430068716	0.42283	3.382138	0.000719	0.044452	4	2	7	3	4	15	28	11	32	26
Wdr5b	8.021181764	1.563556838	0.463327	3.374629	0.000739	0.045278	2	2	6	3	2	13	23	13	21	20
Ankrd44	14.1954419	1.380663596	0.41234	3.34836	0.000813	0.048509	8	6	9	3	3	18	24	24	48	42
Isca1	23.07144872	-1.931621362	0.282023	-6.84916	7.43E-12	3.57E-08	21	35	29	22	28	12	19	10	15	16
Gm10371	201.3515209	-1.573129357	0.231278	-6.8019	1.03E-11	3.57E-08	200	242	249	216	207	109	170	148	166	132
Gm8730	3586.14949	-1.107169607	0.164315	-6.73807	1.61E-11	3.70E-08	3337	3868	3871	3475	3549	2691	3874	2731	3863	3368
Rpl39	744.1025526	-0.928868329	0.145213	-6.39658	1.59E-10	2.75E-07	588	813	777	726	715	630	859	498	1027	801
Ube2l6	19714.11699	-1.63168141	0.268956	-6.06673	1.31E-09	1.81E-06	19986	23515	24970	19713	22004	13281	13490	13386	14733	11901
Rfk	177.5585016	-1.459156938	0.254627	-5.73056	1.00E-08	1.15E-05	184	221	215	173	168	110	124	135	183	124
Gm28723	14.75857043	-1.8271283	0.337047	-5.42099	5.93E-08	5.49E-05	14	14	21	17	19	4	10	10	14	11
Ube2e3	14.95252213	-2.103914564	0.392374	-5.36202	8.23E-08	6.33E-05	18	20	20	16	15	4	5	11	7	15
AK157302	3357.439512	-1.485971924	0.280025	-5.30657	1.12E-07	7.73E-05	2878	4239	4560	3270	3456	2204	2363	2575	3090	2109
Psme3	2859.444352	-1.461489387	0.284615	-5.13497	2.82E-07	0.000166	2693	3417	3516	2995	2929	2072	1941	2286	2251	1933
Smap1	58.50997447	-1.268274576	0.249291	-5.08753	3.63E-07	0.000193	65	68	54	59	57	35	38	48	54	80
Cdr2	5879.468623	-1.625436175	0.320756	-5.06752	4.03E-07	0.000199	5303	6964	7504	7088	6159	3896	3606	4436	4738	2948
Ghitm	56.15724485	-1.57262723	0.312368	-5.03453	4.79E-07	0.000221	56	78	56	65	55	36	29	38	65	35
Paqr9	1567.64954	-1.436145562	0.288379	-4.98006	6.36E-07	0.000275	1529	1589	1898	1760	1691	1119	1139	1250	1386	949
Gm15772	573.4322467	-0.592607197	0.120238	-4.92863	8.28E-07	0.000337	422	503	574	504	552	522	857	371	863	896
Cst3	359.0510206	-0.659535296	0.136516	-4.83118	1.36E-06	0.000497	277	369	334	325	321	347	447	312	423	523
Gm23823	45.63681149	-1.38646363	0.287046	-4.83011	1.36E-06	0.000497	44	39	68	45	47	28	37	40	38	38
Hbb-bt	3268.816328	-1.515700568	0.314469	-4.81987	1.44E-06	0.000497	3877	2855	3777	3175	4048	2463	2250	2378	2505	2061
Gm9625	280.7335985	-1.153623425	0.241266	-4.78155	1.74E-06	0.000547	299	285	306	316	223	215	300	229	276	221
Cript	97.0223158	-1.18467358	0.252684	-4.68835	2.75E-06	0.000791	78	98	128	96	99	85	95	75	85	81
Rexo2	64.53609317	-1.155950799	0.246956	-4.6808	2.86E-06	0.000791	66	55	84	57	66	40	94	44	53	69
Ube2o	399.4246175	-1.470918105	0.314905	-4.67099	3.00E-06	0.000798	376	511	461	402	422	320	252	311	301	259
Dcaf12	572.1854389	-1.594562764	0.347419	-4.58973	4.44E-06	0.001059	559	646	776	614	591	406	281	449	475	317
Scand1	254.0283871	-0.624135991	0.136932	-4.55799	5.16E-06	0.001192	222	240	220	249	206	216	396	210	340	336
Ndufa2	65.89028031	-1.095925481	0.241885	-4.53077	5.88E-06	0.001271	58	83	58	73	60	57	85	36	78	56
Itsn1	235.9614999	-1.521707648	0.34156	-4.45516	8.38E-06	0.001707	195	267	356	252	234	188	172	177	161	129

Slc48a1	3888.504323	-1.280350006	0.288298	-4.44107	8.95E-06	0.00177	3405	4704	4155	4111	4044	2850	3027	3533	3531	2696
Slc25a39	484.7906765	-1.223891092	0.279689	-4.37591	1.21E-05	0.002325	393	510	489	482	633	295	364	462	526	415
Hbb-bs	521.3591239	-1.337800117	0.306903	-4.35904	1.31E-05	0.002444	483	598	693	479	515	474	393	393	389	377
Gm10736	770.2324971	-1.280099514	0.295941	-4.32552	1.52E-05	0.002772	785	754	1058	746	693	650	747	598	456	685
Gm5526	37.91710397	-1.448183794	0.336022	-4.30978	1.63E-05	0.0029	45	42	38	55	24	25	31	32	27	29
Rplp1	505.2427345	-0.655250625	0.15271	-4.29083	1.78E-05	0.003005	377	439	538	528	416	419	854	388	642	654
Oaz1-ps	769.9838483	-1.280380704	0.298825	-4.28472	1.83E-05	0.003016	709	861	769	823	866	569	598	719	651	545
Tpt1-ps3	411.1607025	-0.924798577	0.21859	-4.23075	2.33E-05	0.003664	369	465	382	394	378	353	419	384	458	405
Gm11808	126.2478961	-0.847039122	0.201763	-4.19819	2.69E-05	0.004022	104	117	133	118	126	111	125	122	138	155
Oaz1	1915.942262	-1.37152009	0.326955	-4.19483	2.73E-05	0.004022	1541	2492	1558	1974	2640	1220	1237	1698	1896	1404
Mxi1	29.61507509	-1.661618679	0.397232	-4.18299	2.88E-05	0.004149	28	26	43	36	33	9	17	31	19	25
Alas2	4450.566644	-1.324270973	0.320655	-4.12989	3.63E-05	0.005127	3587	5379	5551	4316	4808	3225	2750	4042	4402	3032
Pim1	469.0207077	-0.645255495	0.156744	-4.11663	3.84E-05	0.005218	410	514	403	397	388	333	506	403	717	850
Gm10221	16.44189097	-1.625635856	0.398726	-4.07708	4.56E-05	0.006071	15	17	13	27	19	14	13	10	12	8
Ucp2	13468.75826	-1.018694534	0.250669	-4.0639	4.83E-05	0.006303	11660	14225	13505	13214	14069	11606	12827	12788	12788	12100
Hba-a1	201.2797548	-1.504772622	0.381654	-3.94277	8.05E-05	0.010137	208	284	231	114	255	172	183	123	127	122
Gm14165	348.9507082	-0.914109351	0.232201	-3.93671	8.26E-05	0.010211	290	441	255	343	354	275	415	313	396	351
Dnajb1	50.89899917	-1.12089902	0.29031	-3.86105	0.000113	0.013474	57	43	37	74	45	29	46	43	68	52
Cyp4b1-ps	31.30024951	-1.960723276	0.51157	-3.83275	0.000127	0.014867	44	28	40	44	26	28	10	19	14	12
Rnf11	352.1969521	-1.220866962	0.319225	-3.82447	0.000131	0.014921	316	371	431	372	337	241	265	375	299	265
Rplp0	31.59763674	-1.33054794	0.347978	-3.82365	0.000131	0.014921	25	30	52	30	31	11	32	31	32	26
Hebp1	34.17409211	-1.580870704	0.415778	-3.8022	0.000143	0.015326	36	28	41	46	37	27	16	29	14	31
Metap2	62.03341237	-1.186773659	0.313645	-3.78381	0.000154	0.016198	68	58	73	67	51	41	59	54	80	37
Gm46515	32.36236111	-1.602622821	0.425621	-3.76538	0.000166	0.016812	33	32	28	62	25	24	17	27	17	25
Hectd4	36.08822034	-1.16277548	0.309184	-3.76079	0.000169	0.016812	37	46	45	25	30	24	32	35	36	33
Rps12	733.7309642	-0.887647517	0.236146	-3.75889	0.000171	0.016812	549	728	552	745	939	467	752	699	985	903
Uba52	87.4481596	-0.873484228	0.232416	-3.75828	0.000171	0.016812	50	100	82	94	95	67	80	80	94	138
Gm27477	4.292822605	-2.82598084	0.753037	-3.75278	0.000175	0.016812	3	4	4	10	7	3	2	0	0	3
2310026L	2.91467245	-3.757603947	1.002204	-3.74934	0.000177	0.016812	5	2	4	7	2	1	0	0	2	0
Ube2r2	1268.197008	-1.166470602	0.312133	-3.7371	0.000186	0.017291	999	1699	1254	1263	1300	894	1033	1281	1293	900
Trir	178.1589965	-0.876693435	0.234693	-3.7355	0.000187	0.017291	104	182	161	223	190	152	219	148	219	177
Plcl2	58.65487371	-0.964361928	0.260119	-3.70739	0.000209	0.018583	70	65	49	57	43	39	62	46	91	59
Rps16-ps2	38.12259623	-0.921911759	0.249573	-3.69396	0.000221	0.019346	29	32	34	43	47	26	56	23	35	64
Atp5b	115.6927823	-0.975764017	0.264989	-3.68228	0.000231	0.019612	79	133	151	111	98	80	109	120	136	114
Trim58	30.37984692	-1.390827932	0.37784	-3.68099	0.000232	0.019612	24	41	31	41	26	26	16	24	33	18
H3c4	9.242140872	-1.77843468	0.483787	-3.67607	0.000237	0.019753	4	13	19	7	11	4	7	4	11	6
Napa	387.0266748	-0.862843136	0.235033	-3.67116	0.000241	0.019897	296	426	353	383	391	365	386	365	446	380
Smim7	58.91283615	-0.678857956	0.185982	-3.65013	0.000262	0.021096	52	54	63	45	54	35	76	52	85	100
Fam104a	41.67583526	-0.905842901	0.24906	-3.63705	0.000276	0.021755	32	50	40	53	27	41	46	28	53	47
Gm10180	59.28430534	-1.224434982	0.336725	-3.6363	0.000277	0.021755	41	82	56	55	73	33	47	64	54	53
Rpl19-ps1	131.5344123	-0.629414075	0.173254	-3.63289	0.00028	0.021797	119	107	139	105	120	107	212	77	190	218
Rpl10a-ps	21.33142143	-1.01621009	0.284132	-3.57655	0.000348	0.026196	23	13	20	25	24	16	24	11	29	28
Hyal3	5.154860743	-2.171109537	0.613679	-3.53786	0.000403	0.029349	5	5	10	7	5	1	7	0	5	2
Ncoa4-ps	13.96113023	-1.602505188	0.453264	-3.53548	0.000407	0.029349	10	21	18	18	11	8	8	12	16	5
H3c6	2.723280871	-3.618616032	1.033493	-3.50135	0.000463	0.033034	7	2	4	3	2	1	2	0	0	0
Gm2000	58.10939295	-0.755669032	0.216305	-3.49354	0.000477	0.033668	45	64	47	53	60	49	76	46	89	62
Stip1	38.35897026	-1.244091873	0.357423	-3.48073	0.0005	0.034271	22	34	54	58	34	28	25	37	35	36
Fhdc1	3.450494221	-3.676661297	1.058066	-3.47489	0.000511	0.034682	5	7	7	3	2	0	0	0	4	0
Reep5	17.64784544	-1.00156509	0.288882	-3.46704	0.000526	0.035364	17	15	23	18	14	14	25	11	16	23
Hba-a2	2386.154232	-0.966402966	0.281122	-3.43767	0.000587	0.038681	2449	2523	2069	2324	2237	2230	2436	2270	2253	2006
Vangl1	40.44485164	-1.391481241	0.405879	-3.42832	0.000607	0.03929	44	37	54	33	46	31	23	39	36	22
Fundc2	52.45760605	-1.267332023	0.374333	-3.38557	0.00071	0.044417	39	53	75	52	56	46	27	45	60	35
Gm15610	11.35627145	-1.31408544	0.38823	-3.38481	0.000712	0.044417	9	20	9	13	9	8	8	8	9	16
Ube2c	244.577066	-1.246269131	0.369681	-3.3712	0.000748	0.045443	255	228	274	235	272	193	135	253	221	174
Cited4	5.964140859	-2.03719254	0.605796	-3.36283	0.000771	0.046436	9	9	6	4	7	5	3	0	2	8

Supplementary Table 10. Enriched GO termed for up-regualted genes in PBMCs of mice immunized with Epitope25-DMNA compared with those having Adjuvant-DMNA.

Summarized GO Category	ID	Description	pvalue	p.adjust	qvalue	Count	geneID
	GO:0050870	positive regulation of T cell activation	0.017558	0.123525	0.084084	2	Il6st/Runx1
	GO:1903039	positive regulation of leukocyte cell-cell adhesion	0.021196	0.123525	0.084084	2	Il6st/Runx1
T cell activation	GO:0022409	positive regulation of cell-cell adhesion	0.029318	0.123525	0.084084	2	Il6st/Runx1
	GO:1903037	regulation of leukocyte cell-cell adhesion	0.037563	0.134154	0.091319	2	Il6st/Runx1
	GO:0050863	regulation of T cell activation	0.03886	0.136351	0.092814	2	Il6st/Runx1
	GO:0007159	leukocyte cell-cell adhesion	0.045201	0.139259	0.094794	2	Il6st/Runx1
	GO:0001959	regulation of cytokine-mediated signaling pathway	0.005734	0.123525	0.084084	2	Il6st/Zbp1
Response to cytokine stimulus	GO:0060759	regulation of response to cytokine stimulus	0.006572	0.123525	0.084084	2	Il6st/Zbp1
	GO:0019221	cytokine-mediated signaling pathway	0.047194	0.139259	0.094794	2	Il6st/Zbp1
	GO:0051607	defense response to virus	0.02373	0.123525	0.084084	2	Zbp1/Ddx17
	GO:0140546	defense response to symbiont	0.023883	0.123525	0.084084	2	Zbp1/Ddx17
Defense response	GO:0009615	response to virus	0.033251	0.129533	0.088174	2	Zbp1/Ddx17
	GO:0035966	response to topologically incorrect protein	0.005815	0.123525	0.084084	2	Cul3/Faf2
	GO:0071560	cellular response to transforming growth factor beta stimulus	0.017025	0.123525	0.084084	2	Vasn/Runx1
	GO:0071559	response to transforming growth factor beta	0.017558	0.123525	0.084084	2	Vasn/Runx1
	GO:0001889	liver development	0.005734	0.123525	0.084084	2	Cul3/Runx1
	GO:0061008	hepaticobiliary system development	0.00598	0.123525	0.084084	2	Cul3/Runx1
	GO:0001825	blastocyst formation	0.001095	0.123525	0.084084	2	Cul3/Uspl1
	GO:0001824	blastocyst development	0.007832	0.123525	0.084084	2	Cul3/Uspl1
	GO:0007519	skeletal muscle tissue development	0.012668	0.123525	0.084084	2	Gpcpd1/Ddx17
Development	GO:0060538	skeletal muscle organ development	0.014098	0.123525	0.084084	2	Gpcpd1/Ddx17
	GO:0007517	muscle organ development	0.037563	0.134154	0.091319	2	Gpcpd1/Ddx17
	GO:0001837	epithelial to mesenchymal transition	0.00728	0.123525	0.084084	2	Vasn/Ddx17
	GO:0048762	mesenchymal cell differentiation	0.017424	0.123525	0.084084	2	Vasn/Ddx17
	GO:0060485	mesenchyme development	0.026065	0.123525	0.084084	2	Vasn/Ddx17
	GO:0000380	alternative mRNA splicing, via spliceosome	0.001953	0.123525	0.084084	2	Malat1/Ddx17
	GO:0000398	mRNA splicing, via spliceosome	0.023425	0.123525	0.084084	2	Malat1/Ddx17
	GO:0048024	regulation of mRNA splicing, via spliceosome	0.004149	0.123525	0.084084	2	Malat1/Ddx17
	GO:0000375	RNA splicing, via transesterification reactions	0.023425	0.123525	0.084084	2	Malat1/Ddx17
	GO:0000377	RNA splicing, via transesterification reactions with bulged adenosine as nucleophile	0.023425	0.123525	0.084084	2	Malat1/Ddx17
mRNA processing	GO:0043484	regulation of RNA splicing	0.010438	0.123525	0.084084	2	Malat1/Ddx17
	GO:0000381	regulation of alternative mRNA splicing, via spliceosome	0.00117	0.123525	0.084084	2	Malat1/Ddx17
	GO:0050684	regulation of mRNA processing	0.000275	0.11784	0.080214	3	Malat1/Iws1/Ddx17
	GO:1903311	regulation of mRNA metabolic process	0.002221	0.123525	0.084084	3	Malat1/Iws1/Ddx17
	GO:0008380	RNA splicing	0.000393	0.11784	0.080214	4	Malat1/Iws1/Ddx17/Ccar2
	GO:0006397	mRNA processing	0.000591	0.118238	0.080485	4	Malat1/Iws1/Ddx17/Ccar2
	GO:0043414	macromolecule methylation	0.002663	0.123525	0.084084	3	Iws1/Kcnq1ot1/Wdr5b
	GO:0032259	methylation	0.003758	0.123525	0.084084	3	Iws1/Kcnq1ot1/Wdr5b
	GO:0018205	peptidyl-lysine modification	0.004579	0.123525	0.084084	3	Iws1/Uspl1/Wdr5b
	GO:0034968	histone lysine methylation	0.005734	0.123525	0.084084	2	Iws1/Wdr5b
Methylation	GO:0018022	peptidyl-lysine methylation	0.006921	0.123525	0.084084	2	Iws1/Wdr5b
	GO:0016571	histone methylation	0.009294	0.123525	0.084084	2	Iws1/Wdr5b
	GO:0006479	protein methylation	0.013734	0.123525	0.084084	2	Iws1/Wdr5b
	GO:0008213	protein alkylation	0.013734	0.123525	0.084084	2	Iws1/Wdr5b

Ubiquitin protein ligase	GO:0000209	protein polyubiquitination	0.018784	0.123525	0.084084	2	Cul3/Rnf166
	GO:0061630	ubiquitin protein ligase activity	0.028508	0.080423	0.03848	2	Cul3/Rnf166
	GO:0061659	ubiquitin-like protein ligase activity	0.030463	0.080423	0.03848	2	Cul3/Rnf166
	GO:0004842	ubiquitin-protein transferase activity	0.045754	0.089714	0.042925	2	Cul3/Rnf166
	GO:0031625	ubiquitin protein ligase binding	0.026606	0.080423	0.03848	2	Cul3/Faf2
	GO:0044389	ubiquitin-like protein ligase binding	0.029316	0.080423	0.03848	2	Cul3/Faf2
Gene expression	GO:0031507	heterochromatin formation	0.002469	0.123525	0.084084	2	H1f0/Kcnq1ot1
	GO:0045814	negative regulation of gene expression, epigenetic	0.002924	0.123525	0.084084	2	H1f0/Kcnq1ot1
	GO:0070828	heterochromatin organization	0.002924	0.123525	0.084084	2	H1f0/Kcnq1ot1
	GO:0040029	epigenetic regulation of gene expression	0.009396	0.123525	0.084084	2	H1f0/Kcnq1ot1
	GO:0006338	chromatin remodeling	0.030325	0.124622	0.084831	2	H1f0/Kcnq1ot1
Other	GO:0031647	regulation of protein stability	0.027347	0.123525	0.084084	2	Cul3/Ccar2
	GO:1901987	regulation of cell cycle phase transition	0.049833	0.1394	0.09489	2	Cul3/Ccar2
	GO:0005819	spindle	0.033153	0.153613	0.121273	2	Cul3/Ccar2
	GO:0043161	proteasome-mediated ubiquitin-dependent protein catabolic process	0.005501	0.123525	0.084084	3	Cul3/Faf2/Ccar2
	GO:0016050	vesicle organization	0.029152	0.123525	0.084084	2	Cul3/Hps5
	GO:0003713	transcription coactivator activity	0.018741	0.080423	0.03848	2	Ddx17/Ccar2
	GO:0031047	gene silencing by RNA	0.011197	0.123525	0.084084	2	Ddx17/Kcnq1ot1
	GO:0004857	enzyme inhibitor activity	0.044021	0.089714	0.042925	2	Faf2/Ccar2
	GO:0032182	ubiquitin-like protein binding	0.003629	0.080423	0.03848	2	Faf2/Uspl1
	GO:0030246	carbohydrate binding	0.02095	0.080423	0.03848	2	Gpcpd1/Vcan
	GO:0031490	chromatin DNA binding	0.003758	0.080423	0.03848	2	H1f0/Ddx17
	GO:0051053	negative regulation of DNA metabolic process	0.00737	0.123525	0.084084	2	H1f0/Zfp729a
	GO:0046486	glycerolipid metabolic process	0.039609	0.13817	0.094052	2	Il6st/Gpcpd1
	GO:0010720	positive regulation of cell development	0.039985	0.138676	0.094397	2	Il6st/Runx1
	GO:0001503	ossification	0.048202	0.1394	0.09489	2	Il6st/Runx1
	GO:0019955	cytokine binding	0.006103	0.080423	0.03848	2	Il6st/Vasn
	GO:0019838	growth factor binding	0.006686	0.080423	0.03848	2	Il6st/Vasn
	GO:0042063	gliogenesis	0.036103	0.13254	0.09022	2	Il6st/Vcan
	GO:0048511	rhythmic process	0.026383	0.123525	0.084084	2	Runx1/Ccar2
	GO:0062023	collagen-containing extracellular matrix	0.039439	0.157755	0.124543	2	Runx1/Vcan
	GO:0016607	nuclear speck	0.031485	0.153613	0.121273	2	Malat1/Ddx17

Supplementary Table 11. Enriched GO termed for down-regualted genes in PBMCs of mice immunized with Epitope25-Sub-Q compared with those having Adjuvant-DMNA.

Summarized GO Category	ID	Description	pvalue	p.adjust	qvalue	Count	geneID
	GO:0015671	oxygen transport	0.000175776	0.009715643	0.008225328	2	Hbb-bt/Hba-a1
	GO:0015669	gas transport	0.000660248	0.023613579	0.019991413	2	Hbb-bt/Hba-a1
	GO:0042744	hydrogen peroxide catabolic process	0.001655969	0.04299531	0.036400115	2	Hbb-bt/Hba-a1
	GO:0042743	hydrogen peroxide metabolic process	0.006275514	0.127183753	0.107674611	2	Hbb-bt/Hba-a1
	GO:0005833	hemoglobin complex	0.0002019	0.002851841	0.002204963	2	Hbb-bt/Hba-a1
	GO:0031838	haptoglobin-hemoglobin complex	0.000241978	0.003038166	0.002349025	2	Hbb-bt/Hba-a1
	GO:0031720	haptoglobin binding	0.000192982	0.005114029	0.003182514	2	Hbb-bt/Hba-a1
Oxygen related metabolism	GO:0005344	oxygen carrier activity	0.000318132	0.005620325	0.003497587	2	Hbb-bt/Hba-a1
	GO:0019825	oxygen binding	0.00111969	0.016184617	0.010071857	2	Hbb-bt/Hba-a1
	GO:0004601	peroxidase activity	0.00547772	0.058063833	0.036133732	2	Hbb-bt/Hba-a1
	GO:0016684	oxidoreductase activity, acting on peroxide as acceptor	0.006042247	0.060044825	0.037366525	2	Hbb-bt/Hba-a1
	GO:0140104	molecular carrier activity	0.008551857	0.06798726	0.042309185	2	Hbb-bt/Hba-a1
	GO:0016209	antioxidant activity	0.01170834	0.077567753	0.048271227	2	Hbb-bt/Hba-a1
	GO:0043177	organic acid binding	0.033956294	0.110742859	0.068916443	2	Hbb-bt/Hba-a1
	GO:0020037	heme binding	0.000252194	0.005620325	0.003497587	4	Hbb-bt/Slc48a1/Hba-a1/Hebp1
	GO:0046906	tetrapyrrole binding	0.000310181	0.005620325	0.003497587	4	Hbb-bt/Slc48a1/Hba-a1/Hebp1
	GO:0072593	reactive oxygen species metabolic process	0.013130661	0.204703631	0.173303455	3	Rfk/Hbb-bt/Hba-a1
	GO:0042776	proton motive force-driven mitochondrial ATP synthesis	0.007570535	0.143840167	0.121776042	2	Ndufa2/Atp5b
	GO:0015986	proton motive force-driven ATP synthesis	0.008733785	0.15903636	0.13464124	2	Ndufa2/Atp5b
	GO:0006754	ATP biosynthetic process	0.019222402	0.22518454	0.190642729	2	Ndufa2/Atp5b
	GO:0009206	purine ribonucleoside triphosphate biosynthetic process	0.023114428	0.22518454	0.190642729	2	Ndufa2/Atp5b
	GO:0009145	purine nucleoside triphosphate biosynthetic process	0.023483106	0.22518454	0.190642729	2	Ndufa2/Atp5b
	GO:0009201	ribonucleoside triphosphate biosynthetic process	0.024981998	0.22518454	0.190642729	2	Ndufa2/Atp5b
	GO:0009142	nucleoside triphosphate biosynthetic process	0.028894721	0.22518454	0.190642729	2	Ndufa2/Atp5b
	GO:0006119	oxidative phosphorylation	0.032189554	0.22518454	0.190642729	2	Ndufa2/Atp5b
	GO:0098800	inner mitochondrial membrane protein complex	0.031650554	0.18391188	0.142195491	2	Ndufa2/Atp5b
	GO:0015399	primary active transmembrane transporter activity	0.034775382	0.110742859	0.068916443	2	Ndufa2/Atp5b
ATP biosynthetic process	GO:0009260	ribonucleotide biosynthetic process	0.008893481	0.15903636	0.13464124	3	Rfk/Ndufa2/Atp5b
	GO:0046390	ribose phosphate biosynthetic process	0.009954136	0.168114304	0.142326687	3	Rfk/Ndufa2/Atp5b
	GO:0009165	nucleotide biosynthetic process	0.016011454	0.22518454	0.190642729	3	Rfk/Ndufa2/Atp5b
	GO:1901293	nucleoside phosphate biosynthetic process	0.016824044	0.22518454	0.190642729	3	Rfk/Ndufa2/Atp5b
	GO:0032781	positive regulation of ATP-dependent activity	0.003380624	0.076126651	0.064449329	2	Dnajb1/Napa
	GO:0043462	regulation of ATP-dependent activity	0.009220099	0.160166288	0.135597845	2	Dnajb1/Napa
	GO:0032592	integral component of mitochondrial membrane	0.010902237	0.086048806	0.066530516	2	Ghitm/Fundc2
	GO:0098573	intrinsic component of mitochondrial membrane	0.011422408	0.086048806	0.066530516	2	Ghitm/Fundc2
	GO:0005759	mitochondrial matrix	0.019426495	0.137199622	0.106078888	3	Rexo2/Alas2/Atp5b
	GO:1990542	mitochondrial transmembrane transport	0.012383546	0.198136737	0.167743879	2	Slc25a39/Ucp2
	GO:0006783	heme biosynthetic process	0.001243601	0.0360052	0.030482242	2	Slc25a39/Alas2
	GO:0006779	porphyrin-containing compound biosynthetic process	0.001767899	0.04299531	0.036400115	2	Slc25a39/Alas2
	GO:0033014	tetrapyrrole biosynthetic process	0.001767899	0.04299531	0.036400115	2	Slc25a39/Alas2
Pigment metabolism	GO:0046148	pigment biosynthetic process	0.004907253	0.102883096	0.087101513	2	Slc25a39/Alas2
	GO:0042168	heme metabolic process	5.20E-05	0.004459455	0.003775404	3	Slc25a39/Alas2/Hebp1
	GO:0006778	porphyrin-containing compound metabolic process	8.90E-05	0.006014103	0.005091579	3	Slc25a39/Alas2/Hebp1
	GO:0033013	tetrapyrrole metabolic process	0.000166603	0.009715643	0.008225328	3	Slc25a39/Alas2/Hebp1
	GO:0042440	pigment metabolic process	0.000348903	0.017677739	0.014966092	3	Slc25a39/Alas2/Hebp1
	GO:0022627	cytosolic small ribosomal subunit	0.000106217	0.001714644	0.001325714	3	Hba-a1/Rps12/Uba52
	GO:0015935	small ribosomal subunit	0.000572791	0.006472539	0.005004385	3	Hba-a1/Rps12/Uba52
	GO:0022626	cytosolic ribosome	8.37E-08	8.43E-06	6.52E-06	6	Rpl39/Rplp1/Hba-a1/Rplp0/Rps12/Uba52
	GO:0044391	ribosomal subunit	2.60E-06	7.35E-05	5.68E-05	6	Rpl39/Rplp1/Hba-a1/Rplp0/Rps12/Uba52
Ribosome	GO:0005840	ribosome	3.74E-07	1.41E-05	1.09E-05	7	Rpl39/Rplp1/Hba-a1/Rplp0/Rps12/Uba52/Gm2000
	GO:0002181	cytoplasmic translation	1.13E-05	0.001951953	0.001652537	5	Rpl39/Rplp1/Rplp0/Rps12/Uba52
	GO:0022625	cytosolic large ribosomal subunit	1.49E-07	8.43E-06	6.52E-06	5	Rpl39/Rplp1/Rplp0/Rps12/Uba52
	GO:0015934	large ribosomal subunit	4.20E-06	9.49E-05	7.34E-05	5	Rpl39/Rplp1/Rplp0/Rps12/Uba52
	GO:0003735	structural constituent of ribosome	6.94E-07	3.68E-05	2.29E-05	6	Rpl39/Rplp1/Rplp0/Rps12/Uba52/Gm2000
	GO:0097428	protein maturation by iron-sulfur cluster transfer	0.00040751	0.017929768	0.01517946	2	Isca1/AK157302

Metal cluster binding	GO:0016226	iron-sulfur cluster assembly	0.001243601	0.0360052	0.030482242	2	Isca1/AK157302
	GO:0031163	metallo-sulfur cluster assembly	0.001243601	0.0360052	0.030482242	2	Isca1/AK157302
	GO:0051537	2 iron, 2 sulfur cluster binding	0.00111969	0.016184617	0.010071857	2	Isca1/AK157302
	GO:0051536	iron-sulfur cluster binding	0.007039524	0.062182465	0.038696801	2	Isca1/AK157302
	GO:0051540	metal cluster binding	0.007039524	0.062182465	0.038696801	2	Isca1/AK157302
	GO:0006790	sulfur compound metabolic process	0.029701951	0.22518454	0.190642729	3	Isca1/AK157302/Hbb-bt
	GO:0051604	protein maturation	0.03738376	0.22518454	0.190642729	3	Isca1/AK157302/Metap2
Ubiquitin ligase activity	GO:0070979	protein K11-linked ubiquitination	0.001767899	0.04299531	0.036400115	2	Ube2e3/Ube2c
	GO:0070534	protein K63-linked ubiquitination	0.007570535	0.143840167	0.121776042	2	Ube2e3/Ube2o
	GO:0070936	protein K48-linked ubiquitination	0.000412856	0.017929768	0.01517946	3	Ube2e3/Ube2r2/Ube2c
	GO:0000151	ubiquitin ligase complex	0.004505305	0.046281774	0.035783766	4	Ube2l6/Dcaf12/Rnf11/Ube2c
	GO:0044389	ubiquitin-like protein ligase binding	0.025318116	0.109736733	0.06829032	3	Ube2l6/Uba52/Ube2c
	GO:0004842	ubiquitin-protein transferase activity	1.52E-05	0.000604376	0.00037611	7	Ube2l6/Ube2e3/Ube2o/Rnf11/Ube2r2/Trim58/Ube2c
	GO:0019787	ubiquitin-like protein transferase activity	2.13E-05	0.000678563	0.000422277	7	Ube2l6/Ube2e3/Ube2o/Rnf11/Ube2r2/Trim58/Ube2c
	GO:0000209	protein polyubiquitination	1.22E-05	0.001951953	0.001652537	6	Ube2l6/Ube2e3/Ube2o/Ube2r2/Trim58/Ube2c
	GO:0061631	ubiquitin conjugating enzyme activity	1.13E-08	1.49E-06	9.26E-07	5	Ube2l6/Ube2e3/Ube2o/Ube2r2/Ube2c
	GO:0061650	ubiquitin-like protein conjugating enzyme activity	1.87E-08	1.49E-06	9.26E-07	5	Ube2l6/Ube2e3/Ube2o/Ube2r2/Ube2c
	GO:0061630	ubiquitin protein ligase activity	0.024347904	0.109736733	0.06829032	3	Ube2o/Rnf11/Trim58
	GO:0061659	ubiquitin-like protein ligase activity	0.026711199	0.109736733	0.06829032	3	Ube2o/Rnf11/Trim58
	GO:0006513	protein monoubiquitination	0.013529229	0.205644286	0.174099819	2	Ube2o/Ube2r2
Development	GO:0030218	erythrocyte differentiation	1.28E-05	0.001951953	0.001652537	5	Smap1/Alas2/Hba-a1/Trim58/Hba-a2
	GO:0034101	erythrocyte homeostasis	1.86E-05	0.002264245	0.001916925	5	Smap1/Alas2/Hba-a1/Trim58/Hba-a2
	GO:0002262	myeloid cell homeostasis	5.87E-05	0.004459455	0.003775404	5	Smap1/Alas2/Hba-a1/Trim58/Hba-a2
	GO:0048872	homeostasis of number of cells	0.000927537	0.03133014	0.026524305	5	Smap1/Alas2/Hba-a1/Trim58/Hba-a2
	GO:0030099	myeloid cell differentiation	0.001971125	0.046093994	0.039023482	5	Smap1/Alas2/Hba-a1/Trim58/Hba-a2
	GO:0048821	erythrocyte development	2.64E-06	0.001607848	0.001361215	4	Alas2/Hba-a1/Trim58/Hba-a2
	GO:0061515	myeloid cell development	4.38E-05	0.0044357	0.003755293	4	Alas2/Hba-a1/Trim58/Hba-a2
	GO:0001824	blastocyst development	0.041494402	0.240562568	0.203661869	2	Ndufa2/Mxi1
Enzyme regulatory activity	GO:0061135	endopeptidase regulator activity	0.009822294	0.070988399	0.044176826	3	Psme3/Cst3/Ube2o
	GO:0061134	peptidase regulator activity	0.014175603	0.090156832	0.056105543	3	Psme3/Cst3/Ube2o
	GO:0004869	cysteine-type endopeptidase inhibitor activity	0.009713219	0.070988399	0.044176826	2	Cst3/Ube2o
	GO:0004857	enzyme inhibitor activity	0.044423278	0.115471391	0.071859058	3	Cst3/Ube2o/Oaz1
Response to interleukin-7	GO:0098760	response to interleukin-7	0.000526464	0.020005636	0.016936905	2	Atp5b/Stip1
	GO:0098761	cellular response to interleukin-7	0.000526464	0.020005636	0.016936905	2	Atp5b/Stip1
Adaptive therogenesis	GO:0120161	regulation of cold-induced thermogenesis	0.035188076	0.22518454	0.190642729	2	Ucp2/Plcl2
	GO:0106106	cold-induced thermogenesis	0.036064089	0.22518454	0.190642729	2	Ucp2/Plcl2
	GO:1990845	adaptive thermogenesis	0.043369019	0.240562568	0.203661869	2	Ucp2/Plcl2
Exonuclease activity	GO:0008408	3'-5' exonuclease activity	0.003630362	0.044402118	0.027631904	2	Rexo2/Trir
	GO:0004527	exonuclease activity	0.008551857	0.06798726	0.042309185	2	Rexo2/Trir
Kinase activity	GO:0019209	kinase activator activity	0.001833241	0.024290442	0.015116197	3	Itsn1/Rplp1/Pim1
	GO:0019207	kinase regulator activity	0.01150297	0.077567753	0.048271227	3	Itsn1/Rplp1/Pim1
	GO:0030295	protein kinase activator activity	0.02195363	0.109736733	0.06829032	2	Rplp1/Pim1
Other	GO:0043197	dendritic spine	0.006976566	0.064885906	0.050167957	3	Cript/Itsn1/Dnajb1
	GO:0044309	neuron spine	0.00746475	0.064885906	0.050167957	3	Cript/Itsn1/Dnajb1
	GO:0006979	response to oxidative stress	0.048279156	0.25000475	0.211655684	3	Cst3/Ucp2/Hba-a1
	GO:0060090	molecular adaptor activity	0.049298727	0.120592272	0.075045836	3	Dcaf12/Itsn1/Napa
	GO:0030544	Hsp70 protein binding	0.004261136	0.048394326	0.030116297	2	Dnajb1/Stip1
	GO:0051087	chaperone binding	0.018339112	0.109736733	0.06829032	2	Dnajb1/Stip1
	GO:0031072	heat shock protein binding	0.030367398	0.109736733	0.06829032	2	Dnajb1/Stip1
	GO:0043209	myelin sheath	6.20E-05	0.001167372	0.000902579	5	Hba-a1/Uba52/Atp5b/Napa/Stip1
	GO:0015833	peptide transport	0.02642318	0.22518454	0.190642729	3	Itsn1/Slc25a39/Ucp2
	GO:0042886	amide transport	0.033908424	0.22518454	0.190642729	3	Itsn1/Slc25a39/Ucp2
	GO:0051865	protein autoubiquitination	0.011282946	0.185406241	0.156966157	2	Rnf11/Trim58
	GO:0043021	ribonucleoprotein complex binding	0.042041335	0.115251246	0.07172206	2	Rplp1/Pim1
	GO:0072583	clathrin-dependent endocytosis	0.00369683	0.080274033	0.06796053	2	Smap1/Itsn1
	GO:0006898	receptor-mediated endocytosis	0.017659468	0.22518454	0.190642729	3	Smap1/Itsn1/Atp5b
